# Genome-wide DNA methylation analysis reveals estrogen-mediated epigenetic repression of metallothionein-1 gene cluster in breast cancer

**DOI:** 10.1186/s13148-015-0045-9

**Published:** 2015-02-24

**Authors:** Rohit R Jadhav, Zhenqing Ye, Rui-Lan Huang, Joseph Liu, Pei-Yin Hsu, Yi-Wen Huang, Leticia B Rangel, Hung-Cheng Lai, Juan Carlos Roa, Nameer B Kirma, Tim Hui-Ming Huang, Victor X Jin

**Affiliations:** Department of Molecular Medicine/Institute of Biotechnology, University of Texas Health Science Center at San Antonio, STRF, Room 225, 7703 Floyd Curl Drive, San Antonio, 78229 TX USA; Department of Obstetrics and Gynecology, Taipei Medical University Shuang Ho Hospital, New Taipei City, 23561 Taiwan; School of Medicine, Taipei Medical University, No. 250, Wu-Hsing Street, Taipei, 110 Taiwan; Graduate Institute of Life Sciences, Department and Graduate Institute of Biochemistry, Tri-Service General Hospital, National Defense Medical Center, Taipei, Taiwan; Department of Obstetrics and Gynecology, Medical College of Wisconsin, 9200 West Wisconsin Avenue, Froedtert Medical College Lab Building (FMCLB) 258, Milwaukee, 53226 WI USA; Departamento de Pathologı’a, Universidad de la Frontera, Claro Solar 115, Temuco, Chile; Department of Pharmaceutical Sciences, Biotechnology Program/RENORBIO, Health Sciences Center, Universidade Federal do Espirito Santo, Av. Marechal Campos, 1468, Maruipe, 29040-090 Vitoria ES Brazil; Programa Ciencias Sem Fronteiras, CNPq, Brasilia, Brazil; Cancer Therapy and Research Center, University of Texas Health Science Center, 7703 Floyd Curl Drive, San Antonio, 78229 TX USA; Department of Epidemiology and Biostatistics, University of Texas Health Science Center, 7703 Floyd Curl Drive, San Antonio, 78229 TX USA

**Keywords:** DNA methylation, MT1

## Abstract

**Background:**

Recent genome-wide analysis has shown that DNA methylation spans long stretches of chromosome regions consisting of clusters of contiguous CpG islands or gene families. Hypermethylation of various gene clusters has been reported in many types of cancer. In this study, we conducted methyl-binding domain capture (MBDCap) sequencing (MBD-seq) analysis on a breast cancer cohort consisting of 77 patients and 10 normal controls, as well as a panel of 38 breast cancer cell lines.

**Results:**

Bioinformatics analysis determined seven gene clusters with a significant difference in overall survival (OS) and further revealed a distinct feature that the conservation of a large gene cluster (approximately 70 kb) metallothionein-1 (MT1) among 45 species is much lower than the average of all RefSeq genes. Furthermore, we found that DNA methylation is an important epigenetic regulator contributing to gene repression of MT1 gene cluster in both ERα positive (ERα+) and ERα negative (ERα−) breast tumors. *In silico* analysis revealed much lower gene expression of this cluster in The Cancer Genome Atlas (TCGA) cohort for ERα + tumors. To further investigate the role of estrogen, we conducted 17β-estradiol (E2) and demethylating agent 5-aza-2′-deoxycytidine (DAC) treatment in various breast cancer cell types. Cell proliferation and invasion assays suggested MT1F and MT1M may play an anti-oncogenic role in breast cancer.

**Conclusions:**

Our data suggests that DNA methylation in large contiguous gene clusters can be potential prognostic markers of breast cancer. Further investigation of these clusters revealed that estrogen mediates epigenetic repression of MT1 cluster in ERα + breast cancer cell lines. In all, our studies identify thousands of breast tumor hypermethylated regions for the first time, in particular, discovering seven large contiguous hypermethylated gene clusters.

**Electronic supplementary material:**

The online version of this article (doi:10.1186/s13148-015-0045-9) contains supplementary material, which is available to authorized users.

## Background

Aberrant epigenetic changes, including DNA methylation and histone modifications, have been known to be the hallmark of cancer [1]. These changes usually disrupt the regulation of many oncogenes or tumor suppressor genes in tumors, resulting in their abnormal expression. DNA methylation occurs mainly at CpG-rich ‘CpG islands’ and surrounding ‘CpG-shore’ regions, where more than 60% of them are located in 5′ promoters [2]. *De novo* hypermethylation of these regions, which is often associated with silencing of many tumor suppressor genes, has been shown to play a crucial role in the development of many types of human cancers [3-6]. Many studies, including ours [7-10], used a quantitative approach based on statistical methods or machine learning algorithms to quantify methylation differences and identify differentially methylated regions (DMRs) from genome-wide methylation profiles in many different tissue or cancer patient cohorts. Such quantitative approaches are thus able to provide more insights into the role of DNA methylation in the development of various diseases such as cancer.

Recent genome-wide analysis of DNA methylation has revealed that this epigenetic process is not only a site specific event but also spans long stretches of chromosome regions consisting of clusters of contiguous CpG islands [11,12] or a gene family [13-15]. Extensive hypermethylation of various gene clusters has previously been reported. For example, hypermethylation of HOXA gene clusters was found in breast and lung cancers [16,17], protocadherin (PCDH) in Wilms’ tumor, the region across chromosome 2q14.2 in colorectal cancer and many others [18-20]. The findings in all these studies warrant a novel gene cluster centric approach towards the investigation of DNA methylation. In an effort to further investigate the mechanism responsible in this long-range epigenetic silencing (LRES), our laboratory previously elucidated the role of estrogen in coordinate repression of these gene clusters in breast cancer [21]. The study revealed that persistent estrogen-mediated LRES leads to recruitment of H3K27me3 repressive chromatin marks, which are accompanied by accumulation of DNA methylation in a gene cluster located at 16p11.2.

In this study, we conducted MBDCap sequencing (MBD-seq) analysis on a breast cancer cohort consisting of 77 patients and 10 normal controls, as well as a panel of 38 breast cancer cell lines. Survival analysis conducted on 60 unique gene clusters determined seven clusters with a significant difference in overall survival (OS) by using methylation levels of genes in the cluster for all patients. Bioinformatics analysis further revealed a distinct feature that the conservation of a large gene cluster (approximately 70 kb) metallothionein-1 (MT1) among 45 species is much lower than the average of all RefSeq genes. We also found that DNA methylation is an important factor contributing to gene repression of MT1 gene cluster regardless of the ERα status. *In silico* analysis using the public domain The Cancer Genome Atlas (TCGA) data revealed that ERα positive (ERα+) breast cancer patients show lower levels of expression for MT1 genes. To investigate if estrogen regulates repression of MT1 cluster in ERα + breast cancer cell types, we conducted 17β-estradiol (E2) and demethylating agent 5-Aza-2′-deoxycytidine (DAC) treatment in various breast cancer cell lines. Our data suggested that both estrogen and DNA methylation mediate repression of the MT1 gene cluster in ERα + breast cancer cell lines. Cell proliferation and invasion assays suggested MT1F and MT1M may have anti-oncogenic roles in breast cancer.

## Results

### MBD-seq identifies differential methylated patterns in breast primary tumors

**Figure 1:**
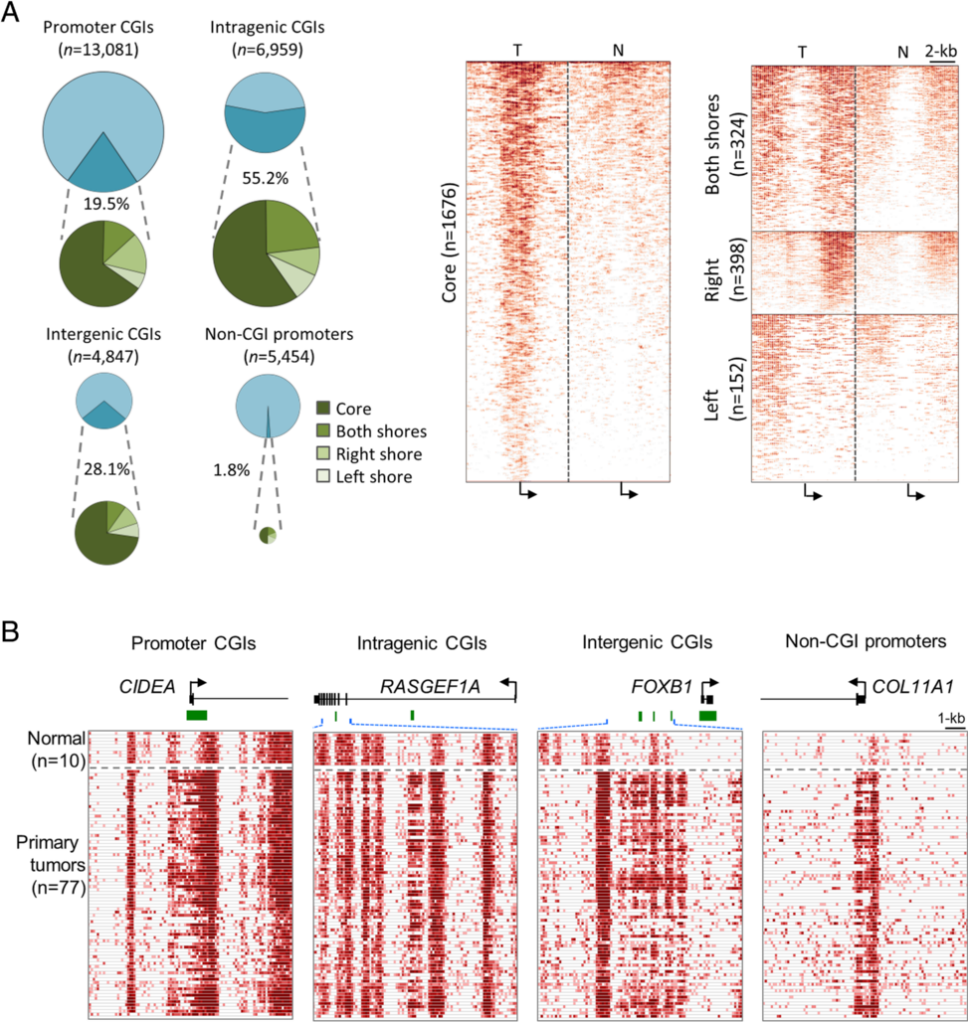
**DNA hypermethylation in breast cancer samples relative to normal breast tissue in promoter and non-promoter CpG islands.** Methyl capture sequencing (MBD-seq) was used to generate DNA methylation profiles of the genomes of breast tumors (*n* = 77) and normal breast tissue (*n* = 10). **(A)** Pie charts demonstrate differential methylation in promoter, intragenic, and intergenic CGIs as well as non-CGI promoter regions (left panel). Methylation of core and shore regions is also demonstrated under each corresponding CGI type (left panel). DNA hypermethylation in core and shore regions are shown for breast tumors compared to normal tissue (right panel). **(B)** Example loci showing promoter CGI, intragenic, intergenic, and non-CGI promoter regions. Dashed squares highlight regions corresponding to breast cancer hypermethylation.

We conducted MBD-seq to investigate methylation patterns on a genome-wide scale for a cohort of breast cancer patients (*n* = 77), Integrative Cancer Biology Program (ICBP) breast cancer cell lines (*n* = 38), and normal mammary tissue (*n* = 10). Over 20 million unique reads were analyzed for all samples, a coverage expected to provide sufficient sequence depth for methylation mapping of the whole genome (Additional file 1: Figure S1). Differential methylation between breast tumor and normal control samples (Additional file 1: Table S3) was observed in promoter CpG islands (CGIs) (19.5% of 13,081 promoter CGIs analyzed; *P* < 0.05), as well as intragenic CGIs (55.2% of 6,959 intragenic CGIs analyzed; *P* < 0.01), intergenic CGIs (28.1% of 4,847 intergenic CGIs analyzed; *P* < 0.01), and non-CGI promoters (1.8% of 5,454 non-CGI promoters; *P* < 0.01) (Figure 1A, left panel). DNA hypermethylation in breast tumors compared to normal control occurred predominantly in CGI cores at transcription start site (TSS) regions and also in regions flanking CGIs, or so-called CpG shores (Figure 1A, right panel). This distinct type of methylation pattern was first reported in colon cancer [22]. Examples of loci exhibiting hypermethylation in breast tumor samples relative to normal tissue in promoter CGIs (CIDEA), intragenic CGIs (*RASGEF1A*), intergenic CGIs (*FOXB1*), and non-CGI promoters (*COL11A1*) are presented in Figure 1B (hypermethylated regions are outlined by dashed squares) and in Additional file 1: Figure S2.

### Survival analysis determines significant hypermethylated gene clusters in breast cancer

**Figure 2:**
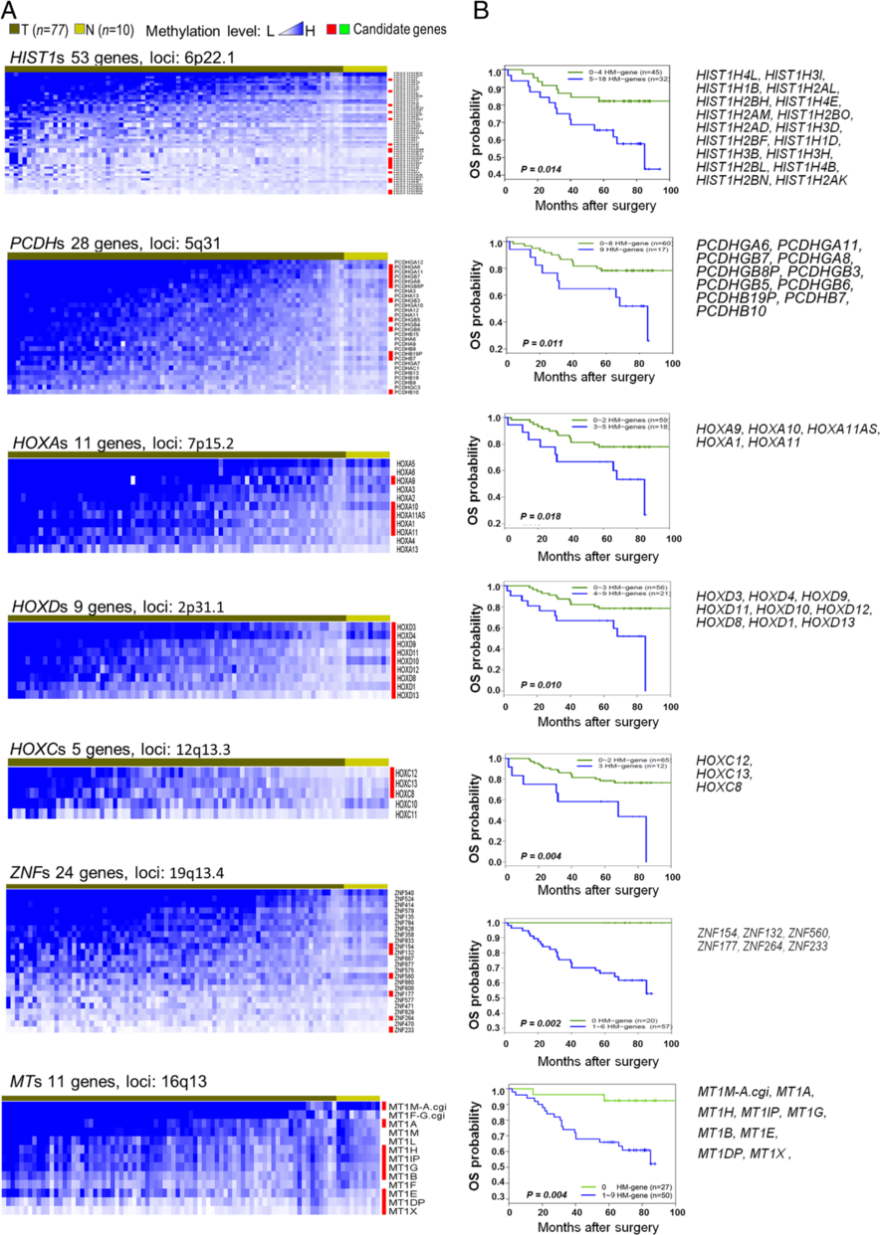
**Gene clusters hypermethylated in breast cancer and associated with poor survival. (A)** Heat maps showing averaged DNA methylation in the different gene cluster loci (left panel). **(B)** Kaplan-Meier survival curves indicating candidate cluster gene hypermethylation are associated with decreased survival (right panel).

Recent studies including ours [11-13] found that DNA methylation patterns span long stretches of chromosome regions that mostly consist of gene clusters in different types of cancer. We therefore examined hypermethylated levels of more than 60 unique gene clusters in our breast cohort (77 tumor vs. 10 normal), where hypermethylation in the breast tumors was defined in relative terms to DNA methylation in normal breast tissue, and then performed survival analysis to determine their significance. Survival analysis, represented by Kaplan-Meier curves, was conducted using the third quartile as the cutoff value to dichotomize patients into high- and low-methylation groups (Figure 2A). We found that methylation levels of 38 of these unique gene clusters were significantly correlated with OS, showing that these gene clusters’ hypermethylated levels were a high risk factor and positively correlated with a poor survival (Figure 2B) (Additional file 2). We further selected seven gene clusters from these which have been reported in the literature to have biological functions and protein domains associated with estrogen interactions and/or cancer development for further investigation [23-28]. Our analyses also determined that most but not all of the hypermethylated genes in a particular gene cluster were able to be included in stratifying patients with a statistical significance, implying that these excluded few genes in the cluster may exhibit a more distinct functional role. For example, 9 of 11 metallothionein-1 (MT1) genes (MT1A, B, E, G, H, L, and X), including two hypothetical genes MT1DP and MT1IP, were able to predict a poor survival with a *P* = 0.004. MT1F and MT1M, which are not in the list, exert anti-oncogenic effects (see last section in Results).

### Bioinformatics analysis reveals a distinct feature of the MT1 gene cluster

We further performed the bioinformatics analysis on these seven gene clusters in order to gain an insight into their underlying characterization, such as guanine-cytosine (GC) contents and phylogenetic conservations. As expected, the highest mean GC contents are around 5′TSS regions for all gene clusters, except PCDH gene family, with a peak around downstream 2 kb of 5′TSS while two gene clusters, HIST1 and PCDH, showed lower mean GC contents than the average of all RefSeq genes (Additional file 1: Figure S9A). Plots of phylogenetic conservation among 45 species (Additional file 1: Figure S9B) showed that phastCons scores of the MT1 gene cluster are below 0.1 at 5′TSS which is lower than the average of all RefSeq genes. Two relatively high conserved regions (two peaks downstream 5′TSS) might be the first exons. Our analysis is in line with a finding that the evolution of the lineage that led to human MT1 has undergone further duplication events that have resulted in 13 younger duplicate isoforms [29] and the divergence of the MT family in mammals. We also observed the ZNF gene cluster that has relatively lower scores of 0.2 but at the same level as the average.

**Figure 3:**
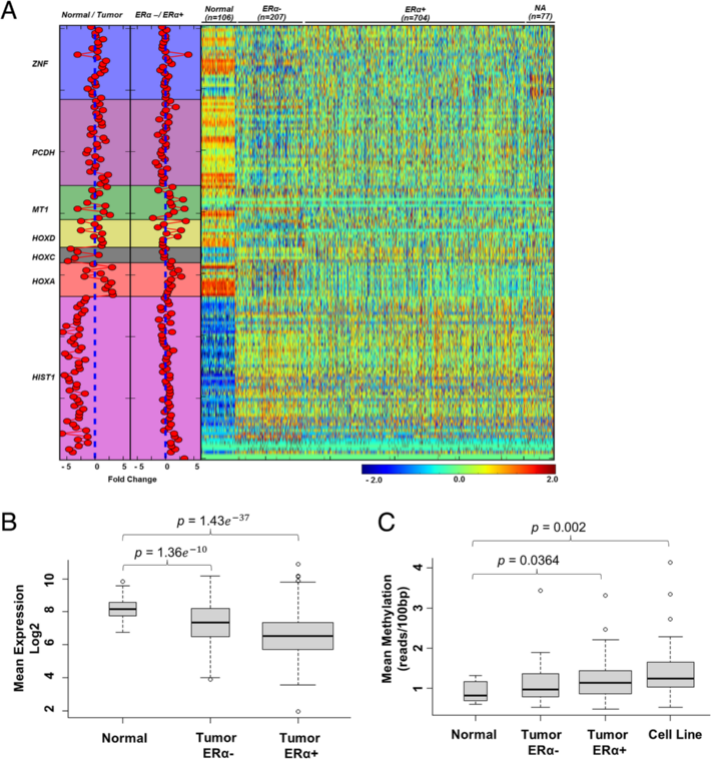
**Lower expression and higher methylation of MT1 gene cluster for ERα** **+ compared to ERα − and normal patient samples is observed in TCGA RNA-seq and our MBD-seq cohort, respectively. (A)** A heat map showing RNA-seq expression data from TCGA for all the genes in identified clusters along with fold change comparisons (left) with normal vs. tumor and ERα − vs. ERα + samples. **(B)** A boxplot showing the significant difference in average gene expressions in each subset compared to normal for all the genes in MT1 gene cluster. **(C)** A boxplot showing significant difference in average MT1 cluster gene methylation for each subset compared to normal samples in our MDB-seq cohort.

**Figure 4:**
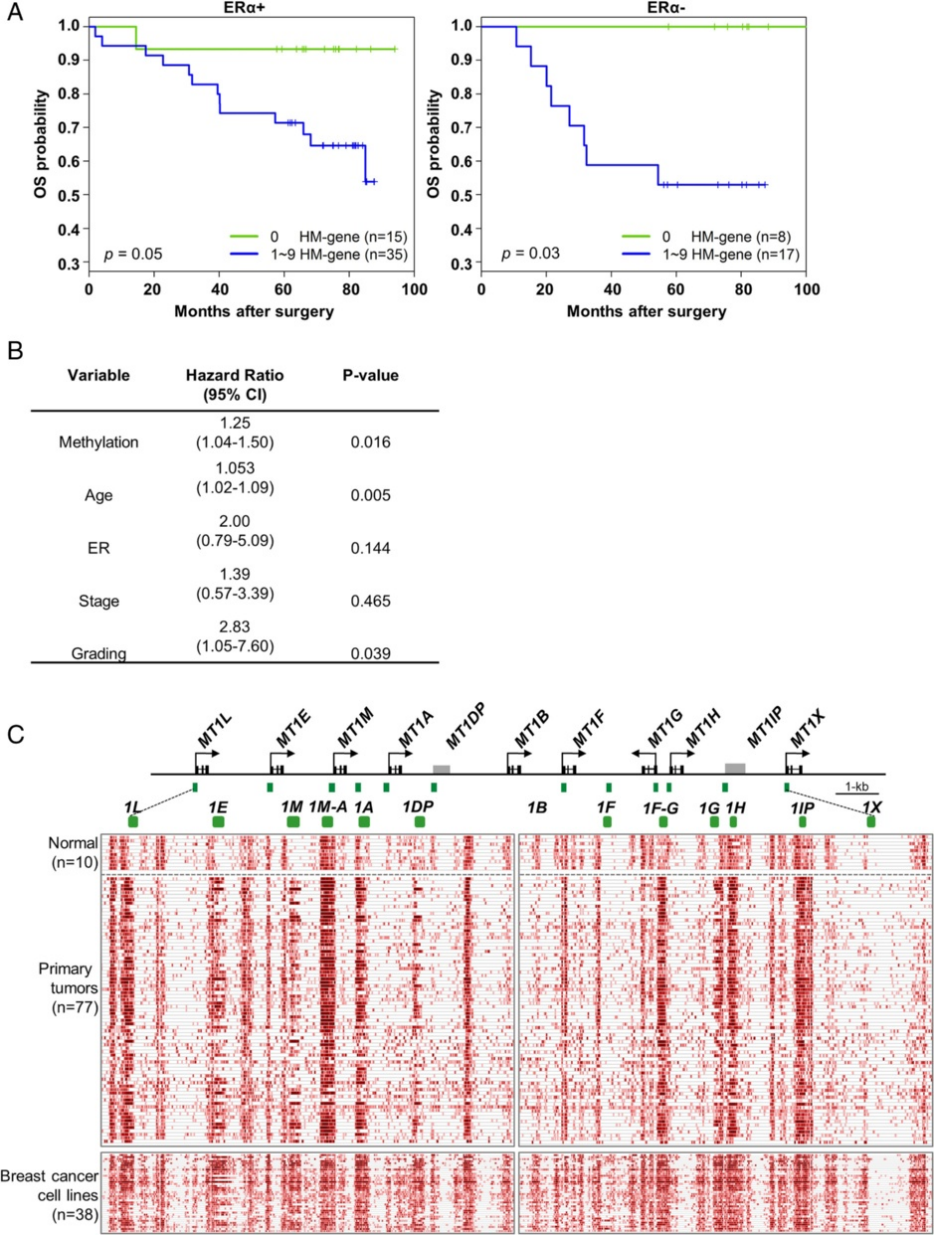
**DNA methylation is associated with poor survival in MT1 gene cluster regardless of ERα** **status of patients. (A)** Kaplan-Meier survival curves indicating that MT1 gene cluster hypermethylation is associated with decreased survival in both ERα + and ERα − patients. **(B)** A table showing the hazard ratios along with 95% confidence intervals and statistical significance for methylation along with other covariates. The groups are the same as the ones used for Figure 4A and MT1 cluster in Figure 2B, that is, patients showing hypermethylation in one to nine MT1 genes in the cluster are grouped together. **(C)** A heat map showing methylation of MT1 gene cluster for normal breast tissue, breast tumors, and breast cancer cell lines. Dashed-line squares highlight differentially methylated regions in these samples. Note that MT1 gene cluster consists of nine loci (MT1L, E, M, A, B, F, G, H, and X) and two pseudogenes (MT1DP and IP) as outlined in the genomic map above the methylation profile.

Next, we utilized the publicly available TCGA breast cancer cohort, including 106 normal tissue and 988 primary tumor samples, and examined the expression values measured by RNA-seq data for these hypermethylated gene clusters. Interestingly, we found five gene clusters, ZNF, PCDH, MT1, HOXD, and HOXA, which showed reduced expression in cancer patients compared to normal tissues. However, the other two clusters, HOXC and HIST1, surprisingly showed increased expression levels (Figure 3A). Although this latter observation is inconsistent with a traditional view that the promoter hypermethylated genes in tumors are usually positively correlated with lower expression, this supported a newly established concept that the methylated status of other gene regions, such as intragenic and 3′TTS, may also play a role in determining the overall expression as demonstrated by many studies [30-32]. Nevertheless, for the first time, our findings provide a correlation between methylation status and gene expression level at a gene cluster scale. A further detailed examination of the MT1 gene cluster revealed that the ERα + tumor samples have a much lower gene expression level than ERα negative (ERα−) tumor samples while both display significantly lower gene expression level than normal tissue samples (Figure 3B). However, their methylation levels showed a decrease in an order of ERα + tumor, ERα − tumor, and normal samples (Figure 3C). This positive correlation prompted us to ask if the epigenetic repression of this gene cluster is associated with the status of ERα level (positive vs. negative) in the breast tumors. To this end, we re-examined K-M survival analysis based on the status of ERα level and found that both ERα + and ERα − patients show poor outcomes for the hypermethylated MT1 genes (Figure 4A). A Cox proportional hazard regression model further confirmed that the hazard ratios show statistical significance for methylation, age, and grading but not for ERα status (Figure 4B). Most of the individual genes in the cluster also showed a significant negative correlation between DNA methylation and gene expression in both ERα + and ERα − patient samples (Additional file 1: Figure S10). This data supported a notion that DNA methylation is an important factor contributing to gene repression regardless of the estrogen receptor (ER) status in breast tumors.

### Hypermethylation of the MT1 gene cluster is validated in different breast cancer cell lines

As the hypermethylated status for the MT1 gene cluster was observed in primary tumor samples, we further hypothesized that they would also be hypermethylated in breast cancer cell lines since these cell lines were isolated, cultured, and homogenized from the primary tumor. In order to confirm this hypothesis, we conducted the MBD-seq analysis on a panel of 38 breast cancer cell lines (Additional file 1: Figure S1). Overall, the mean methylation level for six of the gene clusters in the cell lines was higher than that in normal tissue samples but lower than that in tumor samples (Additional file 1: Figure S11). For the MT1 gene cluster, we found that the cell line data had the highest methylation levels followed by ERα + tumor, ERα − tumor, and normal samples (Figure 3C). A detailed visualized analysis along the MT1 gene cluster further revealed a hypermethylation pattern in CGIs that was associated with most MT1 TSS sites in the breast cancer samples relative to normal breast tissue (Figure 4C). Specifically, hypermethylation was observed in promoter CGIs of MT1L, E, M, A, G, and H as well as non-promoter CGIs M-A and F-G (Figure 4C; hypermethylated regions are denoted by dashed squares). The visualization of other gene clusters is shown in Additional file 1: Figures S3-8. Taken together, our result validated the hypermethylated status of the MT1 gene cluster in many different breast cancer cell lines.

**Figure 5:**
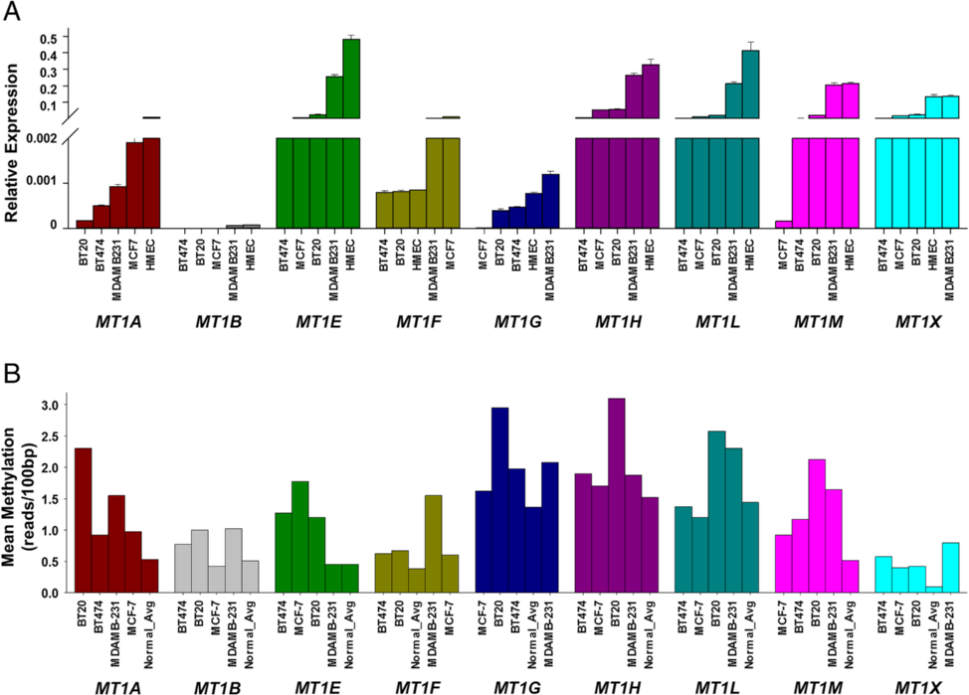
**Basal level expression and DNA methylation for most of the genes in MT1 gene cluster show lower expression and higher methylation in breast cancer cell lines. (A)** RT-qPCR gene expression and **(B)** averaged methylation profiles of MT1 genes in cell lines representing ERα + (MCF7 and BT474) and ERα − (BT20 and MDAMB231) subsets compared to normal (HMEC used for PCR and average of normal samples for DNA methylation) revealing lower levels of expression and higher levels of DNA methylation in cell lines compared to normal.

Next, we performed quantitative reverse transcription PCR (RT-qPCR) to examine the gene expression level for the MT1 gene cluster in selected breast cell lines, including one normal cell line, human mammary epithelial cells (HMEC), two ERα + cell lines, MCF7, BT474, and two ERα − cell lines, BT20 and MDA-MB231. Although there are publicly available gene expression data in 61 breast cancer cell lines [33], the cell lines we used for profiling methylation are not completely overlapping with them, and some of the genes in our study were not in the profiling. As shown in Figure 5A, overall, all genes showed lower expression in four breast cancer cell lines than in the HMEC cell line, except MT1F and MT1X, where MT1F has lower in HMEC than in MCF7 and MDA-MB231 cells, and MT1X has a similar level in MDA-MB231 cells. Meanwhile, there are no clear differences for each individual gene in two ERα − vs. two ERα + breast cancer cell lines. This may be due to the cell model not fully recapitulating the molecular characteristics of the primary tumors. We also found that there are no detectable expression levels for MT1B in all cell lines. This is consistent with the TCGA data showing no expression for this gene in all patients and normal samples. By examining the methylation levels in these cell lines (Figure 5B), we found that all genes showed higher methylation in breast cancer cell lines than normal tissue while we did not find a clear differential pattern between ERα + and ERα − cell lines. These validations further support our earlier observation that the correlation of lower expression with hypermethylation for this gene cluster is independent of the status of ERα in the breast tumors.

### Estrogen mediates epigenetic repression of the MT1 gene cluster in ERα + breast cancer cell lines

**Figure 6:**
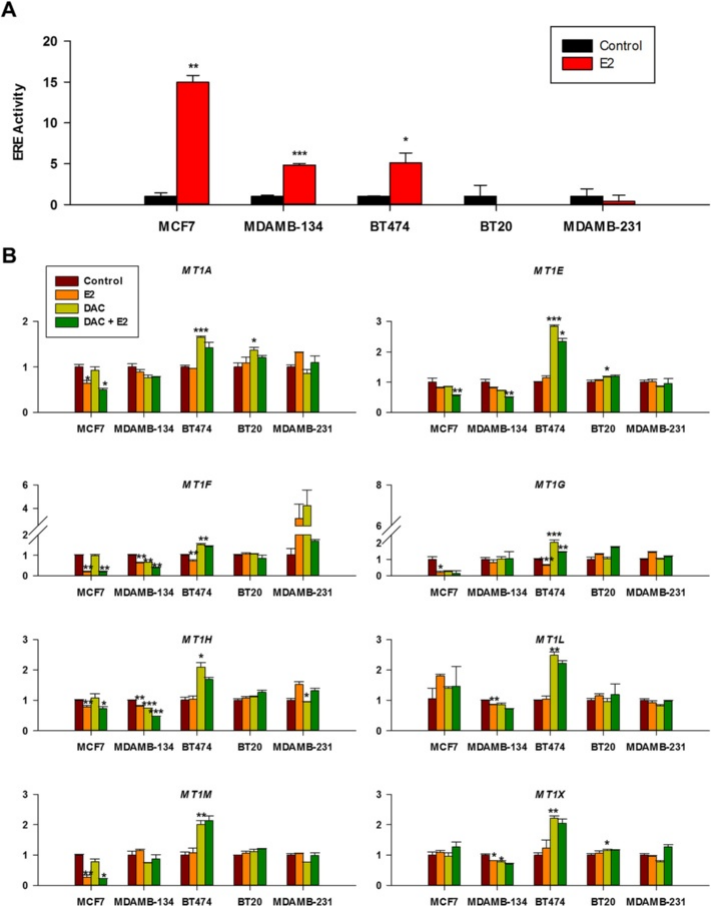
**Differential response to E2 suppression and DAC activation of MT1 gene expression is seen in ERα + cell lines but not in ERα − cell lines. (A)** Luciferase activity of ERE reporter vector normalized to Renilla reporter activity in luminal and basal breast cancer cell lines. **(B)** MT1 expression was carried out by RT-qPCR following E2 and/or DAC treatments as described in the Methods section.

To further investigate whether estrogen mediates epigenetic repression of the cluster in ERα + breast cancer cell lines, we conducted E2 and DAC treatment in various breast cancer cell types. We first used estrogen response element (ERE) luciferase assay to confirm that ERE responds to E2 in ERα + cell lines, MCF7, MDAMB134, and BT474, and not in ERα − cell lines, BT20 and MDA-MB-231 (Figure 6A). We then examined the differential gene expression of eight genes in the cluster before and after the E2 and DAC treatment (Figure 6B). We removed MT1B from this experiment as there was no detectable expression level in the selected cell lines. Surprisingly, for MCF7, a ERα + cell line, the expression level for six genes (MT1A, F, H, M, E, and G) significantly decreased in response to E2 or/and DAC treatment, while no changes were observed for two of them (MT1L and X). Similarly, MDAMB134 which is ERα + cell line but with a slightly lower ERE activity compared to MCF7 showed a marked decrease in expression of five genes (MT1E, F, H, L, and X). However, for BT474, another ERα + cell line, we found that expression levels showed no significant changes for six genes after E2 treatment but significantly increased upon DAC treatment for all eight genes. We also observed that the expression levels for the combination of E2 and DAC treatment are similar to those of E2 treatment. We did not detect any expression changes after E2 or/and DAC treatment in both ERα − cell lines. The distinct expression response of BT474 compared to other ERα + cell lines may be attributed to the altered ER signal transduction pathway which also contributes to its tamoxifen resistance as reported by Wang *et al.* [34]. Clearly, more experiments are needed to further explore the underlying mechanism; our data nevertheless suggest that estrogen plays an important role in mediating epigenetic repression (mainly DNA hypermethylation) of the MT1 gene cluster in ERα + breast cancer cell lines.

### MT1F and MT1M expression exerts anti-oncogenic effects in breast cancer

**Figure 7:**
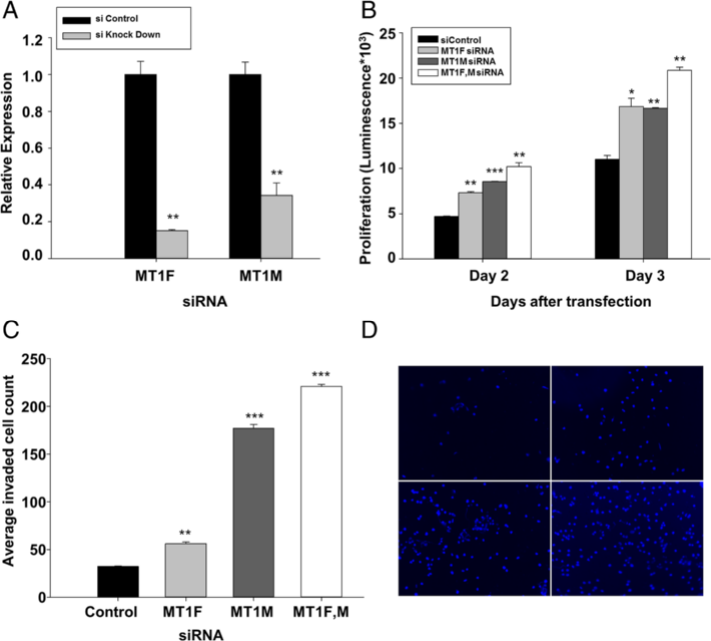
**MT1F and MT1M knockdown leads to increased cellular proliferation and invasion in MCF-7 cells.** Knockdown as well as cellular proliferation **(A, B)** and invasion **(C, D)** assays were carried out as described in the Methods section.

Since MT1 gene clusters were hypermethylated in breast tumors from our cohort and the methylation of the gene cluster was associated with poor survival, we hypothesize that MT1 genes may play a functional role in breast tumorigenesis. In order to test this hypothesis, we selected two genes MT1F and MT1M to further investigate whether knockdown of their expression can impart a more aggressive phenotype to MCF7 cells. The MT1M gene was selected because it is highly methylated in ERα + cells and has been implicated as a tumor suppressor in earlier studies [35], while the selection of MT1F is because it is most highly expressed in MCF7 cells compared to other cell lines (Figure 5A). In addition, both MT1F and MT1M were highly epigenetically repressed by E2 in MCF7 cells (Figure 6B). After sufficiently knocking down MT1F and MT1M in MCF7 cells transfected with specific siRNA for either MT1F or MT1M (Figure 7A and Additional file 1: Table S5), we examined the proliferation and invasion of MCF7 cells. Knockdown of MT1F and MT1M resulted in an increased proliferation in MCF7 cells compared to control siRNA (Figure 7B). Furthermore, knockdown of MT1F and MT1M increased the invasiveness of MCF7 cells, and the combination knockdown had an additive invasive effect (Figure 7C,D). Taken together, we demonstrated that MT1F and MT1M exert anti-oncogenic effects in MCF7 cells.

## Discussion

Our current studies quantitatively analyzed differential methylation patterns at a genome-wide scale on a breast patient cohort and identified many large contiguous hypermethylated regions mainly consisting of gene clusters. Our results re-assert a newly emerging perspective that DNA methylation goes beyond a discrete gene event and often spans long stretches of chromosome regions. Although this phenomenon has lately been observed by several other studies in different types of cancer [12-14,16,17], this study reported for the first time that the tumor hypermethylation levels of a gene cluster (as many as seven gene clusters) (Figure 2) are significantly associated with overall survival in breast cancer patients. More strikingly, the hypermethylation status for seven clusters identified in the patient cohort was recapitulated in a panel of 38 breast cancer cell lines using the same MBD-seq protocol. Since the selection of cell lines, which includes several sub-types of breast cancer, is purely based on the availability at the time conducting the experiments, our cell line data not only validate our patient data but also further suggest that this large contiguous hypermethylation across seven gene clusters may be a distinguishing characteristic of breast cancer and commonly exist across many different sub-types.

Our analysis of intrinsic genomic features on these gene clusters revealed a distinct feature of the MT1 gene cluster. Compared to other gene clusters, such as HOXA, HOXC, HOXD, HISTI1, and ZNF, which are highly conserved among 45 species, the conservation for MT1 cluster is lower and even much below the average of all RefSeq genes. Although an evolutionary study indicates that the MT1 family is mammal-specific with 13 new isoforms in humans, an earlier study investigating promoter DNA methylation has reported one of the MT1 genes (MT1G) to be associated with breast cancer progression [36]. Another study indicated that the expression of the genes in this cluster led to poor overall survival in a subset of invasive breast cancer patients [25]. How these genomic properties affect the biological functions and biological processes of the genes in this cluster, in relation with specific organs, as well as its role in cancer development and progression, is worth further exploration. Interestingly, our correlation analysis of the methylation levels with the gene expression levels using a larger TCGA breast cancer cohort data found that the MT1 gene cluster exerts clear differential expression patterns among ERα + tumor samples and ERα − tumor samples (Figure 3B). Although the HOXD gene cluster showed a similar pattern, this cluster has previously been reported to be hypermethylated in a large cohort of melanomas [37] as well as in astrocytomas [38], a lethal human brain tumor, implying that it is a rather common phenomenon for many types of cancer. So far, only this study has reported the negative correlation of the MT1 cluster in breast cancer, that is, its gene expression levels are decreased upon its hypermethylation, and thus, we speculate that the hypermethylation of this cluster may be breast cancer specific. We also observed some gene clusters having a positive correlation, that is, their gene expression levels were increased upon their hypermethylation in breast tumors.

Despite our data supporting a notion that estrogen mediates epigenetic repression of the MT1 gene cluster in MCF7 cells (Figure 6), the underlying mechanism of what triggers this long-range coordinated repression process remains obscured. Our previous study [21] has shown that persistent estrogen-mediated long-range repression leads to recruitment of H3K27me3 repressive chromatin marks, which are accompanied by the accumulation of DNA methylation in a gene cluster located at 16p11.2. Recent studies have also shown that estrogen and ERα positively regulate the expression of various methyltransferases (DNMTs), thereby contributing to the malignant transformation of cells in various estrogen responsive breast and endometrial cancers [39,40]. We thus speculate that estrogen mediates the recruiting of some chromatin modifying enzymes, such as polycomb complex, then estrogen further recruits DNA methylation machinery, thus triggering a DNA methylation process at a single embedded gene, which then spreads to other neighboring genes due to their closeness and eventually methylation of a whole cluster occurs.

One important finding in our studies is that we demonstrated the invasiveness of MT1F and MT1M in MCF7 cells (Figure 7). Despite other studies finding that loss of MT1 or one of MT1 member genes was significantly correlated with invasiveness in other tumor types [41], our study depicts the anti-oncogenic role in breast cancer cell line. Our results offer mechanistic insights into breast tumorigenesis, suggesting that methylation of MT1 gene cluster is involved in oncogenic events. We have used MCF7 as a model cell line for tumors showing ERα + phenotype, and our findings provide a compelling evidence for a comprehensive mechanistic study of estrogen-mediated epigenetic repression of MT1 cluster in other breast cancer cell lines representing various breast cancer subtypes.

## Conclusions

Our studies used a sequence-based methylation protocol (MBD-seq) to identify thousands of breast tumor hypermethylated regions, in particular, discovering seven large contiguous hypermethylated gene clusters from a breast cancer patient cohort. Importantly, we were able to use the methylation levels of the cluster to stratify the patients for overall survival, pointing to the potential prognostic and therapeutic significance for this epigenetic modification. As these gene clusters were also selected based on their association with cancer development, their prognostic potential in determining overall survival provides a compelling case for future mechanistic studies.

## Methods

### DNA samples

DNA samples of 77 breast tumors (*n* = 77) and 10 normal breast tissues from healthy individuals (*n* = 10) (Additional file 1: Table S1) and 38 ICBP breast cancer cell lines (*n* = 38) were isolated for subsequent DNA methylation analysis. The breast tumor samples were collected from patients at Chile, IRB # 11-11-3239 approved by UTHSCSA. These tissues were in various stages of tumor advancement and from patients representing different ER and PR statuses. The normal samples were obtained from normal individuals undergoing reduction mammoplasty (Additional file 3). All tissues were obtained following approval of the Institutional Review Board committee.

### Cell culture

Most of ICBP panel breast cell lines were obtained from NCI Cancer Biology Program at NCI in November 2008, except HMEC, MCF7, MDAMB134, BT474, BT20, and MDA-MB231 were obtained from American Type Culture Collection (ATCC) (ATCC Breast Cancer Cell Panel, Manassas, VA, USA). All cell lines have been tested and authenticated by ATCC and maintained in our laboratory for less than 6 months during which all experiments were conducted. All cell lines were cultured in ATCC recommended media and conditions. Cell lines MCF7, MDAMB134, BT474, MDA-MB231, and BT20 were cultured in DMEM culture medium (Life Technologies, Grand Island, NY, USA) supplemented with 10% fetal bovine serum (FBS). For all the treatments with E2 and DAC, RPMI culture medium without phenol red was used and supplemented with 10% charcoal stripped-heat inactivated (CSHI) FBS.

### MBDCap sequencing analysis

Methylated DNA was eluted by the MethylMiner Methylated DNA Enrichment Kit (Invitrogen, Carlsbad, CA, USA) for 77 breast tumors and 10 normal samples according to the manufacturer’s instructions. Briefly, 1 μg of genomic DNA was sonicated and captured by MBD proteins. The methylated DNA was eluted in 1-M salt buffer. DNA in each eluted fraction was precipitated by glycogen, sodium acetate, and ethanol and was resuspended in TE buffer. Eluted DNA was used to generate libraries for sequencing following the standard protocols from Illumina. MBDCap-seq libraries were sequenced using the Illumina Genome Analyzer II (Illumina, Inc., San Diego, CA, USA) as per manufacturer’s instructions. Image analysis and base calling were performed with the standard Illumina pipeline. Sequencing reads were mapped by ELAND algorithm. Unique reads were up to 36 base pair reads mapped to the human reference genome (hg18), with up to two mismatches. Reads in satellite regions were excluded due to the large number of amplifications (Additional file 1: Table S2). The methylation level was normalized based on the unique read numbers for each sample by our newly developed linear method. The 100-bp bin size was used for methylation level calculation. Within each bin, the methylation level was quantified by accumulating the read numbers in which whole or part of the read was located. The following equation was used to normalize the methylation level in each bin:

$$ {N}_{\mathrm{Read},t}=\frac{U_{\mathrm{Read},t}}{N_U/{10}^{\wedge}\left(\mathrm{I}\mathrm{N}\mathrm{T}\left({ \log}_{10}{N}_U\right)\right)} $$

where *N*_Read*,t*_ is the normalized read number of the *i*th bin and *U*_Read*,t*_ is the uniquely mapped read number of the *i*th bin, is the total uniquely mapped reads number. ‘INT’ function rounds the element (in the parenthesis) to the nearest integers towards minus infinity, and ‘^’ means the power operator.

### Identification of differentially methylated regions

DMRs were identified by comparing the difference of averaged methylation values in a defined region between tumors vs. normal tissues (Additional file 1: Table S3). The CGIs in the human genome are based on annotations of UCSC (University of California, Santa Cruz Genome Browser). The regions could be any length, but 8-kb was used in this study because the majority of CGIs are within 2-kb up- or down-stream of the transcription start site (5′TSS), and CGI shores are up to 2-kb distance relative to its CGIs [8]. DMRs were identified by Student’s *t*-test to calculate the difference. *A*_*R,G*_ refers to the average methylation level of group *G* at region *R. R is* a given region including *m* bin size and starting at the *s*th bin. *M*_*R,G*_ is the methylation levels of each sample of group *G* at region *R. SA* is sample number for group A. The DMRs (normal controls vs. breast tumors) were defined by *P* < 0.01.

$$ {A}_{R,G}=\frac{\sum_G{M}_RG}{S_G},R=\left({b}_{s+0},{b}_{s+1},\dots, {b}_{s+m}\right) $$

To further characterize methylation patterns within each DMR, methylation values from 8-kb window were divided into three segments equally representing CGI regions and flanking shore regions. For each segment, it was considered an individual DMR if the *P* value is smaller than the thresholds. Within 8-kb windows, the center is the TSS for each transcript or is in the middle of its CGI for each intragenic or intergenic region.

### Survival analysis

The lifetime of OS was defined as the time between the first operation or first-line chemotherapy and end of follow-up or death due to breast cancer. The third quartile (equal 75th percentile) of methylation levels was used to identify high methylation (HM) and low methylation (LM) of each promoter region. HMs and LMs were converted to 1 and 0 binary codes to do Kaplan-Meier survival and Cox proportional hazard model analysis. We select genes as clusters, who are part of HUGO gene families and which consist of genes located on the same chromosome and consecutive geographic region. The selection criteria for combination of family genes were that they were grouped by hazard ratio higher or lower than one with log-rank test *P* < 0.3 calculated for individual genes in the family (here, we assume that including borderline significant genes will collaboratively contribute towards significant association with disease outcome). In a combination of the same family genes, the receiver operating characteristic (ROC) curve was used to select an optimal cutoff value for the numerous HM of promoter regions. Bootstrapping was performed 200 times, and ‘best’ methods were used to classify dead and alive patients by pROC package of R (version 2.13).

### E2 and DAC treatment

Cell lines were incubated in charcoal stripped media for 24 h and then treated with DAC (1 μM) for 72 h. The cells were further treated with E2 (70 nM) for 36 h with and without DAC treatment. RNA was isolated from harvested cells, which were then harvested and subjected to real-time RT-qPCR to examine expression of the MT1 genes. PCR primers are outlined in Additional file 1: Table S4, and conditions are described previously [42]. Relative expression was determined by the formula 2^−*ΔΔCt*^.

### ERE luciferase assay

MCF7 cells were transfected in triplicate with ERE and Renilla vectors, kindly supplied by Dr. Rong Li, Department of Molecular Medicine, University of Texas Health Science Center (50:1 to make total DNA of 110 ng/well) for 24 h. Cells were incubated with charcoal strip media for 24 h and then treated with E2 for 24 h. The ERE and Renilla activity was calculated with Dual-Glo Luciferase Assay Kit (Promega, Madison, WI, USA).

### Cell invasion assay and proliferation assays

Specific siRNA was used to knockdown MT1F and MT1M expression (Thermo Scientific). MCF7 (approximately 50,000 cells) was transfected with MT1F and MT1M siRNA seeded onto the top insert (layered with Matrigel) of an invasion chamber (BD Biosciences, Franklin Lakes, NJ, USA). The invasion chambers were then incubated at 37°C in 5% CO_2_ for 20 h. Cells that did not invade through the Matrigel (on the upper surface of the insert membrane) were mechanically removed with cotton tip applications and several washes with PBS. Invaded cells on the bottom of the coated membranes were visualized using a fluorescence microscope with a × 20 objective after incubation with Hoechst stain (Life Technologies). Images were obtained from four standardized non-overlapping fields. Invaded cells were counted using the Image J software (http://rsbweb.nih.gov/ij/). Invasion assays were done in triplicate; images of four fields per well (covering about 85% of the well) were taken for counting invaded cells. Cellular proliferation was assayed using CellTiter-Glo® Luminescence Kit (Promega, Madison, WI, USA) according to the manufacturer’s directions as described earlier [43].

### Bioinformatics and statistical analysis

We considered statistical significance as *P* < 0.05 for all analyses unless explicitly stated. Student’s *t*-test was used to compare RT-qPCR, and invasion assay results in different treatment and control groups. Statistical significance was assigned as * if *P* < 0.05, ** if *P* < 0.01, and *** if *P* < 0.001.

GC content and phastCons score: For each gene, we retrieved a genomic region with a length of 8-kb DNA sequence spanning from 4-kb upstream to 4-kb downstream around 5′TSS. The regions were then divided into 160 bins with a bin size of 50 bp. The GC content is calculated for each bin as the number of G + C in that particular bin divided by the bin size of 50. The phastCons score is calculated for each bin using the values from UCSC genome browser (http://hgdownload.soe.ucsc.edu/goldenPath/hg18/phastCons44way/). The phastCons score represents conservation information among 45 species. For each gene cluster, we simply calculated the mean and standard deviation values from all member genes in the cluster for all 160 bins. We also used the whole RefSeq gene set as a background control.

*In silico* gene expression correlation: The clinical information and gene expression values for the breast tumors and normal tissues were downloaded from the TCGA website (http://cancergenome.nih.gov/). A total of 106 normal tissue samples and 988 primary tumor samples with mRNA expression information are currently available for the correlation analysis. Of 988 tumor samples, 207 tumor samples are ERα−, 704 tumor samples are ERα+, and 77 tumor samples are classified as unknown due to missing clinical information. We then used the gene expression values for our seven gene clusters and created an expression matrix to examine their expression pattern. We applied a *z*-score normalization method on each individual gene on their log-transformed RPKM values across the sample set. In addition, we have calculated the log2 fold change values for normal group vs. tumor group and ERα − group vs. ERα−, respectively. A visualization map for the matrix was constructed by Python library package.

*In silico* correlation between expression and DNA methylation: RNA-seq and DNA methylation values were retrieved from TCGA using CGDSR library in R. The patient samples showing values for both RNA-seq and DNA methylation for each patient were retained. Scatterplots showing correlation between RNA-seq and DNA methylation were created after excluding the outliers falling in top and bottom 5 quartile of the data.

## Additional files

Additional file 1:
**Genome-wide DNA methylation analysis reveals estrogen-mediated epigenetic repression of Metallothionein-1 gene cluster in breast cancer.** Supporting figures and tables mentioned in the manuscript have been included. Additional file 2:
**Gene family information.** All the gene families and the genes included in the clusters along with their statistical significance scores (as described in the Methods section) have been included. Additional file 3:
**Histologic data.** Clinical information for all the patient samples investigated in this study have been included.
