# Supplementary material for: Genome-wide DNA methylation analysis reveals estrogen-mediated epigenetic repression of metallothionein-1 gene cluster in breast cancer
Source: Clin Epigenetics. 2015 Feb 24;7(1):13. doi: 10.1186/s13148-015-0045-9 (PMC4355986; doi:10.1186/s13148-015-0045-9)
Supplement: Additional file 1: — Genome-wide DNA methylation analysis reveals estrogen-mediated epigenetic repression of Metallothionein-1 gene cluster in breast cancer. Supporting figures and tables mentioned in the manuscript have been included. [file 13148_2015_45_MOESM1_ESM.docx]

**Additional file**

**Genome-wide DNA methylation analysis reveals estrogen-mediated epigenetic repression of Metallothionein-1 gene cluster in breast cancer**

Rohit R. Jadhav, Zhenqing Ye, Rui-Lan Huang, Joseph Liu , Pei-Yin Hsu, Yi-Wen Huang, Leticia B. Rangel, Hung-Cheng Lai, Juan Carlos Roa, Nameer B. Kirma, Tim Hui-Ming Huang, Victor X. Jin

**Table of Contents**

**Figure S1.** Summarized sequencing reads in breast samples**3**

**Figure S2.** Example loci showing hypermethylation in genomic regions**4**

**Figure S3.** Map showing locations of *HIST1* loci on the genome**5**

**Figure S4.** Map showing locations of *PCDH* loci on the genome**6**

**Figure S5.** Map showing locations of *HOXA* loci on the genome**7**

**Figure S6.** Map showing locations of *HOXD* loci on the genome**8**

**Figure S7.** Map showing locations of *HOXC* loci on the genome**9**

**Figure S8.** Map showing locations of *ZNF* loci on the genome**10**

**Figure S9.** Characterization of identified gene clusters with respect to their GC content and phylogenetic conservation.**11**

**Figure S10.** Scatterplots showing correlation between gene expressions and DNA methylation for MT1 genes in TCGA patient samples.**12**

**Figure S11.** Boxplots showing significant difference in average gene methylation in each subset compared to normal samples for each cluster**14**

**Table S1.** Characteristics of Breast cancer patients **15**

**Table S2.** Summary of sequencing reads in breast tumors & normal controls**16**

**Table S3.** Differentially methylated promoter CpG islands in tumors **19**

**Table S4.** List of primer sequences**115**

**Table S5.** List of siRNA sequences **115**


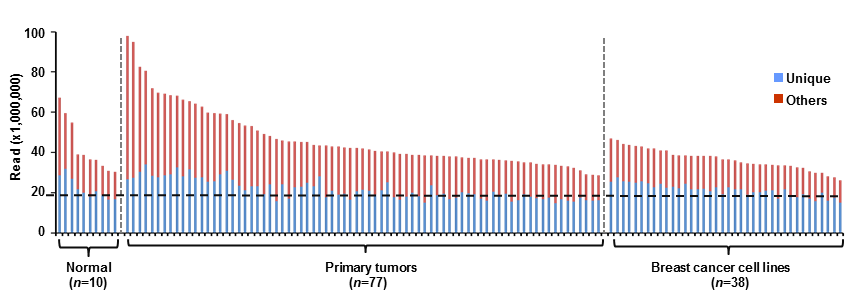


**Figure S1.** Summarized sequencing reads in normal samples (n=10) primary breast tumors (n=77), and breast cancer cell lines (n=38). Each bar represents an individual sample. Blue and red bars indicate unique reads and repeat sequences, respectively.

**
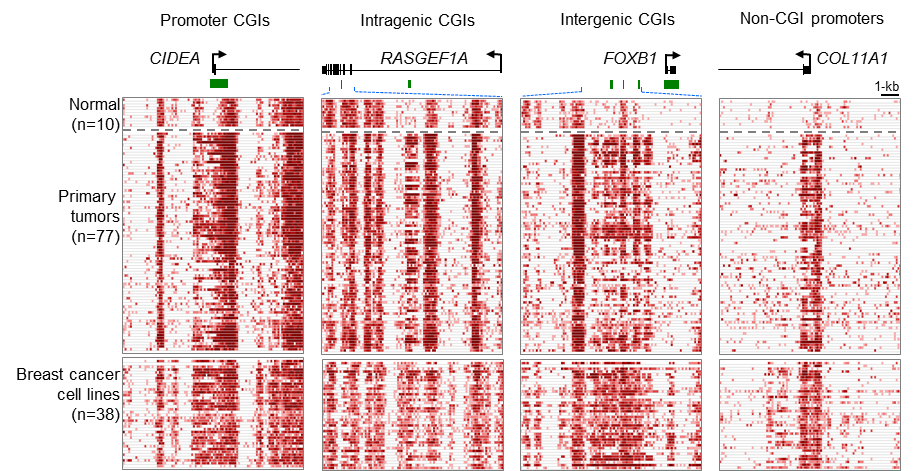
**

**Figure S2.** Example loci showing promoter CGI, intragenic, intergenic and non-CGI promoter regions in a breast cancer cohort as well as a panel of 38 breast cancer cell lines. Dashed squares highlight regions corresponding to breast cancer hypermethylation.

**
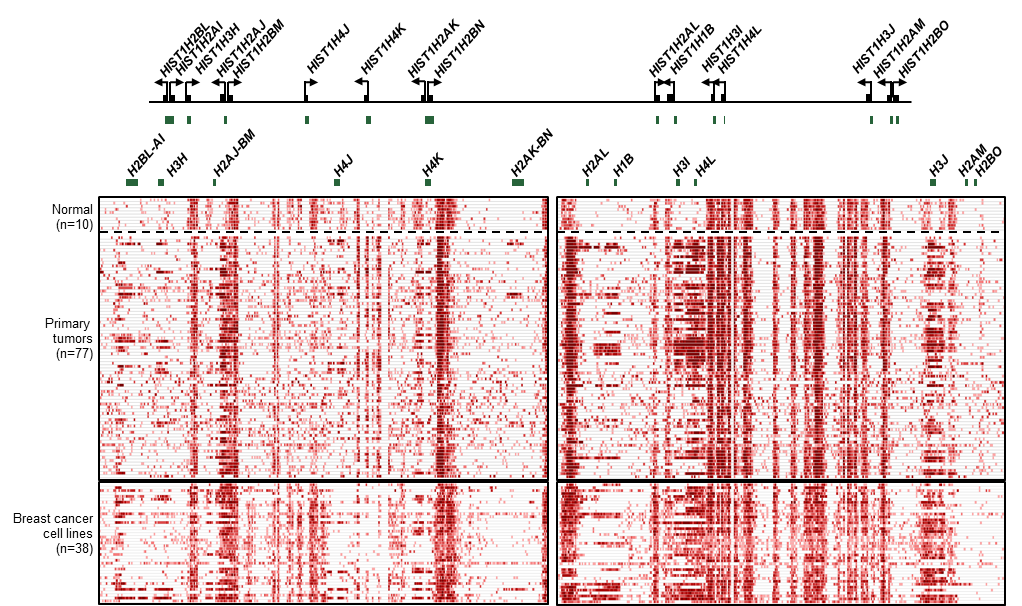
**

**Figure S3.** Map showing genomic organization of the *HIST1* gene cluster and locations of the corresponding loci and CpG islands (green rectangles below map).

**
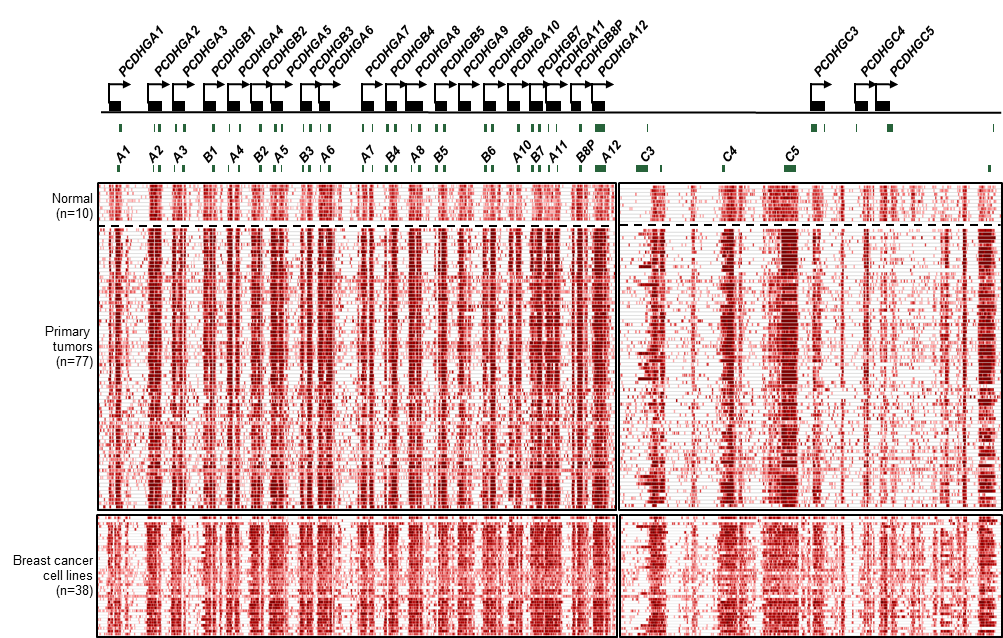
**

**Figure S4.** Map showing genomic organization of the *PCDH* gene cluster and locations of the corresponding loci and CpG islands (green rectangles below map).

**
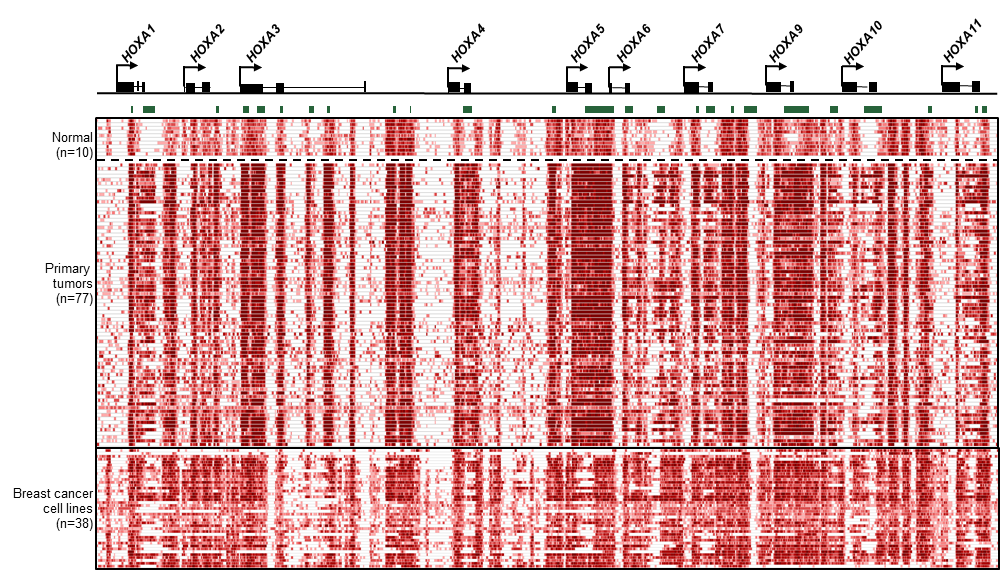
**

**Figure S5.** Map showing genomic organization of the *HOXA* gene cluster and locations of the corresponding loci and CpG islands (green rectangles below map).

**
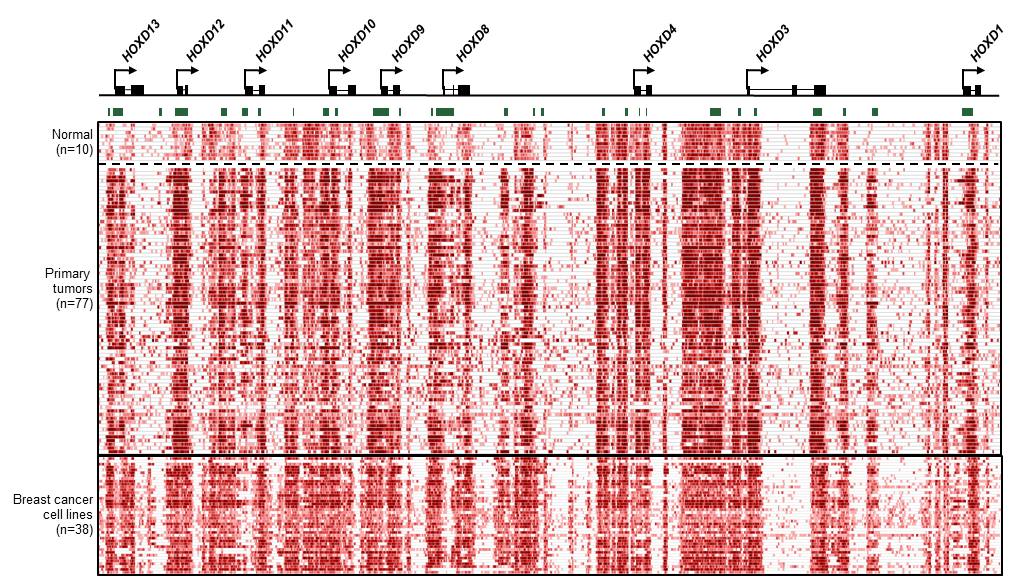
**

**Figure S6.** Map showing genomic organization of the *HOXD* gene cluster and locations of the corresponding loci and CpG islands (green rectangles below map).

**
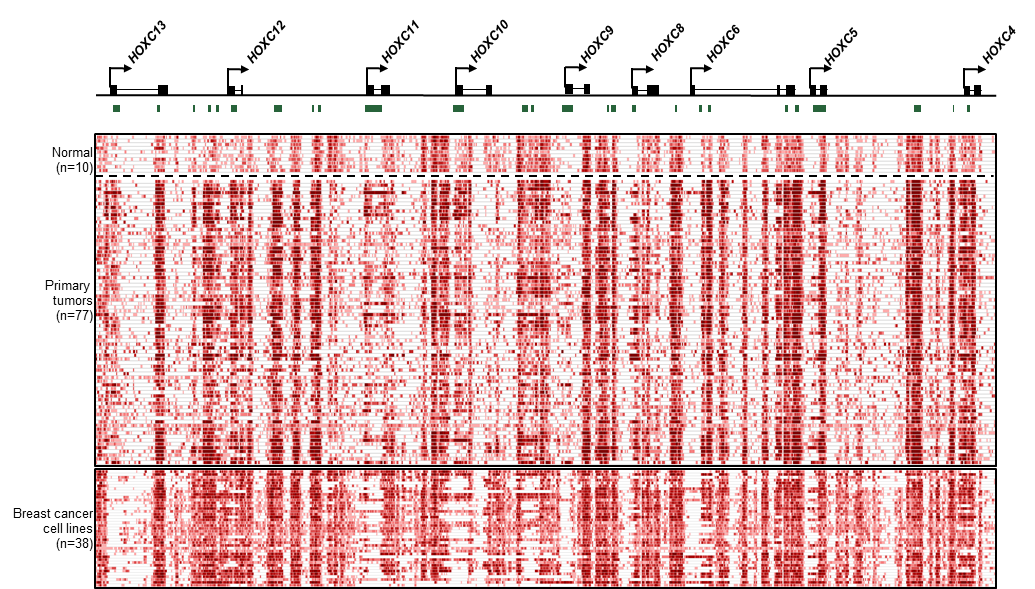
**

**Figure S7.** Map showing genomic organization of the *HOXC* gene cluster and locations of the corresponding loci and CpG islands (green rectangles below map).

**
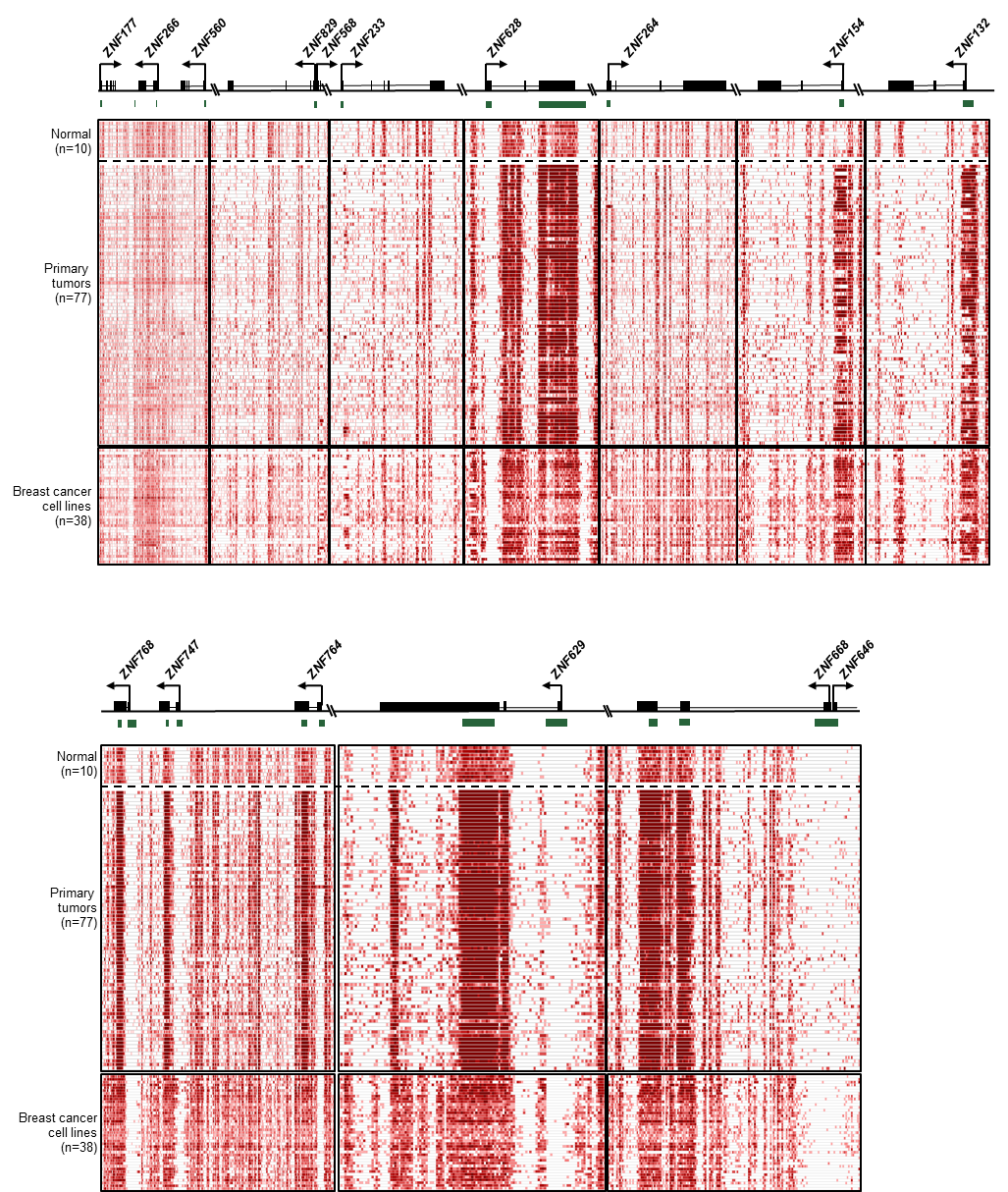
**

**Figure S8.** Map showing genomic organization of the *ZNF* gene clusters and locations of the corresponding loci and CpG islands (green rectangles below map).

**
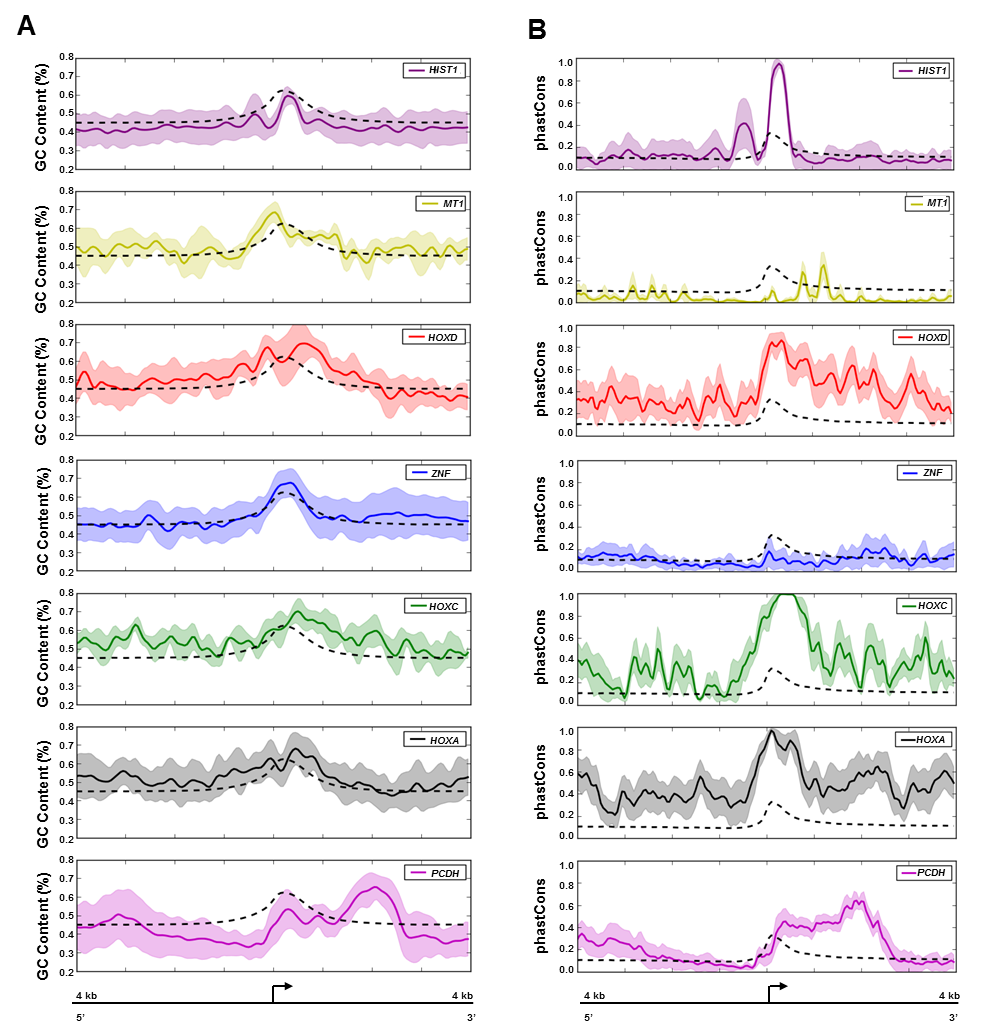
** **Figure S9.** Characterization of identified gene clusters with respect to their GC content and phylogenetic conservation. **A**, Average GC content around the transcription start site (TSS) for genes representing each cluster (solid line) compared to the average GC content level for all the genes in the human genome (dotted line). **B**, Average phylogenetic conservation score around the TSS for genes representing each cluster (solid line) compared to the average phylogenetic conservation score for all the genes in the human genome across species.


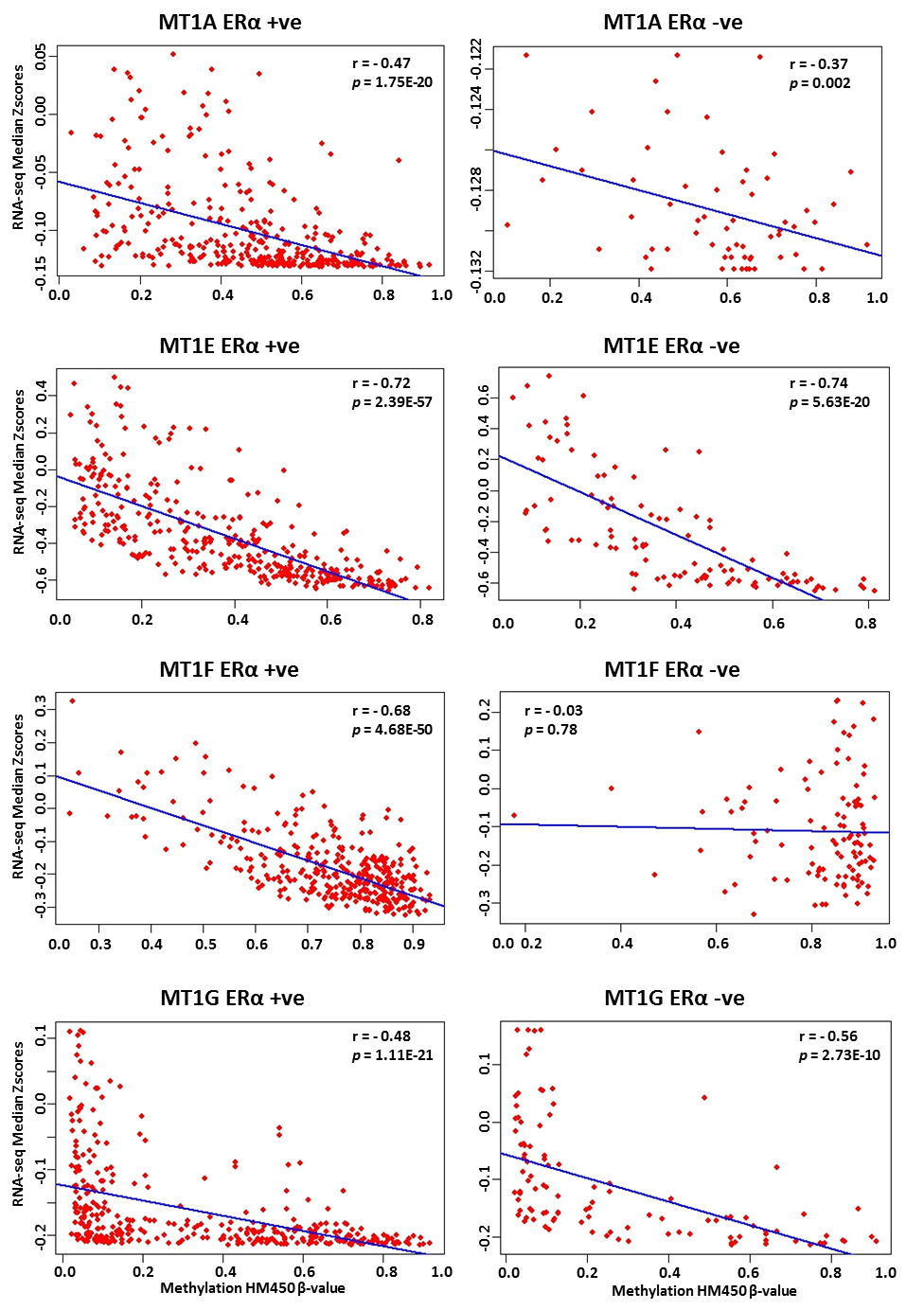

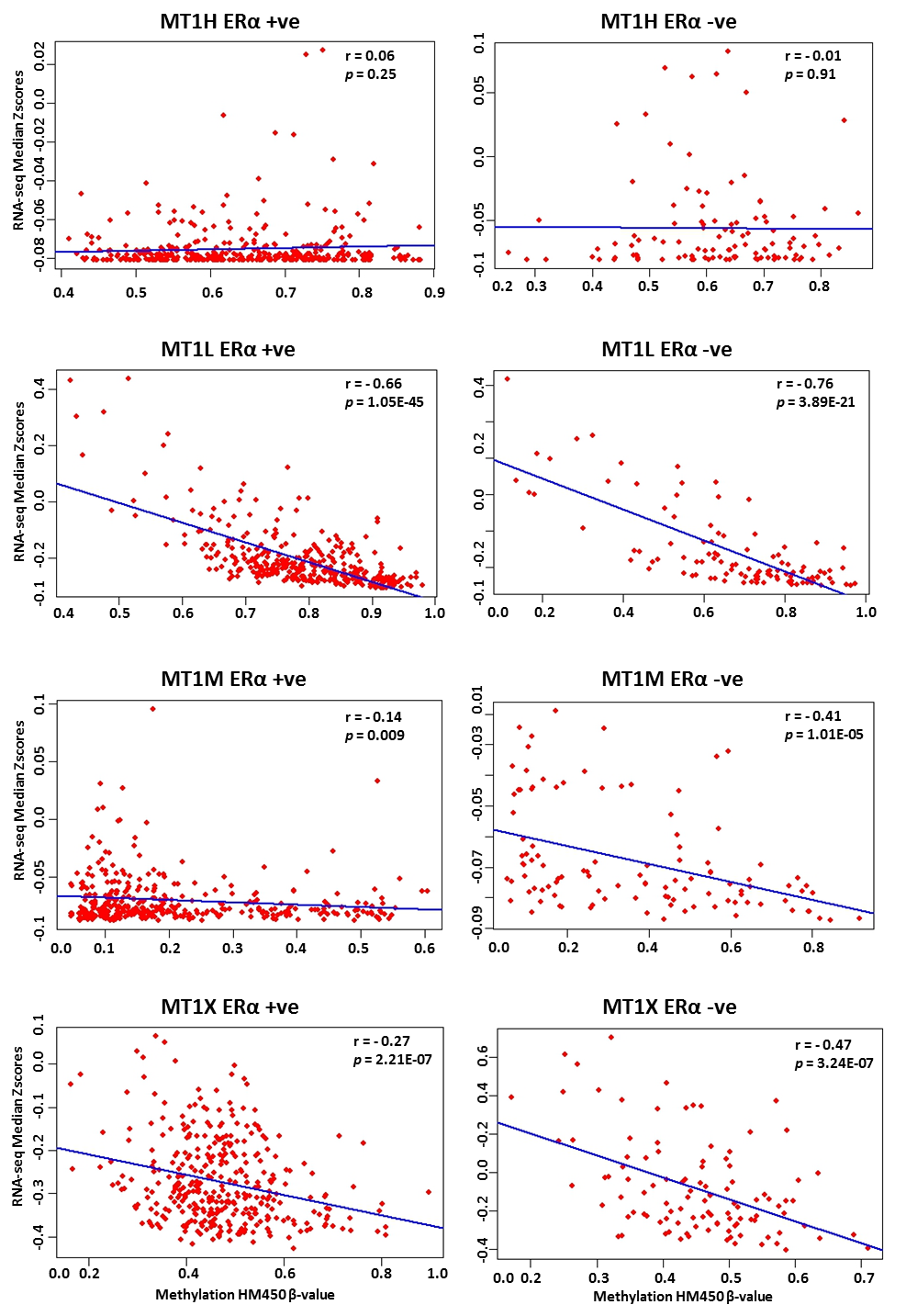


**Figure S10.** Scatterplots showing correlation between gene expressions and DNA methylation for MT1 genes in TCGA patient samples.


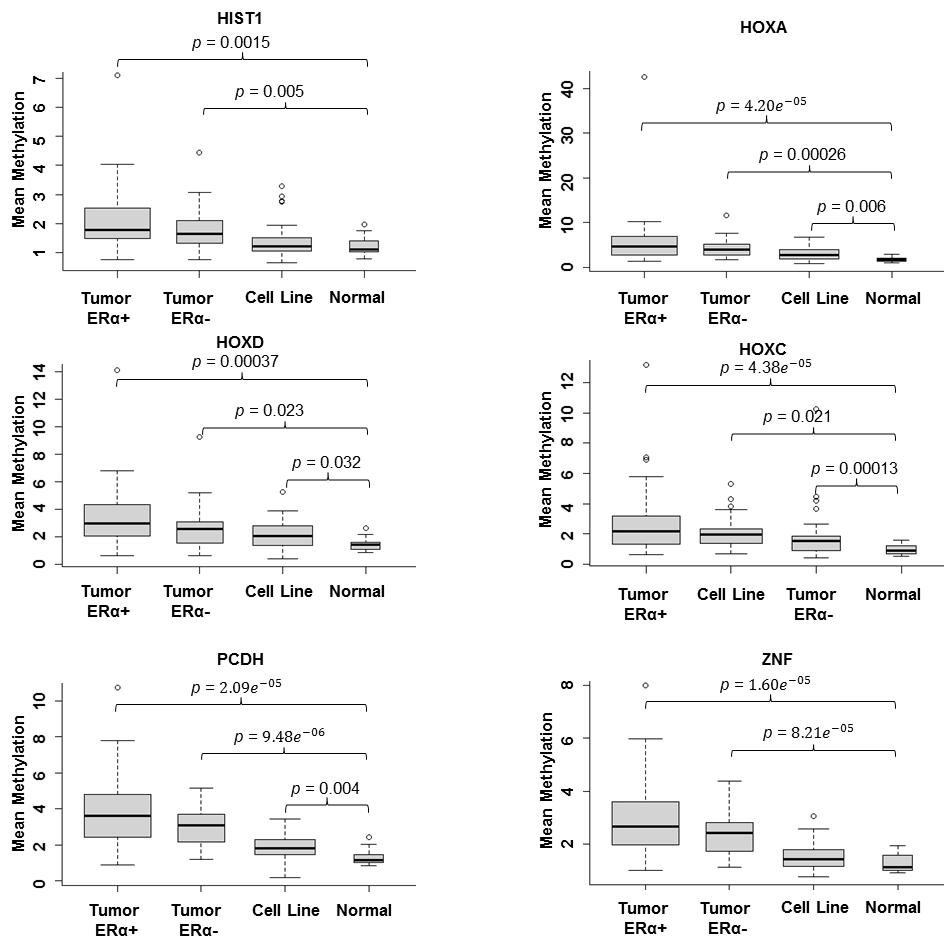


**Figure S11.** Boxplots showing significant difference in average gene methylation in each subset compared to normal samples for each cluster.

| Characteristic | Tumor |
| --- | --- |
| Age |  |
| <50 | 26 |
| 50~59.9 | 15 |
| 60~69.9 | 16 |
| 70~79.9 | 16 |
| 80 and older | 4 |
| Tumor stage |  |
| 1 | 6 |
| 2 | 45 |
| 3 | 23 |
| 4 | 3 |
| Unknown | 0 |
| Elston Tumor grade |  |
| 1 | 12 |
| 2 | 34 |
| 3 | 28 |
| Unknown | 3 |
| ER, PR status |  |
| ER Positive | 50 |
| ER Negative | 25 |
| PR Positive | 34 |
| PR Negative | 41 |
| Unknown | 2 |
| Events |  |
| Alive | 55 |
| Death | 22 |
| Unknown | 0 |
| Total | 77 |

**Table 1** Characteristics of Breast cancer patients

| **Table 2** Summary of sequencing reads in breast tumors and normal controls | | | | | | | |
| --- | --- | --- | --- | --- | --- | --- | --- |
| Subtype | ID | Number of reads | Number of reads | Alignment rate | Number of reads | Number of reads | Unique read rate in total reads |
|  |  | (total) | (aligned) |  | (unique) | (multiple) |  |
| Normal | 1 | 39,142,889 | 34,826,718 | 88.97% | 21,777,398 | 13,049,320 | 55.64% |
| Normal | 2 | 67,288,980 | 52,022,888 | 77.31% | 28,558,779 | 23,464,109 | 42.44% |
| Normal | 3 | 30,280,529 | 27,272,240 | 90.07% | 16,638,996 | 10,633,244 | 54.95% |
| Normal | 4 | 36,425,148 | 32,197,848 | 88.39% | 18,072,494 | 14,125,354 | 49.62% |
| Normal | 5 | 36,393,756 | 32,297,454 | 88.74% | 20,668,007 | 11,629,447 | 56.79% |
| Normal | 6 | 38,714,017 | 33,406,257 | 86.29% | 20,001,858 | 13,404,399 | 51.67% |
| Normal | 7 | 54,815,229 | 43,547,524 | 79.44% | 26,950,087 | 16,597,437 | 49.17% |
| Normal | 8 | 33,373,145 | 30,102,400 | 90.20% | 18,684,801 | 11,417,599 | 55.99% |
| Normal | 9 | 59,528,623 | 53,134,296 | 89.26% | 31,751,559 | 21,382,737 | 53.34% |
| Normal | 10 | 30,972,586 | 27,891,113 | 90.05% | 16,390,364 | 11,500,749 | 52.92% |
| Tumor | 11 | 42,888,942 | 35,226,674 | 82.13% | 18,994,494 | 16,232,180 | 44.29% |
| Tumor | 12 | 80,603,867 | 66,844,195 | 82.93% | 34,170,976 | 32,673,219 | 42.39% |
| Tumor | 13 | 42,217,030 | 31,317,635 | 74.18% | 16,512,475 | 14,805,160 | 39.11% |
| Tumor | 14 | 40,117,502 | 32,831,092 | 81.84% | 17,640,392 | 15,190,700 | 43.97% |
| Tumor | 15 | 43,547,041 | 36,885,604 | 84.70% | 17,847,969 | 19,037,635 | 40.99% |
| Tumor | 16 | 53,263,451 | 43,234,186 | 81.17% | 21,306,291 | 21,927,895 | 40.00% |
| Tumor | 17 | 39,355,877 | 34,459,233 | 87.56% | 16,401,559 | 18,057,674 | 41.67% |
| Tumor | 18 | 45,493,947 | 37,076,851 | 81.50% | 16,916,050 | 20,160,801 | 37.18% |
| Tumor | 19 | 37,999,804 | 32,593,900 | 85.77% | 17,376,444 | 15,217,456 | 45.73% |
| Tumor | 20 | 49,086,774 | 39,458,907 | 80.39% | 19,140,156 | 20,318,751 | 38.99% |
| Tumor | 21 | 68,482,868 | 55,104,652 | 80.46% | 29,198,290 | 25,906,362 | 42.64% |
| Tumor | 22 | 38,485,973 | 30,109,662 | 78.24% | 15,072,785 | 15,036,877 | 39.16% |
| Tumor | 23 | 94,857,665 | 63,216,820 | 66.64% | 27,383,552 | 35,833,268 | 28.87% |
| Tumor | 24 | 97,791,207 | 57,484,734 | 58.78% | 26,745,834 | 30,738,900 | 27.35% |
| Tumor | 25 | 46,767,955 | 31,662,203 | 67.70% | 15,866,928 | 15,795,275 | 33.93% |
| Tumor | 26 | 71,940,037 | 56,374,779 | 78.36% | 28,501,270 | 27,873,509 | 39.62% |
| Tumor | 27 | 64,237,002 | 52,584,055 | 81.86% | 27,401,823 | 25,182,232 | 42.66% |
| Tumor | 28 | 82,552,137 | 57,879,191 | 70.11% | 30,248,111 | 27,631,080 | 36.64% |
| Tumor | 29 | 59,739,721 | 51,379,656 | 86.01% | 25,438,930 | 25,940,726 | 42.58% |
| Tumor | 30 | 38,008,887 | 31,139,973 | 81.93% | 16,868,031 | 14,271,942 | 44.38% |
| Tumor | 31 | 51,018,451 | 41,081,191 | 80.52% | 23,152,648 | 17,928,543 | 45.38% |
| Tumor | 32 | 32,975,722 | 28,486,188 | 86.39% | 15,942,006 | 12,544,182 | 48.34% |
| Tumor | 33 | 56,099,896 | 49,941,735 | 89.02% | 26,431,206 | 23,510,529 | 47.11% |
| Tumor | 34 | 33,803,220 | 28,201,401 | 83.43% | 14,896,395 | 13,305,006 | 44.07% |
| Tumor | 35 | 32,448,673 | 27,522,217 | 84.82% | 15,405,031 | 12,117,186 | 47.48% |
| Tumor | 36 | 33,256,389 | 28,940,558 | 87.02% | 16,739,926 | 12,200,632 | 50.34% |
| Tumor | 37 | 29,208,487 | 26,459,058 | 90.59% | 16,141,458 | 10,317,600 | 55.26% |
| Tumor | 38 | 59,536,010 | 51,168,662 | 85.95% | 25,775,231 | 25,393,431 | 43.29% |
| Tumor | 39 | 35,596,415 | 31,506,139 | 88.51% | 16,318,951 | 15,187,188 | 45.84% |
| Tumor | 40 | 58,938,709 | 53,338,182 | 90.50% | 30,876,191 | 22,461,991 | 52.39% |
| Tumor | 41 | 38,741,286 | 33,622,406 | 86.79% | 18,325,467 | 15,296,939 | 47.30% |
| Tumor | 42 | 42,369,043 | 36,030,610 | 85.04% | 19,555,159 | 16,475,451 | 46.15% |
| Tumor | 43 | 65,595,362 | 58,344,603 | 88.95% | 31,688,602 | 26,656,001 | 48.31% |
| Tumor | 44 | 34,100,625 | 30,553,429 | 89.60% | 16,867,751 | 13,685,678 | 49.46% |
| Tumor | 45 | 34,002,550 | 29,743,706 | 87.47% | 17,511,169 | 12,232,537 | 51.50% |
| Tumor | 46 | 37,198,652 | 33,474,453 | 89.99% | 19,735,234 | 13,739,219 | 53.05% |
| Tumor | 47 | 41,448,552 | 35,873,647 | 86.55% | 21,067,220 | 14,806,427 | 50.83% |
| Tumor | 48 | 35,025,542 | 30,888,682 | 88.19% | 18,092,705 | 12,795,977 | 51.66% |
| Tumor | 49 | 59,299,231 | 53,349,051 | 89.97% | 29,047,189 | 24,301,862 | 48.98% |
| Tumor | 50 | 69,163,263 | 53,987,502 | 78.06% | 28,597,620 | 25,389,882 | 41.35% |
| Tumor | 51 | 36,443,533 | 32,055,057 | 87.96% | 16,025,646 | 16,029,411 | 43.97% |
| Tumor | 52 | 69,760,607 | 59,957,267 | 85.95% | 27,697,066 | 32,260,201 | 39.70% |
| Tumor | 53 | 62,737,994 | 54,226,704 | 86.43% | 27,629,375 | 26,597,329 | 44.04% |
| Tumor | 54 | 45,336,158 | 38,675,897 | 85.31% | 22,800,971 | 15,874,926 | 50.29% |
| Tumor | 55 | 38,244,261 | 31,217,140 | 81.63% | 18,610,559 | 12,606,581 | 48.66% |
| Tumor | 56 | 36,430,658 | 31,011,744 | 85.13% | 20,584,757 | 10,426,987 | 56.50% |
| Tumor | 57 | 36,037,414 | 27,900,597 | 77.42% | 19,419,214 | 8,481,383 | 53.89% |
| Tumor | 58 | 36,341,116 | 29,195,454 | 80.34% | 18,235,694 | 10,959,760 | 50.18% |
| Tumor | 59 | 40,548,748 | 34,274,613 | 84.53% | 21,233,731 | 13,040,882 | 52.37% |
| Tumor | 60 | 43,646,943 | 36,604,959 | 83.87% | 23,188,270 | 13,416,689 | 53.13% |
| Tumor | 61 | 43,048,290 | 35,166,555 | 81.69% | 20,934,112 | 14,232,443 | 48.63% |
| Tumor | 62 | 28,720,222 | 24,967,813 | 86.93% | 16,198,306 | 8,769,507 | 56.40% |
| Tumor | 63 | 35,103,641 | 30,066,086 | 85.65% | 19,247,240 | 10,818,846 | 54.83% |
| Tumor | 64 | 38,440,439 | 33,850,010 | 88.06% | 23,787,687 | 10,062,323 | 61.88% |
| Tumor | 65 | 45,976,525 | 39,356,672 | 85.60% | 24,104,240 | 15,252,432 | 52.43% |
| Tumor | 66 | 31,201,043 | 27,224,713 | 87.26% | 17,480,411 | 9,744,302 | 56.03% |
| Tumor | 67 | 38,768,911 | 33,393,134 | 86.13% | 19,989,864 | 13,403,270 | 51.56% |
| Tumor | 68 | 36,527,108 | 30,595,349 | 83.76% | 16,972,822 | 13,622,527 | 46.47% |
| Tumor | 69 | 66,273,088 | 52,040,111 | 78.52% | 28,051,231 | 23,988,880 | 42.33% |
| Tumor | 70 | 37,169,655 | 30,302,366 | 81.52% | 19,228,319 | 11,074,047 | 51.73% |
| Tumor | 71 | 34,320,301 | 28,897,983 | 84.20% | 17,228,979 | 11,669,004 | 50.20% |
| Tumor | 72 | 45,154,079 | 38,519,641 | 85.31% | 25,024,048 | 13,495,593 | 55.42% |
| Tumor | 73 | 38,222,876 | 32,831,583 | 85.90% | 19,488,567 | 13,343,016 | 50.99% |
| Tumor | 74 | 40,389,207 | 35,829,049 | 88.71% | 25,162,844 | 10,666,205 | 62.30% |
| Tumor | 75 | 39,321,039 | 31,779,509 | 80.82% | 17,877,403 | 13,902,106 | 45.47% |
| Tumor | 76 | 42,156,771 | 34,986,243 | 82.99% | 20,725,003 | 14,261,240 | 49.16% |
| Tumor | 77 | 43,565,980 | 37,808,745 | 86.79% | 28,046,739 | 9,762,006 | 64.38% |
| Tumor | 78 | 48,172,238 | 39,869,306 | 82.76% | 24,136,845 | 15,732,461 | 50.11% |
| Tumor | 79 | 53,214,685 | 42,758,946 | 80.35% | 23,099,049 | 19,659,897 | 43.41% |
| Tumor | 80 | 68,172,932 | 58,207,163 | 85.38% | 32,587,998 | 25,619,165 | 47.80% |
| Tumor | 81 | 37,466,879 | 33,471,437 | 89.34% | 20,317,473 | 13,153,964 | 54.23% |
| Tumor | 82 | 54,711,031 | 44,329,769 | 81.03% | 23,465,013 | 20,864,756 | 42.89% |
| Tumor | 83 | 40,672,275 | 34,665,666 | 85.23% | 18,916,356 | 15,749,310 | 46.51% |
| Tumor | 84 | 35,821,332 | 29,746,925 | 83.04% | 15,603,274 | 14,143,651 | 43.56% |
| Tumor | 85 | 41,998,905 | 36,500,790 | 86.91% | 21,519,634 | 14,981,156 | 51.24% |
| Tumor | 86 | 45,245,183 | 38,994,542 | 86.18% | 22,914,577 | 16,079,965 | 50.65% |
| Tumor | 87 | 28,993,301 | 25,301,465 | 87.27% | 15,994,551 | 9,306,914 | 55.17% |
|  | Average | 46,305,219 | 38,421,347 | 84% | 21,542,779 | 16,878,568 | 48% |
|  | Total | 4,028,554,035 | 3,342,657,163 |  | 1,874,221,730 | 1,468,435,433 |  |

**Table 3.** Differentially methylated promoter CpG islands in tumors

| **Gene Symbol** | **Gene ID** | **Chromosome** | **Region start** | **Region end** | **P-value** | **Region Type** |
| --- | --- | --- | --- | --- | --- | --- |
| TTLL5 | 254173 | 1 | 1095148 | 1103148 | 0.0016 | Tumor hypermethylation in CpG island core |
| HES5 | 388585 | 1 | 2447544 | 2455544 | 0.0001 | Tumor hypermethylation in CpG island core |
| FLJ42875 | 440556 | 1 | 2970149 | 2978149 | 0.0003 | Tumor hypermethylation in CpG island core |
| WDR8 | 49856 | 1 | 3552531 | 3560531 | 0.0018 | Tumor hypermethylation in CpG island core |
| TP73 | 7161 | 1 | 3593095 | 3601095 | 0.006 | Tumor hypermethylation in CpG island core |
| KIAA0495 | 57212 | 1 | 3649746 | 3657746 | 0.0084 | Tumor hypermethylation in CpG island core |
| AJAP1 | 55966 | 1 | 4610964 | 4618964 | 0 | Tumor hypermethylation in CpG island core |
| HES2 | 54626 | 1 | 6398566 | 6406566 | 0.0002 | Tumor hypermethylation in CpG island core |
| KLHL21 | 9903 | 1 | 6581516 | 6589516 | 0.0013 | Tumor hypermethylation in CpG island core |
| CLSTN1 | 22883 | 1 | 9630376 | 9638376 | 0.0027 | Tumor hypermethylation in CpG island core |
| TNFRSF8 | 943 | 1 | 12042020 | 12050020 | 0.0001 | Tumor hypermethylation in CpG island core |
| TNFRSF1B | 7133 | 1 | 12145646 | 12153646 | 0.0099 | Tumor hypermethylation in CpG island core |
| PDPN | 10630 | 1 | 13778838 | 13786838 | 0 | Tumor hypermethylation in CpG island core |
| PAX7 | 5081 | 1 | 18826086 | 18834086 | 0 | Tumor hypermethylation in CpG island core |
| FAM43B | 163933 | 1 | 20747518 | 20755518 | 0 | Tumor hypermethylation in CpG island core |
| ALPL | 249 | 1 | 21704444 | 21712444 | 0.0043 | Tumor hypermethylation in CpG island core |
| RUNX3 | 864 | 1 | 25125357 | 25133357 | 0.0003 | Tumor hypermethylation in CpG island core |
| SLC30A2 | 7780 | 1 | 26241191 | 26249191 | 0.0064 | Tumor hypermethylation in CpG island core |
| LIN28 | 79727 | 1 | 26605855 | 26613855 | 0 | Tumor hypermethylation in CpG island core |
| TMEM200B | 399474 | 1 | 29319008 | 29327008 | 0.0044 | Tumor hypermethylation in CpG island core |
| ZBTB8B | 728116 | 1 | 32699244 | 32707244 | 0.003 | Tumor hypermethylation in CpG island core |
| CSMD2 | 114784 | 1 | 34400030 | 34408030 | 0 | Tumor hypermethylation in CpG island core |
| TFAP2E | 339488 | 1 | 35807557 | 35815557 | 0.0037 | Tumor hypermethylation in CpG island core |
| GRIK3 | 2899 | 1 | 37268431 | 37276431 | 0 | Tumor hypermethylation in CpG island core |
| EPHA10 | 284656 | 1 | 37999411 | 38007411 | 0.0005 | Tumor hypermethylation in CpG island core |
| POU3F1 | 5453 | 1 | 38281037 | 38289037 | 0.0008 | Tumor hypermethylation in CpG island core |
| NT5C1A | 84618 | 1 | 39906297 | 39914297 | 0.0026 | Tumor hypermethylation in CpG island core |
| HPCAL4 | 51440 | 1 | 39925676 | 39933676 | 0.0088 | Tumor hypermethylation in CpG island core |
| COL9A2 | 1298 | 1 | 40551526 | 40559526 | 0.002 | Tumor hypermethylation in CpG island core |
| HPDL | 84842 | 1 | 45561131 | 45569131 | 0.0036 | Tumor hypermethylation in CpG island core |
| CCDC17 | 149483 | 1 | 45858316 | 45866316 | 0.0044 | Tumor hypermethylation in CpG island core |
| C1orf190 | 541468 | 1 | 46437592 | 46445592 | 0.001 | Tumor hypermethylation in CpG island core |
| TAL1 | 6886 | 1 | 47464030 | 47472030 | 0 | Tumor hypermethylation in CpG island core |
| FOXE3 | 2301 | 1 | 47650330 | 47658330 | 0.002 | Tumor hypermethylation in CpG island core |
| MGC12982 | 84793 | 1 | 47668900 | 47676900 | 0.0003 | Tumor hypermethylation in CpG island core |
| ELAVL4 | 1996 | 1 | 50282272 | 50290272 | 0.0072 | Tumor hypermethylation in CpG island core |
| OSBPL9 | 114883 | 1 | 51964080 | 51972080 | 0 | Tumor hypermethylation in CpG island core |
| GPX7 | 2882 | 1 | 52836630 | 52844630 | 0.0003 | Tumor hypermethylation in CpG island core |
| PODN | 127435 | 1 | 53296472 | 53304472 | 0 | Tumor hypermethylation in CpG island core |
| FLJ40434 | 163742 | 1 | 53674281 | 53682281 | 0.0029 | Tumor hypermethylation in CpG island core |
| DMRTB1 | 63948 | 1 | 53693659 | 53701659 | 0.0003 | Tumor hypermethylation in CpG island core |
| TTC22 | 55001 | 1 | 55035529 | 55043529 | 0.0098 | Tumor hypermethylation in CpG island core |
| PCSK9 | 255738 | 1 | 55273807 | 55281807 | 0 | Tumor hypermethylation in CpG island core |
| OMA1 | 115209 | 1 | 58484799 | 58492799 | 0 | Tumor hypermethylation in CpG island core |
| L1TD1 | 54596 | 1 | 62429061 | 62437061 | 0.0002 | Tumor hypermethylation in CpG island core |
| CACHD1 | 57685 | 1 | 64705063 | 64713063 | 0.0009 | Tumor hypermethylation in CpG island core |
| DNAJC6 | 9829 | 1 | 65499017 | 65507017 | 0.0003 | Tumor hypermethylation in CpG island core |
| PDE4B | 5142 | 1 | 66027443 | 66035443 | 0 | Tumor hypermethylation in CpG island core |
| SGIP1 | 84251 | 1 | 66768412 | 66776412 | 0.0001 | Tumor hypermethylation in CpG island core |
| ST6GALNAC3 | 256435 | 1 | 76308976 | 76316976 | 0.0013 | Tumor hypermethylation in CpG island core |
| ST6GALNAC5 | 81849 | 1 | 77101773 | 77109773 | 0.0002 | Tumor hypermethylation in CpG island core |
| GIPC2 | 54810 | 1 | 78280176 | 78288176 | 0.0006 | Tumor hypermethylation in CpG island core |
| LPHN2 | 23266 | 1 | 82034669 | 82042669 | 0 | Tumor hypermethylation in CpG island core |
| COL24A1 | 255631 | 1 | 86390709 | 86398709 | 0.0003 | Tumor hypermethylation in CpG island core |
| LRRC8C | 84230 | 1 | 89867231 | 89875231 | 0.0016 | Tumor hypermethylation in CpG island core |
| BARHL2 | 343472 | 1 | 90951382 | 90959382 | 0 | Tumor hypermethylation in CpG island core |
| GFI1 | 2672 | 1 | 92717944 | 92725944 | 0 | Tumor hypermethylation in CpG island core |
| MIR137 | 406928 | 1 | 98280315 | 98288315 | 0 | Tumor hypermethylation in CpG island core |
| LPPR5 | 163404 | 1 | 99239037 | 99247037 | 0.0004 | Tumor hypermethylation in CpG island core |
| S1PR1 | 1901 | 1 | 101470892 | 101478892 | 0 | Tumor hypermethylation in CpG island core |
| NTNG2 | 84628 | 1 | 107480267 | 107488267 | 0 | Tumor hypermethylation in CpG island core |
| SLC6A17 | 388662 | 1 | 110490654 | 110498654 | 0.0004 | Tumor hypermethylation in CpG island core |
| KCNC4 | 3749 | 1 | 110551587 | 110559587 | 0.0017 | Tumor hypermethylation in CpG island core |
| KCNA3 | 3738 | 1 | 111015178 | 111023178 | 0.0018 | Tumor hypermethylation in CpG island core |
| SLC16A1 | 6566 | 1 | 113296208 | 113304208 | 0 | Tumor hypermethylation in CpG island core |
| SYT6 | 148281 | 1 | 114493995 | 114501995 | 0 | Tumor hypermethylation in CpG island core |
| NGF | 4803 | 1 | 115678380 | 115686380 | 0.0081 | Tumor hypermethylation in CpG island core |
| HIST2H2BA | 337875 | 1 | 120703556 | 120711556 | 0.002 | Tumor hypermethylation in CpG island core |
| MRPS21 | 54460 | 1 | 148528892 | 148536892 | 0.006 | Tumor hypermethylation in CpG island core |
| ADAMTSL4 | 54507 | 1 | 148784521 | 148792521 | 0.0007 | Tumor hypermethylation in CpG island core |
| SEMA6C | 10500 | 1 | 149381728 | 149389728 | 0.0026 | Tumor hypermethylation in CpG island core |
| C1orf230 | 284485 | 1 | 149956636 | 149964636 | 0.0065 | Tumor hypermethylation in CpG island core |
| LOC100132111 | 1E+08 | 1 | 150073568 | 150081568 | 0.0006 | Tumor hypermethylation in CpG island core |
| CRCT1 | 54544 | 1 | 150749601 | 150757601 | 0.0088 | Tumor hypermethylation in CpG island core |
| NPR1 | 4881 | 1 | 151913787 | 151921787 | 0.0013 | Tumor hypermethylation in CpG island core |
| SHE | 126669 | 1 | 152737213 | 152745213 | 0.0006 | Tumor hypermethylation in CpG island core |
| TRIM46 | 80128 | 1 | 153408983 | 153416983 | 0.0079 | Tumor hypermethylation in CpG island core |
| FAM189B | 10712 | 1 | 153487898 | 153495898 | 0.0079 | Tumor hypermethylation in CpG island core |
| RUSC1 | 23623 | 1 | 153553263 | 153561263 | 0 | Tumor hypermethylation in CpG island core |
| SYT12 | 91683 | 1 | 154091913 | 154099913 | 0.008 | Tumor hypermethylation in CpG island core |
| BCAN | 63827 | 1 | 154874363 | 154882363 | 0.0063 | Tumor hypermethylation in CpG island core |
| NES | 10763 | 1 | 154909813 | 154917813 | 0.0001 | Tumor hypermethylation in CpG island core |
| PEAR1 | 375033 | 1 | 155126146 | 155134146 | 0.0027 | Tumor hypermethylation in CpG island core |
| CADM3 | 57863 | 1 | 157404000 | 157412000 | 0.002 | Tumor hypermethylation in CpG island core |
| KCNJ10 | 3766 | 1 | 158302585 | 158310585 | 0.0006 | Tumor hypermethylation in CpG island core |
| VANGL2 | 57216 | 1 | 158632990 | 158640990 | 0.0007 | Tumor hypermethylation in CpG island core |
| KLHDC9 | 126823 | 1 | 159330777 | 159338777 | 0.0022 | Tumor hypermethylation in CpG island core |
| HSPA6 | 3310 | 1 | 159756659 | 159764659 | 0.0035 | Tumor hypermethylation in CpG island core |
| HSPA7 | 3311 | 1 | 159838472 | 159846472 | 0.0003 | Tumor hypermethylation in CpG island core |
| LMX1A | 4009 | 1 | 163587641 | 163595641 | 0 | Tumor hypermethylation in CpG island core |
| FAM78B | 149297 | 1 | 164398830 | 164406830 | 0.001 | Tumor hypermethylation in CpG island core |
| PRRX1 | 5396 | 1 | 168895936 | 168903936 | 0 | Tumor hypermethylation in CpG island core |
| DNM3 | 26052 | 1 | 170073243 | 170081243 | 0 | Tumor hypermethylation in CpG island core |
| ANKRD45 | 339416 | 1 | 171901624 | 171909624 | 0.0053 | Tumor hypermethylation in CpG island core |
| ASTN1 | 460 | 1 | 175396647 | 175404647 | 0.0006 | Tumor hypermethylation in CpG island core |
| FAM5B | 57795 | 1 | 175403255 | 175411255 | 0 | Tumor hypermethylation in CpG island core |
| NPHS2 | 7827 | 1 | 177807707 | 177815707 | 0.0016 | Tumor hypermethylation in CpG island core |
| FAM163A | 148753 | 1 | 177974920 | 177982920 | 0 | Tumor hypermethylation in CpG island core |
| KIAA1614 | 57710 | 1 | 179144935 | 179152935 | 0.0029 | Tumor hypermethylation in CpG island core |
| CACNA1E | 777 | 1 | 179715338 | 179723338 | 0.0005 | Tumor hypermethylation in CpG island core |
| C1orf14 | 81626 | 1 | 181185176 | 181193176 | 0.0001 | Tumor hypermethylation in CpG island core |
| NMNAT2 | 23057 | 1 | 181650360 | 181658360 | 0.0002 | Tumor hypermethylation in CpG island core |
| PTGS2 | 5743 | 1 | 184912182 | 184920182 | 0.0003 | Tumor hypermethylation in CpG island core |
| RGS2 | 6012 | 1 | 191040791 | 191048791 | 0 | Tumor hypermethylation in CpG island core |
| KCNT2 | 343450 | 1 | 194840122 | 194848122 | 0.0002 | Tumor hypermethylation in CpG island core |
| LHX9 | 56956 | 1 | 196144257 | 196152257 | 0 | Tumor hypermethylation in CpG island core |
| GPR25 | 2848 | 1 | 199104788 | 199112788 | 0.0001 | Tumor hypermethylation in CpG island core |
| KIF21B | 23046 | 1 | 199255451 | 199263451 | 0.0005 | Tumor hypermethylation in CpG island core |
| PKP1 | 5317 | 1 | 199515202 | 199523202 | 0.0068 | Tumor hypermethylation in CpG island core |
| NAV1 | 89796 | 1 | 199880072 | 199888072 | 0.0069 | Tumor hypermethylation in CpG island core |
| SYT2 | 127833 | 1 | 200875204 | 200883204 | 0.0052 | Tumor hypermethylation in CpG island core |
| NFASC | 23114 | 1 | 203060404 | 203068404 | 0 | Tumor hypermethylation in CpG island core |
| RASSF3 | 283349 | 1 | 204743501 | 204751501 | 0.0055 | Tumor hypermethylation in CpG island core |
| YOD1 | 55432 | 1 | 205287045 | 205295045 | 0.0011 | Tumor hypermethylation in CpG island core |
| CR1 | 1378 | 1 | 205732095 | 205740095 | 0.0071 | Tumor hypermethylation in CpG island core |
| CR1L | 1379 | 1 | 205881080 | 205889080 | 0.0028 | Tumor hypermethylation in CpG island core |
| G0S2 | 50486 | 1 | 207911292 | 207919292 | 0.0001 | Tumor hypermethylation in CpG island core |
| SYT14 | 255928 | 1 | 208174160 | 208182160 | 0 | Tumor hypermethylation in CpG island core |
| VASH2 | 79805 | 1 | 211186509 | 211194509 | 0.0006 | Tumor hypermethylation in CpG island core |
| PROX1 | 5629 | 1 | 212224482 | 212232482 | 0 | Tumor hypermethylation in CpG island core |
| PTPN14 | 5784 | 1 | 212787265 | 212795265 | 0.0032 | Tumor hypermethylation in CpG island core |
| KCNK2 | 3776 | 1 | 213319182 | 213327182 | 0.01 | Tumor hypermethylation in CpG island core |
| ESRRG | 2104 | 1 | 215373720 | 215381720 | 0 | Tumor hypermethylation in CpG island core |
| TGFB2 | 7042 | 1 | 216582013 | 216590013 | 0.001 | Tumor hypermethylation in CpG island core |
| SLC30A10 | 55532 | 1 | 218164616 | 218172616 | 0.0013 | Tumor hypermethylation in CpG island core |
| MARK1 | 4139 | 1 | 218764190 | 218772190 | 0.0096 | Tumor hypermethylation in CpG island core |
| HLX | 3142 | 1 | 219115365 | 219123365 | 0 | Tumor hypermethylation in CpG island core |
| CNIH3 | 149111 | 1 | 222866801 | 222874801 | 0 | Tumor hypermethylation in CpG island core |
| MIXL1 | 83881 | 1 | 224474005 | 224482005 | 0.0031 | Tumor hypermethylation in CpG island core |
| WNT3A | 89780 | 1 | 226257374 | 226265374 | 0 | Tumor hypermethylation in CpG island core |
| TRIM17 | 51127 | 1 | 226667206 | 226675206 | 0.0075 | Tumor hypermethylation in CpG island core |
| HIST3H2A | 92815 | 1 | 226708183 | 226716183 | 0 | Tumor hypermethylation in CpG island core |
| FAM89A | 375061 | 1 | 229238618 | 229246618 | 0 | Tumor hypermethylation in CpG island core |
| TRIM67 | 440730 | 1 | 229361296 | 229369296 | 0 | Tumor hypermethylation in CpG island core |
| KCNK1 | 3775 | 1 | 231812372 | 231820372 | 0.0074 | Tumor hypermethylation in CpG island core |
| SLC35F3 | 148641 | 1 | 232103301 | 232111301 | 0.0022 | Tumor hypermethylation in CpG island core |
| GNG4 | 2786 | 1 | 233876677 | 233884677 | 0.0008 | Tumor hypermethylation in CpG island core |
| NID1 | 4811 | 1 | 234291104 | 234299104 | 0 | Tumor hypermethylation in CpG island core |
| ACTN2 | 88 | 1 | 234912392 | 234920392 | 0.0008 | Tumor hypermethylation in CpG island core |
| RYR2 | 6262 | 1 | 235268324 | 235276324 | 0.0001 | Tumor hypermethylation in CpG island core |
| FMN2 | 56776 | 1 | 238317807 | 238325807 | 0 | Tumor hypermethylation in CpG island core |
| RGS7 | 6000 | 1 | 239583101 | 239591101 | 0 | Tumor hypermethylation in CpG island core |
| PLD5 | 200150 | 1 | 240750621 | 240758621 | 0 | Tumor hypermethylation in CpG island core |
| LOC149134 | 149134 | 1 | 245015541 | 245023541 | 0.0033 | Tumor hypermethylation in CpG island core |
| ADARB2 | 105 | 10 | 1765718 | 1773718 | 0.0004 | Tumor hypermethylation in CpG island core |
| PFKP | 5214 | 10 | 3095751 | 3103751 | 0.0002 | Tumor hypermethylation in CpG island core |
| CALML3 | 810 | 10 | 5552923 | 5560923 | 0.0009 | Tumor hypermethylation in CpG island core |
| SFMBT2 | 57713 | 10 | 7489456 | 7497456 | 0.0002 | Tumor hypermethylation in CpG island core |
| CUGBP2 | 10659 | 10 | 11095898 | 11103898 | 0 | Tumor hypermethylation in CpG island core |
| CCDC3 | 83643 | 10 | 13079710 | 13087710 | 0.0066 | Tumor hypermethylation in CpG island core |
| FAM171A1 | 221061 | 10 | 15449064 | 15457064 | 0.0029 | Tumor hypermethylation in CpG island core |
| VIM | 7431 | 10 | 17306263 | 17314263 | 0 | Tumor hypermethylation in CpG island core |
| SPAG6 | 9576 | 10 | 22670404 | 22678404 | 0.0002 | Tumor hypermethylation in CpG island core |
| PTF1A | 256297 | 10 | 23517465 | 23525465 | 0 | Tumor hypermethylation in CpG island core |
| KIAA1217 | 56243 | 10 | 24019680 | 24027680 | 0 | Tumor hypermethylation in CpG island core |
| LOC100128811 | 1E+08 | 10 | 25501211 | 25509211 | 0 | Tumor hypermethylation in CpG island core |
| MYO3A | 53904 | 10 | 26259007 | 26267007 | 0.0019 | Tumor hypermethylation in CpG island core |
| GAD2 | 2572 | 10 | 26541241 | 26549241 | 0 | Tumor hypermethylation in CpG island core |
| APBB1IP | 54518 | 10 | 26763271 | 26771271 | 0.0028 | Tumor hypermethylation in CpG island core |
| PTCHD3 | 374308 | 10 | 27739303 | 27747303 | 0.0012 | Tumor hypermethylation in CpG island core |
| FZD8 | 8325 | 10 | 35966368 | 35974368 | 0 | Tumor hypermethylation in CpG island core |
| ANKRD30A | 91074 | 10 | 37450790 | 37458790 | 0.0045 | Tumor hypermethylation in CpG island core |
| CXCL12 | 6387 | 10 | 44196548 | 44204548 | 0.0007 | Tumor hypermethylation in CpG island core |
| SYT15 | 83849 | 10 | 46386607 | 46394607 | 0.0013 | Tumor hypermethylation in CpG island core |
| GDF10 | 2662 | 10 | 48055172 | 48063172 | 0.0009 | Tumor hypermethylation in CpG island core |
| SLC18A3 | 6572 | 10 | 50484352 | 50492352 | 0 | Tumor hypermethylation in CpG island core |
| DKK1 | 22943 | 10 | 53740046 | 53748046 | 0 | Tumor hypermethylation in CpG island core |
| BICC1 | 80114 | 10 | 59938909 | 59946909 | 0.0002 | Tumor hypermethylation in CpG island core |
| PHYHIPL | 84457 | 10 | 60602353 | 60610353 | 0 | Tumor hypermethylation in CpG island core |
| TMEM26 | 219623 | 10 | 62879214 | 62887214 | 0.001 | Tumor hypermethylation in CpG island core |
| C10orf107 | 219621 | 10 | 63088724 | 63096724 | 0.0088 | Tumor hypermethylation in CpG island core |
| EGR2 | 1959 | 10 | 64242132 | 64250132 | 0.0001 | Tumor hypermethylation in CpG island core |
| NEUROG3 | 50674 | 10 | 70999128 | 71007128 | 0.0001 | Tumor hypermethylation in CpG island core |
| NODAL | 4838 | 10 | 71867471 | 71875471 | 0 | Tumor hypermethylation in CpG island core |
| KIAA1274 | 27143 | 10 | 71904569 | 71912569 | 0.0008 | Tumor hypermethylation in CpG island core |
| CDH23 | 64072 | 10 | 72822696 | 72830696 | 0.0009 | Tumor hypermethylation in CpG island core |
| CHST3 | 9469 | 10 | 73390125 | 73398125 | 0.0002 | Tumor hypermethylation in CpG island core |
| SPOCK2 | 9806 | 10 | 73514796 | 73522796 | 0.0004 | Tumor hypermethylation in CpG island core |
| ZNF503 | 84858 | 10 | 76827519 | 76835519 | 0 | Tumor hypermethylation in CpG island core |
| DYDC1 | 143241 | 10 | 82102480 | 82110480 | 0.0001 | Tumor hypermethylation in CpG island core |
| NRG3 | 10718 | 10 | 83621049 | 83629049 | 0.0001 | Tumor hypermethylation in CpG island core |
| PCDH21 | 92211 | 10 | 85940496 | 85948496 | 0.0048 | Tumor hypermethylation in CpG island core |
| GRID1 | 2894 | 10 | 88112230 | 88120230 | 0.0003 | Tumor hypermethylation in CpG island core |
| AGAP11 | 119385 | 10 | 88716477 | 88724477 | 0.0001 | Tumor hypermethylation in CpG island core |
| CH25H | 9023 | 10 | 90953051 | 90961051 | 0.0002 | Tumor hypermethylation in CpG island core |
| CYP26C1 | 340665 | 10 | 94807010 | 94815010 | 0 | Tumor hypermethylation in CpG island core |
| GPR120 | 338557 | 10 | 95312411 | 95320411 | 0 | Tumor hypermethylation in CpG island core |
| TLL2 | 7093 | 10 | 98259658 | 98267658 | 0.0007 | Tumor hypermethylation in CpG island core |
| SFRP5 | 6425 | 10 | 99517746 | 99525746 | 0.0003 | Tumor hypermethylation in CpG island core |
| CRTAC1 | 55118 | 10 | 99776575 | 99784575 | 0 | Tumor hypermethylation in CpG island core |
| SEC31B | 25956 | 10 | 102265585 | 102273585 | 0.0041 | Tumor hypermethylation in CpG island core |
| FGF8 | 2253 | 10 | 103521817 | 103529817 | 0.0011 | Tumor hypermethylation in CpG island core |
| PITX3 | 5309 | 10 | 103987221 | 103995221 | 0.0087 | Tumor hypermethylation in CpG island core |
| INA | 9118 | 10 | 105022909 | 105030909 | 0 | Tumor hypermethylation in CpG island core |
| SORCS3 | 22986 | 10 | 106386848 | 106394848 | 0 | Tumor hypermethylation in CpG island core |
| SORCS1 | 114815 | 10 | 108910282 | 108918282 | 0.0018 | Tumor hypermethylation in CpG island core |
| ADD3 | 120 | 10 | 111753700 | 111761700 | 0.0032 | Tumor hypermethylation in CpG island core |
| ADRB1 | 153 | 10 | 115789795 | 115797795 | 0.0012 | Tumor hypermethylation in CpG island core |
| EMX2OS | 196047 | 10 | 119290569 | 119298569 | 0 | Tumor hypermethylation in CpG island core |
| PRLHR | 2834 | 10 | 120341150 | 120349150 | 0 | Tumor hypermethylation in CpG island core |
| PPAPDC1A | 196051 | 10 | 122202455 | 122210455 | 0.003 | Tumor hypermethylation in CpG island core |
| TACC2 | 10579 | 10 | 123909094 | 123917094 | 0.0015 | Tumor hypermethylation in CpG island core |
| HTRA1 | 5654 | 10 | 124207030 | 124215030 | 0.0006 | Tumor hypermethylation in CpG island core |
| HMX3 | 340784 | 10 | 124881556 | 124889556 | 0 | Tumor hypermethylation in CpG island core |
| GPR26 | 2849 | 10 | 125411860 | 125419860 | 0.0096 | Tumor hypermethylation in CpG island core |
| NKX1-2 | 390010 | 10 | 126124540 | 126132540 | 0 | Tumor hypermethylation in CpG island core |
| DOCK1 | 1793 | 10 | 128580012 | 128588012 | 0.0076 | Tumor hypermethylation in CpG island core |
| FOXI2 | 399823 | 10 | 129421527 | 129429527 | 0 | Tumor hypermethylation in CpG island core |
| EBF3 | 253738 | 10 | 131648081 | 131656081 | 0 | Tumor hypermethylation in CpG island core |
| TCERG1L | 256536 | 10 | 132995974 | 133003974 | 0 | Tumor hypermethylation in CpG island core |
| DPYSL4 | 10570 | 10 | 133846403 | 133854403 | 0 | Tumor hypermethylation in CpG island core |
| NKX6-2 | 84504 | 10 | 134445527 | 134453527 | 0 | Tumor hypermethylation in CpG island core |
| UTF1 | 8433 | 10 | 134889767 | 134897767 | 0 | Tumor hypermethylation in CpG island core |
| VENTX | 27287 | 10 | 134897397 | 134905397 | 0 | Tumor hypermethylation in CpG island core |
| CALY | 50632 | 10 | 134996465 | 135004465 | 0.0003 | Tumor hypermethylation in CpG island core |
| CYP2E1 | 1571 | 10 | 135186856 | 135194856 | 0.0018 | Tumor hypermethylation in CpG island core |
| SCT | 6343 | 11 | 613173 | 621173 | 0.0026 | Tumor hypermethylation in CpG island core |
| DRD4 | 1815 | 11 | 623304 | 631304 | 0 | Tumor hypermethylation in CpG island core |
| AP2A2 | 161 | 11 | 911840 | 919840 | 0.0061 | Tumor hypermethylation in CpG island core |
| MUC6 | 4588 | 11 | 1022706 | 1030706 | 0.0002 | Tumor hypermethylation in CpG island core |
| LSP1 | 390387 | 11 | 1844674 | 1852674 | 0.0004 | Tumor hypermethylation in CpG island core |
| IGF2 | 3481 | 11 | 2112780 | 2120780 | 0.0001 | Tumor hypermethylation in CpG island core |
| ASCL2 | 430 | 11 | 2244758 | 2252758 | 0 | Tumor hypermethylation in CpG island core |
| KCNQ1 | 3784 | 11 | 2418796 | 2426796 | 0.0003 | Tumor hypermethylation in CpG island core |
| KCNQ1OT1 | 10984 | 11 | 2673804 | 2681804 | 0 | Tumor hypermethylation in CpG island core |
| KCNQ1DN | 55539 | 11 | 2843838 | 2851838 | 0.0049 | Tumor hypermethylation in CpG island core |
| PRKCDBP | 112464 | 11 | 6294316 | 6302316 | 0 | Tumor hypermethylation in CpG island core |
| ZNF215 | 7762 | 11 | 6900229 | 6908229 | 0.0025 | Tumor hypermethylation in CpG island core |
| RBMXL2 | 27288 | 11 | 7062740 | 7070740 | 0.0021 | Tumor hypermethylation in CpG island core |
| SYT9 | 143425 | 11 | 7225756 | 7233756 | 0.0063 | Tumor hypermethylation in CpG island core |
| RIC3 | 79608 | 11 | 8143166 | 8151166 | 0.0008 | Tumor hypermethylation in CpG island core |
| DKK3 | 27122 | 11 | 11982762 | 11990762 | 0.0062 | Tumor hypermethylation in CpG island core |
| RASSF10 | 644943 | 11 | 12983271 | 12991271 | 0 | Tumor hypermethylation in CpG island core |
| INSC | 387755 | 11 | 15089059 | 15097059 | 0.0035 | Tumor hypermethylation in CpG island core |
| DKFZp686O24166 | 374383 | 11 | 17325892 | 17333892 | 0.0022 | Tumor hypermethylation in CpG island core |
| ABCC8 | 6833 | 11 | 17451025 | 17459025 | 0.005 | Tumor hypermethylation in CpG island core |
| MYOD1 | 4654 | 11 | 17693685 | 17701685 | 0 | Tumor hypermethylation in CpG island core |
| KCNC1 | 3746 | 11 | 17710070 | 17718070 | 0.0035 | Tumor hypermethylation in CpG island core |
| PTPN5 | 84867 | 11 | 18765965 | 18773965 | 0 | Tumor hypermethylation in CpG island core |
| NAV2 | 89797 | 11 | 19687456 | 19695456 | 0 | Tumor hypermethylation in CpG island core |
| NELL1 | 4745 | 11 | 20643692 | 20651692 | 0 | Tumor hypermethylation in CpG island core |
| ANO5 | 203859 | 11 | 22167297 | 22175297 | 0.0002 | Tumor hypermethylation in CpG island core |
| LUZP2 | 338645 | 11 | 24471131 | 24479131 | 0.0001 | Tumor hypermethylation in CpG island core |
| KCNA4 | 3739 | 11 | 29991064 | 29999064 | 0.0002 | Tumor hypermethylation in CpG island core |
| WT1 | 7490 | 11 | 32409663 | 32417663 | 0 | Tumor hypermethylation in CpG island core |
| SLC1A2 | 6506 | 11 | 35393681 | 35401681 | 0.0034 | Tumor hypermethylation in CpG island core |
| FJX1 | 24147 | 11 | 35592310 | 35600310 | 0.0058 | Tumor hypermethylation in CpG island core |
| PRR5L | 79899 | 11 | 36350110 | 36358110 | 0.0016 | Tumor hypermethylation in CpG island core |
| ALX4 | 60529 | 11 | 44284292 | 44292292 | 0 | Tumor hypermethylation in CpG island core |
| DGKZ | 8525 | 11 | 46307314 | 46315314 | 0.0005 | Tumor hypermethylation in CpG island core |
| CHRM4 | 1132 | 11 | 46360683 | 46368683 | 0.0045 | Tumor hypermethylation in CpG island core |
| LRP4 | 4038 | 11 | 46892652 | 46900652 | 0.0009 | Tumor hypermethylation in CpG island core |
| PACSIN3 | 29763 | 11 | 47160534 | 47168534 | 0.0014 | Tumor hypermethylation in CpG island core |
| LRRC10B | 390205 | 11 | 61028847 | 61036847 | 0 | Tumor hypermethylation in CpG island core |
| FADS2 | 9415 | 11 | 61348288 | 61356288 | 0.0006 | Tumor hypermethylation in CpG island core |
| MTA2 | 9219 | 11 | 62121879 | 62129879 | 0.0065 | Tumor hypermethylation in CpG island core |
| MIR1237 | 1E+08 | 11 | 63888649 | 63896649 | 0 | Tumor hypermethylation in CpG island core |
| RASGRP2 | 10235 | 11 | 64264905 | 64272905 | 0.0001 | Tumor hypermethylation in CpG island core |
| TIGD3 | 220359 | 11 | 64874857 | 64882857 | 0.0048 | Tumor hypermethylation in CpG island core |
| SNX32 | 254122 | 11 | 65353985 | 65361985 | 0.0001 | Tumor hypermethylation in CpG island core |
| CD248 | 57124 | 11 | 65837091 | 65845091 | 0.0001 | Tumor hypermethylation in CpG island core |
| B3GNT1 | 11041 | 11 | 65867737 | 65875737 | 0.0072 | Tumor hypermethylation in CpG island core |
| NPAS4 | 266743 | 11 | 65941050 | 65949050 | 0 | Tumor hypermethylation in CpG island core |
| ACTN3 | 89 | 11 | 66066966 | 66074966 | 0.0063 | Tumor hypermethylation in CpG island core |
| SSH3 | 54961 | 11 | 66823494 | 66831494 | 0 | Tumor hypermethylation in CpG island core |
| GSTP1 | 2950 | 11 | 67103641 | 67111641 | 0 | Tumor hypermethylation in CpG island core |
| HBM | 4041 | 11 | 67832683 | 67840683 | 0.0068 | Tumor hypermethylation in CpG island core |
| CPT1A | 1374 | 11 | 68361975 | 68369975 | 0.0003 | Tumor hypermethylation in CpG island core |
| FGF19 | 9965 | 11 | 69224287 | 69232287 | 0 | Tumor hypermethylation in CpG island core |
| FGF4 | 2249 | 11 | 69295352 | 69303352 | 0 | Tumor hypermethylation in CpG island core |
| FGF3 | 2248 | 11 | 69339129 | 69347129 | 0 | Tumor hypermethylation in CpG island core |
| PHOX2A | 401 | 11 | 71628868 | 71636868 | 0 | Tumor hypermethylation in CpG island core |
| KCNE3 | 10008 | 11 | 73852248 | 73860248 | 0.0005 | Tumor hypermethylation in CpG island core |
| CHRDL2 | 25884 | 11 | 74115834 | 74123834 | 0.0082 | Tumor hypermethylation in CpG island core |
| MAP6 | 4135 | 11 | 75053127 | 75061127 | 0.0026 | Tumor hypermethylation in CpG island core |
| LRRC32 | 2615 | 11 | 76055439 | 76063439 | 0.0007 | Tumor hypermethylation in CpG island core |
| FAM181B | 220382 | 11 | 82118554 | 82126554 | 0.0005 | Tumor hypermethylation in CpG island core |
| ME3 | 56110 | 11 | 86056888 | 86064888 | 0.0004 | Tumor hypermethylation in CpG island core |
| RAB38 | 23682 | 11 | 87544247 | 87552247 | 0.0002 | Tumor hypermethylation in CpG island core |
| NOX4 | 50507 | 11 | 88860301 | 88868301 | 0.0046 | Tumor hypermethylation in CpG island core |
| MTNR1B | 4544 | 11 | 92338436 | 92346436 | 0.0003 | Tumor hypermethylation in CpG island core |
| GPR83 | 10888 | 11 | 93770233 | 93778233 | 0.0019 | Tumor hypermethylation in CpG island core |
| AMOTL1 | 154810 | 11 | 94137155 | 94145155 | 0.0001 | Tumor hypermethylation in CpG island core |
| TRPC6 | 7225 | 11 | 100955869 | 100963869 | 0 | Tumor hypermethylation in CpG island core |
| GRIA4 | 2893 | 11 | 104982620 | 104990620 | 0.0001 | Tumor hypermethylation in CpG island core |
| MIR34B | 407041 | 11 | 110884872 | 110892872 | 0.0018 | Tumor hypermethylation in CpG island core |
| LAYN | 143903 | 11 | 110912442 | 110920442 | 0.0001 | Tumor hypermethylation in CpG island core |
| BCL9L | 283149 | 11 | 118282823 | 118290823 | 0.0012 | Tumor hypermethylation in CpG island core |
| ASAM | 79827 | 11 | 122567217 | 122575217 | 0.0009 | Tumor hypermethylation in CpG island core |
| ROBO3 | 64221 | 11 | 124236514 | 124244514 | 0 | Tumor hypermethylation in CpG island core |
| FEZ1 | 11178 | 11 | 124867333 | 124875333 | 0.0061 | Tumor hypermethylation in CpG island core |
| ETS1 | 2113 | 11 | 127893415 | 127901415 | 0 | Tumor hypermethylation in CpG island core |
| OPCML | 4978 | 11 | 132314247 | 132322247 | 0.0025 | Tumor hypermethylation in CpG island core |
| IGSF9B | 22997 | 11 | 133328090 | 133336090 | 0.0002 | Tumor hypermethylation in CpG island core |
| JAM3 | 83700 | 11 | 133440029 | 133448029 | 0.0045 | Tumor hypermethylation in CpG island core |
| GLB1L3 | 112937 | 11 | 133647484 | 133655484 | 0 | Tumor hypermethylation in CpG island core |
| B3GAT1 | 27087 | 11 | 133783022 | 133791022 | 0.0002 | Tumor hypermethylation in CpG island core |
| PRMT8 | 56341 | 12 | 3466685 | 3474685 | 0 | Tumor hypermethylation in CpG island core |
| CCND2 | 894 | 12 | 4249162 | 4257162 | 0 | Tumor hypermethylation in CpG island core |
| KCNA6 | 3742 | 12 | 4784602 | 4792602 | 0.0006 | Tumor hypermethylation in CpG island core |
| KCNA1 | 3736 | 12 | 4885333 | 4893333 | 0 | Tumor hypermethylation in CpG island core |
| KCNA5 | 3741 | 12 | 5019345 | 5027345 | 0.0073 | Tumor hypermethylation in CpG island core |
| NTF3 | 4908 | 12 | 5407540 | 5415540 | 0 | Tumor hypermethylation in CpG island core |
| LTBR | 4055 | 12 | 6359617 | 6367617 | 0.0007 | Tumor hypermethylation in CpG island core |
| IFFO1 | 25900 | 12 | 6524535 | 6532535 | 0.0098 | Tumor hypermethylation in CpG island core |
| SLC2A14 | 144195 | 12 | 7912762 | 7920762 | 0.0033 | Tumor hypermethylation in CpG island core |
| PDE3A | 5139 | 12 | 20409463 | 20417463 | 0 | Tumor hypermethylation in CpG island core |
| ST8SIA1 | 6489 | 12 | 22374915 | 22382915 | 0.0015 | Tumor hypermethylation in CpG island core |
| BCAT1 | 586 | 12 | 24989575 | 24997575 | 0.0009 | Tumor hypermethylation in CpG island core |
| CPNE8 | 144402 | 12 | 37581687 | 37589687 | 0 | Tumor hypermethylation in CpG island core |
| ADAMTS20 | 80070 | 12 | 42227991 | 42235991 | 0 | Tumor hypermethylation in CpG island core |
| DBX2 | 440097 | 12 | 43727149 | 43735149 | 0 | Tumor hypermethylation in CpG island core |
| COL2A1 | 1280 | 12 | 46680552 | 46688552 | 0 | Tumor hypermethylation in CpG island core |
| C12orf68 | 387856 | 12 | 46859632 | 46867632 | 0.0004 | Tumor hypermethylation in CpG island core |
| PRPH | 5961 | 12 | 47971175 | 47979175 | 0.0001 | Tumor hypermethylation in CpG island core |
| FAIM2 | 23017 | 12 | 48579987 | 48587987 | 0.0058 | Tumor hypermethylation in CpG island core |
| FIGNL2 | 401720 | 12 | 50498475 | 50506475 | 0.0079 | Tumor hypermethylation in CpG island core |
| ACVRL1 | 94 | 12 | 50583468 | 50591468 | 0.0002 | Tumor hypermethylation in CpG island core |
| GRASP | 160622 | 12 | 50683014 | 50691014 | 0 | Tumor hypermethylation in CpG island core |
| ATP5G2 | 517 | 12 | 52352376 | 52360376 | 0.0029 | Tumor hypermethylation in CpG island core |
| HOXC13 | 3229 | 12 | 52614842 | 52622842 | 0 | Tumor hypermethylation in CpG island core |
| HOXC11 | 3227 | 12 | 52649176 | 52657176 | 0.0008 | Tumor hypermethylation in CpG island core |
| HOXC10 | 3226 | 12 | 52661212 | 52669212 | 0.0042 | Tumor hypermethylation in CpG island core |
| HOXC8 | 3224 | 12 | 52685156 | 52693156 | 0.0015 | Tumor hypermethylation in CpG island core |
| HOXC4 | 3221 | 12 | 52729927 | 52737927 | 0 | Tumor hypermethylation in CpG island core |
| LOC400043 | 400043 | 12 | 52802121 | 52810121 | 0.0008 | Tumor hypermethylation in CpG island core |
| ITGA5 | 3678 | 12 | 53095317 | 53103317 | 0.0035 | Tumor hypermethylation in CpG island core |
| KIF5A | 3798 | 12 | 56226113 | 56234113 | 0.0065 | Tumor hypermethylation in CpG island core |
| GEFT | 115557 | 12 | 56286229 | 56294229 | 0 | Tumor hypermethylation in CpG island core |
| LOC100130776 | 1E+08 | 12 | 56402289 | 56410289 | 0.0001 | Tumor hypermethylation in CpG island core |
| AGAP2 | 116986 | 12 | 56414296 | 56422296 | 0 | Tumor hypermethylation in CpG island core |
| FAM19A2 | 338811 | 12 | 60868818 | 60876818 | 0.0003 | Tumor hypermethylation in CpG island core |
| HMGA2 | 8091 | 12 | 64500506 | 64508506 | 0 | Tumor hypermethylation in CpG island core |
| LGR5 | 8549 | 12 | 70116079 | 70124079 | 0 | Tumor hypermethylation in CpG island core |
| LOC283392 | 283392 | 12 | 70949556 | 70957556 | 0 | Tumor hypermethylation in CpG island core |
| MYF6 | 4618 | 12 | 79621576 | 79629576 | 0.0069 | Tumor hypermethylation in CpG island core |
| SLC6A15 | 55117 | 12 | 83826737 | 83834737 | 0.001 | Tumor hypermethylation in CpG island core |
| ALX1 | 8092 | 12 | 84194166 | 84202166 | 0 | Tumor hypermethylation in CpG island core |
| C12orf12 | 196477 | 12 | 89869084 | 89877084 | 0.0077 | Tumor hypermethylation in CpG island core |
| USP44 | 84101 | 12 | 94462751 | 94470751 | 0.0002 | Tumor hypermethylation in CpG island core |
| SLC5A8 | 160728 | 12 | 100124147 | 100132147 | 0.0009 | Tumor hypermethylation in CpG island core |
| ASCL1 | 429 | 12 | 101871581 | 101879581 | 0 | Tumor hypermethylation in CpG island core |
| TXNRD1 | 7296 | 12 | 103129688 | 103137688 | 0.0006 | Tumor hypermethylation in CpG island core |
| EID3 | 493861 | 12 | 103217678 | 103225678 | 0.0003 | Tumor hypermethylation in CpG island core |
| CHST11 | 50515 | 12 | 103370907 | 103378907 | 0 | Tumor hypermethylation in CpG island core |
| BTBD11 | 121551 | 12 | 106232326 | 106240326 | 0 | Tumor hypermethylation in CpG island core |
| ASCL4 | 121549 | 12 | 106688291 | 106696291 | 0.0091 | Tumor hypermethylation in CpG island core |
| FOXN4 | 121643 | 12 | 108227408 | 108235408 | 0.0001 | Tumor hypermethylation in CpG island core |
| ALDH2 | 217 | 12 | 110684728 | 110692728 | 0.0033 | Tumor hypermethylation in CpG island core |
| LHX5 | 64211 | 12 | 112390260 | 112398260 | 0 | Tumor hypermethylation in CpG island core |
| TBX5 | 6910 | 12 | 113322086 | 113330086 | 0.0001 | Tumor hypermethylation in CpG island core |
| NOS1 | 340719 | 12 | 116279965 | 116287965 | 0.0002 | Tumor hypermethylation in CpG island core |
| SRRM4 | 84530 | 12 | 117899778 | 117907778 | 0 | Tumor hypermethylation in CpG island core |
| B3GNT4 | 79369 | 12 | 121250180 | 121258180 | 0.0088 | Tumor hypermethylation in CpG island core |
| TMEM132C | 92293 | 12 | 127313900 | 127321900 | 0 | Tumor hypermethylation in CpG island core |
| TMEM132D | 121256 | 12 | 128950165 | 128958165 | 0 | Tumor hypermethylation in CpG island core |
| FZD10 | 11211 | 12 | 129208984 | 129216984 | 0 | Tumor hypermethylation in CpG island core |
| PIWIL1 | 9271 | 12 | 129384566 | 129392566 | 0.0003 | Tumor hypermethylation in CpG island core |
| GALNT9 | 50614 | 12 | 131411978 | 131419978 | 0.0087 | Tumor hypermethylation in CpG island core |
| P2RX2 | 22953 | 12 | 131701475 | 131709475 | 0 | Tumor hypermethylation in CpG island core |
| GJB2 | 2706 | 13 | 19661114 | 19669114 | 0.006 | Tumor hypermethylation in CpG island core |
| FAM123A | 219287 | 13 | 24639857 | 24647857 | 0 | Tumor hypermethylation in CpG island core |
| SHISA2 | 387914 | 13 | 25519198 | 25527198 | 0 | Tumor hypermethylation in CpG island core |
| GSX1 | 219409 | 13 | 27260779 | 27268779 | 0 | Tumor hypermethylation in CpG island core |
| CDX2 | 1045 | 13 | 27437317 | 27445317 | 0 | Tumor hypermethylation in CpG island core |
| FLT1 | 2321 | 13 | 27963265 | 27971265 | 0.0001 | Tumor hypermethylation in CpG island core |
| C13orf33 | 84935 | 13 | 30374311 | 30382311 | 0.0005 | Tumor hypermethylation in CpG island core |
| KL | 9365 | 13 | 32484570 | 32492570 | 0 | Tumor hypermethylation in CpG island core |
| MAB21L1 | 4081 | 13 | 34944832 | 34952832 | 0.0013 | Tumor hypermethylation in CpG island core |
| CCNA1 | 8900 | 13 | 35899966 | 35907966 | 0 | Tumor hypermethylation in CpG island core |
| FREM2 | 341640 | 13 | 38155172 | 38163172 | 0.0004 | Tumor hypermethylation in CpG island core |
| TNFSF11 | 8600 | 13 | 42042290 | 42050290 | 0 | Tumor hypermethylation in CpG island core |
| MLNR | 2862 | 13 | 48688474 | 48696474 | 0.0032 | Tumor hypermethylation in CpG island core |
| LECT1 | 11061 | 13 | 52207948 | 52215948 | 0.0002 | Tumor hypermethylation in CpG island core |
| PCDH8 | 5100 | 13 | 52316775 | 52324775 | 0 | Tumor hypermethylation in CpG island core |
| PCDH9 | 5101 | 13 | 66698469 | 66706469 | 0 | Tumor hypermethylation in CpG island core |
| ATXN8OS | 6315 | 13 | 69575345 | 69583345 | 0.0003 | Tumor hypermethylation in CpG island core |
| KCTD12 | 115207 | 13 | 76354541 | 76362541 | 0.0007 | Tumor hypermethylation in CpG island core |
| EDNRB | 1910 | 13 | 77386967 | 77394967 | 0.0002 | Tumor hypermethylation in CpG island core |
| POU4F1 | 5457 | 13 | 78071696 | 78079696 | 0 | Tumor hypermethylation in CpG island core |
| SLITRK5 | 26050 | 13 | 87118870 | 87126870 | 0.0002 | Tumor hypermethylation in CpG island core |
| GPC5 | 2262 | 13 | 90844935 | 90852935 | 0.0025 | Tumor hypermethylation in CpG island core |
| GPC6 | 10082 | 13 | 92673078 | 92681078 | 0 | Tumor hypermethylation in CpG island core |
| SOX21 | 11166 | 13 | 94158390 | 94166390 | 0 | Tumor hypermethylation in CpG island core |
| DZIP1 | 22873 | 13 | 95090958 | 95098958 | 0.0005 | Tumor hypermethylation in CpG island core |
| RNF113B | 140432 | 13 | 97623522 | 97631522 | 0.0018 | Tumor hypermethylation in CpG island core |
| NALCN | 259232 | 13 | 100862814 | 100870814 | 0.0056 | Tumor hypermethylation in CpG island core |
| FGF14 | 2259 | 13 | 101362996 | 101370996 | 0 | Tumor hypermethylation in CpG island core |
| FAM155A | 728215 | 13 | 107313461 | 107321461 | 0 | Tumor hypermethylation in CpG island core |
| COL4A1 | 1282 | 13 | 109753497 | 109761497 | 0 | Tumor hypermethylation in CpG island core |
| COL4A2 | 1284 | 13 | 109753631 | 109761631 | 0 | Tumor hypermethylation in CpG island core |
| SOX1 | 6656 | 13 | 111765913 | 111773913 | 0 | Tumor hypermethylation in CpG island core |
| GRTP1 | 79774 | 13 | 113062464 | 113070464 | 0.0097 | Tumor hypermethylation in CpG island core |
| FLJ44054 | 643365 | 13 | 113523333 | 113531333 | 0.0001 | Tumor hypermethylation in CpG island core |
| MMP14 | 4323 | 14 | 22371632 | 22379632 | 0 | Tumor hypermethylation in CpG island core |
| EFS | 10278 | 14 | 22900682 | 22908682 | 0.0019 | Tumor hypermethylation in CpG island core |
| REC8 | 60412 | 14 | 23707073 | 23715073 | 0.0001 | Tumor hypermethylation in CpG island core |
| CIDEB | 27141 | 14 | 23846416 | 23854416 | 0.0039 | Tumor hypermethylation in CpG island core |
| ADCY4 | 196883 | 14 | 23869704 | 23877704 | 0.0001 | Tumor hypermethylation in CpG island core |
| STXBP6 | 29091 | 14 | 24584935 | 24592935 | 0 | Tumor hypermethylation in CpG island core |
| NOVA1 | 4857 | 14 | 26132800 | 26140800 | 0 | Tumor hypermethylation in CpG island core |
| FOXG1 | 2290 | 14 | 28302037 | 28310037 | 0 | Tumor hypermethylation in CpG island core |
| INSM2 | 84684 | 14 | 35068998 | 35076998 | 0.0001 | Tumor hypermethylation in CpG island core |
| SFTA3 | 253970 | 14 | 36048741 | 36056741 | 0 | Tumor hypermethylation in CpG island core |
| PAX9 | 5083 | 14 | 36192523 | 36200523 | 0 | Tumor hypermethylation in CpG island core |
| CLEC14A | 161198 | 14 | 37791325 | 37799325 | 0 | Tumor hypermethylation in CpG island core |
| LRFN5 | 145581 | 14 | 41142513 | 41150513 | 0 | Tumor hypermethylation in CpG island core |
| MDGA2 | 161357 | 14 | 47209738 | 47217738 | 0 | Tumor hypermethylation in CpG island core |
| ABHD12B | 145447 | 14 | 50404627 | 50412627 | 0.0093 | Tumor hypermethylation in CpG island core |
| TRIM9 | 114088 | 14 | 50628172 | 50636172 | 0.0003 | Tumor hypermethylation in CpG island core |
| NID2 | 22795 | 14 | 51601696 | 51609696 | 0 | Tumor hypermethylation in CpG island core |
| PTGDR | 5729 | 14 | 51800180 | 51808180 | 0 | Tumor hypermethylation in CpG island core |
| PTGER2 | 5732 | 14 | 51846765 | 51854765 | 0 | Tumor hypermethylation in CpG island core |
| BMP4 | 652 | 14 | 53487020 | 53495020 | 0 | Tumor hypermethylation in CpG island core |
| OTX2 | 5015 | 14 | 56342937 | 56350937 | 0 | Tumor hypermethylation in CpG island core |
| C14orf39 | 317761 | 14 | 60018517 | 60026517 | 0.0001 | Tumor hypermethylation in CpG island core |
| SIX6 | 4990 | 14 | 60041690 | 60049690 | 0 | Tumor hypermethylation in CpG island core |
| SIX1 | 6495 | 14 | 60181908 | 60189908 | 0 | Tumor hypermethylation in CpG island core |
| FLJ43390 | 646113 | 14 | 61649827 | 61657827 | 0.0011 | Tumor hypermethylation in CpG island core |
| ESR2 | 2100 | 14 | 63826881 | 63834881 | 0.0048 | Tumor hypermethylation in CpG island core |
| HSPA2 | 3306 | 14 | 64072938 | 64080938 | 0.0006 | Tumor hypermethylation in CpG island core |
| VSX2 | 338917 | 14 | 73771927 | 73779927 | 0 | Tumor hypermethylation in CpG island core |
| LTBP2 | 4054 | 14 | 74144787 | 74152787 | 0.0085 | Tumor hypermethylation in CpG island core |
| VASH1 | 22846 | 14 | 76293987 | 76301987 | 0.0004 | Tumor hypermethylation in CpG island core |
| NGB | 58157 | 14 | 76803408 | 76811408 | 0.0066 | Tumor hypermethylation in CpG island core |
| ISM2 | 145501 | 14 | 77030963 | 77038963 | 0.0096 | Tumor hypermethylation in CpG island core |
| FLRT2 | 23768 | 14 | 85062240 | 85070240 | 0 | Tumor hypermethylation in CpG island core |
| GALC | 2581 | 14 | 87525660 | 87533660 | 0.0089 | Tumor hypermethylation in CpG island core |
| KCNK13 | 56659 | 14 | 89593860 | 89601860 | 0 | Tumor hypermethylation in CpG island core |
| FBLN5 | 10516 | 14 | 91479799 | 91487799 | 0.0007 | Tumor hypermethylation in CpG island core |
| RIN3 | 79890 | 14 | 92045877 | 92053877 | 0.0003 | Tumor hypermethylation in CpG island core |
| CHGA | 1113 | 14 | 92455197 | 92463197 | 0.0013 | Tumor hypermethylation in CpG island core |
| BCL11B | 64919 | 14 | 98803575 | 98811575 | 0 | Tumor hypermethylation in CpG island core |
| BEGAIN | 57596 | 14 | 100100160 | 100108160 | 0 | Tumor hypermethylation in CpG island core |
| DLK1 | 8788 | 14 | 100259005 | 100267005 | 0.0046 | Tumor hypermethylation in CpG island core |
| MEG3 | 55384 | 14 | 100358197 | 100366197 | 0 | Tumor hypermethylation in CpG island core |
| MIR127 | 406914 | 14 | 100415068 | 100423068 | 0 | Tumor hypermethylation in CpG island core |
| MIR1247 | 1E+08 | 14 | 101092512 | 101100512 | 0.0005 | Tumor hypermethylation in CpG island core |
| CKB | 1152 | 14 | 103054923 | 103062923 | 0.0059 | Tumor hypermethylation in CpG island core |
| TDRD9 | 122402 | 14 | 103460569 | 103468569 | 0.0025 | Tumor hypermethylation in CpG island core |
| ASPG | 374569 | 14 | 103617800 | 103625800 | 0.005 | Tumor hypermethylation in CpG island core |
| TMEM179 | 388021 | 14 | 104138142 | 104146142 | 0.002 | Tumor hypermethylation in CpG island core |
| CRIP1 | 25927 | 14 | 105020301 | 105028301 | 0.0067 | Tumor hypermethylation in CpG island core |
| SNURF | 8926 | 15 | 22747162 | 22755162 | 0.0057 | Tumor hypermethylation in CpG island core |
| GABRB3 | 2562 | 15 | 24565344 | 24573344 | 0 | Tumor hypermethylation in CpG island core |
| GABRA5 | 2558 | 15 | 24659365 | 24667365 | 0 | Tumor hypermethylation in CpG island core |
| FAM189A1 | 23359 | 15 | 27646219 | 27654219 | 0 | Tumor hypermethylation in CpG island core |
| GREM1 | 26585 | 15 | 30793496 | 30801496 | 0 | Tumor hypermethylation in CpG island core |
| GJD2 | 57369 | 15 | 32829981 | 32837981 | 0 | Tumor hypermethylation in CpG island core |
| MEIS2 | 4212 | 15 | 35173795 | 35181795 | 0 | Tumor hypermethylation in CpG island core |
| GPR176 | 11245 | 15 | 37996385 | 38004385 | 0.003 | Tumor hypermethylation in CpG island core |
| ITPKA | 3706 | 15 | 39569413 | 39577413 | 0.0095 | Tumor hypermethylation in CpG island core |
| LTK | 4058 | 15 | 39589377 | 39597377 | 0 | Tumor hypermethylation in CpG island core |
| MGA | 23269 | 15 | 39735901 | 39743901 | 0.0013 | Tumor hypermethylation in CpG island core |
| CATSPER2 | 117155 | 15 | 41724331 | 41732331 | 0.0084 | Tumor hypermethylation in CpG island core |
| DUOXA1 | 90527 | 15 | 43205349 | 43213349 | 0 | Tumor hypermethylation in CpG island core |
| SEMA6D | 80031 | 15 | 45793977 | 45801977 | 0.0001 | Tumor hypermethylation in CpG island core |
| FBN1 | 2200 | 15 | 46721277 | 46729277 | 0 | Tumor hypermethylation in CpG island core |
| GLDN | 342035 | 15 | 49417004 | 49425004 | 0.0029 | Tumor hypermethylation in CpG island core |
| ALDH1A2 | 8854 | 15 | 56141198 | 56149198 | 0.0003 | Tumor hypermethylation in CpG island core |
| FOXB1 | 27023 | 15 | 58079712 | 58087712 | 0 | Tumor hypermethylation in CpG island core |
|  | 0 | 15 | 63152206 | 63160206 | 0 | Tumor hypermethylation in CpG island core |
| MEGF11 | 84465 | 15 | 64329129 | 64337129 | 0.0059 | Tumor hypermethylation in CpG island core |
| ITGA11 | 22801 | 15 | 66507546 | 66515546 | 0.0059 | Tumor hypermethylation in CpG island core |
| GRAMD2 | 196996 | 15 | 70273190 | 70281190 | 0.0027 | Tumor hypermethylation in CpG island core |
| HCN4 | 10021 | 15 | 71444658 | 71452658 | 0.0001 | Tumor hypermethylation in CpG island core |
| LOC283731 | 283731 | 15 | 72204672 | 72212672 | 0.0001 | Tumor hypermethylation in CpG island core |
| ISLR | 3671 | 15 | 72249957 | 72257957 | 0.0012 | Tumor hypermethylation in CpG island core |
| SCAMP5 | 192683 | 15 | 73070953 | 73078953 | 0.0051 | Tumor hypermethylation in CpG island core |
| ISL2 | 64843 | 15 | 74412201 | 74420201 | 0 | Tumor hypermethylation in CpG island core |
| CHRNA3 | 1136 | 15 | 76696692 | 76704692 | 0.0066 | Tumor hypermethylation in CpG island core |
| ADAMTS7 | 11173 | 15 | 76886828 | 76894828 | 0.0057 | Tumor hypermethylation in CpG island core |
| RASGRF1 | 5923 | 15 | 77166270 | 77174270 | 0.0002 | Tumor hypermethylation in CpG island core |
| ANKRD34C | 390616 | 15 | 77358200 | 77366200 | 0 | Tumor hypermethylation in CpG island core |
| CPEB1 | 64506 | 15 | 81109783 | 81117783 | 0 | Tumor hypermethylation in CpG island core |
| TM6SF1 | 53346 | 15 | 81563327 | 81571327 | 0.0096 | Tumor hypermethylation in CpG island core |
| BNC1 | 646 | 15 | 81740472 | 81748472 | 0 | Tumor hypermethylation in CpG island core |
| ADAMTSL3 | 57188 | 15 | 82109841 | 82117841 | 0.0006 | Tumor hypermethylation in CpG island core |
| ALPK3 | 57538 | 15 | 83156914 | 83164914 | 0.0041 | Tumor hypermethylation in CpG island core |
| NTRK3 | 4916 | 15 | 86596665 | 86604665 | 0 | Tumor hypermethylation in CpG island core |
| ACAN | 176 | 15 | 87143677 | 87151677 | 0.0001 | Tumor hypermethylation in CpG island core |
| HAPLN3 | 145864 | 15 | 87235774 | 87243774 | 0.0001 | Tumor hypermethylation in CpG island core |
| MIR9-3 | 407051 | 15 | 87708251 | 87716251 | 0 | Tumor hypermethylation in CpG island core |
| LOC254559 | 254559 | 15 | 87718276 | 87726276 | 0.0003 | Tumor hypermethylation in CpG island core |
| MESP2 | 145873 | 15 | 88116592 | 88124592 | 0.0038 | Tumor hypermethylation in CpG island core |
| ANPEP | 290 | 15 | 88155076 | 88163076 | 0.001 | Tumor hypermethylation in CpG island core |
| C15orf38 | 348110 | 15 | 88253226 | 88261226 | 0.0064 | Tumor hypermethylation in CpG island core |
| FES | 2242 | 15 | 89224668 | 89232668 | 0.0001 | Tumor hypermethylation in CpG island core |
| ST8SIA2 | 8128 | 15 | 90734143 | 90742143 | 0 | Tumor hypermethylation in CpG island core |
| RGMA | 56963 | 15 | 91413393 | 91421393 | 0.0096 | Tumor hypermethylation in CpG island core |
| SYNM | 23336 | 15 | 97458808 | 97466808 | 0.0063 | Tumor hypermethylation in CpG island core |
| LRRK1 | 79705 | 15 | 99272982 | 99280982 | 0 | Tumor hypermethylation in CpG island core |
| HBM | 4041 | 16 | 151972 | 159972 | 0 | Tumor hypermethylation in CpG island core |
| ARHGDIG | 398 | 16 | 266606 | 274606 | 0.0012 | Tumor hypermethylation in CpG island core |
| LOC146336 | 146336 | 16 | 1064732 | 1072732 | 0 | Tumor hypermethylation in CpG island core |
| C16orf38 | 390667 | 16 | 1474469 | 1482469 | 0 | Tumor hypermethylation in CpG island core |
| C16orf73 | 254528 | 16 | 1858180 | 1866180 | 0.0035 | Tumor hypermethylation in CpG island core |
| HS3ST6 | 64711 | 16 | 1904232 | 1912232 | 0 | Tumor hypermethylation in CpG island core |
| ABCA3 | 21 | 16 | 2326748 | 2334748 | 0.0015 | Tumor hypermethylation in CpG island core |
| TESSP1 | 360226 | 16 | 2784486 | 2792486 | 0.0034 | Tumor hypermethylation in CpG island core |
| PRSS21 | 10942 | 16 | 2803164 | 2811164 | 0.0086 | Tumor hypermethylation in CpG island core |
| CLDN9 | 9080 | 16 | 2998457 | 3006457 | 0 | Tumor hypermethylation in CpG island core |
| CLDN6 | 9074 | 16 | 3004189 | 3012189 | 0 | Tumor hypermethylation in CpG island core |
| MMP28 | 79148 | 16 | 3032682 | 3040682 | 0.0001 | Tumor hypermethylation in CpG island core |
| ZNF597 | 146434 | 16 | 3429491 | 3437491 | 0.0003 | Tumor hypermethylation in CpG island core |
| CLUAP1 | 23059 | 16 | 3486963 | 3494963 | 0.0014 | Tumor hypermethylation in CpG island core |
| C16orf5 | 29965 | 16 | 4524817 | 4532817 | 0.0053 | Tumor hypermethylation in CpG island core |
| A2BP1 | 54715 | 16 | 6005132 | 6013132 | 0 | Tumor hypermethylation in CpG island core |
| GRIN2A | 2903 | 16 | 10180112 | 10188112 | 0 | Tumor hypermethylation in CpG island core |
| ITPRIPL2 | 162073 | 16 | 19028754 | 19036754 | 0.0094 | Tumor hypermethylation in CpG island core |
| GPRC5B | 51704 | 16 | 19799652 | 19807652 | 0.0003 | Tumor hypermethylation in CpG island core |
| GPR139 | 124274 | 16 | 19988601 | 19996601 | 0.0001 | Tumor hypermethylation in CpG island core |
| HS3ST2 | 9956 | 16 | 22729360 | 22737360 | 0 | Tumor hypermethylation in CpG island core |
| CHP2 | 63928 | 16 | 23669448 | 23677448 | 0.0083 | Tumor hypermethylation in CpG island core |
| PRKCB | 5579 | 16 | 23750800 | 23758800 | 0 | Tumor hypermethylation in CpG island core |
| CACNG3 | 10368 | 16 | 24170376 | 24178376 | 0.0017 | Tumor hypermethylation in CpG island core |
| HS3ST4 | 9951 | 16 | 25606847 | 25614847 | 0 | Tumor hypermethylation in CpG island core |
| SLC7A5P1 | 81893 | 16 | 29528539 | 29536539 | 0.002 | Tumor hypermethylation in CpG island core |
| DOC2A | 8448 | 16 | 29925902 | 29933902 | 0.0099 | Tumor hypermethylation in CpG island core |
| COX6A2 | 1339 | 16 | 31343222 | 31351222 | 0.0038 | Tumor hypermethylation in CpG island core |
| TGFB1I1 | 7041 | 16 | 31386976 | 31394976 | 0.0041 | Tumor hypermethylation in CpG island core |
| CSDAP1 | 440359 | 16 | 31484346 | 31492346 | 0.0079 | Tumor hypermethylation in CpG island core |
| MIR1826 | 1E+08 | 16 | 33869008 | 33877008 | 0 | Tumor hypermethylation in CpG island core |
| CBLN1 | 869 | 16 | 47869216 | 47877216 | 0 | Tumor hypermethylation in CpG island core |
| MT1E | 4493 | 16 | 55213085 | 55221085 | 0.0015 | Tumor hypermethylation in CpG island core |
| KIFC3 | 3801 | 16 | 56389940 | 56397940 | 0.0004 | Tumor hypermethylation in CpG island core |
| CDH8 | 1006 | 16 | 60623537 | 60631537 | 0 | Tumor hypermethylation in CpG island core |
| CDH11 | 1009 | 16 | 63709420 | 63717420 | 0 | Tumor hypermethylation in CpG island core |
| BEAN | 146227 | 16 | 65014740 | 65022740 | 0.0011 | Tumor hypermethylation in CpG island core |
| CMTM2 | 146225 | 16 | 65166851 | 65174851 | 0.0025 | Tumor hypermethylation in CpG island core |
| CCDC79 | 283847 | 16 | 65389024 | 65397024 | 0.0048 | Tumor hypermethylation in CpG island core |
| CA7 | 766 | 16 | 65431782 | 65439782 | 0.0002 | Tumor hypermethylation in CpG island core |
| RRAD | 6236 | 16 | 65512940 | 65520940 | 0.0007 | Tumor hypermethylation in CpG island core |
| HSF4 | 3299 | 16 | 65750788 | 65758788 | 0.0003 | Tumor hypermethylation in CpG island core |
| ELMO3 | 79767 | 16 | 65786528 | 65794528 | 0.0001 | Tumor hypermethylation in CpG island core |
| TPPP3 | 51673 | 16 | 65980922 | 65988922 | 0.0002 | Tumor hypermethylation in CpG island core |
| THAP11 | 57215 | 16 | 66429713 | 66437713 | 0.0094 | Tumor hypermethylation in CpG island core |
| NFATC3 | 4775 | 16 | 66672875 | 66680875 | 0.0011 | Tumor hypermethylation in CpG island core |
| ESRP2 | 80004 | 16 | 66823637 | 66831637 | 0.0072 | Tumor hypermethylation in CpG island core |
| VAT1L | 57687 | 16 | 76375983 | 76383983 | 0 | Tumor hypermethylation in CpG island core |
| CDH13 | 1012 | 16 | 81214078 | 81222078 | 0 | Tumor hypermethylation in CpG island core |
| FOXC2 | 2303 | 16 | 85154357 | 85162357 | 0 | Tumor hypermethylation in CpG island core |
| FBXO31 | 79791 | 16 | 85970895 | 85978895 | 0.0015 | Tumor hypermethylation in CpG island core |
| JPH3 | 57338 | 16 | 86189999 | 86197999 | 0.0001 | Tumor hypermethylation in CpG island core |
| CPNE7 | 27132 | 16 | 88165676 | 88173676 | 0.0001 | Tumor hypermethylation in CpG island core |
| LOC730755 | 730755 | 17 | 289129 | 297129 | 0.0058 | Tumor hypermethylation in CpG island core |
| BHLHA9 | 727857 | 17 | 1116607 | 1124607 | 0.0013 | Tumor hypermethylation in CpG island core |
| ALOX15 | 246 | 17 | 4487709 | 4495709 | 0.0001 | Tumor hypermethylation in CpG island core |
| VMO1 | 284013 | 17 | 4632469 | 4640469 | 0.0002 | Tumor hypermethylation in CpG island core |
| C17orf107 | 1E+08 | 17 | 4739726 | 4747726 | 0 | Tumor hypermethylation in CpG island core |
| WSCD1 | 23302 | 17 | 5910657 | 5918657 | 0.0044 | Tumor hypermethylation in CpG island core |
| SLC13A5 | 284111 | 17 | 6553464 | 6561464 | 0.0001 | Tumor hypermethylation in CpG island core |
| ALOX12 | 239 | 17 | 6836107 | 6844107 | 0 | Tumor hypermethylation in CpG island core |
| BCL6B | 255877 | 17 | 6863092 | 6871092 | 0.0005 | Tumor hypermethylation in CpG island core |
| SLC16A11 | 162515 | 17 | 6883966 | 6891966 | 0.0036 | Tumor hypermethylation in CpG island core |
| SOX15 | 6665 | 17 | 7430212 | 7438212 | 0.0005 | Tumor hypermethylation in CpG island core |
| TMEM88 | 92162 | 17 | 7695108 | 7703108 | 0 | Tumor hypermethylation in CpG island core |
| KCNAB3 | 9196 | 17 | 7769478 | 7777478 | 0.004 | Tumor hypermethylation in CpG island core |
| GUCY2D | 3000 | 17 | 7842712 | 7850712 | 0.0067 | Tumor hypermethylation in CpG island core |
| PGAP3 | 93210 | 17 | 7992478 | 8000478 | 0 | Tumor hypermethylation in CpG island core |
| GAS7 | 8522 | 17 | 10038593 | 10046593 | 0 | Tumor hypermethylation in CpG island core |
| TMEM220 | 388335 | 17 | 10570371 | 10578371 | 0.0016 | Tumor hypermethylation in CpG island core |
| DNAH9 | 1770 | 17 | 11438472 | 11446472 | 0.0062 | Tumor hypermethylation in CpG island core |
| HS3ST3A1 | 9955 | 17 | 13441969 | 13449969 | 0.0005 | Tumor hypermethylation in CpG island core |
| HS3ST3B1 | 9953 | 17 | 14141230 | 14149230 | 0 | Tumor hypermethylation in CpG island core |
| ADORA2B | 136 | 17 | 15784955 | 15792955 | 0.0016 | Tumor hypermethylation in CpG island core |
| PLD6 | 201164 | 17 | 17046371 | 17054371 | 0 | Tumor hypermethylation in CpG island core |
| KCNJ12 | 3768 | 17 | 21216291 | 21224291 | 0.0001 | Tumor hypermethylation in CpG island core |
| TMEM97 | 27346 | 17 | 23666247 | 23674247 | 0.0084 | Tumor hypermethylation in CpG island core |
| SARM1 | 23098 | 17 | 23719113 | 23727113 | 0.0001 | Tumor hypermethylation in CpG island core |
| PROCA1 | 147011 | 17 | 24058999 | 24066999 | 0.0007 | Tumor hypermethylation in CpG island core |
| RAB34 | 83871 | 17 | 24065035 | 24073035 | 0 | Tumor hypermethylation in CpG island core |
| SEZ6 | 124925 | 17 | 24353207 | 24361207 | 0.0017 | Tumor hypermethylation in CpG island core |
| ADAP2 | 55803 | 17 | 26268879 | 26276879 | 0.0039 | Tumor hypermethylation in CpG island core |
| RAB11FIP4 | 84440 | 17 | 26738767 | 26746767 | 0.0025 | Tumor hypermethylation in CpG island core |
| C17orf102 | 400591 | 17 | 29926501 | 29934501 | 0 | Tumor hypermethylation in CpG island core |
| TMEM132E | 124842 | 17 | 29927880 | 29935880 | 0 | Tumor hypermethylation in CpG island core |
| SLFN11 | 91607 | 17 | 30720833 | 30728833 | 0 | Tumor hypermethylation in CpG island core |
| SLFN13 | 146857 | 17 | 30795969 | 30803969 | 0.0022 | Tumor hypermethylation in CpG island core |
| HNF1B | 6928 | 17 | 33175209 | 33183209 | 0 | Tumor hypermethylation in CpG island core |
| C17orf98 | 388381 | 17 | 34247168 | 34255168 | 0 | Tumor hypermethylation in CpG island core |
| ARL5C | 390790 | 17 | 34571940 | 34579940 | 0.0025 | Tumor hypermethylation in CpG island core |
| STAC2 | 342667 | 17 | 34631500 | 34639500 | 0 | Tumor hypermethylation in CpG island core |
| PPP1R1B | 84152 | 17 | 35032704 | 35040704 | 0 | Tumor hypermethylation in CpG island core |
| ZPBP2 | 124626 | 17 | 35273980 | 35281980 | 0.0044 | Tumor hypermethylation in CpG island core |
| HAP1 | 9001 | 17 | 37140424 | 37148424 | 0.0022 | Tumor hypermethylation in CpG island core |
| HSPB9 | 94086 | 17 | 37524281 | 37532281 | 0.0014 | Tumor hypermethylation in CpG island core |
| KCNH4 | 23415 | 17 | 37582822 | 37590822 | 0.0001 | Tumor hypermethylation in CpG island core |
| MPP2 | 4355 | 17 | 39336639 | 39344639 | 0.0014 | Tumor hypermethylation in CpG island core |
| NAGS | 162417 | 17 | 39433557 | 39441557 | 0 | Tumor hypermethylation in CpG island core |
| TMEM101 | 84336 | 17 | 39443871 | 39451871 | 0.0016 | Tumor hypermethylation in CpG island core |
| SLC25A39 | 51629 | 17 | 39753743 | 39761743 | 0.0011 | Tumor hypermethylation in CpG island core |
| FZD2 | 2535 | 17 | 39986450 | 39994450 | 0.0073 | Tumor hypermethylation in CpG island core |
| C17orf104 | 284071 | 17 | 40085287 | 40093287 | 0 | Tumor hypermethylation in CpG island core |
| GJC1 | 125111 | 17 | 40259133 | 40267133 | 0 | Tumor hypermethylation in CpG island core |
| C1QL1 | 10882 | 17 | 40397170 | 40405170 | 0 | Tumor hypermethylation in CpG island core |
| ARHGAP27 | 201176 | 17 | 40835232 | 40843232 | 0.0087 | Tumor hypermethylation in CpG island core |
| CRHR1 | 1394 | 17 | 41213408 | 41221408 | 0.0024 | Tumor hypermethylation in CpG island core |
| WNT9B | 7484 | 17 | 42279966 | 42287966 | 0.0003 | Tumor hypermethylation in CpG island core |
| ITGB3 | 3690 | 17 | 42682206 | 42690206 | 0.0016 | Tumor hypermethylation in CpG island core |
| LOC100272146 | 1E+08 | 17 | 42851841 | 42859841 | 0.0048 | Tumor hypermethylation in CpG island core |
| TBX21 | 30009 | 17 | 43161608 | 43169608 | 0.0007 | Tumor hypermethylation in CpG island core |
| HOXB4 | 3214 | 17 | 44006742 | 44014742 | 0.0003 | Tumor hypermethylation in CpG island core |
| LOC404266 | 404266 | 17 | 44024318 | 44032318 | 0.0003 | Tumor hypermethylation in CpG island core |
| HOXB7 | 3217 | 17 | 44039382 | 44047382 | 0 | Tumor hypermethylation in CpG island core |
| HOXB13 | 10481 | 17 | 44157110 | 44165110 | 0 | Tumor hypermethylation in CpG island core |
| IGF2BP1 | 10642 | 17 | 44425772 | 44433772 | 0 | Tumor hypermethylation in CpG island core |
| DLX3 | 1747 | 17 | 45423587 | 45431587 | 0 | Tumor hypermethylation in CpG island core |
| CHAD | 1101 | 17 | 45897226 | 45905226 | 0.0004 | Tumor hypermethylation in CpG island core |
| MYCBPAP | 84073 | 17 | 45936743 | 45944743 | 0.0018 | Tumor hypermethylation in CpG island core |
| CACNA1G | 8913 | 17 | 45989447 | 45997447 | 0 | Tumor hypermethylation in CpG island core |
| CA10 | 56934 | 17 | 47587131 | 47595131 | 0 | Tumor hypermethylation in CpG island core |
| HLF | 3131 | 17 | 50693319 | 50701319 | 0 | Tumor hypermethylation in CpG island core |
| RNF126P1 | 376412 | 17 | 52473837 | 52481837 | 0 | Tumor hypermethylation in CpG island core |
| HSF5 | 124535 | 17 | 53916758 | 53924758 | 0 | Tumor hypermethylation in CpG island core |
| RAD51C | 5889 | 17 | 54120961 | 54128961 | 0.0088 | Tumor hypermethylation in CpG island core |
| CA4 | 762 | 17 | 55578083 | 55586083 | 0 | Tumor hypermethylation in CpG island core |
| C17orf64 | 124773 | 17 | 55850646 | 55858646 | 0 | Tumor hypermethylation in CpG island core |
| C17orf82 | 388407 | 17 | 56839893 | 56847893 | 0.0025 | Tumor hypermethylation in CpG island core |
| TBX4 | 9496 | 17 | 56884588 | 56892588 | 0 | Tumor hypermethylation in CpG island core |
| MRC2 | 9902 | 17 | 58054493 | 58062493 | 0.0028 | Tumor hypermethylation in CpG island core |
| ACE | 1636 | 17 | 58904165 | 58912165 | 0.0001 | Tumor hypermethylation in CpG island core |
| LIMD2 | 80774 | 17 | 59127251 | 59135251 | 0.0016 | Tumor hypermethylation in CpG island core |
| ERN1 | 2081 | 17 | 59557234 | 59565234 | 0.0063 | Tumor hypermethylation in CpG island core |
| CCDC46 | 201134 | 17 | 61614449 | 61622449 | 0.0008 | Tumor hypermethylation in CpG island core |
| LOC440461 | 440461 | 17 | 63702395 | 63710395 | 0 | Tumor hypermethylation in CpG island core |
| ARSG | 22901 | 17 | 63762917 | 63770917 | 0.0086 | Tumor hypermethylation in CpG island core |
| FAM20A | 54757 | 17 | 64104690 | 64112690 | 0.0006 | Tumor hypermethylation in CpG island core |
| KCNJ2 | 3759 | 17 | 65673270 | 65681270 | 0 | Tumor hypermethylation in CpG island core |
| SDK2 | 54549 | 17 | 69147822 | 69155822 | 0 | Tumor hypermethylation in CpG island core |
| MGC16275 | 85001 | 17 | 69717055 | 69725055 | 0 | Tumor hypermethylation in CpG island core |
| TTYH2 | 94015 | 17 | 69717290 | 69725290 | 0 | Tumor hypermethylation in CpG island core |
| RAB37 | 326624 | 17 | 70174850 | 70182850 | 0.0073 | Tumor hypermethylation in CpG island core |
| GRIN2C | 2905 | 17 | 70363602 | 70371602 | 0 | Tumor hypermethylation in CpG island core |
| FADS6 | 283985 | 17 | 70397300 | 70405300 | 0.0054 | Tumor hypermethylation in CpG island core |
| SLC16A5 | 9121 | 17 | 70591649 | 70599649 | 0.0001 | Tumor hypermethylation in CpG island core |
| MYO15B | 80022 | 17 | 71091733 | 71099733 | 0 | Tumor hypermethylation in CpG island core |
| QRICH2 | 84074 | 17 | 71811356 | 71819356 | 0 | Tumor hypermethylation in CpG island core |
| CYGB | 114757 | 17 | 72041377 | 72049377 | 0.0001 | Tumor hypermethylation in CpG island core |
| MGAT5B | 146664 | 17 | 72372392 | 72380392 | 0.0001 | Tumor hypermethylation in CpG island core |
| LOC283999 | 283999 | 17 | 73734985 | 73742985 | 0.0022 | Tumor hypermethylation in CpG island core |
| TIMP2 | 7077 | 17 | 74429067 | 74437067 | 0.0047 | Tumor hypermethylation in CpG island core |
| CBX2 | 84733 | 17 | 75362587 | 75370587 | 0.0076 | Tumor hypermethylation in CpG island core |
| NPTX1 | 4884 | 17 | 76060999 | 76068999 | 0 | Tumor hypermethylation in CpG island core |
| FSCN2 | 25794 | 17 | 77106011 | 77114011 | 0.0007 | Tumor hypermethylation in CpG island core |
| DYSFIP1 | 116729 | 17 | 77382215 | 77390215 | 0.005 | Tumor hypermethylation in CpG island core |
| MAFG | 4097 | 17 | 77470701 | 77478701 | 0.0014 | Tumor hypermethylation in CpG island core |
| NOTUM | 147111 | 17 | 77508347 | 77516347 | 0.0046 | Tumor hypermethylation in CpG island core |
| EMILIN2 | 84034 | 18 | 2833027 | 2841027 | 0.0001 | Tumor hypermethylation in CpG island core |
| DLGAP1 | 9229 | 18 | 3866135 | 3874135 | 0.0088 | Tumor hypermethylation in CpG island core |
| LOC642597 | 642597 | 18 | 5183255 | 5191255 | 0 | Tumor hypermethylation in CpG island core |
| LAMA1 | 284217 | 18 | 7103813 | 7111813 | 0 | Tumor hypermethylation in CpG island core |
| PTPRM | 5797 | 18 | 7553313 | 7561313 | 0 | Tumor hypermethylation in CpG island core |
| RAB31 | 11031 | 18 | 9694227 | 9702227 | 0.0046 | Tumor hypermethylation in CpG island core |
| APCDD1 | 147495 | 18 | 10440624 | 10448624 | 0.0025 | Tumor hypermethylation in CpG island core |
| FAM38B | 63895 | 18 | 11134761 | 11142761 | 0.0001 | Tumor hypermethylation in CpG island core |
| CIDEA | 1149 | 18 | 12240317 | 12248317 | 0.0051 | Tumor hypermethylation in CpG island core |
| TUBB6 | 84617 | 18 | 12294256 | 12302256 | 0.0012 | Tumor hypermethylation in CpG island core |
| CDH2 | 1000 | 18 | 24007443 | 24015443 | 0 | Tumor hypermethylation in CpG island core |
| DSC2 | 1825 | 18 | 26872779 | 26880779 | 0.0001 | Tumor hypermethylation in CpG island core |
| NOL4 | 8715 | 18 | 30053444 | 30061444 | 0 | Tumor hypermethylation in CpG island core |
| BRUNOL4 | 56853 | 18 | 33395998 | 33403998 | 0 | Tumor hypermethylation in CpG island core |
| ST8SIA5 | 29906 | 18 | 42587037 | 42595037 | 0 | Tumor hypermethylation in CpG island core |
| LIPG | 9388 | 18 | 45338424 | 45346424 | 0 | Tumor hypermethylation in CpG island core |
| TCF4 | 6934 | 18 | 51402858 | 51410858 | 0 | Tumor hypermethylation in CpG island core |
| ST8SIA3 | 51046 | 18 | 53166718 | 53174718 | 0 | Tumor hypermethylation in CpG island core |
| GRP | 29094 | 18 | 55034379 | 55042379 | 0 | Tumor hypermethylation in CpG island core |
| RAX | 30062 | 18 | 55087605 | 55095605 | 0 | Tumor hypermethylation in CpG island core |
| CCBE1 | 147372 | 18 | 55511624 | 55519624 | 0.0004 | Tumor hypermethylation in CpG island core |
| DOK6 | 220164 | 18 | 65215263 | 65223263 | 0 | Tumor hypermethylation in CpG island core |
| NETO1 | 81832 | 18 | 68681790 | 68689790 | 0 | Tumor hypermethylation in CpG island core |
| FAM69C | 125704 | 18 | 70271483 | 70279483 | 0.0064 | Tumor hypermethylation in CpG island core |
| GALR1 | 2587 | 18 | 73086995 | 73094995 | 0 | Tumor hypermethylation in CpG island core |
| LOC100130522 | 1E+08 | 18 | 76002797 | 76010797 | 0.0004 | Tumor hypermethylation in CpG island core |
| KIR2DS1 | 3806 | 19 | 253787 | 261787 | 0 | Tumor hypermethylation in CpG island core |
| MIER2 | 54531 | 19 | 291791 | 299791 | 0.0066 | Tumor hypermethylation in CpG island core |
| PALM | 5064 | 19 | 655952 | 663952 | 0.0088 | Tumor hypermethylation in CpG island core |
| ELANE | 1991 | 19 | 799290 | 807290 | 0 | Tumor hypermethylation in CpG island core |
| PLK5P | 126520 | 19 | 1471077 | 1479077 | 0.0006 | Tumor hypermethylation in CpG island core |
| S1PR4 | 8698 | 19 | 3125765 | 3133765 | 0 | Tumor hypermethylation in CpG island core |
| MATK | 4145 | 19 | 3733415 | 3741415 | 0.0026 | Tumor hypermethylation in CpG island core |
| SEMA6B | 10501 | 19 | 4505503 | 4513503 | 0.0029 | Tumor hypermethylation in CpG island core |
| ZNRF4 | 148066 | 19 | 5402425 | 5410425 | 0.0013 | Tumor hypermethylation in CpG island core |
| CAPS | 8618 | 19 | 5861192 | 5869192 | 0.0088 | Tumor hypermethylation in CpG island core |
| MIR220B | 1E+08 | 19 | 6442958 | 6450958 | 0.0002 | Tumor hypermethylation in CpG island core |
| TNFSF9 | 8744 | 19 | 6478009 | 6486009 | 0 | Tumor hypermethylation in CpG island core |
| CLEC4GP1 | 440508 | 19 | 7754369 | 7762369 | 0.009 | Tumor hypermethylation in CpG island core |
| CD320 | 51293 | 19 | 8275240 | 8283240 | 0.0014 | Tumor hypermethylation in CpG island core |
| KANK3 | 256949 | 19 | 8310146 | 8318146 | 0.0004 | Tumor hypermethylation in CpG island core |
| ANGPTL4 | 51129 | 19 | 8331010 | 8339010 | 0.003 | Tumor hypermethylation in CpG island core |
| ADAMTS10 | 81794 | 19 | 8577588 | 8585588 | 0.0001 | Tumor hypermethylation in CpG island core |
| ACTL9 | 284382 | 19 | 8666172 | 8674172 | 0.0064 | Tumor hypermethylation in CpG island core |
| ICAM4 | 3386 | 19 | 10254649 | 10262649 | 0.0002 | Tumor hypermethylation in CpG island core |
| ICAM5 | 7087 | 19 | 10257654 | 10265654 | 0 | Tumor hypermethylation in CpG island core |
| ZGLP1 | 1E+08 | 19 | 10277233 | 10285233 | 0.0053 | Tumor hypermethylation in CpG island core |
| RAVER1 | 125950 | 19 | 10301314 | 10309314 | 0.0007 | Tumor hypermethylation in CpG island core |
| KANK2 | 25959 | 19 | 11162266 | 11170266 | 0.0005 | Tumor hypermethylation in CpG island core |
| ELAVL3 | 1995 | 19 | 11448803 | 11456803 | 0 | Tumor hypermethylation in CpG island core |
| CACNA1A | 773 | 19 | 13474274 | 13482274 | 0.0002 | Tumor hypermethylation in CpG island core |
| CCDC105 | 126402 | 19 | 14978538 | 14986538 | 0.0062 | Tumor hypermethylation in CpG island core |
| EPHX3 | 79852 | 19 | 15200231 | 15208231 | 0 | Tumor hypermethylation in CpG island core |
| F2RL3 | 9002 | 19 | 16856825 | 16864825 | 0.0044 | Tumor hypermethylation in CpG island core |
| JAK3 | 3718 | 19 | 17815841 | 17823841 | 0.003 | Tumor hypermethylation in CpG island core |
| SLC5A5 | 6528 | 19 | 17839781 | 17847781 | 0 | Tumor hypermethylation in CpG island core |
| HAPLN4 | 404037 | 19 | 19230596 | 19238596 | 0.0022 | Tumor hypermethylation in CpG island core |
| TSSK6 | 83983 | 19 | 19483469 | 19491469 | 0.0001 | Tumor hypermethylation in CpG island core |
| YJEFN3 | 374887 | 19 | 19496719 | 19504719 | 0.0033 | Tumor hypermethylation in CpG island core |
| PBX4 | 80714 | 19 | 19586439 | 19594439 | 0.0023 | Tumor hypermethylation in CpG island core |
| TDRD12 | 91646 | 19 | 37898518 | 37906518 | 0.004 | Tumor hypermethylation in CpG island core |
| CHST8 | 64377 | 19 | 38800700 | 38808700 | 0.0069 | Tumor hypermethylation in CpG island core |
| LOC643719 | 643719 | 19 | 39756436 | 39764436 | 0.0055 | Tumor hypermethylation in CpG island core |
| FFAR1 | 2864 | 19 | 40530284 | 40538284 | 0.0044 | Tumor hypermethylation in CpG island core |
| HSPB6 | 126393 | 19 | 40935770 | 40943770 | 0 | Tumor hypermethylation in CpG island core |
| KIRREL2 | 84063 | 19 | 41035649 | 41043649 | 0.0056 | Tumor hypermethylation in CpG island core |
| SDHAF1 | 644096 | 19 | 41173940 | 41181940 | 0.0078 | Tumor hypermethylation in CpG island core |
| CLIP3 | 25999 | 19 | 41211615 | 41219615 | 0 | Tumor hypermethylation in CpG island core |
| COX7A1 | 1346 | 19 | 41331611 | 41339611 | 0 | Tumor hypermethylation in CpG island core |
| ZFP82 | 284406 | 19 | 41597390 | 41605390 | 0.0004 | Tumor hypermethylation in CpG island core |
| PPP1R14A | 94274 | 19 | 43435012 | 43443012 | 0.0007 | Tumor hypermethylation in CpG island core |
| FBXO17 | 115290 | 19 | 44154220 | 44162220 | 0.0011 | Tumor hypermethylation in CpG island core |
| FBXO27 | 126433 | 19 | 44211038 | 44219038 | 0.0033 | Tumor hypermethylation in CpG island core |
| SYCN | 342898 | 19 | 44382746 | 44390746 | 0.0032 | Tumor hypermethylation in CpG island core |
| LRFN1 | 57622 | 19 | 44493816 | 44501816 | 0.0001 | Tumor hypermethylation in CpG island core |
| CKM | 1158 | 19 | 50513974 | 50521974 | 0.0008 | Tumor hypermethylation in CpG island core |
| FLJ40125 | 147699 | 19 | 50689570 | 50697570 | 0.0001 | Tumor hypermethylation in CpG island core |
| RSPH6A | 81492 | 19 | 51006445 | 51014445 | 0.0004 | Tumor hypermethylation in CpG island core |
| PGLYRP1 | 8993 | 19 | 51214163 | 51222163 | 0.0045 | Tumor hypermethylation in CpG island core |
| HIF3A | 64344 | 19 | 51488144 | 51496144 | 0.0015 | Tumor hypermethylation in CpG island core |
| CCDC8 | 83987 | 19 | 51604759 | 51612759 | 0 | Tumor hypermethylation in CpG island core |
| PNMAL2 | 57469 | 19 | 51687009 | 51695009 | 0.0018 | Tumor hypermethylation in CpG island core |
| KCNJ14 | 3770 | 19 | 53652317 | 53660317 | 0.0015 | Tumor hypermethylation in CpG island core |
| FAM83E | 54854 | 19 | 53804506 | 53812506 | 0.001 | Tumor hypermethylation in CpG island core |
| MAMSTR | 284358 | 19 | 53910788 | 53918788 | 0.0007 | Tumor hypermethylation in CpG island core |
| RASIP1 | 54922 | 19 | 53931782 | 53939782 | 0.0066 | Tumor hypermethylation in CpG island core |
| KCNA7 | 3743 | 19 | 54264010 | 54272010 | 0.0045 | Tumor hypermethylation in CpG island core |
| CCDC155 | 147872 | 19 | 54579317 | 54587317 | 0.0095 | Tumor hypermethylation in CpG island core |
| PTH2 | 113091 | 19 | 54614510 | 54622510 | 0 | Tumor hypermethylation in CpG island core |
| FCGRT | 2217 | 19 | 54704247 | 54712247 | 0.0015 | Tumor hypermethylation in CpG island core |
| RCN3 | 57333 | 19 | 54718686 | 54726686 | 0 | Tumor hypermethylation in CpG island core |
| CPT1C | 126129 | 19 | 54882212 | 54890212 | 0 | Tumor hypermethylation in CpG island core |
| FLJ26850 | 400710 | 19 | 55241748 | 55249748 | 0.0039 | Tumor hypermethylation in CpG island core |
| C19orf41 | 126123 | 19 | 55354350 | 55362350 | 0.0086 | Tumor hypermethylation in CpG island core |
| KCNC3 | 3748 | 19 | 55520446 | 55528446 | 0 | Tumor hypermethylation in CpG island core |
| JOSD2 | 126119 | 19 | 55702157 | 55710157 | 0.008 | Tumor hypermethylation in CpG island core |
| CLEC11A | 6320 | 19 | 55914416 | 55922416 | 0.0004 | Tumor hypermethylation in CpG island core |
| KLK10 | 5655 | 19 | 56210766 | 56218766 | 0.0006 | Tumor hypermethylation in CpG island core |
| ZNF577 | 84765 | 19 | 57079001 | 57087001 | 0.0014 | Tumor hypermethylation in CpG island core |
| ZNF880 | 400713 | 19 | 57560981 | 57568981 | 0.0018 | Tumor hypermethylation in CpG island core |
| ZNF677 | 342926 | 19 | 58445923 | 58453923 | 0.0046 | Tumor hypermethylation in CpG island core |
| CACNG8 | 59283 | 19 | 59154105 | 59162105 | 0.002 | Tumor hypermethylation in CpG island core |
| CACNG6 | 59285 | 19 | 59183353 | 59191353 | 0.0013 | Tumor hypermethylation in CpG island core |
| TTYH1 | 57348 | 19 | 59614446 | 59622446 | 0.0016 | Tumor hypermethylation in CpG island core |
| TNNI3 | 7137 | 19 | 60356912 | 60364912 | 0.0013 | Tumor hypermethylation in CpG island core |
| SYT5 | 6861 | 19 | 60379532 | 60387532 | 0.0047 | Tumor hypermethylation in CpG island core |
| COX6B2 | 125965 | 19 | 60553994 | 60561994 | 0 | Tumor hypermethylation in CpG island core |
| ZNF667 | 63934 | 19 | 61676582 | 61684582 | 0 | Tumor hypermethylation in CpG island core |
| ZNF471 | 57573 | 19 | 61707023 | 61715023 | 0.0034 | Tumor hypermethylation in CpG island core |
| ZNF470 | 388566 | 19 | 61766701 | 61774701 | 0.0023 | Tumor hypermethylation in CpG island core |
| PEG3 | 5178 | 19 | 62039906 | 62047906 | 0 | Tumor hypermethylation in CpG island core |
| ZIM2 | 23619 | 19 | 62039909 | 62047909 | 0 | Tumor hypermethylation in CpG island core |
| USP29 | 57663 | 19 | 62319320 | 62327320 | 0.0079 | Tumor hypermethylation in CpG island core |
| ZNF264 | 9422 | 19 | 62390679 | 62398679 | 0.0092 | Tumor hypermethylation in CpG island core |
| ZIK1 | 284307 | 19 | 62783439 | 62791439 | 0.0001 | Tumor hypermethylation in CpG island core |
| ZNF154 | 7710 | 19 | 62908391 | 62916391 | 0 | Tumor hypermethylation in CpG island core |
| ZNF135 | 7694 | 19 | 63258418 | 63266418 | 0.0023 | Tumor hypermethylation in CpG island core |
| ZNF132 | 7691 | 19 | 63639401 | 63647401 | 0 | Tumor hypermethylation in CpG island core |
| FAM150B | 285016 | 2 | 274308 | 282308 | 0 | Tumor hypermethylation in CpG island core |
| SNTG2 | 54221 | 2 | 932553 | 940553 | 0.0007 | Tumor hypermethylation in CpG island core |
| PXN | 7837 | 2 | 1723298 | 1731298 | 0.002 | Tumor hypermethylation in CpG island core |
| SOX11 | 6664 | 2 | 5746249 | 5754249 | 0 | Tumor hypermethylation in CpG island core |
| KLF11 | 8462 | 2 | 10097132 | 10105132 | 0.0004 | Tumor hypermethylation in CpG island core |
| SLC25A21 | 89874 | 2 | 10501904 | 10509904 | 0.0013 | Tumor hypermethylation in CpG island core |
| VSNL1 | 7447 | 2 | 17581287 | 17589287 | 0 | Tumor hypermethylation in CpG island core |
| OSR1 | 130497 | 2 | 19417853 | 19425853 | 0 | Tumor hypermethylation in CpG island core |
| GDF7 | 151449 | 2 | 20725904 | 20733904 | 0.001 | Tumor hypermethylation in CpG island core |
| KLHL29 | 114818 | 2 | 23457802 | 23465802 | 0 | Tumor hypermethylation in CpG island core |
| MFSD2B | 388931 | 2 | 24082456 | 24090456 | 0.0017 | Tumor hypermethylation in CpG island core |
| C2orf84 | 653140 | 2 | 24247475 | 24255475 | 0.002 | Tumor hypermethylation in CpG island core |
| POMC | 5443 | 2 | 25241063 | 25249063 | 0.0085 | Tumor hypermethylation in CpG island core |
| FAM59B | 150946 | 2 | 26245463 | 26253463 | 0.0017 | Tumor hypermethylation in CpG island core |
| KCNK3 | 3777 | 2 | 26765084 | 26773084 | 0.0002 | Tumor hypermethylation in CpG island core |
| DPYSL5 | 56896 | 2 | 26920472 | 26928472 | 0 | Tumor hypermethylation in CpG island core |
| SLC30A3 | 7781 | 2 | 27335464 | 27343464 | 0.0003 | Tumor hypermethylation in CpG island core |
| UCN | 7349 | 2 | 27380634 | 27388634 | 0 | Tumor hypermethylation in CpG island core |
| KRTCAP3 | 200634 | 2 | 27514736 | 27522736 | 0.0042 | Tumor hypermethylation in CpG island core |
| CLIP4 | 79745 | 2 | 29187811 | 29195811 | 0.0018 | Tumor hypermethylation in CpG island core |
| ALK | 238 | 2 | 29993936 | 30001936 | 0 | Tumor hypermethylation in CpG island core |
| C6orf1 | 221491 | 2 | 30303900 | 30311900 | 0.0002 | Tumor hypermethylation in CpG island core |
| SRD5A2 | 6716 | 2 | 31655544 | 31663544 | 0.0094 | Tumor hypermethylation in CpG island core |
| CYP1B1 | 1545 | 2 | 38152827 | 38160827 | 0.0001 | Tumor hypermethylation in CpG island core |
| LOC375196 | 375196 | 2 | 39036989 | 39044989 | 0.0012 | Tumor hypermethylation in CpG island core |
| KCNG3 | 170850 | 2 | 42570741 | 42578741 | 0.005 | Tumor hypermethylation in CpG island core |
| HAAO | 23498 | 2 | 42869255 | 42877255 | 0.001 | Tumor hypermethylation in CpG island core |
| SIX3 | 6496 | 2 | 45018540 | 45026540 | 0.0002 | Tumor hypermethylation in CpG island core |
| KCNK12 | 56660 | 2 | 47646974 | 47654974 | 0 | Tumor hypermethylation in CpG island core |
| NRXN1 | 9378 | 2 | 50424398 | 50432398 | 0 | Tumor hypermethylation in CpG island core |
| LOC100302652 | 1E+08 | 2 | 53936674 | 53944674 | 0.0009 | Tumor hypermethylation in CpG island core |
| EFEMP1 | 2202 | 2 | 56000436 | 56008436 | 0.0006 | Tumor hypermethylation in CpG island core |
| LOC100132215 | 1E+08 | 2 | 63124350 | 63132350 | 0.0013 | Tumor hypermethylation in CpG island core |
| CRIP1 | 25927 | 2 | 68396687 | 68404687 | 0.0008 | Tumor hypermethylation in CpG island core |
| VAX2 | 25806 | 2 | 70977227 | 70985227 | 0 | Tumor hypermethylation in CpG island core |
| ANKRD53 | 79998 | 2 | 71055082 | 71063082 | 0.0032 | Tumor hypermethylation in CpG island core |
| DYSF | 8291 | 2 | 71530260 | 71538260 | 0.002 | Tumor hypermethylation in CpG island core |
| CYP26B1 | 56603 | 2 | 72224471 | 72232471 | 0.0015 | Tumor hypermethylation in CpG island core |
| EMX1 | 2016 | 2 | 72994111 | 73002111 | 0 | Tumor hypermethylation in CpG island core |
| NOTO | 344022 | 2 | 73278893 | 73286893 | 0 | Tumor hypermethylation in CpG island core |
| TLX2 | 3196 | 2 | 74591118 | 74599118 | 0 | Tumor hypermethylation in CpG island core |
| DOK1 | 1796 | 2 | 74631367 | 74639367 | 0.0001 | Tumor hypermethylation in CpG island core |
| LRRTM1 | 347730 | 2 | 80380998 | 80388998 | 0 | Tumor hypermethylation in CpG island core |
| C2orf89 | 129293 | 2 | 84957763 | 84965763 | 0.0004 | Tumor hypermethylation in CpG island core |
| VAMP5 | 10791 | 2 | 85661041 | 85669041 | 0.0039 | Tumor hypermethylation in CpG island core |
| FOXI3 | 344167 | 2 | 88529168 | 88537168 | 0.0001 | Tumor hypermethylation in CpG island core |
| FLJ40330 | 645784 | 2 | 88842533 | 88850533 | 0.0022 | Tumor hypermethylation in CpG island core |
| ADRA2B | 151 | 2 | 96141615 | 96149615 | 0.0033 | Tumor hypermethylation in CpG island core |
| ITPRIPL1 | 150771 | 2 | 96350788 | 96358788 | 0.0011 | Tumor hypermethylation in CpG island core |
| FAM178B | 51252 | 2 | 97012028 | 97020028 | 0.0056 | Tumor hypermethylation in CpG island core |
| ZAP70 | 7535 | 2 | 97713300 | 97721300 | 0.0001 | Tumor hypermethylation in CpG island core |
| CNGA3 | 1261 | 2 | 98325049 | 98333049 | 0 | Tumor hypermethylation in CpG island core |
| IL1RL2 | 8808 | 2 | 102165864 | 102173864 | 0.0063 | Tumor hypermethylation in CpG island core |
| ST6GAL2 | 84620 | 2 | 106865042 | 106873042 | 0 | Tumor hypermethylation in CpG island core |
| SLC5A7 | 60482 | 2 | 107965426 | 107973426 | 0.0061 | Tumor hypermethylation in CpG island core |
| LOC100287216 | 1E+08 | 2 | 109109007 | 109117007 | 0.0006 | Tumor hypermethylation in CpG island core |
| 10-Sep | 151011 | 2 | 109725072 | 109733072 | 0.0017 | Tumor hypermethylation in CpG island core |
| MERTK | 10461 | 2 | 112368661 | 112376661 | 0.0011 | Tumor hypermethylation in CpG island core |
| LOC654433 | 654433 | 2 | 113706316 | 113714316 | 0.0018 | Tumor hypermethylation in CpG island core |
| DPP10 | 57628 | 2 | 115632201 | 115640201 | 0 | Tumor hypermethylation in CpG island core |
| EN1 | 2019 | 2 | 119318229 | 119326229 | 0.0005 | Tumor hypermethylation in CpG island core |
| CNTNAP5 | 129684 | 2 | 124495333 | 124503333 | 0.0001 | Tumor hypermethylation in CpG island core |
| GYPC | 2995 | 2 | 127126153 | 127134153 | 0.0002 | Tumor hypermethylation in CpG island core |
| LOC401010 | 401010 | 2 | 131914937 | 131922937 | 0.0001 | Tumor hypermethylation in CpG island core |
| NXPH2 | 11249 | 2 | 139250281 | 139258281 | 0.0012 | Tumor hypermethylation in CpG island core |
| RPRM | 56475 | 2 | 154039568 | 154047568 | 0.0001 | Tumor hypermethylation in CpG island core |
| GALNT7 | 117248 | 2 | 154432671 | 154440671 | 0 | Tumor hypermethylation in CpG island core |
| KCNJ3 | 3760 | 2 | 155259338 | 155267338 | 0.0055 | Tumor hypermethylation in CpG island core |
| LY75 | 4065 | 2 | 160465508 | 160473508 | 0.0046 | Tumor hypermethylation in CpG island core |
| TBR1 | 10716 | 2 | 161976865 | 161984865 | 0.0018 | Tumor hypermethylation in CpG island core |
| DLX1 | 1745 | 2 | 172654453 | 172662453 | 0 | Tumor hypermethylation in CpG island core |
| SP9 | 1E+08 | 2 | 174904066 | 174912066 | 0 | Tumor hypermethylation in CpG island core |
| WIPF1 | 7456 | 2 | 175251873 | 175259873 | 0.0004 | Tumor hypermethylation in CpG island core |
| HOXD13 | 3239 | 2 | 176661777 | 176669777 | 0 | Tumor hypermethylation in CpG island core |
| HOXD12 | 3238 | 2 | 176668775 | 176676775 | 0 | Tumor hypermethylation in CpG island core |
| HOXD11 | 3237 | 2 | 176676329 | 176684329 | 0.0002 | Tumor hypermethylation in CpG island core |
| HOXD10 | 3236 | 2 | 176685737 | 176693737 | 0 | Tumor hypermethylation in CpG island core |
| HOXD9 | 3235 | 2 | 176691658 | 176699658 | 0 | Tumor hypermethylation in CpG island core |
| HOXD8 | 3234 | 2 | 176698722 | 176706722 | 0 | Tumor hypermethylation in CpG island core |
| HOXD3 | 3232 | 2 | 176733050 | 176741050 | 0.0011 | Tumor hypermethylation in CpG island core |
| HOXD1 | 3231 | 2 | 176757552 | 176765552 | 0.0013 | Tumor hypermethylation in CpG island core |
| PRKRA | 8575 | 2 | 179019730 | 179027730 | 0.0041 | Tumor hypermethylation in CpG island core |
| ZNF385B | 151126 | 2 | 180430477 | 180438477 | 0.0001 | Tumor hypermethylation in CpG island core |
| ITGA4 | 3676 | 2 | 182025863 | 182033863 | 0 | Tumor hypermethylation in CpG island core |
| FRZB | 2487 | 2 | 183435743 | 183443743 | 0.0045 | Tumor hypermethylation in CpG island core |
| ZNF804A | 91752 | 2 | 185167337 | 185175337 | 0.0011 | Tumor hypermethylation in CpG island core |
| C2orf88 | 84281 | 2 | 190749833 | 190757833 | 0 | Tumor hypermethylation in CpG island core |
| TMEFF2 | 23671 | 2 | 192763889 | 192771889 | 0 | Tumor hypermethylation in CpG island core |
| BOLL | 66037 | 2 | 198354319 | 198362319 | 0.0004 | Tumor hypermethylation in CpG island core |
| ZDBF2 | 57683 | 2 | 206843767 | 206851767 | 0 | Tumor hypermethylation in CpG island core |
| ADAM28 | 10863 | 2 | 207012612 | 207020612 | 0.0004 | Tumor hypermethylation in CpG island core |
| LOC200726 | 200726 | 2 | 207211386 | 207219386 | 0.002 | Tumor hypermethylation in CpG island core |
| PTH2R | 5746 | 2 | 208975800 | 208983800 | 0.0004 | Tumor hypermethylation in CpG island core |
| WNT10A | 80326 | 2 | 219449498 | 219457498 | 0.0064 | Tumor hypermethylation in CpG island core |
| IHH | 3549 | 2 | 219629433 | 219637433 | 0 | Tumor hypermethylation in CpG island core |
| PTPRN | 5798 | 2 | 219878387 | 219886387 | 0.0032 | Tumor hypermethylation in CpG island core |
| RESP18 | 389075 | 2 | 219902143 | 219910143 | 0.0033 | Tumor hypermethylation in CpG island core |
| DES | 1674 | 2 | 219987342 | 219995342 | 0.0019 | Tumor hypermethylation in CpG island core |
| SPEG | 10290 | 2 | 220003943 | 220011943 | 0.0092 | Tumor hypermethylation in CpG island core |
| EPHA4 | 2043 | 2 | 222141254 | 222149254 | 0.0033 | Tumor hypermethylation in CpG island core |
| SPHKAP | 80309 | 2 | 228750605 | 228758605 | 0.0001 | Tumor hypermethylation in CpG island core |
| DNER | 92737 | 2 | 230283530 | 230291530 | 0 | Tumor hypermethylation in CpG island core |
| ECEL1P2 | 347694 | 2 | 232955998 | 232963998 | 0 | Tumor hypermethylation in CpG island core |
| ECEL1 | 9427 | 2 | 233056776 | 233064776 | 0.0001 | Tumor hypermethylation in CpG island core |
| NGEF | 25791 | 2 | 233497105 | 233505105 | 0.0001 | Tumor hypermethylation in CpG island core |
| RBM44 | 375316 | 2 | 238368126 | 238376126 | 0.0007 | Tumor hypermethylation in CpG island core |
| KIF1A | 547 | 2 | 241404297 | 241412297 | 0.0028 | Tumor hypermethylation in CpG island core |
| TCF15 | 6939 | 20 | 534910 | 542910 | 0.0003 | Tumor hypermethylation in CpG island core |
| RSPO4 | 343637 | 20 | 926907 | 934907 | 0.0022 | Tumor hypermethylation in CpG island core |
| SIRPA | 140885 | 20 | 1819424 | 1827424 | 0.0014 | Tumor hypermethylation in CpG island core |
| EBF4 | 57593 | 20 | 2617523 | 2625523 | 0.0001 | Tumor hypermethylation in CpG island core |
| CPXM1 | 56265 | 20 | 2725282 | 2733282 | 0 | Tumor hypermethylation in CpG island core |
| OXT | 5020 | 20 | 2996265 | 3004265 | 0.0004 | Tumor hypermethylation in CpG island core |
| SLC4A11 | 83959 | 20 | 3162373 | 3170373 | 0.0096 | Tumor hypermethylation in CpG island core |
| ADAM33 | 80332 | 20 | 3606738 | 3614738 | 0.0064 | Tumor hypermethylation in CpG island core |
| HSPA12B | 116835 | 20 | 3657355 | 3665355 | 0.0041 | Tumor hypermethylation in CpG island core |
| CENPB | 1059 | 20 | 3711337 | 3719337 | 0.0075 | Tumor hypermethylation in CpG island core |
| ADRA1D | 146 | 20 | 4173659 | 4181659 | 0.0001 | Tumor hypermethylation in CpG island core |
| RASSF2 | 9770 | 20 | 4748291 | 4756291 | 0 | Tumor hypermethylation in CpG island core |
| PLCB1 | 23236 | 20 | 8057295 | 8065295 | 0.002 | Tumor hypermethylation in CpG island core |
| C20orf103 | 24141 | 20 | 9439270 | 9447270 | 0 | Tumor hypermethylation in CpG island core |
| SNAP25 | 6616 | 20 | 10143476 | 10151476 | 0 | Tumor hypermethylation in CpG island core |
| PCSK2 | 5126 | 20 | 17151630 | 17159630 | 0 | Tumor hypermethylation in CpG island core |
| NKX2-4 | 644524 | 20 | 21322047 | 21330047 | 0 | Tumor hypermethylation in CpG island core |
| C20orf56 | 140828 | 20 | 22503280 | 22511280 | 0 | Tumor hypermethylation in CpG island core |
| SSTR4 | 6754 | 20 | 22960056 | 22968056 | 0.0072 | Tumor hypermethylation in CpG island core |
| THBD | 7056 | 20 | 22974301 | 22982301 | 0 | Tumor hypermethylation in CpG island core |
| TMEM90B | 79953 | 20 | 24393834 | 24401834 | 0.0001 | Tumor hypermethylation in CpG island core |
| VSX1 | 30813 | 20 | 25006767 | 25014767 | 0 | Tumor hypermethylation in CpG island core |
| LOC284798 | 284798 | 20 | 25073426 | 25081426 | 0.0037 | Tumor hypermethylation in CpG island core |
| REM1 | 28954 | 20 | 29522765 | 29530765 | 0.0006 | Tumor hypermethylation in CpG island core |
| HCK | 3055 | 20 | 30099717 | 30107717 | 0 | Tumor hypermethylation in CpG island core |
| C20orf134 | 170487 | 20 | 31713964 | 31721964 | 0.0089 | Tumor hypermethylation in CpG island core |
| MAP1LC3A | 84557 | 20 | 32606179 | 32614179 | 0.0015 | Tumor hypermethylation in CpG island core |
| MYL9 | 10398 | 20 | 34599310 | 34607310 | 0.0048 | Tumor hypermethylation in CpG island core |
| NNAT | 4826 | 20 | 35579020 | 35587020 | 0 | Tumor hypermethylation in CpG island core |
| C20orf95 | 343578 | 20 | 36659990 | 36667990 | 0.0013 | Tumor hypermethylation in CpG island core |
| SLC32A1 | 140679 | 20 | 36782518 | 36790518 | 0 | Tumor hypermethylation in CpG island core |
| PPP1R16B | 26051 | 20 | 36863761 | 36871761 | 0.0002 | Tumor hypermethylation in CpG island core |
| PTPRT | 11122 | 20 | 41247971 | 41255971 | 0 | Tumor hypermethylation in CpG island core |
| L3MBTL | 26013 | 20 | 41572466 | 41580466 | 0.0026 | Tumor hypermethylation in CpG island core |
| TOX2 | 84969 | 20 | 41972905 | 41980905 | 0 | Tumor hypermethylation in CpG island core |
| GDAP1L1 | 78997 | 20 | 42305321 | 42313321 | 0.0001 | Tumor hypermethylation in CpG island core |
| RBPJL | 11317 | 20 | 43364904 | 43372904 | 0.0034 | Tumor hypermethylation in CpG island core |
| PLTP | 5360 | 20 | 43970193 | 43978193 | 0 | Tumor hypermethylation in CpG island core |
| CD40 | 958 | 20 | 44176312 | 44184312 | 0.0004 | Tumor hypermethylation in CpG island core |
| ZNF334 | 55713 | 20 | 44571601 | 44579601 | 0.0028 | Tumor hypermethylation in CpG island core |
| KCNG1 | 3755 | 20 | 49069082 | 49077082 | 0.0006 | Tumor hypermethylation in CpG island core |
| TSHZ2 | 128553 | 20 | 51018283 | 51026283 | 0.0001 | Tumor hypermethylation in CpG island core |
| CYP24A1 | 1591 | 20 | 52219923 | 52227923 | 0 | Tumor hypermethylation in CpG island core |
| DOK5 | 55816 | 20 | 52521672 | 52529672 | 0 | Tumor hypermethylation in CpG island core |
| CBLN4 | 140689 | 20 | 54009419 | 54017419 | 0 | Tumor hypermethylation in CpG island core |
| BMP7 | 655 | 20 | 55271114 | 55279114 | 0.0023 | Tumor hypermethylation in CpG island core |
| RAB22A | 57403 | 20 | 56314176 | 56322176 | 0.0024 | Tumor hypermethylation in CpG island core |
| GNAS | 2778 | 20 | 56844189 | 56852189 | 0.0001 | Tumor hypermethylation in CpG island core |
| ZNF831 | 128611 | 20 | 57195469 | 57203469 | 0.0051 | Tumor hypermethylation in CpG island core |
| EDN3 | 1908 | 20 | 57304893 | 57312893 | 0.007 | Tumor hypermethylation in CpG island core |
| PHACTR3 | 116154 | 20 | 57608997 | 57616997 | 0.0002 | Tumor hypermethylation in CpG island core |
| CDH4 | 1002 | 20 | 59256953 | 59264953 | 0 | Tumor hypermethylation in CpG island core |
| GATA5 | 140628 | 20 | 60480421 | 60488421 | 0 | Tumor hypermethylation in CpG island core |
| NTSR1 | 4923 | 20 | 60806633 | 60814633 | 0.0056 | Tumor hypermethylation in CpG island core |
| COL9A3 | 1299 | 20 | 60914858 | 60922858 | 0.0003 | Tumor hypermethylation in CpG island core |
| SLC17A9 | 63910 | 20 | 61050443 | 61058443 | 0.0055 | Tumor hypermethylation in CpG island core |
| BHLHE23 | 128408 | 20 | 61104832 | 61112832 | 0 | Tumor hypermethylation in CpG island core |
| HAR1B | 768097 | 20 | 61200116 | 61208116 | 0 | Tumor hypermethylation in CpG island core |
| MIR124-3 | 406909 | 20 | 61276296 | 61284296 | 0 | Tumor hypermethylation in CpG island core |
| NKAIN4 | 128414 | 20 | 61352337 | 61360337 | 0 | Tumor hypermethylation in CpG island core |
| CHRNA4 | 1137 | 20 | 61459139 | 61467139 | 0 | Tumor hypermethylation in CpG island core |
| KCNQ2 | 3785 | 20 | 61570437 | 61578437 | 0.0001 | Tumor hypermethylation in CpG island core |
| SRMS | 6725 | 20 | 61645301 | 61653301 | 0.0007 | Tumor hypermethylation in CpG island core |
| C20orf135 | 140701 | 20 | 61959009 | 61967009 | 0.0002 | Tumor hypermethylation in CpG island core |
| MIR941-1 | 1E+08 | 20 | 62017237 | 62025237 | 0.0019 | Tumor hypermethylation in CpG island core |
| SOX18 | 54345 | 20 | 62147423 | 62155423 | 0.0005 | Tumor hypermethylation in CpG island core |
| TCEA2 | 6919 | 20 | 62154882 | 62162882 | 0.0062 | Tumor hypermethylation in CpG island core |
| NPBWR2 | 2832 | 20 | 62204628 | 62212628 | 0.0025 | Tumor hypermethylation in CpG island core |
| C21orf81 | 391267 | 21 | 14270636 | 14278636 | 0.0001 | Tumor hypermethylation in CpG island core |
| BTG3 | 10950 | 21 | 17903139 | 17911139 | 0.0028 | Tumor hypermethylation in CpG island core |
| CHODL | 140578 | 21 | 18535020 | 18543020 | 0.0004 | Tumor hypermethylation in CpG island core |
| NCAM2 | 4685 | 21 | 21288503 | 21296503 | 0.0034 | Tumor hypermethylation in CpG island core |
| CYYR1 | 116159 | 21 | 26863452 | 26871452 | 0.0031 | Tumor hypermethylation in CpG island core |
| ADAMTS1 | 9510 | 21 | 27135599 | 27143599 | 0 | Tumor hypermethylation in CpG island core |
| ADAMTS5 | 11096 | 21 | 27257310 | 27265310 | 0 | Tumor hypermethylation in CpG island core |
| GRIK1 | 2897 | 21 | 30230153 | 30238153 | 0.002 | Tumor hypermethylation in CpG island core |
| TIAM1 | 7074 | 21 | 31849161 | 31857161 | 0 | Tumor hypermethylation in CpG island core |
| OLIG1 | 116448 | 21 | 33360319 | 33368319 | 0 | Tumor hypermethylation in CpG island core |
| IFNGR2 | 3460 | 21 | 33693071 | 33701071 | 0.0091 | Tumor hypermethylation in CpG island core |
| CLIC6 | 54102 | 21 | 34959557 | 34967557 | 0.0006 | Tumor hypermethylation in CpG island core |
| CBR1 | 873 | 21 | 36360154 | 36368154 | 0.0004 | Tumor hypermethylation in CpG island core |
| KCNJ6 | 3763 | 21 | 38206566 | 38214566 | 0 | Tumor hypermethylation in CpG island core |
| ERG | 2078 | 21 | 38951574 | 38959574 | 0 | Tumor hypermethylation in CpG island core |
| CBS | 875 | 21 | 43365109 | 43373109 | 0.0008 | Tumor hypermethylation in CpG island core |
| HSF2BP | 11077 | 21 | 43899802 | 43907802 | 0.0099 | Tumor hypermethylation in CpG island core |
| SUMO3 | 6612 | 21 | 45058472 | 45066472 | 0.0014 | Tumor hypermethylation in CpG island core |
| COL18A1 | 80781 | 21 | 45645524 | 45653524 | 0.003 | Tumor hypermethylation in CpG island core |
| COL6A2 | 1292 | 21 | 46338460 | 46346460 | 0.0064 | Tumor hypermethylation in CpG island core |
| GSC2 | 2928 | 22 | 17513796 | 17521796 | 0 | Tumor hypermethylation in CpG island core |
| LOC150185 | 150185 | 22 | 17930362 | 17938362 | 0.0088 | Tumor hypermethylation in CpG island core |
| TBX1 | 6899 | 22 | 18120225 | 18128225 | 0.0011 | Tumor hypermethylation in CpG island core |
| SCARF2 | 91179 | 22 | 19118146 | 19126146 | 0.0004 | Tumor hypermethylation in CpG island core |
| MGC16703 | 113691 | 22 | 19694576 | 19702576 | 0.0081 | Tumor hypermethylation in CpG island core |
| SLC7A4 | 6545 | 22 | 19712847 | 19720847 | 0.008 | Tumor hypermethylation in CpG island core |
| ZNF280B | 140883 | 22 | 21189505 | 21197505 | 0.0015 | Tumor hypermethylation in CpG island core |
| DERL3 | 91319 | 22 | 22507199 | 22515199 | 0.001 | Tumor hypermethylation in CpG island core |
| C22orf45 | 646023 | 22 | 23216783 | 23224783 | 0.0037 | Tumor hypermethylation in CpG island core |
| TTC28 | 23331 | 22 | 27401853 | 27409853 | 0.002 | Tumor hypermethylation in CpG island core |
| KREMEN1 | 83999 | 22 | 27795065 | 27803065 | 0.0006 | Tumor hypermethylation in CpG island core |
| NEFH | 4744 | 22 | 28202180 | 28210180 | 0 | Tumor hypermethylation in CpG island core |
| ELFN2 | 114794 | 22 | 36149451 | 36157451 | 0.0043 | Tumor hypermethylation in CpG island core |
| SOX10 | 6663 | 22 | 36706485 | 36714485 | 0.0022 | Tumor hypermethylation in CpG island core |
| NHP2L1 | 4809 | 22 | 40404502 | 40412502 | 0.0048 | Tumor hypermethylation in CpG island core |
| MEI1 | 150365 | 22 | 40421463 | 40429463 | 0.0018 | Tumor hypermethylation in CpG island core |
| SERHL | 94009 | 22 | 41222528 | 41230528 | 0.0058 | Tumor hypermethylation in CpG island core |
| SCUBE1 | 80274 | 22 | 42065299 | 42073299 | 0.0055 | Tumor hypermethylation in CpG island core |
| GRAMD4 | 23151 | 22 | 45397321 | 45405321 | 0 | Tumor hypermethylation in CpG island core |
| MOV10L1 | 54456 | 22 | 48866561 | 48874561 | 0.002 | Tumor hypermethylation in CpG island core |
| TRABD | 80305 | 22 | 48962486 | 48970486 | 0.0005 | Tumor hypermethylation in CpG island core |
| ADM2 | 79924 | 22 | 49262877 | 49270877 | 0.0019 | Tumor hypermethylation in CpG island core |
| KLHDC7B | 113730 | 22 | 49329327 | 49337327 | 0 | Tumor hypermethylation in CpG island core |
| CPT1B | 1375 | 22 | 49359744 | 49367744 | 0.0063 | Tumor hypermethylation in CpG island core |
| SHANK3 | 85358 | 22 | 49455935 | 49463935 | 0.0009 | Tumor hypermethylation in CpG island core |
| CHL1 | 10752 | 3 | 209649 | 217649 | 0 | Tumor hypermethylation in CpG island core |
| LRRN1 | 57633 | 3 | 3812120 | 3820120 | 0.0004 | Tumor hypermethylation in CpG island core |
| GRM7 | 2917 | 3 | 6873801 | 6881801 | 0 | Tumor hypermethylation in CpG island core |
| LHFPL3 | 375612 | 3 | 9566486 | 9574486 | 0.0004 | Tumor hypermethylation in CpG island core |
| CPNE9 | 151835 | 3 | 9716509 | 9724509 | 0.0008 | Tumor hypermethylation in CpG island core |
| IRAK2 | 3656 | 3 | 10177562 | 10185562 | 0.0009 | Tumor hypermethylation in CpG island core |
| SLC6A1 | 6529 | 3 | 11005419 | 11013419 | 0 | Tumor hypermethylation in CpG island core |
| SYN2 | 6854 | 3 | 12016861 | 12024861 | 0.0002 | Tumor hypermethylation in CpG island core |
| WNT7A | 7476 | 3 | 13892619 | 13900619 | 0 | Tumor hypermethylation in CpG island core |
| DAZL | 1618 | 3 | 16618010 | 16626010 | 0.0071 | Tumor hypermethylation in CpG island core |
| ELK1 | 131096 | 3 | 19161020 | 19169020 | 0.0001 | Tumor hypermethylation in CpG island core |
| LRRC3B | 116135 | 3 | 26635303 | 26643303 | 0.0095 | Tumor hypermethylation in CpG island core |
| EOMES | 8320 | 3 | 27734789 | 27742789 | 0 | Tumor hypermethylation in CpG island core |
| TRIM71 | 131405 | 3 | 32830513 | 32838513 | 0 | Tumor hypermethylation in CpG island core |
| ITGA9 | 3680 | 3 | 37464816 | 37472816 | 0.0019 | Tumor hypermethylation in CpG island core |
| DLEC1 | 9940 | 3 | 38051699 | 38059699 | 0.0006 | Tumor hypermethylation in CpG island core |
| RIPK2 | 8767 | 3 | 42277399 | 42285399 | 0 | Tumor hypermethylation in CpG island core |
| VIPR1 | 7433 | 3 | 42515107 | 42523107 | 0.0015 | Tumor hypermethylation in CpG island core |
| KBTBD5 | 131377 | 3 | 42698014 | 42706014 | 0.0061 | Tumor hypermethylation in CpG island core |
| ZNF662 | 389114 | 3 | 42918661 | 42926661 | 0.0005 | Tumor hypermethylation in CpG island core |
| ZNF660 | 285349 | 3 | 44597459 | 44605459 | 0.0009 | Tumor hypermethylation in CpG island core |
| SIT1 | 54716 | 3 | 45809039 | 45817039 | 0.0015 | Tumor hypermethylation in CpG island core |
| CELSR3 | 1951 | 3 | 48671352 | 48679352 | 0 | Tumor hypermethylation in CpG island core |
| CCDC36 | 339834 | 3 | 49207936 | 49215936 | 0.0002 | Tumor hypermethylation in CpG island core |
| GPX1 | 2876 | 3 | 49366795 | 49374795 | 0.0002 | Tumor hypermethylation in CpG island core |
| HYAL2 | 8692 | 3 | 50329974 | 50337974 | 0.0049 | Tumor hypermethylation in CpG island core |
| C3orf18 | 51161 | 3 | 50576227 | 50584227 | 0.0001 | Tumor hypermethylation in CpG island core |
| GPR62 | 118442 | 3 | 51960369 | 51968369 | 0.0048 | Tumor hypermethylation in CpG island core |
| CAPS | 8618 | 3 | 62832104 | 62840104 | 0 | Tumor hypermethylation in CpG island core |
| SYNPR | 132204 | 3 | 63234953 | 63242953 | 0.004 | Tumor hypermethylation in CpG island core |
| FAM19A4 | 151647 | 3 | 69060401 | 69068401 | 0 | Tumor hypermethylation in CpG island core |
| PDZRN3 | 23024 | 3 | 73752762 | 73760762 | 0 | Tumor hypermethylation in CpG island core |
| FILIP1L | 11259 | 3 | 101073736 | 101081736 | 0.0007 | Tumor hypermethylation in CpG island core |
| CD200 | 4345 | 3 | 113530605 | 113538605 | 0.0019 | Tumor hypermethylation in CpG island core |
| FSTL1 | 11167 | 3 | 121648608 | 121656608 | 0.0008 | Tumor hypermethylation in CpG island core |
| CASR | 846 | 3 | 123381219 | 123389219 | 0.0004 | Tumor hypermethylation in CpG island core |
| PARP15 | 165631 | 3 | 123775138 | 123783138 | 0.0099 | Tumor hypermethylation in CpG island core |
| ALDH1L1 | 10840 | 3 | 127378175 | 127386175 | 0 | Tumor hypermethylation in CpG island core |
| PLXNA1 | 5361 | 3 | 128186126 | 128194126 | 0.0047 | Tumor hypermethylation in CpG island core |
| PODXL2 | 50512 | 3 | 128826728 | 128834728 | 0.0097 | Tumor hypermethylation in CpG island core |
| GATA2 | 2624 | 3 | 129686063 | 129694063 | 0 | Tumor hypermethylation in CpG island core |
| CCDC48 | 79825 | 3 | 130199161 | 130207161 | 0.0002 | Tumor hypermethylation in CpG island core |
| TRH | 7200 | 3 | 131171803 | 131179803 | 0 | Tumor hypermethylation in CpG island core |
| COL29A1 | 256076 | 3 | 131543048 | 131551048 | 0.0083 | Tumor hypermethylation in CpG island core |
| NUDT16P | 152195 | 3 | 132559378 | 132567378 | 0.0024 | Tumor hypermethylation in CpG island core |
| TF | 7018 | 3 | 134943666 | 134951666 | 0.0058 | Tumor hypermethylation in CpG island core |
| EPHB1 | 2047 | 3 | 135992949 | 136000949 | 0 | Tumor hypermethylation in CpG island core |
| TMEM22 | 80723 | 3 | 138016550 | 138024550 | 0 | Tumor hypermethylation in CpG island core |
| ESYT3 | 83850 | 3 | 139632104 | 139640104 | 0.0002 | Tumor hypermethylation in CpG island core |
| PRR23A | 729627 | 3 | 140203800 | 140211800 | 0.0006 | Tumor hypermethylation in CpG island core |
| PRR23B | 389151 | 3 | 140218458 | 140226458 | 0 | Tumor hypermethylation in CpG island core |
| PRR23C | 389152 | 3 | 140242424 | 140250424 | 0 | Tumor hypermethylation in CpG island core |
| SPSB4 | 92369 | 3 | 142249432 | 142257432 | 0 | Tumor hypermethylation in CpG island core |
| PAQR9 | 344838 | 3 | 144160868 | 144168868 | 0.0005 | Tumor hypermethylation in CpG island core |
| CHST2 | 9435 | 3 | 144317357 | 144325357 | 0 | Tumor hypermethylation in CpG island core |
| ZIC4 | 84107 | 3 | 148588874 | 148596874 | 0 | Tumor hypermethylation in CpG island core |
| ZIC1 | 7545 | 3 | 148605870 | 148613870 | 0 | Tumor hypermethylation in CpG island core |
| P2RY1 | 5028 | 3 | 154031425 | 154039425 | 0.0026 | Tumor hypermethylation in CpG island core |
| GPR149 | 344758 | 3 | 155626198 | 155634198 | 0.0036 | Tumor hypermethylation in CpG island core |
| KCNAB1 | 7881 | 3 | 157487469 | 157495469 | 0.0009 | Tumor hypermethylation in CpG island core |
| SHOX2 | 6474 | 3 | 159302646 | 159310646 | 0 | Tumor hypermethylation in CpG island core |
| C3orf50 | 93556 | 3 | 169446003 | 169454003 | 0 | Tumor hypermethylation in CpG island core |
| LRRC34 | 151827 | 3 | 171009006 | 171017006 | 0 | Tumor hypermethylation in CpG island core |
| CLDN11 | 5010 | 3 | 171615346 | 171623346 | 0 | Tumor hypermethylation in CpG island core |
| SLC7A14 | 57709 | 3 | 171782557 | 171790557 | 0.0002 | Tumor hypermethylation in CpG island core |
| EIF5A2 | 56648 | 3 | 172105120 | 172113120 | 0.0041 | Tumor hypermethylation in CpG island core |
| GHSR | 2693 | 3 | 173644897 | 173652897 | 0 | Tumor hypermethylation in CpG island core |
| GNB4 | 59345 | 3 | 180648065 | 180656065 | 0 | Tumor hypermethylation in CpG island core |
| PEX5L | 51555 | 3 | 181233211 | 181241211 | 0.0016 | Tumor hypermethylation in CpG island core |
| SOX2 | 6657 | 3 | 182908415 | 182916415 | 0 | Tumor hypermethylation in CpG island core |
| B3GNT5 | 84002 | 3 | 184449725 | 184457725 | 0 | Tumor hypermethylation in CpG island core |
| MCF2L2 | 23101 | 3 | 184624549 | 184632549 | 0.0044 | Tumor hypermethylation in CpG island core |
| MIR1224 | 1E+08 | 3 | 185437886 | 185445886 | 0.0043 | Tumor hypermethylation in CpG island core |
| C3orf70 | 285382 | 3 | 186349496 | 186357496 | 0.0056 | Tumor hypermethylation in CpG island core |
| IGF2BP2 | 10644 | 3 | 187021521 | 187029521 | 0.0028 | Tumor hypermethylation in CpG island core |
| ETV5 | 2119 | 3 | 187305595 | 187313595 | 0 | Tumor hypermethylation in CpG island core |
| DGKG | 1608 | 3 | 187558717 | 187566717 | 0.0026 | Tumor hypermethylation in CpG island core |
| ST6GAL1 | 6480 | 3 | 188127209 | 188135209 | 0.0056 | Tumor hypermethylation in CpG island core |
| FGF12 | 2257 | 3 | 193605532 | 193613532 | 0 | Tumor hypermethylation in CpG island core |
| LOC348840 | 348840 | 3 | 199287939 | 199295939 | 0.0018 | Tumor hypermethylation in CpG island core |
| ZNF876P | 642280 | 4 | 192388 | 200388 | 0.0001 | Tumor hypermethylation in CpG island core |
| CPLX1 | 10815 | 4 | 805945 | 813945 | 0.0003 | Tumor hypermethylation in CpG island core |
| RNF212 | 285498 | 4 | 1093582 | 1101582 | 0.0028 | Tumor hypermethylation in CpG island core |
| SPON2 | 10417 | 4 | 1152641 | 1160641 | 0 | Tumor hypermethylation in CpG island core |
| LOC100130872 | 1E+08 | 4 | 1188750 | 1196750 | 0 | Tumor hypermethylation in CpG island core |
| MIR943 | 1E+08 | 4 | 1954002 | 1962002 | 0 | Tumor hypermethylation in CpG island core |
| NAT8L | 339983 | 4 | 2027036 | 2035036 | 0.001 | Tumor hypermethylation in CpG island core |
| C4orf44 | 345222 | 4 | 3216564 | 3224564 | 0.0004 | Tumor hypermethylation in CpG island core |
| ADRA2C | 152 | 4 | 3734093 | 3742093 | 0.0022 | Tumor hypermethylation in CpG island core |
| OTOP1 | 133060 | 4 | 4275522 | 4283522 | 0.0026 | Tumor hypermethylation in CpG island core |
| D4S234E | 27065 | 4 | 4434883 | 4442883 | 0 | Tumor hypermethylation in CpG island core |
| STK32B | 55351 | 4 | 5100427 | 5108427 | 0 | Tumor hypermethylation in CpG island core |
| EVC2 | 132884 | 4 | 5757195 | 5765195 | 0.0018 | Tumor hypermethylation in CpG island core |
| DRD5 | 1816 | 4 | 9388355 | 9396355 | 0.0048 | Tumor hypermethylation in CpG island core |
| ZNF518B | 85460 | 4 | 10064130 | 10072130 | 0.0095 | Tumor hypermethylation in CpG island core |
| HS3ST1 | 9957 | 4 | 11035635 | 11043635 | 0 | Tumor hypermethylation in CpG island core |
| LOC285548 | 285548 | 4 | 13154546 | 13162546 | 0 | Tumor hypermethylation in CpG island core |
| CD38 | 952 | 4 | 15385028 | 15393028 | 0.0013 | Tumor hypermethylation in CpG island core |
| PROM1 | 8842 | 4 | 15690692 | 15698692 | 0 | Tumor hypermethylation in CpG island core |
| SLIT2 | 9353 | 4 | 19860332 | 19868332 | 0 | Tumor hypermethylation in CpG island core |
| PCDH7 | 5099 | 4 | 30327134 | 30335134 | 0 | Tumor hypermethylation in CpG island core |
| KIAA1239 | 57495 | 4 | 36919084 | 36927084 | 0 | Tumor hypermethylation in CpG island core |
| UCHL1 | 7345 | 4 | 40949654 | 40957654 | 0.0047 | Tumor hypermethylation in CpG island core |
| DCAF4L1 | 285429 | 4 | 41674469 | 41682469 | 0.0077 | Tumor hypermethylation in CpG island core |
| BEND4 | 389206 | 4 | 41845652 | 41853652 | 0.0014 | Tumor hypermethylation in CpG island core |
| SHISA3 | 152573 | 4 | 42090612 | 42098612 | 0 | Tumor hypermethylation in CpG island core |
| KCTD8 | 386617 | 4 | 44141581 | 44149581 | 0 | Tumor hypermethylation in CpG island core |
| GABRA4 | 2557 | 4 | 46686337 | 46694337 | 0.0003 | Tumor hypermethylation in CpG island core |
| CORIN | 10699 | 4 | 47530816 | 47538816 | 0.0005 | Tumor hypermethylation in CpG island core |
| ZAR1 | 326340 | 4 | 48183065 | 48191065 | 0.006 | Tumor hypermethylation in CpG island core |
| SPATA18 | 132671 | 4 | 52608349 | 52616349 | 0.005 | Tumor hypermethylation in CpG island core |
| GSX2 | 170825 | 4 | 54657004 | 54665004 | 0 | Tumor hypermethylation in CpG island core |
| KIT | 3815 | 4 | 55214851 | 55222851 | 0.0003 | Tumor hypermethylation in CpG island core |
| KDR | 3791 | 4 | 55682519 | 55690519 | 0.0021 | Tumor hypermethylation in CpG island core |
| ARL9 | 132946 | 4 | 57062131 | 57070131 | 0.0096 | Tumor hypermethylation in CpG island core |
| HOPX | 84525 | 4 | 57213445 | 57221445 | 0 | Tumor hypermethylation in CpG island core |
| IGFBP7 | 3490 | 4 | 57667296 | 57675296 | 0 | Tumor hypermethylation in CpG island core |
| EPHA5 | 2044 | 4 | 66214248 | 66222248 | 0 | Tumor hypermethylation in CpG island core |
| CXCL6 | 6372 | 4 | 74917136 | 74925136 | 0.0089 | Tumor hypermethylation in CpG island core |
| CXCL1 | 2919 | 4 | 74949972 | 74957972 | 0.0062 | Tumor hypermethylation in CpG island core |
| CXCL2 | 2920 | 4 | 75179861 | 75187861 | 0.0032 | Tumor hypermethylation in CpG island core |
| CDKL2 | 8999 | 4 | 76770745 | 76778745 | 0.001 | Tumor hypermethylation in CpG island core |
| PRDM8 | 56978 | 4 | 81321447 | 81329447 | 0.006 | Tumor hypermethylation in CpG island core |
| FGF5 | 2250 | 4 | 81402765 | 81410765 | 0 | Tumor hypermethylation in CpG island core |
| BMP3 | 651 | 4 | 82167142 | 82175142 | 0.0022 | Tumor hypermethylation in CpG island core |
| SNCA | 6622 | 4 | 90973373 | 90981373 | 0 | Tumor hypermethylation in CpG island core |
| GRID2 | 2895 | 4 | 93440572 | 93448572 | 0 | Tumor hypermethylation in CpG island core |
| ATOH1 | 474 | 4 | 94965100 | 94973100 | 0 | Tumor hypermethylation in CpG island core |
| UNC5C | 8633 | 4 | 96685185 | 96693185 | 0 | Tumor hypermethylation in CpG island core |
| DDIT4L | 115265 | 4 | 101326636 | 101334636 | 0.0035 | Tumor hypermethylation in CpG island core |
| LOC641518 | 641518 | 4 | 109308724 | 109316724 | 0 | Tumor hypermethylation in CpG island core |
| COL25A1 | 84570 | 4 | 110439248 | 110447248 | 0.0003 | Tumor hypermethylation in CpG island core |
| PITX2 | 5308 | 4 | 111759703 | 111767703 | 0 | Tumor hypermethylation in CpG island core |
| NEUROG2 | 63973 | 4 | 113652777 | 113660777 | 0 | Tumor hypermethylation in CpG island core |
| PRDM5 | 11107 | 4 | 122059463 | 122067463 | 0.0014 | Tumor hypermethylation in CpG island core |
| C4orf31 | 79625 | 4 | 122209123 | 122217123 | 0 | Tumor hypermethylation in CpG island core |
| QRFPR | 84109 | 4 | 122517631 | 122525631 | 0.0015 | Tumor hypermethylation in CpG island core |
| FGF13 | 2258 | 4 | 123963312 | 123971312 | 0.0069 | Tumor hypermethylation in CpG island core |
| FAT4 | 79633 | 4 | 126453016 | 126461016 | 0 | Tumor hypermethylation in CpG island core |
| C4orf49 | 84709 | 4 | 140416942 | 140424942 | 0.0058 | Tumor hypermethylation in CpG island core |
| UCP1 | 7350 | 4 | 141705409 | 141713409 | 0 | Tumor hypermethylation in CpG island core |
| POU4F2 | 5458 | 4 | 147775494 | 147783494 | 0 | Tumor hypermethylation in CpG island core |
| SFRP2 | 6423 | 4 | 154925678 | 154933678 | 0 | Tumor hypermethylation in CpG island core |
| DCHS2 | 54798 | 4 | 155628327 | 155636327 | 0.003 | Tumor hypermethylation in CpG island core |
| LRAT | 9227 | 4 | 155880612 | 155888612 | 0.0001 | Tumor hypermethylation in CpG island core |
| NPY2R | 4887 | 4 | 156345230 | 156353230 | 0.0008 | Tumor hypermethylation in CpG island core |
| GUCY1B3 | 2983 | 4 | 156895575 | 156903575 | 0 | Tumor hypermethylation in CpG island core |
| NPY5R | 4889 | 4 | 164480540 | 164488540 | 0.0004 | Tumor hypermethylation in CpG island core |
| 1-Mar | 55016 | 4 | 165519857 | 165527857 | 0.0003 | Tumor hypermethylation in CpG island core |
| GALNTL6 | 442117 | 4 | 172967149 | 172975149 | 0.0069 | Tumor hypermethylation in CpG island core |
| HAND2 | 9464 | 4 | 174683953 | 174691953 | 0 | Tumor hypermethylation in CpG island core |
| MGC45800 | 90768 | 4 | 183298662 | 183306662 | 0 | Tumor hypermethylation in CpG island core |
| STOX2 | 56977 | 4 | 185059502 | 185067502 | 0 | Tumor hypermethylation in CpG island core |
| HELT | 391723 | 4 | 186173076 | 186181076 | 0 | Tumor hypermethylation in CpG island core |
| FAT1 | 2195 | 4 | 187877981 | 187885981 | 0 | Tumor hypermethylation in CpG island core |
| SLC9A3 | 6550 | 5 | 573549 | 581549 | 0 | Tumor hypermethylation in CpG island core |
| TERT | 7015 | 5 | 1344162 | 1352162 | 0 | Tumor hypermethylation in CpG island core |
| SLC6A3 | 6531 | 5 | 1494543 | 1502543 | 0 | Tumor hypermethylation in CpG island core |
| IRX1 | 79192 | 5 | 3645167 | 3653167 | 0 | Tumor hypermethylation in CpG island core |
| ADAMTS16 | 170690 | 5 | 5189442 | 5197442 | 0.0004 | Tumor hypermethylation in CpG island core |
| ADCY2 | 108 | 5 | 7445342 | 7453342 | 0.008 | Tumor hypermethylation in CpG island core |
| SEMA5A | 9037 | 5 | 9595233 | 9603233 | 0 | Tumor hypermethylation in CpG island core |
| ANKRD33B | 651746 | 5 | 10613434 | 10621434 | 0.0001 | Tumor hypermethylation in CpG island core |
| 11-Mar | 441061 | 5 | 16228897 | 16236897 | 0 | Tumor hypermethylation in CpG island core |
| BASP1 | 10409 | 5 | 17266749 | 17274749 | 0 | Tumor hypermethylation in CpG island core |
| CDH6 | 1004 | 5 | 31225552 | 31233552 | 0.0098 | Tumor hypermethylation in CpG island core |
| C16orf35 | 8131 | 5 | 32743421 | 32751421 | 0 | Tumor hypermethylation in CpG island core |
| ADAMTS12 | 81792 | 5 | 33923881 | 33931881 | 0.0003 | Tumor hypermethylation in CpG island core |
| EGFLAM | 133584 | 5 | 38290289 | 38298289 | 0 | Tumor hypermethylation in CpG island core |
| OSMR | 9180 | 5 | 38877716 | 38885716 | 0.0004 | Tumor hypermethylation in CpG island core |
| OXCT1 | 5019 | 5 | 41902548 | 41910548 | 0.0063 | Tumor hypermethylation in CpG island core |
| C5orf39 | 389289 | 5 | 43072204 | 43080204 | 0 | Tumor hypermethylation in CpG island core |
| LOC153684 | 153684 | 5 | 43073992 | 43081992 | 0 | Tumor hypermethylation in CpG island core |
| MGC42105 | 167359 | 5 | 43224083 | 43232083 | 0 | Tumor hypermethylation in CpG island core |
| ISL1 | 3670 | 5 | 50710714 | 50718714 | 0 | Tumor hypermethylation in CpG island core |
| PDE4D | 5144 | 5 | 59221378 | 59229378 | 0.0002 | Tumor hypermethylation in CpG island core |
| HTR1A | 3350 | 5 | 63289302 | 63297302 | 0 | Tumor hypermethylation in CpG island core |
| FAM159B | 1E+08 | 5 | 64017890 | 64025890 | 0.0002 | Tumor hypermethylation in CpG island core |
| CARTPT | 9607 | 5 | 71046745 | 71054745 | 0.0001 | Tumor hypermethylation in CpG island core |
| SV2C | 22987 | 5 | 75411060 | 75419060 | 0.0003 | Tumor hypermethylation in CpG island core |
| F2R | 2149 | 5 | 76043623 | 76051623 | 0.0087 | Tumor hypermethylation in CpG island core |
| ANKRD34B | 340120 | 5 | 79898060 | 79906060 | 0.0002 | Tumor hypermethylation in CpG island core |
| ACOT12 | 134526 | 5 | 80721744 | 80729744 | 0.0004 | Tumor hypermethylation in CpG island core |
| VCAN | 1462 | 5 | 82799248 | 82807248 | 0.0001 | Tumor hypermethylation in CpG island core |
| EDIL3 | 10085 | 5 | 83712367 | 83720367 | 0 | Tumor hypermethylation in CpG island core |
| LOC645323 | 645323 | 5 | 88000902 | 88008902 | 0 | Tumor hypermethylation in CpG island core |
| GPR150 | 285601 | 5 | 94977735 | 94985735 | 0.0001 | Tumor hypermethylation in CpG island core |
| PCSK1 | 5122 | 5 | 95790708 | 95798708 | 0 | Tumor hypermethylation in CpG island core |
| ST8SIA4 | 7903 | 5 | 100262886 | 100270886 | 0.0006 | Tumor hypermethylation in CpG island core |
| EFNA5 | 1946 | 5 | 107030495 | 107038495 | 0 | Tumor hypermethylation in CpG island core |
| KCNN2 | 3781 | 5 | 113721914 | 113729914 | 0 | Tumor hypermethylation in CpG island core |
| TRIM36 | 55521 | 5 | 114540142 | 114548142 | 0.0051 | Tumor hypermethylation in CpG island core |
| CDO1 | 1036 | 5 | 115176304 | 115184304 | 0 | Tumor hypermethylation in CpG island core |
| LVRN | 206338 | 5 | 115322049 | 115330049 | 0 | Tumor hypermethylation in CpG island core |
| SNCAIP | 9627 | 5 | 121671718 | 121679718 | 0.0001 | Tumor hypermethylation in CpG island core |
| PRDM6 | 93166 | 5 | 122448739 | 122456739 | 0.0041 | Tumor hypermethylation in CpG island core |
| FBN2 | 2201 | 5 | 127897634 | 127905634 | 0 | Tumor hypermethylation in CpG island core |
| PDLIM4 | 8572 | 5 | 131617249 | 131625249 | 0.009 | Tumor hypermethylation in CpG island core |
| TCF7 | 6932 | 5 | 133474300 | 133482300 | 0.0024 | Tumor hypermethylation in CpG island core |
| NEUROG1 | 4762 | 5 | 134895538 | 134903538 | 0 | Tumor hypermethylation in CpG island core |
| CXCL14 | 9547 | 5 | 134938868 | 134946868 | 0 | Tumor hypermethylation in CpG island core |
| LOC389332 | 389332 | 5 | 135552750 | 135560750 | 0.0081 | Tumor hypermethylation in CpG island core |
| LOC389333 | 389333 | 5 | 138754784 | 138762784 | 0 | Tumor hypermethylation in CpG island core |
| NDUFA2 | 4695 | 5 | 139989194 | 139997194 | 0.0005 | Tumor hypermethylation in CpG island core |
| DND1 | 373863 | 5 | 140029355 | 140037355 | 0.0001 | Tumor hypermethylation in CpG island core |
| PCDHA3 | 56145 | 5 | 140156966 | 140164966 | 0.0058 | Tumor hypermethylation in CpG island core |
| PCDHA13 | 56136 | 5 | 140238037 | 140246037 | 0.0069 | Tumor hypermethylation in CpG island core |
| PCDHAC1 | 56135 | 5 | 140282485 | 140290485 | 0.0001 | Tumor hypermethylation in CpG island core |
| PCDHGB6 | 56100 | 5 | 140763953 | 140771953 | 0.0016 | Tumor hypermethylation in CpG island core |
| PCDHGC3 | 5098 | 5 | 140831752 | 140839752 | 0.0001 | Tumor hypermethylation in CpG island core |
| POU4F3 | 5459 | 5 | 145694779 | 145702779 | 0 | Tumor hypermethylation in CpG island core |
| DPYSL3 | 1809 | 5 | 146809453 | 146817453 | 0.0002 | Tumor hypermethylation in CpG island core |
| CDX1 | 1044 | 5 | 149522536 | 149530536 | 0.0015 | Tumor hypermethylation in CpG island core |
| ARSI | 340075 | 5 | 149658718 | 149666718 | 0.0013 | Tumor hypermethylation in CpG island core |
| GPX3 | 2878 | 5 | 150376191 | 150384191 | 0.0003 | Tumor hypermethylation in CpG island core |
| GLRA1 | 2741 | 5 | 151280590 | 151288590 | 0.0031 | Tumor hypermethylation in CpG island core |
| HAND1 | 9421 | 5 | 153834017 | 153842017 | 0 | Tumor hypermethylation in CpG island core |
| ADAM19 | 8728 | 5 | 156931346 | 156939346 | 0.0019 | Tumor hypermethylation in CpG island core |
| SOX30 | 11063 | 5 | 157008006 | 157016006 | 0.0063 | Tumor hypermethylation in CpG island core |
| EBF1 | 1879 | 5 | 158455366 | 158463366 | 0 | Tumor hypermethylation in CpG island core |
| GABRB2 | 2561 | 5 | 160903708 | 160911708 | 0 | Tumor hypermethylation in CpG island core |
| RANBP17 | 64901 | 5 | 170217599 | 170225599 | 0 | Tumor hypermethylation in CpG island core |
| TLX3 | 30012 | 5 | 170664892 | 170672892 | 0 | Tumor hypermethylation in CpG island core |
| C5orf47 | 133491 | 5 | 173344767 | 173352767 | 0.0043 | Tumor hypermethylation in CpG island core |
| DRD1 | 1812 | 5 | 174799769 | 174807769 | 0.0042 | Tumor hypermethylation in CpG island core |
| ARL10 | 285598 | 5 | 175721107 | 175729107 | 0.0006 | Tumor hypermethylation in CpG island core |
| SNCB | 6620 | 5 | 175986163 | 175994163 | 0.0017 | Tumor hypermethylation in CpG island core |
| UNC5A | 90249 | 5 | 176166165 | 176174165 | 0 | Tumor hypermethylation in CpG island core |
| NSD1 | 64324 | 5 | 176488685 | 176496685 | 0.003 | Tumor hypermethylation in CpG island core |
| PFN3 | 345456 | 5 | 176756243 | 176764243 | 0.0001 | Tumor hypermethylation in CpG island core |
| COL23A1 | 91522 | 5 | 177946162 | 177954162 | 0.0005 | Tumor hypermethylation in CpG island core |
| ZNF454 | 285676 | 5 | 178296829 | 178304829 | 0.0001 | Tumor hypermethylation in CpG island core |
| GRM6 | 2916 | 5 | 178350730 | 178358730 | 0.0005 | Tumor hypermethylation in CpG island core |
| ADAMTS2 | 9509 | 5 | 178700935 | 178708935 | 0 | Tumor hypermethylation in CpG island core |
| LTC4S | 4056 | 5 | 179149591 | 179157591 | 0.0099 | Tumor hypermethylation in CpG island core |
| GFPT2 | 9945 | 5 | 179708921 | 179716921 | 0.0024 | Tumor hypermethylation in CpG island core |
| SCGB3A1 | 92304 | 5 | 179947093 | 179955093 | 0.0008 | Tumor hypermethylation in CpG island core |
| FLT4 | 2324 | 5 | 180005230 | 180013230 | 0.0013 | Tumor hypermethylation in CpG island core |
| MGAT1 | 116255 | 5 | 180158654 | 180166654 | 0.0039 | Tumor hypermethylation in CpG island core |
| TRIM7 | 81786 | 5 | 180560783 | 180568783 | 0.006 | Tumor hypermethylation in CpG island core |
| IRF4 | 3662 | 6 | 332751 | 340751 | 0 | Tumor hypermethylation in CpG island core |
| FOXQ1 | 94234 | 6 | 1253674 | 1261674 | 0 | Tumor hypermethylation in CpG island core |
| FOXF2 | 2295 | 6 | 1331067 | 1339067 | 0 | Tumor hypermethylation in CpG island core |
| FOXC1 | 2296 | 6 | 1551679 | 1559679 | 0 | Tumor hypermethylation in CpG island core |
| SERPINB9 | 5272 | 6 | 2844544 | 2852544 | 0.0001 | Tumor hypermethylation in CpG island core |
| C6orf145 | 221749 | 6 | 3693245 | 3701245 | 0.0008 | Tumor hypermethylation in CpG island core |
| FAM50B | 26240 | 6 | 3790630 | 3798630 | 0 | Tumor hypermethylation in CpG island core |
| PPP1R3G | 648791 | 6 | 5026718 | 5034718 | 0.0088 | Tumor hypermethylation in CpG island core |
| NRN1 | 51299 | 6 | 5948632 | 5956632 | 0 | Tumor hypermethylation in CpG island core |
| BMP6 | 654 | 6 | 7668009 | 7676009 | 0 | Tumor hypermethylation in CpG island core |
| GCM2 | 9247 | 6 | 10986084 | 10994084 | 0 | Tumor hypermethylation in CpG island core |
| RBM24 | 221662 | 6 | 17385787 | 17393787 | 0.006 | Tumor hypermethylation in CpG island core |
| ID4 | 3400 | 6 | 19941595 | 19949595 | 0 | Tumor hypermethylation in CpG island core |
| HDGFL1 | 154150 | 6 | 22673656 | 22681656 | 0.0041 | Tumor hypermethylation in CpG island core |
| HIST1H4A | 8359 | 6 | 26125885 | 26133885 | 0 | Tumor hypermethylation in CpG island core |
| HIST1H2AB | 8335 | 6 | 26137775 | 26145775 | 0.0007 | Tumor hypermethylation in CpG island core |
| HIST1H2BB | 3018 | 6 | 26147864 | 26155864 | 0 | Tumor hypermethylation in CpG island core |
| HIST1H3C | 8352 | 6 | 26149617 | 26157617 | 0 | Tumor hypermethylation in CpG island core |
| HIST1H4D | 8360 | 6 | 26293283 | 26301283 | 0.0031 | Tumor hypermethylation in CpG island core |
| HIST1H4E | 8367 | 6 | 26308851 | 26316851 | 0.0022 | Tumor hypermethylation in CpG island core |
| HIST1H3E | 8353 | 6 | 26329361 | 26337361 | 0.0069 | Tumor hypermethylation in CpG island core |
| HIST1H4F | 8361 | 6 | 26344632 | 26352632 | 0 | Tumor hypermethylation in CpG island core |
| HIST1H3F | 8968 | 6 | 26354814 | 26362814 | 0 | Tumor hypermethylation in CpG island core |
| HIST1H2BH | 8345 | 6 | 26355857 | 26363857 | 0.0001 | Tumor hypermethylation in CpG island core |
| HIST1H3G | 8355 | 6 | 26375591 | 26383591 | 0 | Tumor hypermethylation in CpG island core |
| HIST1H2BI | 8346 | 6 | 26377182 | 26385182 | 0 | Tumor hypermethylation in CpG island core |
| HCG11 | 493812 | 6 | 26625912 | 26633912 | 0.0077 | Tumor hypermethylation in CpG island core |
| HIST1H2BJ | 8970 | 6 | 27204554 | 27212554 | 0 | Tumor hypermethylation in CpG island core |
| HIST1H4I | 8294 | 6 | 27211066 | 27219066 | 0.0056 | Tumor hypermethylation in CpG island core |
| POM121L2 | 94026 | 6 | 27383990 | 27391990 | 0.0008 | Tumor hypermethylation in CpG island core |
| HIST1H1B | 3009 | 6 | 27939338 | 27947338 | 0 | Tumor hypermethylation in CpG island core |
| HIST1H3I | 8354 | 6 | 27944078 | 27952078 | 0 | Tumor hypermethylation in CpG island core |
| HIST1H4L | 8368 | 6 | 27945268 | 27953268 | 0 | Tumor hypermethylation in CpG island core |
| HIST1H3J | 8356 | 6 | 27962549 | 27970549 | 0.0052 | Tumor hypermethylation in CpG island core |
| ZSCAN12 | 9753 | 6 | 28471523 | 28479523 | 0.0001 | Tumor hypermethylation in CpG island core |
| GABBR1 | 10537 | 6 | 29699984 | 29707984 | 0.009 | Tumor hypermethylation in CpG island core |
| HCG4 | 54435 | 6 | 29864829 | 29872829 | 0.0006 | Tumor hypermethylation in CpG island core |
| HCG4P6 | 80868 | 6 | 29997407 | 30005407 | 0.0011 | Tumor hypermethylation in CpG island core |
| HLA-J | 3137 | 6 | 30077726 | 30085726 | 0.0001 | Tumor hypermethylation in CpG island core |
| COL11A2 | 1302 | 6 | 33264223 | 33272223 | 0.0034 | Tumor hypermethylation in CpG island core |
| HMGA1 | 3159 | 6 | 34308554 | 34316554 | 0.0088 | Tumor hypermethylation in CpG island core |
| TULP1 | 7287 | 6 | 35584625 | 35592625 | 0.0072 | Tumor hypermethylation in CpG island core |
| GLP1R | 2740 | 6 | 39120534 | 39128534 | 0.0005 | Tumor hypermethylation in CpG island core |
| KCNK17 | 89822 | 6 | 39386214 | 39394214 | 0 | Tumor hypermethylation in CpG island core |
| LRFN2 | 57497 | 6 | 40659104 | 40667104 | 0.0062 | Tumor hypermethylation in CpG island core |
| MDFI | 4188 | 6 | 41710172 | 41718172 | 0.0001 | Tumor hypermethylation in CpG island core |
| GNMT | 27232 | 6 | 43032477 | 43040477 | 0.0039 | Tumor hypermethylation in CpG island core |
| RSPH9 | 221421 | 6 | 43716787 | 43724787 | 0.0007 | Tumor hypermethylation in CpG island core |
| TDRD6 | 221400 | 6 | 46759570 | 46767570 | 0.0001 | Tumor hypermethylation in CpG island core |
| PLA2G7 | 7941 | 6 | 46807110 | 46815110 | 0 | Tumor hypermethylation in CpG island core |
| PAQR8 | 85315 | 6 | 52330884 | 52338884 | 0.001 | Tumor hypermethylation in CpG island core |
| HCRTR2 | 131450 | 6 | 55143029 | 55151029 | 0.0072 | Tumor hypermethylation in CpG island core |
| COL9A1 | 1297 | 6 | 71045632 | 71053632 | 0.0011 | Tumor hypermethylation in CpG island core |
| KCNQ5 | 56479 | 6 | 73384291 | 73392291 | 0.003 | Tumor hypermethylation in CpG island core |
| C6orf147 | 387097 | 6 | 74072809 | 74080809 | 0.005 | Tumor hypermethylation in CpG island core |
| DPPA5 | 340168 | 6 | 74116674 | 74124674 | 0.0004 | Tumor hypermethylation in CpG island core |
| C6orf221 | 154288 | 6 | 74125120 | 74133120 | 0.0008 | Tumor hypermethylation in CpG island core |
| HTR1B | 3351 | 6 | 78225839 | 78233839 | 0 | Tumor hypermethylation in CpG island core |
| SNAP91 | 9892 | 6 | 84471846 | 84479846 | 0.002 | Tumor hypermethylation in CpG island core |
| RIPPLY2 | 134701 | 6 | 84615703 | 84623703 | 0.0001 | Tumor hypermethylation in CpG island core |
| TBX18 | 9096 | 6 | 85526618 | 85534618 | 0 | Tumor hypermethylation in CpG island core |
| NT5E | 4907 | 6 | 86212020 | 86220020 | 0 | Tumor hypermethylation in CpG island core |
| CNR1 | 56144 | 6 | 88928486 | 88936486 | 0.0001 | Tumor hypermethylation in CpG island core |
| EPHA7 | 2045 | 6 | 94182021 | 94190021 | 0 | Tumor hypermethylation in CpG island core |
| MCHR2 | 84539 | 6 | 100544820 | 100552820 | 0.0062 | Tumor hypermethylation in CpG island core |
| SIM1 | 6492 | 6 | 101014272 | 101022272 | 0 | Tumor hypermethylation in CpG island core |
| BVES | 11149 | 6 | 105687236 | 105695236 | 0 | Tumor hypermethylation in CpG island core |
| AIM1 | 51151 | 6 | 107062422 | 107070422 | 0 | Tumor hypermethylation in CpG island core |
| SOBP | 55084 | 6 | 107914009 | 107922009 | 0 | Tumor hypermethylation in CpG island core |
| NR2E1 | 7101 | 6 | 108589907 | 108597907 | 0 | Tumor hypermethylation in CpG island core |
| GPR6 | 2830 | 6 | 110402990 | 110410990 | 0.0048 | Tumor hypermethylation in CpG island core |
| C6orf186 | 728464 | 6 | 110782168 | 110790168 | 0.0077 | Tumor hypermethylation in CpG island core |
| SLC16A10 | 117247 | 6 | 111511473 | 111519473 | 0.0009 | Tumor hypermethylation in CpG island core |
| MARCKS | 4082 | 6 | 114281219 | 114289219 | 0 | Tumor hypermethylation in CpG island core |
| FAM162B | 221303 | 6 | 117189579 | 117197579 | 0.0028 | Tumor hypermethylation in CpG island core |
| VGLL2 | 245806 | 6 | 117689413 | 117697413 | 0 | Tumor hypermethylation in CpG island core |
| SLC35F1 | 222553 | 6 | 118331381 | 118339381 | 0 | Tumor hypermethylation in CpG island core |
| CLVS2 | 134829 | 6 | 123355280 | 123363280 | 0.0002 | Tumor hypermethylation in CpG island core |
| NKAIN2 | 154215 | 6 | 124162767 | 124170767 | 0.0001 | Tumor hypermethylation in CpG island core |
| STL | 7955 | 6 | 125321872 | 125329872 | 0 | Tumor hypermethylation in CpG island core |
| HEY2 | 23493 | 6 | 126108424 | 126116424 | 0 | Tumor hypermethylation in CpG island core |
| EYA4 | 2070 | 6 | 133600187 | 133608187 | 0.0002 | Tumor hypermethylation in CpG island core |
| SLC35D3 | 340146 | 6 | 137281094 | 137289094 | 0 | Tumor hypermethylation in CpG island core |
| OLIG3 | 167826 | 6 | 137853224 | 137861224 | 0 | Tumor hypermethylation in CpG island core |
| NMBR | 4829 | 6 | 142447629 | 142455629 | 0.0058 | Tumor hypermethylation in CpG island core |
| PHACTR2 | 9749 | 6 | 144036794 | 144044794 | 0.0005 | Tumor hypermethylation in CpG island core |
| GRM1 | 2911 | 6 | 146386474 | 146394474 | 0.0001 | Tumor hypermethylation in CpG island core |
| SAMD5 | 389432 | 6 | 147867755 | 147875755 | 0 | Tumor hypermethylation in CpG island core |
| ULBP1 | 80329 | 6 | 150322835 | 150330835 | 0 | Tumor hypermethylation in CpG island core |
| PPP1R14C | 81706 | 6 | 150501880 | 150509880 | 0.0068 | Tumor hypermethylation in CpG island core |
| PLEKHG1 | 57480 | 6 | 150958691 | 150966691 | 0.0007 | Tumor hypermethylation in CpG island core |
| AKAP12 | 9590 | 6 | 151598826 | 151606826 | 0.0036 | Tumor hypermethylation in CpG island core |
| SYNE1 | 23345 | 6 | 152995679 | 153003679 | 0.0043 | Tumor hypermethylation in CpG island core |
| RGS17 | 26575 | 6 | 153490082 | 153498082 | 0 | Tumor hypermethylation in CpG island core |
| OPRM1 | 4988 | 6 | 154398238 | 154406238 | 0.003 | Tumor hypermethylation in CpG island core |
| FNDC1 | 84624 | 6 | 159506416 | 159514416 | 0.0012 | Tumor hypermethylation in CpG island core |
| SOAT2 | 8435 | 6 | 160098978 | 160106978 | 0.0009 | Tumor hypermethylation in CpG island core |
| SLC22A3 | 6581 | 6 | 160685414 | 160693414 | 0.0002 | Tumor hypermethylation in CpG island core |
| PDE10A | 10846 | 6 | 165991574 | 165999574 | 0 | Tumor hypermethylation in CpG island core |
| C6orf176 | 90632 | 6 | 166317517 | 166325517 | 0.0025 | Tumor hypermethylation in CpG island core |
| T | 6862 | 6 | 166498121 | 166506121 | 0 | Tumor hypermethylation in CpG island core |
| SMOC2 | 64094 | 6 | 168580679 | 168588679 | 0.001 | Tumor hypermethylation in CpG island core |
| PDGFA | 5154 | 7 | 522007 | 530007 | 0.0036 | Tumor hypermethylation in CpG island core |
| PRKAR1B | 5575 | 7 | 714687 | 722687 | 0.0073 | Tumor hypermethylation in CpG island core |
| MIR339 | 442907 | 7 | 1025188 | 1033188 | 0 | Tumor hypermethylation in CpG island core |
| GPR146 | 115330 | 7 | 1059666 | 1067666 | 0 | Tumor hypermethylation in CpG island core |
| RADIL | 55698 | 7 | 4885861 | 4893861 | 0.007 | Tumor hypermethylation in CpG island core |
| LOC389458 | 389458 | 7 | 5074263 | 5082263 | 0.0025 | Tumor hypermethylation in CpG island core |
| FSCN1 | 6624 | 7 | 5594979 | 5602979 | 0 | Tumor hypermethylation in CpG island core |
| NXPH1 | 30010 | 7 | 8436109 | 8444109 | 0 | Tumor hypermethylation in CpG island core |
| TWIST1 | 7291 | 7 | 19119820 | 19127820 | 0 | Tumor hypermethylation in CpG island core |
| FERD3L | 222894 | 7 | 19147569 | 19155569 | 0 | Tumor hypermethylation in CpG island core |
| TMEM196 | 256130 | 7 | 19774929 | 19782929 | 0 | Tumor hypermethylation in CpG island core |
| SP8 | 221833 | 7 | 20789033 | 20797033 | 0 | Tumor hypermethylation in CpG island core |
| IGF2BP3 | 10643 | 7 | 23472520 | 23480520 | 0 | Tumor hypermethylation in CpG island core |
| NPY | 4852 | 7 | 24286331 | 24294331 | 0 | Tumor hypermethylation in CpG island core |
| DFNA5 | 1687 | 7 | 24759608 | 24767608 | 0.0003 | Tumor hypermethylation in CpG island core |
| MIR148A | 406940 | 7 | 25952131 | 25960131 | 0.0015 | Tumor hypermethylation in CpG island core |
| SKAP2 | 8935 | 7 | 26866866 | 26874866 | 0.0097 | Tumor hypermethylation in CpG island core |
| HOXA1 | 3198 | 7 | 27098150 | 27106150 | 0 | Tumor hypermethylation in CpG island core |
| HOXA4 | 3201 | 7 | 27132924 | 27140924 | 0 | Tumor hypermethylation in CpG island core |
| HOXA5 | 3202 | 7 | 27145812 | 27153812 | 0 | Tumor hypermethylation in CpG island core |
| NCOA6 | 23054 | 7 | 27158821 | 27166821 | 0.0001 | Tumor hypermethylation in CpG island core |
| HOXA9 | 3205 | 7 | 27167674 | 27175674 | 0 | Tumor hypermethylation in CpG island core |
| HOXA10 | 3206 | 7 | 27176480 | 27184480 | 0.0006 | Tumor hypermethylation in CpG island core |
| HOXA11 | 3207 | 7 | 27187360 | 27195360 | 0 | Tumor hypermethylation in CpG island core |
| HOXA13 | 3209 | 7 | 27202250 | 27210250 | 0 | Tumor hypermethylation in CpG island core |
| TRIL | 9865 | 7 | 28960554 | 28968554 | 0 | Tumor hypermethylation in CpG island core |
| CRHR2 | 1395 | 7 | 30684665 | 30692665 | 0 | Tumor hypermethylation in CpG island core |
| ADCYAP1R1 | 117 | 7 | 31054666 | 31062666 | 0.0004 | Tumor hypermethylation in CpG island core |
| PDE1C | 5137 | 7 | 32073516 | 32081516 | 0.0004 | Tumor hypermethylation in CpG island core |
| BMPER | 168667 | 7 | 33907636 | 33915636 | 0 | Tumor hypermethylation in CpG island core |
| TBX20 | 57057 | 7 | 35256236 | 35264236 | 0 | Tumor hypermethylation in CpG island core |
| ELMO1 | 9844 | 7 | 37451036 | 37459036 | 0 | Tumor hypermethylation in CpG island core |
| SFRP4 | 6424 | 7 | 37919050 | 37927050 | 0.0002 | Tumor hypermethylation in CpG island core |
| HECW1 | 23072 | 7 | 43114722 | 43122722 | 0 | Tumor hypermethylation in CpG island core |
| IGFBP3 | 3486 | 7 | 45923396 | 45931396 | 0 | Tumor hypermethylation in CpG island core |
| C7orf57 | 136288 | 7 | 48037641 | 48045641 | 0 | Tumor hypermethylation in CpG island core |
| VWC2 | 375567 | 7 | 49779802 | 49787802 | 0.0029 | Tumor hypermethylation in CpG island core |
| VSTM2A | 222008 | 7 | 54573512 | 54581512 | 0.0015 | Tumor hypermethylation in CpG island core |
| EGFR | 1956 | 7 | 55050218 | 55058218 | 0 | Tumor hypermethylation in CpG island core |
| WBSCR17 | 64409 | 7 | 70231724 | 70239724 | 0 | Tumor hypermethylation in CpG island core |
| CALN1 | 83698 | 7 | 71436144 | 71444144 | 0 | Tumor hypermethylation in CpG island core |
| FZD3 | 8326 | 7 | 72482044 | 72490044 | 0.0015 | Tumor hypermethylation in CpG island core |
| CLDN3 | 1365 | 7 | 72818536 | 72826536 | 0.0071 | Tumor hypermethylation in CpG island core |
| MAGI2 | 9863 | 7 | 78916826 | 78924826 | 0 | Tumor hypermethylation in CpG island core |
| ZNF804B | 219578 | 7 | 88222688 | 88230688 | 0 | Tumor hypermethylation in CpG island core |
| SGCE | 8910 | 7 | 94119457 | 94127457 | 0.0005 | Tumor hypermethylation in CpG island core |
| DLX6 | 1750 | 7 | 96469225 | 96477225 | 0.0002 | Tumor hypermethylation in CpG island core |
| TAC1 | 6863 | 7 | 97195206 | 97203206 | 0 | Tumor hypermethylation in CpG island core |
| NPTX2 | 4885 | 7 | 98080532 | 98088532 | 0 | Tumor hypermethylation in CpG island core |
| TMEM130 | 222865 | 7 | 98301609 | 98309609 | 0.0028 | Tumor hypermethylation in CpG island core |
| ZNF655 | 79027 | 7 | 98989980 | 98997980 | 0 | Tumor hypermethylation in CpG island core |
| SAP25 | 1E+08 | 7 | 100005206 | 100013206 | 0.0001 | Tumor hypermethylation in CpG island core |
| ACTL6B | 51412 | 7 | 100088020 | 100096020 | 0.0006 | Tumor hypermethylation in CpG island core |
| RELN | 5649 | 7 | 103413199 | 103421199 | 0 | Tumor hypermethylation in CpG island core |
| LHFPL3 | 375612 | 7 | 103752339 | 103760339 | 0 | Tumor hypermethylation in CpG island core |
| LAMB1 | 3912 | 7 | 107427040 | 107435040 | 0.0009 | Tumor hypermethylation in CpG island core |
| CAV2 | 858 | 7 | 115922679 | 115930679 | 0.0008 | Tumor hypermethylation in CpG island core |
| WNT2 | 7472 | 7 | 116746579 | 116754579 | 0 | Tumor hypermethylation in CpG island core |
| CTTNBP2 | 83992 | 7 | 117296797 | 117304797 | 0.0004 | Tumor hypermethylation in CpG island core |
| WNT16 | 51384 | 7 | 120752325 | 120760325 | 0.0054 | Tumor hypermethylation in CpG island core |
| PTPRZ1 | 5803 | 7 | 121296394 | 121304394 | 0 | Tumor hypermethylation in CpG island core |
| FEZF1 | 389549 | 7 | 121727801 | 121735801 | 0 | Tumor hypermethylation in CpG island core |
| TMEM229A | 730130 | 7 | 123456759 | 123464759 | 0.0006 | Tumor hypermethylation in CpG island core |
| GRM8 | 2918 | 7 | 126675664 | 126683664 | 0 | Tumor hypermethylation in CpG island core |
| FLNC | 2318 | 7 | 128253718 | 128261718 | 0.0001 | Tumor hypermethylation in CpG island core |
| KCP | 375616 | 7 | 128334009 | 128342009 | 0.0022 | Tumor hypermethylation in CpG island core |
| LOC407835 | 407835 | 7 | 128549560 | 128557560 | 0.0023 | Tumor hypermethylation in CpG island core |
| SMOX | 54498 | 7 | 128611948 | 128619948 | 0 | Tumor hypermethylation in CpG island core |
| MESTIT1 | 317751 | 7 | 129914249 | 129922249 | 0 | Tumor hypermethylation in CpG island core |
| KLF14 | 136259 | 7 | 130065428 | 130073428 | 0.004 | Tumor hypermethylation in CpG island core |
| PLXNA4 | 91584 | 7 | 131907863 | 131915863 | 0.0002 | Tumor hypermethylation in CpG island core |
| AKR1B1 | 231 | 7 | 133790428 | 133798428 | 0.0001 | Tumor hypermethylation in CpG island core |
| CHRM2 | 1129 | 7 | 136200408 | 136208408 | 0 | Tumor hypermethylation in CpG island core |
| KLRG2 | 346689 | 7 | 138814997 | 138822997 | 0.0029 | Tumor hypermethylation in CpG island core |
| CNTNAP2 | 26047 | 7 | 145440385 | 145448385 | 0 | Tumor hypermethylation in CpG island core |
| RARRES2 | 5919 | 7 | 149665696 | 149673696 | 0 | Tumor hypermethylation in CpG island core |
| TMEM176B | 28959 | 7 | 150124554 | 150132554 | 0 | Tumor hypermethylation in CpG island core |
| FASTK | 10922 | 7 | 150404884 | 150412884 | 0.0081 | Tumor hypermethylation in CpG island core |
| WDR86 | 349136 | 7 | 150734057 | 150742057 | 0 | Tumor hypermethylation in CpG island core |
| DPP6 | 1804 | 7 | 153211351 | 153219351 | 0 | Tumor hypermethylation in CpG island core |
| EN2 | 2020 | 7 | 154939584 | 154947584 | 0 | Tumor hypermethylation in CpG island core |
| PTPRN2 | 5799 | 7 | 158069243 | 158077243 | 0.0001 | Tumor hypermethylation in CpG island core |
| VIPR2 | 7434 | 7 | 158626410 | 158634410 | 0 | Tumor hypermethylation in CpG island core |
| C8orf42 | 157695 | 8 | 481331 | 489331 | 0.001 | Tumor hypermethylation in CpG island core |
| MIR596 | 693181 | 8 | 1748803 | 1756803 | 0.0003 | Tumor hypermethylation in CpG island core |
| CLDN23 | 137075 | 8 | 8593075 | 8601075 | 0.0068 | Tumor hypermethylation in CpG island core |
| SOX7 | 83595 | 8 | 10621432 | 10629432 | 0.0004 | Tumor hypermethylation in CpG island core |
| XKR6 | 286046 | 8 | 11092285 | 11100285 | 0 | Tumor hypermethylation in CpG island core |
| GATA4 | 2626 | 8 | 11595125 | 11603125 | 0 | Tumor hypermethylation in CpG island core |
| SGCZ | 137868 | 8 | 15136163 | 15144163 | 0.0005 | Tumor hypermethylation in CpG island core |
| EFHA2 | 286097 | 8 | 16925117 | 16933117 | 0.0004 | Tumor hypermethylation in CpG island core |
| PSD3 | 23362 | 8 | 18911476 | 18919476 | 0.0017 | Tumor hypermethylation in CpG island core |
| LPL | 4023 | 8 | 19836861 | 19844861 | 0 | Tumor hypermethylation in CpG island core |
| GFRA2 | 2675 | 8 | 21698292 | 21706292 | 0 | Tumor hypermethylation in CpG island core |
| TNFRSF10C | 8794 | 8 | 23012378 | 23020378 | 0.0065 | Tumor hypermethylation in CpG island core |
| TNFRSF10D | 8793 | 8 | 23073485 | 23081485 | 0.0021 | Tumor hypermethylation in CpG island core |
| LOXL2 | 4017 | 8 | 23313667 | 23321667 | 0.0033 | Tumor hypermethylation in CpG island core |
| NKX2-6 | 137814 | 8 | 23615867 | 23623867 | 0 | Tumor hypermethylation in CpG island core |
| NEFM | 4741 | 8 | 24823178 | 24831178 | 0 | Tumor hypermethylation in CpG island core |
| NEFL | 4747 | 8 | 24866048 | 24874048 | 0 | Tumor hypermethylation in CpG island core |
| EBF2 | 64641 | 8 | 25954309 | 25962309 | 0 | Tumor hypermethylation in CpG island core |
| PNMA2 | 10687 | 8 | 26423400 | 26431400 | 0.0021 | Tumor hypermethylation in CpG island core |
| ADRA1A | 148 | 8 | 26774839 | 26782839 | 0 | Tumor hypermethylation in CpG island core |
| NRG1 | 3084 | 8 | 31612809 | 31620809 | 0 | Tumor hypermethylation in CpG island core |
| UNC5D | 137970 | 8 | 35208516 | 35216516 | 0 | Tumor hypermethylation in CpG island core |
| GPR124 | 25960 | 8 | 37769581 | 37777581 | 0.0006 | Tumor hypermethylation in CpG island core |
| ADRB3 | 155 | 8 | 37939341 | 37947341 | 0 | Tumor hypermethylation in CpG island core |
| TACC1 | 6867 | 8 | 38759878 | 38767878 | 0.0055 | Tumor hypermethylation in CpG island core |
| SFRP1 | 6422 | 8 | 41282147 | 41290147 | 0 | Tumor hypermethylation in CpG island core |
| SNAI2 | 6591 | 8 | 49992541 | 50000541 | 0 | Tumor hypermethylation in CpG island core |
| SNTG1 | 54212 | 8 | 50983149 | 50991149 | 0.0006 | Tumor hypermethylation in CpG island core |
| FAM150A | 389658 | 8 | 53636574 | 53644574 | 0 | Tumor hypermethylation in CpG island core |
| NPBWR1 | 2831 | 8 | 54011020 | 54019020 | 0 | Tumor hypermethylation in CpG island core |
| OPRK1 | 4986 | 8 | 54322747 | 54330747 | 0.0037 | Tumor hypermethylation in CpG island core |
| RGS20 | 8601 | 8 | 54951994 | 54959994 | 0 | Tumor hypermethylation in CpG island core |
| XKR4 | 114786 | 8 | 56173570 | 56181570 | 0 | Tumor hypermethylation in CpG island core |
| LYN | 4067 | 8 | 56950939 | 56958939 | 0.0004 | Tumor hypermethylation in CpG island core |
| MOCOS | 55034 | 8 | 57185095 | 57193095 | 0.0001 | Tumor hypermethylation in CpG island core |
| PENK | 5179 | 8 | 57517836 | 57525836 | 0 | Tumor hypermethylation in CpG island core |
| C8orf71 | 26138 | 8 | 58350655 | 58358655 | 0.0001 | Tumor hypermethylation in CpG island core |
| TOX | 9760 | 8 | 60190321 | 60198321 | 0 | Tumor hypermethylation in CpG island core |
| MIR124-2 | 406908 | 8 | 65450259 | 65458259 | 0 | Tumor hypermethylation in CpG island core |
| BHLHE22 | 27319 | 8 | 65651367 | 65659367 | 0 | Tumor hypermethylation in CpG island core |
| CYP7B1 | 9420 | 8 | 65869902 | 65877902 | 0.0068 | Tumor hypermethylation in CpG island core |
| PREX2 | 80243 | 8 | 69023156 | 69031156 | 0.0001 | Tumor hypermethylation in CpG island core |
| PRDM14 | 63978 | 8 | 71142116 | 71150116 | 0 | Tumor hypermethylation in CpG island core |
| MSC | 51312 | 8 | 72915285 | 72923285 | 0 | Tumor hypermethylation in CpG island core |
| KCNB2 | 9312 | 8 | 73608179 | 73616179 | 0 | Tumor hypermethylation in CpG island core |
| LOC100192378 | 1E+08 | 8 | 77754065 | 77762065 | 0 | Tumor hypermethylation in CpG island core |
| PKIA | 5569 | 8 | 79586890 | 79594890 | 0.0003 | Tumor hypermethylation in CpG island core |
| STMN2 | 11075 | 8 | 80681934 | 80689934 | 0.0001 | Tumor hypermethylation in CpG island core |
| FABP5 | 387934 | 8 | 82351339 | 82359339 | 0.0038 | Tumor hypermethylation in CpG island core |
| CA3 | 761 | 8 | 86534307 | 86542307 | 0.0005 | Tumor hypermethylation in CpG island core |
| DCAF4L2 | 138009 | 8 | 88951412 | 88959412 | 0.0098 | Tumor hypermethylation in CpG island core |
| TMEM64 | 169200 | 8 | 91723309 | 91731309 | 0.0001 | Tumor hypermethylation in CpG island core |
| GDF6 | 392255 | 8 | 97238196 | 97246196 | 0 | Tumor hypermethylation in CpG island core |
| SDC2 | 6383 | 8 | 97571057 | 97579057 | 0 | Tumor hypermethylation in CpG island core |
| LAPTM4B | 55353 | 8 | 98852984 | 98860984 | 0.007 | Tumor hypermethylation in CpG island core |
| MATN2 | 4147 | 8 | 98946486 | 98954486 | 0.0055 | Tumor hypermethylation in CpG island core |
| C8orf47 | 203111 | 8 | 99141925 | 99149925 | 0.0053 | Tumor hypermethylation in CpG island core |
| KCNS2 | 3788 | 8 | 99504425 | 99512425 | 0.007 | Tumor hypermethylation in CpG island core |
| RGS22 | 26166 | 8 | 101183520 | 101191520 | 0.003 | Tumor hypermethylation in CpG island core |
| RIMS2 | 9699 | 8 | 104578151 | 104586151 | 0 | Tumor hypermethylation in CpG island core |
| ZFPM2 | 23414 | 8 | 106396322 | 106404322 | 0 | Tumor hypermethylation in CpG island core |
| RSPO2 | 340419 | 8 | 109161089 | 109169089 | 0 | Tumor hypermethylation in CpG island core |
| KCNV1 | 27012 | 8 | 111052135 | 111060135 | 0.0001 | Tumor hypermethylation in CpG island core |
| COL14A1 | 7373 | 8 | 121202532 | 121210532 | 0 | Tumor hypermethylation in CpG island core |
| HAS2AS | 594842 | 8 | 122716766 | 122724766 | 0.0002 | Tumor hypermethylation in CpG island core |
| ADCY8 | 114 | 8 | 132118017 | 132126017 | 0 | Tumor hypermethylation in CpG island core |
| FAM135B | 51059 | 8 | 139574247 | 139582247 | 0 | Tumor hypermethylation in CpG island core |
| KCNK9 | 51305 | 8 | 140780481 | 140788481 | 0 | Tumor hypermethylation in CpG island core |
| BAI1 | 575 | 8 | 143538378 | 143546378 | 0.0009 | Tumor hypermethylation in CpG island core |
| LY6K | 54742 | 8 | 143774530 | 143782530 | 0 | Tumor hypermethylation in CpG island core |
| LY6H | 4062 | 8 | 144309428 | 144317428 | 0.0035 | Tumor hypermethylation in CpG island core |
| LOC100130274 | 1E+08 | 8 | 144858267 | 144866267 | 0 | Tumor hypermethylation in CpG island core |
| MAPK15 | 225689 | 8 | 144866494 | 144874494 | 0.0057 | Tumor hypermethylation in CpG island core |
| KIAA1875 | 340390 | 8 | 145230616 | 145238616 | 0 | Tumor hypermethylation in CpG island core |
| C8ORFK29 | 340393 | 8 | 145545313 | 145553313 | 0.0024 | Tumor hypermethylation in CpG island core |
| MIR939 | 1E+08 | 8 | 145586253 | 145594253 | 0 | Tumor hypermethylation in CpG island core |
| SLC39A4 | 55630 | 8 | 145608725 | 145616725 | 0.0001 | Tumor hypermethylation in CpG island core |
| DMRT1 | 1761 | 9 | 827689 | 835689 | 0.0002 | Tumor hypermethylation in CpG island core |
| DMRT3 | 58524 | 9 | 962963 | 970963 | 0.0002 | Tumor hypermethylation in CpG island core |
| KCNV2 | 169522 | 9 | 2703525 | 2711525 | 0.0027 | Tumor hypermethylation in CpG island core |
| GLIS3 | 169792 | 9 | 4286035 | 4294035 | 0 | Tumor hypermethylation in CpG island core |
| UHRF2 | 115426 | 9 | 6399150 | 6407150 | 0.0064 | Tumor hypermethylation in CpG island core |
| NFIB | 4781 | 9 | 14299945 | 14307945 | 0 | Tumor hypermethylation in CpG island core |
| BNC2 | 54796 | 9 | 16856786 | 16864786 | 0 | Tumor hypermethylation in CpG island core |
| LOC554202 | 554202 | 9 | 21545697 | 21553697 | 0.009 | Tumor hypermethylation in CpG island core |
| ELAVL2 | 1993 | 9 | 23807843 | 23815843 | 0.0001 | Tumor hypermethylation in CpG island core |
| CNTFR | 1271 | 9 | 34575722 | 34583722 | 0.0001 | Tumor hypermethylation in CpG island core |
| NPR2 | 10641 | 9 | 35778405 | 35786405 | 0.0031 | Tumor hypermethylation in CpG island core |
| IGFBPL1 | 347252 | 9 | 38410444 | 38418444 | 0.0055 | Tumor hypermethylation in CpG island core |
| PRKACG | 5568 | 9 | 70814859 | 70822859 | 0.0092 | Tumor hypermethylation in CpG island core |
| RORB | 6096 | 9 | 76298071 | 76306071 | 0 | Tumor hypermethylation in CpG island core |
| PSAT1 | 29968 | 9 | 80097878 | 80105878 | 0 | Tumor hypermethylation in CpG island core |
| TLE4 | 7091 | 9 | 81372697 | 81380697 | 0 | Tumor hypermethylation in CpG island core |
| FRMD3 | 257019 | 9 | 85339168 | 85347168 | 0.0016 | Tumor hypermethylation in CpG island core |
| NTRK2 | 4915 | 9 | 86469285 | 86477285 | 0 | Tumor hypermethylation in CpG island core |
| RORA | 6095 | 9 | 93748265 | 93756265 | 0.0048 | Tumor hypermethylation in CpG island core |
| NCRNA00092 | 1E+08 | 9 | 97819858 | 97827858 | 0.0001 | Tumor hypermethylation in CpG island core |
| FOXE1 | 2304 | 9 | 99651357 | 99659357 | 0 | Tumor hypermethylation in CpG island core |
| GABBR2 | 9568 | 9 | 100507300 | 100515300 | 0 | Tumor hypermethylation in CpG island core |
| COL15A1 | 1306 | 9 | 100741958 | 100749958 | 0.0004 | Tumor hypermethylation in CpG island core |
| LPPR1 | 54886 | 9 | 102826851 | 102834851 | 0 | Tumor hypermethylation in CpG island core |
| C9orf125 | 84302 | 9 | 103285296 | 103293296 | 0 | Tumor hypermethylation in CpG island core |
| GRIN3A | 116443 | 9 | 103536683 | 103544683 | 0 | Tumor hypermethylation in CpG island core |
| ACTL7B | 10880 | 9 | 110654096 | 110662096 | 0.0056 | Tumor hypermethylation in CpG island core |
| AKAP2 | 445815 | 9 | 111846796 | 111854796 | 0.0003 | Tumor hypermethylation in CpG island core |
| SVEP1 | 79987 | 9 | 112377981 | 112385981 | 0 | Tumor hypermethylation in CpG island core |
| LPAR1 | 1902 | 9 | 112836186 | 112844186 | 0.0013 | Tumor hypermethylation in CpG island core |
| ZFP37 | 7539 | 9 | 114854817 | 114862817 | 0.0012 | Tumor hypermethylation in CpG island core |
| DBC1 | 57805 | 9 | 121167560 | 121175560 | 0 | Tumor hypermethylation in CpG island core |
| CHAT | 10044 | 9 | 129553404 | 129561404 | 0 | Tumor hypermethylation in CpG island core |
| PIP5KL1 | 138429 | 9 | 129725591 | 129733591 | 0.0041 | Tumor hypermethylation in CpG island core |
| MIR219-2 | 407003 | 9 | 130190814 | 130198814 | 0 | Tumor hypermethylation in CpG island core |
| PKN3 | 29941 | 9 | 130500622 | 130508622 | 0.0085 | Tumor hypermethylation in CpG island core |
| C9orf171 | 389799 | 9 | 134271431 | 134279431 | 0.0001 | Tumor hypermethylation in CpG island core |
| GBGT1 | 26301 | 9 | 135025122 | 135033122 | 0.0011 | Tumor hypermethylation in CpG island core |
| ABO | 28 | 9 | 135136451 | 135144451 | 0.0019 | Tumor hypermethylation in CpG island core |
| RNU6ATAC | 1E+08 | 9 | 136015507 | 136023507 | 0.004 | Tumor hypermethylation in CpG island core |
| SOHLH1 | 402381 | 9 | 137727195 | 137735195 | 0.0004 | Tumor hypermethylation in CpG island core |
| LHX3 | 8022 | 9 | 138230825 | 138238825 | 0 | Tumor hypermethylation in CpG island core |
| PTGDS | 5730 | 9 | 138987776 | 138995776 | 0.0016 | Tumor hypermethylation in CpG island core |
| ENTPD2 | 954 | 9 | 139064326 | 139072326 | 0.006 | Tumor hypermethylation in CpG island core |
| GRIN1 | 114787 | 9 | 139149429 | 139157429 | 0 | Tumor hypermethylation in CpG island core |
| CACNA1B | 774 | 9 | 139888061 | 139896061 | 0.0003 | Tumor hypermethylation in CpG island core |
| NLGN4X | 57502 | X | 6151888 | 6159888 | 0 | Tumor hypermethylation in CpG island core |
| KAL1 | 3730 | X | 8656227 | 8664227 | 0 | Tumor hypermethylation in CpG island core |
| TBL1X | 6907 | X | 9389200 | 9397200 | 0 | Tumor hypermethylation in CpG island core |
| SHROOM2 | 357 | X | 9710495 | 9718495 | 0.0049 | Tumor hypermethylation in CpG island core |
| WWC3 | 55841 | X | 9939794 | 9947794 | 0.0012 | Tumor hypermethylation in CpG island core |
| ARHGAP6 | 395 | X | 11589742 | 11597742 | 0 | Tumor hypermethylation in CpG island core |
| FRMPD4 | 9758 | X | 12062505 | 12070505 | 0.0091 | Tumor hypermethylation in CpG island core |
| PRPS2 | 5634 | X | 12715413 | 12723413 | 0.0032 | Tumor hypermethylation in CpG island core |
| TMSL3 | 7117 | X | 12899145 | 12907145 | 0.0001 | Tumor hypermethylation in CpG island core |
| RAB9A | 9367 | X | 13613261 | 13621261 | 0 | Tumor hypermethylation in CpG island core |
| GPM6B | 2824 | X | 13862752 | 13870752 | 0 | Tumor hypermethylation in CpG island core |
| RBBP7 | 5931 | X | 16794455 | 16802455 | 0.0002 | Tumor hypermethylation in CpG island core |
| NHS | 4810 | X | 17299463 | 17307463 | 0.0002 | Tumor hypermethylation in CpG island core |
| SCML1 | 6322 | X | 17661512 | 17669512 | 0.0066 | Tumor hypermethylation in CpG island core |
| BEND2 | 139105 | X | 18144945 | 18152945 | 0.001 | Tumor hypermethylation in CpG island core |
| SCML2 | 10389 | X | 18278765 | 18286765 | 0 | Tumor hypermethylation in CpG island core |
| CDKL5 | 6792 | X | 18349645 | 18357645 | 0.0001 | Tumor hypermethylation in CpG island core |
| PHKA2 | 5256 | X | 18908401 | 18916401 | 0.0006 | Tumor hypermethylation in CpG island core |
| GPR64 | 10149 | X | 19046598 | 19054598 | 0.0004 | Tumor hypermethylation in CpG island core |
| SH3KBP1 | 30011 | X | 19811665 | 19819665 | 0.0001 | Tumor hypermethylation in CpG island core |
| MAP7D2 | 256714 | X | 20041035 | 20049035 | 0.0001 | Tumor hypermethylation in CpG island core |
| RPS6KA3 | 6197 | X | 20190671 | 20198671 | 0 | Tumor hypermethylation in CpG island core |
| CNKSR2 | 22866 | X | 21298456 | 21306456 | 0.0001 | Tumor hypermethylation in CpG island core |
| KLHL34 | 257240 | X | 21582369 | 21590369 | 0 | Tumor hypermethylation in CpG island core |
| YY2 | 404281 | X | 21780523 | 21788523 | 0.0048 | Tumor hypermethylation in CpG island core |
| PTCHD1 | 139411 | X | 23258905 | 23266905 | 0.0001 | Tumor hypermethylation in CpG island core |
| ACOT9 | 23597 | X | 23667328 | 23675328 | 0.0088 | Tumor hypermethylation in CpG island core |
| APOO | 79135 | X | 23831978 | 23839978 | 0.006 | Tumor hypermethylation in CpG island core |
| NR0B1 | 190 | X | 30233416 | 30241416 | 0.0018 | Tumor hypermethylation in CpG island core |
| GK | 2710 | X | 30577396 | 30585396 | 0.0038 | Tumor hypermethylation in CpG island core |
| TMEM47 | 83604 | X | 34581326 | 34589326 | 0.0065 | Tumor hypermethylation in CpG island core |
| MID1IP1 | 58526 | X | 38544016 | 38552016 | 0.0012 | Tumor hypermethylation in CpG island core |
| LOC100132831 | 1E+08 | X | 40573393 | 40581393 | 0.0074 | Tumor hypermethylation in CpG island core |
| MAOA | 4128 | X | 43396352 | 43404352 | 0.0049 | Tumor hypermethylation in CpG island core |
| CHST7 | 56548 | X | 46314135 | 46322135 | 0 | Tumor hypermethylation in CpG island core |
| RP2 | 390916 | X | 46577290 | 46585290 | 0.0026 | Tumor hypermethylation in CpG island core |
| PHF16 | 9767 | X | 46652679 | 46660679 | 0.0043 | Tumor hypermethylation in CpG island core |
| NDUFB11 | 54539 | X | 46885039 | 46893039 | 0.0003 | Tumor hypermethylation in CpG island core |
| CDK16 | 5127 | X | 46958471 | 46966471 | 0.0032 | Tumor hypermethylation in CpG island core |
| USP11 | 8237 | X | 46973257 | 46981257 | 0.0024 | Tumor hypermethylation in CpG island core |
| SYN1 | 6853 | X | 47360200 | 47368200 | 0.004 | Tumor hypermethylation in CpG island core |
| ELK1 | 131096 | X | 47390947 | 47398947 | 0.0075 | Tumor hypermethylation in CpG island core |
| WDR13 | 64743 | X | 48337167 | 48345167 | 0.0007 | Tumor hypermethylation in CpG island core |
| SUV39H1 | 6839 | X | 48436074 | 48444074 | 0.0041 | Tumor hypermethylation in CpG island core |
| HDAC6 | 10013 | X | 48541430 | 48549430 | 0.0013 | Tumor hypermethylation in CpG island core |
| PQBP1 | 10084 | X | 48636138 | 48644138 | 0.0002 | Tumor hypermethylation in CpG island core |
| PIM2 | 11040 | X | 48657357 | 48665357 | 0.0026 | Tumor hypermethylation in CpG island core |
| OTUD5 | 55593 | X | 48695837 | 48703837 | 0.0001 | Tumor hypermethylation in CpG island core |
| TFE3 | 7030 | X | 48783934 | 48791934 | 0.0015 | Tumor hypermethylation in CpG island core |
| PLP2 | 5355 | X | 48911127 | 48919127 | 0.0003 | Tumor hypermethylation in CpG island core |
| PRICKLE3 | 4007 | X | 48925720 | 48933720 | 0.0079 | Tumor hypermethylation in CpG island core |
| PPP1R3F | 89801 | X | 49009260 | 49017260 | 0.0001 | Tumor hypermethylation in CpG island core |
| DGKK | 139189 | X | 50226477 | 50234477 | 0 | Tumor hypermethylation in CpG island core |
| SHROOM4 | 57477 | X | 50569784 | 50577784 | 0.0021 | Tumor hypermethylation in CpG island core |
| CXorf67 | 340602 | X | 51162506 | 51170506 | 0.0034 | Tumor hypermethylation in CpG island core |
| NUDT11 | 55190 | X | 51252199 | 51260199 | 0.002 | Tumor hypermethylation in CpG island core |
| TSPYL2 | 64061 | X | 53124266 | 53132266 | 0.0051 | Tumor hypermethylation in CpG island core |
| SMC1A | 8243 | X | 53462343 | 53470343 | 0 | Tumor hypermethylation in CpG island core |
| PHF8 | 23133 | X | 54083332 | 54091332 | 0.0011 | Tumor hypermethylation in CpG island core |
| WNK3 | 65267 | X | 54397163 | 54405163 | 0 | Tumor hypermethylation in CpG island core |
| FGD1 | 2245 | X | 54535324 | 54543324 | 0.0001 | Tumor hypermethylation in CpG island core |
| OPHN1 | 4983 | X | 67566024 | 67574024 | 0.0086 | Tumor hypermethylation in CpG island core |
| EFNB1 | 1947 | X | 67961564 | 67969564 | 0.0002 | Tumor hypermethylation in CpG island core |
| FAM155B | 27112 | X | 68637802 | 68645802 | 0.0001 | Tumor hypermethylation in CpG island core |
| EDA | 1896 | X | 68748635 | 68756635 | 0.0027 | Tumor hypermethylation in CpG island core |
| OTUD6A | 139562 | X | 69195065 | 69203065 | 0.0034 | Tumor hypermethylation in CpG island core |
| TAF1 | 6872 | X | 70498838 | 70506838 | 0.0013 | Tumor hypermethylation in CpG island core |
| INGX | 27160 | X | 70625329 | 70633329 | 0.0044 | Tumor hypermethylation in CpG island core |
| OGT | 8473 | X | 70665636 | 70673636 | 0.0067 | Tumor hypermethylation in CpG island core |
| ACRC | 93953 | X | 70710598 | 70718598 | 0.0082 | Tumor hypermethylation in CpG island core |
| NHSL2 | 340527 | X | 71043662 | 71051662 | 0.003 | Tumor hypermethylation in CpG island core |
| RGAG4 | 340526 | X | 71264476 | 71272476 | 0.0068 | Tumor hypermethylation in CpG island core |
| CITED1 | 4435 | X | 71438489 | 71446489 | 0 | Tumor hypermethylation in CpG island core |
| SLC16A2 | 6567 | X | 73553809 | 73561809 | 0 | Tumor hypermethylation in CpG island core |
| KIAA2022 | 340533 | X | 74058012 | 74066012 | 0.0003 | Tumor hypermethylation in CpG island core |
| POU3F4 | 5456 | X | 82645924 | 82653924 | 0.0033 | Tumor hypermethylation in CpG island core |
| PABPC5 | 140886 | X | 90572252 | 90580252 | 0.006 | Tumor hypermethylation in CpG island core |
| NAP1L3 | 4675 | X | 92811264 | 92819264 | 0.0003 | Tumor hypermethylation in CpG island core |
| DIAPH2 | 1730 | X | 95822317 | 95830317 | 0.0068 | Tumor hypermethylation in CpG island core |
| GPRASP1 | 9737 | X | 101788949 | 101796949 | 0.0018 | Tumor hypermethylation in CpG island core |
| GPRASP2 | 114928 | X | 101849995 | 101857995 | 0.0081 | Tumor hypermethylation in CpG island core |
| BHLHB9 | 80823 | X | 101883562 | 101891562 | 0.009 | Tumor hypermethylation in CpG island core |
| BEX4 | 56271 | X | 102352675 | 102360675 | 0.0061 | Tumor hypermethylation in CpG island core |
| TCEAL3 | 85012 | X | 102745489 | 102753489 | 0.0081 | Tumor hypermethylation in CpG island core |
| ESX1 | 80712 | X | 103382255 | 103390255 | 0 | Tumor hypermethylation in CpG island core |
| IL1RAPL2 | 26280 | X | 103693651 | 103701651 | 0.002 | Tumor hypermethylation in CpG island core |
| IRS4 | 8471 | X | 107862263 | 107870263 | 0 | Tumor hypermethylation in CpG island core |
| ACSL4 | 2182 | X | 108859277 | 108867277 | 0.0072 | Tumor hypermethylation in CpG island core |
| HTR2C | 3358 | X | 113720806 | 113728806 | 0 | Tumor hypermethylation in CpG island core |
| PLS3 | 5358 | X | 114697461 | 114705461 | 0 | Tumor hypermethylation in CpG island core |
| WDR44 | 54521 | X | 117360069 | 117368069 | 0.0093 | Tumor hypermethylation in CpG island core |
| DOCK11 | 139818 | X | 117509899 | 117517899 | 0.0001 | Tumor hypermethylation in CpG island core |
| IL13RA1 | 3597 | X | 117741586 | 117749586 | 0.002 | Tumor hypermethylation in CpG island core |
| LONRF3 | 79836 | X | 117988740 | 117996740 | 0 | Tumor hypermethylation in CpG island core |
| PGRMC1 | 10857 | X | 118250238 | 118258238 | 0.0014 | Tumor hypermethylation in CpG island core |
| LOC100303728 | 1E+08 | X | 118483111 | 118491111 | 0.0002 | Tumor hypermethylation in CpG island core |
| UBE2A | 7319 | X | 118588526 | 118596526 | 0.0024 | Tumor hypermethylation in CpG island core |
| NKRF | 55922 | X | 118619841 | 118627841 | 0.0031 | Tumor hypermethylation in CpG island core |
| 6-Sep | 23157 | X | 118707361 | 118715361 | 0 | Tumor hypermethylation in CpG island core |
| ANKRD58 | 347454 | X | 118772603 | 118780603 | 0.0001 | Tumor hypermethylation in CpG island core |
| RPL39 | 6170 | X | 118805634 | 118813634 | 0.0024 | Tumor hypermethylation in CpG island core |
| RNF113A | 7737 | X | 118885819 | 118893819 | 0.0001 | Tumor hypermethylation in CpG island core |
| THOC2 | 57187 | X | 122690585 | 122698585 | 0.0001 | Tumor hypermethylation in CpG island core |
| STAG2 | 10735 | X | 122918155 | 122926155 | 0 | Tumor hypermethylation in CpG island core |
| DCAF12L1 | 139170 | X | 125510523 | 125518523 | 0.0058 | Tumor hypermethylation in CpG island core |
| APLN | 8862 | X | 128612595 | 128620595 | 0.007 | Tumor hypermethylation in CpG island core |
| ZDHHC9 | 51114 | X | 128801149 | 128809149 | 0.0022 | Tumor hypermethylation in CpG island core |
| BCORL1 | 63035 | X | 128940349 | 128948349 | 0 | Tumor hypermethylation in CpG island core |
| ELF4 | 2000 | X | 129068369 | 129076369 | 0.0001 | Tumor hypermethylation in CpG island core |
| RAB33A | 9363 | X | 129129453 | 129137453 | 0.0006 | Tumor hypermethylation in CpG island core |
| RAP2C | 57826 | X | 131175870 | 131183870 | 0.0081 | Tumor hypermethylation in CpG island core |
| GPC4 | 2239 | X | 132372871 | 132380871 | 0 | Tumor hypermethylation in CpG island core |
| GPC3 | 2719 | X | 132943339 | 132951339 | 0.0062 | Tumor hypermethylation in CpG island core |
| FHL1 | 27259 | X | 135053224 | 135061224 | 0.0003 | Tumor hypermethylation in CpG island core |
| RBMX | 27316 | X | 135786605 | 135794605 | 0.0076 | Tumor hypermethylation in CpG island core |
| ZIC3 | 7547 | X | 136472011 | 136480011 | 0 | Tumor hypermethylation in CpG island core |
| SOX3 | 6658 | X | 139410891 | 139418891 | 0 | Tumor hypermethylation in CpG island core |
| SLITRK4 | 139065 | X | 142546685 | 142554685 | 0.0014 | Tumor hypermethylation in CpG island core |
| SLITRK2 | 84631 | X | 144703038 | 144711038 | 0.0045 | Tumor hypermethylation in CpG island core |
| AFF2 | 2334 | X | 147385830 | 147393830 | 0.0014 | Tumor hypermethylation in CpG island core |
| CD99L2 | 83692 | X | 149813837 | 149821837 | 0.0008 | Tumor hypermethylation in CpG island core |
| HMGB3 | 3149 | X | 149898420 | 149906420 | 0.0031 | Tumor hypermethylation in CpG island core |
| GPR50 | 9248 | X | 150091713 | 150099713 | 0.0007 | Tumor hypermethylation in CpG island core |
| GABRE | 2564 | X | 150889807 | 150897807 | 0.0095 | Tumor hypermethylation in CpG island core |
| FAM58A | 92002 | X | 152513826 | 152521826 | 0.0069 | Tumor hypermethylation in CpG island core |
| DUSP9 | 1852 | X | 152557090 | 152565090 | 0.0004 | Tumor hypermethylation in CpG island core |
| PNCK | 139728 | X | 152587937 | 152595937 | 0.0015 | Tumor hypermethylation in CpG island core |
| BCAP31 | 10134 | X | 152638776 | 152646776 | 0 | Tumor hypermethylation in CpG island core |
| IDH3G | 3421 | X | 152709161 | 152717161 | 0 | Tumor hypermethylation in CpG island core |
| PDZD4 | 57595 | X | 152745197 | 152753197 | 0 | Tumor hypermethylation in CpG island core |
| HCFC1 | 3054 | X | 152886013 | 152894013 | 0 | Tumor hypermethylation in CpG island core |
| IRAK1 | 3654 | X | 152934536 | 152942536 | 0.0053 | Tumor hypermethylation in CpG island core |
| TKTL1 | 8277 | X | 153173220 | 153181220 | 0.0037 | Tumor hypermethylation in CpG island core |
| EMD | 2010 | X | 153256790 | 153264790 | 0 | Tumor hypermethylation in CpG island core |
| RPL10 | 6138 | X | 153275764 | 153283764 | 0 | Tumor hypermethylation in CpG island core |
| DNASE1L1 | 1774 | X | 153289621 | 153297621 | 0.0034 | Tumor hypermethylation in CpG island core |
| GDI1 | 2664 | X | 153314452 | 153322452 | 0.003 | Tumor hypermethylation in CpG island core |
| LAGE3 | 8270 | X | 153356790 | 153364790 | 0.0064 | Tumor hypermethylation in CpG island core |
| G6PD | 8266 | X | 153364126 | 153372126 | 0.001 | Tumor hypermethylation in CpG island core |
| SLC10A3 | 8273 | X | 153368196 | 153376196 | 0.0097 | Tumor hypermethylation in CpG island core |
| FAM3A | 60343 | X | 153393760 | 153401760 | 0.0006 | Tumor hypermethylation in CpG island core |
| G6PD | 8266 | X | 153424427 | 153432427 | 0.0001 | Tumor hypermethylation in CpG island core |
| NOC2L | 26155 | 1 | 880542 | 888542 | 0 | Tumor hypermethylation in CpG island left shore |
| MMP23B | 8510 | 1 | 1553422 | 1561422 | 0 | Tumor hypermethylation in CpG island left shore |
| PANK4 | 55229 | 1 | 2443895 | 2451895 | 0 | Tumor hypermethylation in CpG island left shore |
| FOXD2 | 2306 | 1 | 47670275 | 47678275 | 0 | Tumor hypermethylation in CpG island left shore |
| POLR3GL | 84265 | 1 | 144177744 | 144185744 | 0 | Tumor hypermethylation in CpG island left shore |
| LIX1L | 128077 | 1 | 144184441 | 144192441 | 0 | Tumor hypermethylation in CpG island left shore |
| EFNA1 | 1942 | 1 | 153362972 | 153370972 | 0.002 | Tumor hypermethylation in CpG island left shore |
| NTRK1 | 4914 | 1 | 155093294 | 155101294 | 0.002 | Tumor hypermethylation in CpG island left shore |
| PFKFB2 | 5208 | 1 | 205289242 | 205297242 | 0.0004 | Tumor hypermethylation in CpG island left shore |
| ZNF678 | 339500 | 1 | 225813842 | 225821842 | 0 | Tumor hypermethylation in CpG island left shore |
| C10orf140 | 387640 | 10 | 21850617 | 21858617 | 0.0001 | Tumor hypermethylation in CpG island left shore |
| LOC220930 | 220930 | 10 | 31644030 | 31652030 | 0.0007 | Tumor hypermethylation in CpG island left shore |
| NRP1 | 8829 | 10 | 33659839 | 33667839 | 0.0002 | Tumor hypermethylation in CpG island left shore |
| CHAT | 10044 | 10 | 50487159 | 50495159 | 0 | Tumor hypermethylation in CpG island left shore |
| BMS1P4 | 729096 | 10 | 75156278 | 75164278 | 0 | Tumor hypermethylation in CpG island left shore |
| BUB3 | 9184 | 10 | 124899749 | 124907749 | 0 | Tumor hypermethylation in CpG island left shore |
| SIGIRR | 59307 | 11 | 403397 | 411397 | 0 | Tumor hypermethylation in CpG island left shore |
| CD151 | 977 | 11 | 818951 | 826951 | 0 | Tumor hypermethylation in CpG island left shore |
| MIR675 | 1E+08 | 11 | 1970637 | 1978637 | 0 | Tumor hypermethylation in CpG island left shore |
| CALCA | 796 | 11 | 14946408 | 14954408 | 0 | Tumor hypermethylation in CpG island left shore |
| PAX6 | 5080 | 11 | 31785455 | 31793455 | 0 | Tumor hypermethylation in CpG island left shore |
| MIR129-2 | 406918 | 11 | 43555519 | 43563519 | 0 | Tumor hypermethylation in CpG island left shore |
| MDK | 10305 | 11 | 46355794 | 46363794 | 0.0006 | Tumor hypermethylation in CpG island left shore |
| EEF1G | 1937 | 11 | 62094036 | 62102036 | 0.0096 | Tumor hypermethylation in CpG island left shore |
| BSCL2 | 26580 | 11 | 62226363 | 62234363 | 0.0002 | Tumor hypermethylation in CpG island left shore |
| RCOR2 | 283248 | 11 | 63436892 | 63444892 | 0.0001 | Tumor hypermethylation in CpG island left shore |
| TM7SF2 | 7108 | 11 | 64631916 | 64639916 | 0.0001 | Tumor hypermethylation in CpG island left shore |
| FIBP | 9158 | 11 | 65408586 | 65416586 | 0.0087 | Tumor hypermethylation in CpG island left shore |
| CCND1 | 595 | 11 | 69161053 | 69169053 | 0.0001 | Tumor hypermethylation in CpG island left shore |
| C11orf88 | 399949 | 11 | 110886719 | 110894719 | 0.0012 | Tumor hypermethylation in CpG island left shore |
| BARX2 | 8538 | 11 | 128747090 | 128755090 | 0 | Tumor hypermethylation in CpG island left shore |
| GRIN2B | 2904 | 12 | 14020289 | 14028289 | 0.0044 | Tumor hypermethylation in CpG island left shore |
| HOXC5 | 3222 | 12 | 52709098 | 52717098 | 0 | Tumor hypermethylation in CpG island left shore |
| SHMT2 | 6472 | 12 | 55906094 | 55914094 | 0.0032 | Tumor hypermethylation in CpG island left shore |
| DCTN2 | 10540 | 12 | 56223245 | 56231245 | 0.007 | Tumor hypermethylation in CpG island left shore |
| PUS1 | 80324 | 12 | 130975697 | 130983697 | 0.0041 | Tumor hypermethylation in CpG island left shore |
| CDKN3 | 1033 | 14 | 53929422 | 53937422 | 0.0003 | Tumor hypermethylation in CpG island left shore |
| C14orf162 | 56936 | 14 | 69103671 | 69111671 | 0.0002 | Tumor hypermethylation in CpG island left shore |
| KIF26A | 26153 | 14 | 103670812 | 103678812 | 0 | Tumor hypermethylation in CpG island left shore |
| DLL4 | 54567 | 15 | 39004838 | 39012838 | 0.0092 | Tumor hypermethylation in CpG island left shore |
| RPP25 | 54913 | 15 | 73032828 | 73040828 | 0.0018 | Tumor hypermethylation in CpG island left shore |
| DECR2 | 26063 | 16 | 387858 | 395858 | 0.0032 | Tumor hypermethylation in CpG island left shore |
| MSLNL | 401827 | 16 | 768927 | 776927 | 0.0001 | Tumor hypermethylation in CpG island left shore |
| SPSB3 | 90864 | 16 | 1768582 | 1776582 | 0.0002 | Tumor hypermethylation in CpG island left shore |
| GFER | 2671 | 16 | 1970150 | 1978150 | 0.0004 | Tumor hypermethylation in CpG island left shore |
| E4F1 | 1877 | 16 | 2209567 | 2217567 | 0.0088 | Tumor hypermethylation in CpG island left shore |
| DNASE1L2 | 1775 | 16 | 2222468 | 2230468 | 0 | Tumor hypermethylation in CpG island left shore |
| PAQR4 | 124222 | 16 | 2955342 | 2963342 | 0 | Tumor hypermethylation in CpG island left shore |
| TNFRSF12A | 51330 | 16 | 3006313 | 3014313 | 0.0004 | Tumor hypermethylation in CpG island left shore |
| ZNF668 | 79759 | 16 | 30989005 | 30997005 | 0.0051 | Tumor hypermethylation in CpG island left shore |
| SALL1 | 6299 | 16 | 49738009 | 49746009 | 0 | Tumor hypermethylation in CpG island left shore |
| IRX3 | 79191 | 16 | 52873879 | 52881879 | 0 | Tumor hypermethylation in CpG island left shore |
| LOC283856 | 283856 | 16 | 54778507 | 54786507 | 0.0029 | Tumor hypermethylation in CpG island left shore |
| TRADD | 8717 | 16 | 65747313 | 65755313 | 0 | Tumor hypermethylation in CpG island left shore |
| FOXF1 | 2294 | 16 | 85097633 | 85105633 | 0 | Tumor hypermethylation in CpG island left shore |
| LOC100129637 | 1E+08 | 16 | 86292791 | 86300791 | 0 | Tumor hypermethylation in CpG island left shore |
| CYBA | 1535 | 16 | 87240958 | 87248958 | 0.0047 | Tumor hypermethylation in CpG island left shore |
| MIR132 | 406921 | 17 | 1896052 | 1904052 | 0.0026 | Tumor hypermethylation in CpG island left shore |
| GLTPD2 | 388323 | 17 | 4634993 | 4642993 | 0.0002 | Tumor hypermethylation in CpG island left shore |
| FGF11 | 2256 | 17 | 7279412 | 7287412 | 0.0014 | Tumor hypermethylation in CpG island left shore |
| RPL23A | 6147 | 17 | 24067126 | 24075126 | 0 | Tumor hypermethylation in CpG island left shore |
| ANKRD13B | 124930 | 17 | 24940652 | 24948652 | 0.0004 | Tumor hypermethylation in CpG island left shore |
| LASP1 | 3927 | 17 | 34275637 | 34283637 | 0.0033 | Tumor hypermethylation in CpG island left shore |
| HEXIM1 | 10614 | 17 | 40576466 | 40584466 | 0 | Tumor hypermethylation in CpG island left shore |
| WNT3 | 7473 | 17 | 42247081 | 42255081 | 0.0004 | Tumor hypermethylation in CpG island left shore |
| HOXB5 | 3215 | 17 | 44022102 | 44030102 | 0.0038 | Tumor hypermethylation in CpG island left shore |
| SRP68 | 6730 | 17 | 71576202 | 71584202 | 0 | Tumor hypermethylation in CpG island left shore |
| CBX8 | 57332 | 17 | 75381485 | 75389485 | 0 | Tumor hypermethylation in CpG island left shore |
| CBX4 | 8535 | 17 | 75423808 | 75431808 | 0 | Tumor hypermethylation in CpG island left shore |
| BAHCC1 | 57597 | 17 | 76984134 | 76992134 | 0 | Tumor hypermethylation in CpG island left shore |
| PCYT2 | 5833 | 17 | 77458586 | 77466586 | 0.0006 | Tumor hypermethylation in CpG island left shore |
| UTS2R | 2837 | 17 | 77921489 | 77929489 | 0 | Tumor hypermethylation in CpG island left shore |
| TMEM200C | 645369 | 18 | 5878103 | 5886103 | 0 | Tumor hypermethylation in CpG island left shore |
| SALL3 | 27164 | 18 | 74837262 | 74845262 | 0 | Tumor hypermethylation in CpG island left shore |
| PPAP2C | 8612 | 19 | 238435 | 246435 | 0.0014 | Tumor hypermethylation in CpG island left shore |
| PTBP1 | 5725 | 19 | 744391 | 752391 | 0.0029 | Tumor hypermethylation in CpG island left shore |
| SLC25A23 | 79085 | 19 | 1397147 | 1405147 | 0.0001 | Tumor hypermethylation in CpG island left shore |
| MIR1909 | 1E+08 | 19 | 1763237 | 1771237 | 0 | Tumor hypermethylation in CpG island left shore |
| C19orf77 | 284422 | 19 | 3427540 | 3435540 | 0 | Tumor hypermethylation in CpG island left shore |
| MIR181C | 406957 | 19 | 13842512 | 13850512 | 0.0041 | Tumor hypermethylation in CpG island left shore |
| NANOS3 | 342977 | 19 | 13844949 | 13852949 | 0.004 | Tumor hypermethylation in CpG island left shore |
| USE1 | 65264 | 19 | 17183154 | 17191154 | 0.0002 | Tumor hypermethylation in CpG island left shore |
| COPE | 11316 | 19 | 18887199 | 18895199 | 0.0023 | Tumor hypermethylation in CpG island left shore |
| C19orf55 | 148137 | 19 | 40936883 | 40944883 | 0 | Tumor hypermethylation in CpG island left shore |
| ZNF540 | 163255 | 19 | 42730147 | 42738147 | 0.0008 | Tumor hypermethylation in CpG island left shore |
| RTN2 | 6253 | 19 | 50688151 | 50696151 | 0.0033 | Tumor hypermethylation in CpG island left shore |
| FBXO46 | 23403 | 19 | 50921991 | 50929991 | 0.002 | Tumor hypermethylation in CpG island left shore |
| MIR935 | 1E+08 | 19 | 59173372 | 59181372 | 0.0001 | Tumor hypermethylation in CpG island left shore |
| NAT14 | 57106 | 19 | 60684405 | 60692405 | 0 | Tumor hypermethylation in CpG island left shore |
| U2AF2 | 11338 | 19 | 60853227 | 60861227 | 0.002 | Tumor hypermethylation in CpG island left shore |
| TCF3 | 83439 | 2 | 85210244 | 85218244 | 0.0059 | Tumor hypermethylation in CpG island left shore |
| BCL2L11 | 10018 | 2 | 111590961 | 111598961 | 0.0005 | Tumor hypermethylation in CpG island left shore |
| CCDC115 | 84317 | 2 | 130812392 | 130820392 | 0.0029 | Tumor hypermethylation in CpG island left shore |
| GPR148 | 344561 | 2 | 131199112 | 131207112 | 0.0006 | Tumor hypermethylation in CpG island left shore |
| SATB2 | 23314 | 2 | 200029500 | 200037500 | 0 | Tumor hypermethylation in CpG island left shore |
| CHRND | 1144 | 2 | 233095165 | 233103165 | 0.0001 | Tumor hypermethylation in CpG island left shore |
| ESPNL | 339768 | 2 | 238669689 | 238677689 | 0.0082 | Tumor hypermethylation in CpG island left shore |
| PROKR2 | 128674 | 20 | 5239015 | 5247015 | 0 | Tumor hypermethylation in CpG island left shore |
| NKX2-2 | 4821 | 20 | 21438664 | 21446664 | 0 | Tumor hypermethylation in CpG island left shore |
| MAFB | 9935 | 20 | 38747290 | 38755290 | 0 | Tumor hypermethylation in CpG island left shore |
| TFAP2C | 7022 | 20 | 54633764 | 54641764 | 0 | Tumor hypermethylation in CpG island left shore |
| DIDO1 | 11083 | 20 | 61024290 | 61032290 | 0.001 | Tumor hypermethylation in CpG island left shore |
| PPDPF | 79144 | 20 | 61618576 | 61626576 | 0.0004 | Tumor hypermethylation in CpG island left shore |
| SIM2 | 6493 | 21 | 36989860 | 36997860 | 0 | Tumor hypermethylation in CpG island left shore |
| LZTR1 | 8216 | 22 | 19662557 | 19670557 | 0.0011 | Tumor hypermethylation in CpG island left shore |
| ALG12 | 79087 | 22 | 48694110 | 48702110 | 0.0012 | Tumor hypermethylation in CpG island left shore |
| SELO | 83642 | 22 | 48977534 | 48985534 | 0.0001 | Tumor hypermethylation in CpG island left shore |
| NCAPH2 | 29781 | 22 | 49289510 | 49297510 | 0.0001 | Tumor hypermethylation in CpG island left shore |
| CNTN4 | 152330 | 3 | 2113246 | 2121246 | 0.0001 | Tumor hypermethylation in CpG island left shore |
| RASSF1 | 11186 | 3 | 50345899 | 50353899 | 0 | Tumor hypermethylation in CpG island left shore |
| RSRC1 | 51319 | 3 | 159306585 | 159314585 | 0.0038 | Tumor hypermethylation in CpG island left shore |
| NLGN1 | 22871 | 3 | 174594937 | 174602937 | 0 | Tumor hypermethylation in CpG island left shore |
| ZNF141 | 7700 | 4 | 317595 | 325595 | 0 | Tumor hypermethylation in CpG island left shore |
| EVC | 2121 | 4 | 5759824 | 5767824 | 0.0018 | Tumor hypermethylation in CpG island left shore |
| CRMP1 | 1400 | 4 | 5937216 | 5945216 | 0 | Tumor hypermethylation in CpG island left shore |
| NKX3-2 | 579 | 4 | 13151212 | 13159212 | 0 | Tumor hypermethylation in CpG island left shore |
| NKX6-1 | 4825 | 4 | 85634411 | 85642411 | 0 | Tumor hypermethylation in CpG island left shore |
| LEF1 | 51176 | 4 | 109305561 | 109313561 | 0.0001 | Tumor hypermethylation in CpG island left shore |
| PCDH19 | 57575 | 4 | 134285919 | 134293919 | 0 | Tumor hypermethylation in CpG island left shore |
| NBLA00301 | 79804 | 4 | 174684183 | 174692183 | 0.0001 | Tumor hypermethylation in CpG island left shore |
| FLJ42709 | 441094 | 5 | 92928255 | 92936255 | 0.0046 | Tumor hypermethylation in CpG island left shore |
| NR2F1 | 7025 | 5 | 92940798 | 92948798 | 0 | Tumor hypermethylation in CpG island left shore |
| MSX2 | 4488 | 5 | 174080180 | 174088180 | 0.0071 | Tumor hypermethylation in CpG island left shore |
| TRIM41 | 90933 | 5 | 180578911 | 180586911 | 0 | Tumor hypermethylation in CpG island left shore |
| TUBB2B | 347733 | 6 | 3168967 | 3176967 | 0.0001 | Tumor hypermethylation in CpG island left shore |
| TFAP2A | 7020 | 6 | 10516593 | 10524593 | 0 | Tumor hypermethylation in CpG island left shore |
| TMEM14C | 51522 | 6 | 10827133 | 10835133 | 0.0049 | Tumor hypermethylation in CpG island left shore |
| SYCP2L | 221711 | 6 | 10991049 | 10999049 | 0.0001 | Tumor hypermethylation in CpG island left shore |
| HIST1H3A | 8350 | 6 | 26124696 | 26132696 | 0.0007 | Tumor hypermethylation in CpG island left shore |
| HIST1H4G | 8369 | 6 | 26351184 | 26359184 | 0.0038 | Tumor hypermethylation in CpG island left shore |
| HIST1H2BO | 8348 | 6 | 27965181 | 27973181 | 0.0029 | Tumor hypermethylation in CpG island left shore |
| LY6G5C | 80741 | 6 | 31752129 | 31760129 | 0.0007 | Tumor hypermethylation in CpG island left shore |
| EHMT2 | 10919 | 6 | 31969443 | 31977443 | 0.0091 | Tumor hypermethylation in CpG island left shore |
| POU3F2 | 5454 | 6 | 99385300 | 99393300 | 0 | Tumor hypermethylation in CpG island left shore |
| EPDR1 | 54749 | 7 | 37922687 | 37930687 | 0.0065 | Tumor hypermethylation in CpG island left shore |
| POU6F2 | 11281 | 7 | 38980133 | 38988133 | 0.0077 | Tumor hypermethylation in CpG island left shore |
| ATP5J2 | 9551 | 7 | 98897744 | 98905744 | 0.0022 | Tumor hypermethylation in CpG island left shore |
| LOC157627 | 157627 | 8 | 9794249 | 9802249 | 0 | Tumor hypermethylation in CpG island left shore |
| MIR124-1 | 406907 | 8 | 9794392 | 9802392 | 0 | Tumor hypermethylation in CpG island left shore |
| RALYL | 138046 | 8 | 85255654 | 85263654 | 0 | Tumor hypermethylation in CpG island left shore |
| SHARPIN | 81858 | 8 | 145227128 | 145235128 | 0.0013 | Tumor hypermethylation in CpG island left shore |
| PAX5 | 5079 | 9 | 37020476 | 37028476 | 0 | Tumor hypermethylation in CpG island left shore |
| ANKRD19 | 138649 | 9 | 94607713 | 94615713 | 0.0057 | Tumor hypermethylation in CpG island left shore |
| MIR24-1 | 407012 | 9 | 96884123 | 96892123 | 0.0027 | Tumor hypermethylation in CpG island left shore |
| LMX1B | 4010 | 9 | 128412568 | 128420568 | 0.0016 | Tumor hypermethylation in CpG island left shore |
| PRDM12 | 59335 | 9 | 132525801 | 132533801 | 0 | Tumor hypermethylation in CpG island left shore |
| BARHL1 | 56751 | 9 | 134443813 | 134451813 | 0 | Tumor hypermethylation in CpG island left shore |
| C9orf116 | 138162 | 9 | 137527582 | 137535582 | 0.0062 | Tumor hypermethylation in CpG island left shore |
| KCNT1 | 57582 | 9 | 137729851 | 137737851 | 0.0004 | Tumor hypermethylation in CpG island left shore |
| ABCA2 | 20 | 9 | 139038561 | 139046561 | 0.0005 | Tumor hypermethylation in CpG island left shore |
| NELF | 26012 | 9 | 139469607 | 139477607 | 0 | Tumor hypermethylation in CpG island left shore |
| MIR106A | 406899 | X | 133127974 | 133135974 | 0 | Tumor hypermethylation in CpG island left shore |
| FAM132A | 388581 | 1 | 1167965 | 1175965 | 0 | Tumor hypermethylation in CpG island right shore |
| LOC441869 | 441869 | 1 | 1342513 | 1350513 | 0.0013 | Tumor hypermethylation in CpG island right shore |
| SKI | 6497 | 1 | 2145993 | 2153993 | 0 | Tumor hypermethylation in CpG island right shore |
| RNF207 | 388591 | 1 | 6184775 | 6192775 | 0.0012 | Tumor hypermethylation in CpG island right shore |
| IGSF21 | 84966 | 1 | 18302826 | 18310826 | 0.0003 | Tumor hypermethylation in CpG island right shore |
| NBL1 | 4681 | 1 | 19839394 | 19847394 | 0.0014 | Tumor hypermethylation in CpG island right shore |
| LCK | 3932 | 1 | 32508298 | 32516298 | 0.0013 | Tumor hypermethylation in CpG island right shore |
| FAM176B | 55194 | 1 | 36558342 | 36566342 | 0 | Tumor hypermethylation in CpG island right shore |
| B4GALT2 | 8704 | 1 | 44213452 | 44221452 | 0.0045 | Tumor hypermethylation in CpG island right shore |
| RNF220 | 55182 | 1 | 44639546 | 44647546 | 0 | Tumor hypermethylation in CpG island right shore |
| BEST4 | 266675 | 1 | 45022013 | 45030013 | 0.0005 | Tumor hypermethylation in CpG island right shore |
| LHX8 | 431707 | 1 | 75362706 | 75370706 | 0 | Tumor hypermethylation in CpG island right shore |
| CELSR2 | 1952 | 1 | 109590163 | 109598163 | 0.0042 | Tumor hypermethylation in CpG island right shore |
| ALX3 | 257 | 1 | 110410845 | 110418845 | 0 | Tumor hypermethylation in CpG island right shore |
| NHLH2 | 4808 | 1 | 116180856 | 116188856 | 0 | Tumor hypermethylation in CpG island right shore |
| TBX15 | 6913 | 1 | 119329702 | 119337702 | 0 | Tumor hypermethylation in CpG island right shore |
| SEC22B | 9554 | 1 | 143803763 | 143811763 | 0 | Tumor hypermethylation in CpG island right shore |
| C2CD4D | 1E+08 | 1 | 150075657 | 150083657 | 0.001 | Tumor hypermethylation in CpG island right shore |
| S100A6 | 6277 | 1 | 151771341 | 151779341 | 0.0009 | Tumor hypermethylation in CpG island right shore |
| C1orf104 | 284618 | 1 | 153556562 | 153564562 | 0 | Tumor hypermethylation in CpG island right shore |
| PAQR6 | 79957 | 1 | 154480416 | 154488416 | 0.0026 | Tumor hypermethylation in CpG island right shore |
| C1orf92 | 149499 | 1 | 155153047 | 155161047 | 0.0007 | Tumor hypermethylation in CpG island right shore |
| LHX4 | 89884 | 1 | 178462064 | 178470064 | 0.0006 | Tumor hypermethylation in CpG island right shore |
| ITPKB | 3707 | 1 | 224989499 | 224997499 | 0 | Tumor hypermethylation in CpG island right shore |
| JMJD4 | 65094 | 1 | 225985735 | 225993735 | 0.0093 | Tumor hypermethylation in CpG island right shore |
| DUSP5P | 574029 | 1 | 226843279 | 226851279 | 0.0082 | Tumor hypermethylation in CpG island right shore |
| ACTA1 | 58 | 1 | 227632466 | 227640466 | 0 | Tumor hypermethylation in CpG island right shore |
| KLF6 | 1316 | 10 | 3813473 | 3821473 | 0.0038 | Tumor hypermethylation in CpG island right shore |
| FLJ45983 | 399717 | 10 | 8131453 | 8139453 | 0 | Tumor hypermethylation in CpG island right shore |
| C1QL3 | 389941 | 10 | 16600010 | 16608010 | 0.0091 | Tumor hypermethylation in CpG island right shore |
| MKX | 283078 | 10 | 28070784 | 28078784 | 0 | Tumor hypermethylation in CpG island right shore |
| CHAT | 10044 | 10 | 50483146 | 50491146 | 0 | Tumor hypermethylation in CpG island right shore |
| C10orf116 | 10974 | 10 | 88714167 | 88722167 | 0.0001 | Tumor hypermethylation in CpG island right shore |
| HHEX | 3087 | 10 | 94435660 | 94443660 | 0.0017 | Tumor hypermethylation in CpG island right shore |
| CYP26A1 | 1592 | 10 | 94819221 | 94827221 | 0.0009 | Tumor hypermethylation in CpG island right shore |
| HPSE2 | 60495 | 10 | 100981622 | 100989622 | 0 | Tumor hypermethylation in CpG island right shore |
| TLX1 | 3195 | 10 | 102877050 | 102885050 | 0 | Tumor hypermethylation in CpG island right shore |
| LBX1 | 10660 | 10 | 102974707 | 102982707 | 0 | Tumor hypermethylation in CpG island right shore |
| KCNIP2 | 30819 | 10 | 103589667 | 103597667 | 0.0009 | Tumor hypermethylation in CpG island right shore |
| ADRA2A | 150 | 10 | 112822779 | 112830779 | 0.0068 | Tumor hypermethylation in CpG island right shore |
| MMP21 | 118856 | 10 | 127450380 | 127458380 | 0.009 | Tumor hypermethylation in CpG island right shore |
| ADAM8 | 101 | 10 | 134936397 | 134944397 | 0.002 | Tumor hypermethylation in CpG island right shore |
| NLRP6 | 171389 | 11 | 264569 | 272569 | 0.0001 | Tumor hypermethylation in CpG island right shore |
| ATHL1 | 80162 | 11 | 275137 | 283137 | 0.0019 | Tumor hypermethylation in CpG island right shore |
| RNH1 | 284203 | 11 | 492821 | 500821 | 0 | Tumor hypermethylation in CpG island right shore |
| HRAS | 3265 | 11 | 521550 | 529550 | 0 | Tumor hypermethylation in CpG island right shore |
| CD81 | 975 | 11 | 2351122 | 2359122 | 0.0056 | Tumor hypermethylation in CpG island right shore |
| SPON1 | 10418 | 11 | 13936489 | 13944489 | 0.003 | Tumor hypermethylation in CpG island right shore |
| TRMT112 | 51504 | 11 | 63837609 | 63845609 | 0.0007 | Tumor hypermethylation in CpG island right shore |
| PCNXL3 | 399909 | 11 | 65136358 | 65144358 | 0.0003 | Tumor hypermethylation in CpG island right shore |
| DKFZp761E198 | 91056 | 11 | 65300398 | 65308398 | 0.0016 | Tumor hypermethylation in CpG island right shore |
| TMEM151A | 256472 | 11 | 65811948 | 65819948 | 0.0001 | Tumor hypermethylation in CpG island right shore |
| RIN1 | 9610 | 11 | 65856576 | 65864576 | 0.0038 | Tumor hypermethylation in CpG island right shore |
| ZDHHC24 | 254359 | 11 | 66066247 | 66074247 | 0.0099 | Tumor hypermethylation in CpG island right shore |
| LRFN4 | 78999 | 11 | 66377451 | 66385451 | 0 | Tumor hypermethylation in CpG island right shore |
| PPP1CA | 5499 | 11 | 66921952 | 66929952 | 0.0054 | Tumor hypermethylation in CpG island right shore |
| ARHGEF17 | 9828 | 11 | 72693310 | 72701310 | 0.0024 | Tumor hypermethylation in CpG island right shore |
| ODZ4 | 26011 | 11 | 78825343 | 78833343 | 0.0018 | Tumor hypermethylation in CpG island right shore |
| PGR | 5241 | 11 | 100501754 | 100509754 | 0 | Tumor hypermethylation in CpG island right shore |
| NCAM1 | 4684 | 11 | 112333204 | 112341204 | 0.0023 | Tumor hypermethylation in CpG island right shore |
| PHLDB1 | 23187 | 11 | 117979515 | 117987515 | 0 | Tumor hypermethylation in CpG island right shore |
| BSX | 390259 | 11 | 122353589 | 122361589 | 0 | Tumor hypermethylation in CpG island right shore |
| ESAM | 90952 | 11 | 124133433 | 124141433 | 0 | Tumor hypermethylation in CpG island right shore |
| WNT1 | 7471 | 12 | 47654502 | 47662502 | 0 | Tumor hypermethylation in CpG island right shore |
| DDN | 23109 | 12 | 47675355 | 47683355 | 0.0003 | Tumor hypermethylation in CpG island right shore |
| C1QL4 | 338761 | 12 | 48013238 | 48021238 | 0.0015 | Tumor hypermethylation in CpG island right shore |
| HOXC6 | 3223 | 12 | 52704460 | 52712460 | 0 | Tumor hypermethylation in CpG island right shore |
| ARHGAP9 | 64333 | 12 | 56153995 | 56161995 | 0.0012 | Tumor hypermethylation in CpG island right shore |
| AVPR1A | 552 | 12 | 61828857 | 61836857 | 0 | Tumor hypermethylation in CpG island right shore |
| KCNC2 | 3747 | 12 | 73885778 | 73893778 | 0.0028 | Tumor hypermethylation in CpG island right shore |
| PHLDA1 | 22822 | 12 | 74707823 | 74715823 | 0.0097 | Tumor hypermethylation in CpG island right shore |
| SOCS2 | 8835 | 12 | 92483728 | 92491728 | 0 | Tumor hypermethylation in CpG island right shore |
| RFX4 | 5992 | 12 | 105497162 | 105505162 | 0 | Tumor hypermethylation in CpG island right shore |
| FICD | 11153 | 12 | 107429180 | 107437180 | 0.0038 | Tumor hypermethylation in CpG island right shore |
| TMEM233 | 387890 | 12 | 118511646 | 118519646 | 0.0001 | Tumor hypermethylation in CpG island right shore |
| DDX51 | 317781 | 12 | 131190833 | 131198833 | 0.0002 | Tumor hypermethylation in CpG island right shore |
| DLEU1 | 10301 | 13 | 49593678 | 49601678 | 0 | Tumor hypermethylation in CpG island right shore |
| PCDH17 | 27253 | 13 | 57099789 | 57107789 | 0 | Tumor hypermethylation in CpG island right shore |
| ZIC2 | 7546 | 13 | 99428319 | 99436319 | 0 | Tumor hypermethylation in CpG island right shore |
| IRS2 | 8660 | 13 | 109232915 | 109240915 | 0 | Tumor hypermethylation in CpG island right shore |
| F7 | 2155 | 13 | 112804105 | 112812105 | 0.0006 | Tumor hypermethylation in CpG island right shore |
| RASA3 | 22821 | 13 | 113912197 | 113920197 | 0.0071 | Tumor hypermethylation in CpG island right shore |
| NFATC4 | 4776 | 14 | 23901984 | 23909984 | 0.0025 | Tumor hypermethylation in CpG island right shore |
| SSTR1 | 6751 | 14 | 37742954 | 37750954 | 0 | Tumor hypermethylation in CpG island right shore |
| DCAF4 | 26094 | 14 | 72458792 | 72466792 | 0.0004 | Tumor hypermethylation in CpG island right shore |
| C14orf4 | 64207 | 14 | 76560787 | 76568787 | 0 | Tumor hypermethylation in CpG island right shore |
| KCNK10 | 54207 | 14 | 87859009 | 87867009 | 0.0004 | Tumor hypermethylation in CpG island right shore |
| PTPN21 | 11099 | 14 | 88086876 | 88094876 | 0.0029 | Tumor hypermethylation in CpG island right shore |
| ANKRD9 | 122416 | 14 | 102041881 | 102049881 | 0 | Tumor hypermethylation in CpG island right shore |
| C14orf73 | 91828 | 14 | 102632233 | 102640233 | 0.0002 | Tumor hypermethylation in CpG island right shore |
| TMEM121 | 80757 | 14 | 105059997 | 105067997 | 0.0002 | Tumor hypermethylation in CpG island right shore |
| OCA2 | 4948 | 15 | 26014053 | 26022053 | 0 | Tumor hypermethylation in CpG island right shore |
| ONECUT1 | 3175 | 15 | 50865501 | 50873501 | 0 | Tumor hypermethylation in CpG island right shore |
| MESDC1 | 59274 | 15 | 79076349 | 79084349 | 0.0072 | Tumor hypermethylation in CpG island right shore |
| MEX3B | 84206 | 15 | 80121416 | 80129416 | 0.0002 | Tumor hypermethylation in CpG island right shore |
| RCCD1 | 91433 | 15 | 89295109 | 89303109 | 0.0008 | Tumor hypermethylation in CpG island right shore |
| NR2F2 | 7026 | 15 | 94671114 | 94679114 | 0.0011 | Tumor hypermethylation in CpG island right shore |
| HBQ1 | 3049 | 16 | 166334 | 174334 | 0.0036 | Tumor hypermethylation in CpG island right shore |
| PDIA2 | 64714 | 16 | 269118 | 277118 | 0.0004 | Tumor hypermethylation in CpG island right shore |
| NME4 | 4833 | 16 | 383192 | 391192 | 0.0045 | Tumor hypermethylation in CpG island right shore |
| C16orf11 | 146325 | 16 | 546422 | 554422 | 0 | Tumor hypermethylation in CpG island right shore |
| C16orf13 | 84326 | 16 | 622348 | 630348 | 0 | Tumor hypermethylation in CpG island right shore |
| WDR24 | 84219 | 16 | 676401 | 684401 | 0.0001 | Tumor hypermethylation in CpG island right shore |
| METRN | 79006 | 16 | 701173 | 709173 | 0.0019 | Tumor hypermethylation in CpG island right shore |
| SOX8 | 30812 | 16 | 967808 | 975808 | 0 | Tumor hypermethylation in CpG island right shore |
| UBE2I | 7329 | 16 | 1295180 | 1303180 | 0.0022 | Tumor hypermethylation in CpG island right shore |
| TELO2 | 9894 | 16 | 1479352 | 1487352 | 0 | Tumor hypermethylation in CpG island right shore |
| IGFALS | 3483 | 16 | 1779735 | 1787735 | 0 | Tumor hypermethylation in CpG island right shore |
| RNF151 | 146310 | 16 | 1952875 | 1960875 | 0.0008 | Tumor hypermethylation in CpG island right shore |
| NOXO1 | 124056 | 16 | 1967185 | 1975185 | 0 | Tumor hypermethylation in CpG island right shore |
| SYNGR3 | 9143 | 16 | 1975968 | 1983968 | 0.0001 | Tumor hypermethylation in CpG island right shore |
| TRAF7 | 84231 | 16 | 2141799 | 2149799 | 0.0001 | Tumor hypermethylation in CpG island right shore |
| MLST8 | 64223 | 16 | 2191450 | 2199450 | 0.0001 | Tumor hypermethylation in CpG island right shore |
| CEMP1 | 752014 | 16 | 2517420 | 2525420 | 0.0001 | Tumor hypermethylation in CpG island right shore |
| KREMEN2 | 79412 | 16 | 2950217 | 2958217 | 0 | Tumor hypermethylation in CpG island right shore |
| SNN | 8303 | 16 | 11665801 | 11673801 | 0.0085 | Tumor hypermethylation in CpG island right shore |
| SHISA9 | 729993 | 16 | 12898977 | 12906977 | 0.004 | Tumor hypermethylation in CpG island right shore |
| SH2B1 | 25970 | 16 | 28778578 | 28786578 | 0.0021 | Tumor hypermethylation in CpG island right shore |
| CORO1A | 11151 | 16 | 30098426 | 30106426 | 0.0032 | Tumor hypermethylation in CpG island right shore |
| ZNF48 | 197407 | 16 | 30310557 | 30318557 | 0.0012 | Tumor hypermethylation in CpG island right shore |
| ZNF768 | 79724 | 16 | 30441411 | 30449411 | 0.0026 | Tumor hypermethylation in CpG island right shore |
| ZNF785 | 146540 | 16 | 30500511 | 30508511 | 0.0044 | Tumor hypermethylation in CpG island right shore |
| ZNF629 | 23361 | 16 | 30702024 | 30710024 | 0.0009 | Tumor hypermethylation in CpG island right shore |
| BCKDK | 10295 | 16 | 31023162 | 31031162 | 0.005 | Tumor hypermethylation in CpG island right shore |
| PRSS36 | 146547 | 16 | 31064916 | 31072916 | 0.0067 | Tumor hypermethylation in CpG island right shore |
| FBXL8 | 55336 | 16 | 65747391 | 65755391 | 0 | Tumor hypermethylation in CpG island right shore |
| RLTPR | 146206 | 16 | 66232530 | 66240530 | 0 | Tumor hypermethylation in CpG island right shore |
| HAS3 | 3038 | 16 | 67693660 | 67701660 | 0.0021 | Tumor hypermethylation in CpG island right shore |
| LDHD | 197257 | 16 | 73704166 | 73712166 | 0 | Tumor hypermethylation in CpG island right shore |
| CDT1 | 81620 | 16 | 87393686 | 87401686 | 0 | Tumor hypermethylation in CpG island right shore |
| CBFA2T3 | 863 | 16 | 87531109 | 87539109 | 0.007 | Tumor hypermethylation in CpG island right shore |
| C16orf81 | 283860 | 16 | 87749128 | 87757128 | 0.0009 | Tumor hypermethylation in CpG island right shore |
| MYO1C | 4643 | 17 | 1331801 | 1339801 | 0.001 | Tumor hypermethylation in CpG island right shore |
| WDR81 | 124997 | 17 | 1570583 | 1578583 | 0.0001 | Tumor hypermethylation in CpG island right shore |
| TNK1 | 8711 | 17 | 7221088 | 7229088 | 0.0002 | Tumor hypermethylation in CpG island right shore |
| TMEM102 | 284114 | 17 | 7275485 | 7283485 | 0.0003 | Tumor hypermethylation in CpG island right shore |
| LSMD1 | 84316 | 17 | 7697897 | 7705897 | 0 | Tumor hypermethylation in CpG island right shore |
| TRAPPC1 | 58485 | 17 | 7772042 | 7780042 | 0.0048 | Tumor hypermethylation in CpG island right shore |
| SDF2 | 6388 | 17 | 24009060 | 24017060 | 0.0046 | Tumor hypermethylation in CpG island right shore |
| NEUROD2 | 4761 | 17 | 35013701 | 35021701 | 0 | Tumor hypermethylation in CpG island right shore |
| STAT5A | 6776 | 17 | 37689090 | 37697090 | 0.0078 | Tumor hypermethylation in CpG island right shore |
| PTRF | 284119 | 17 | 37824864 | 37832864 | 0.0088 | Tumor hypermethylation in CpG island right shore |
| WNK4 | 65266 | 17 | 38182174 | 38190174 | 0.0031 | Tumor hypermethylation in CpG island right shore |
| SH3D20 | 201175 | 17 | 40862065 | 40870065 | 0 | Tumor hypermethylation in CpG island right shore |
| HOXB2 | 3212 | 17 | 43973392 | 43981392 | 0 | Tumor hypermethylation in CpG island right shore |
| LOC404266 | 404266 | 17 | 44020652 | 44028652 | 0.0033 | Tumor hypermethylation in CpG island right shore |
| HOXB8 | 3218 | 17 | 44043300 | 44051300 | 0 | Tumor hypermethylation in CpG island right shore |
| NGFR | 4804 | 17 | 44923653 | 44931653 | 0 | Tumor hypermethylation in CpG island right shore |
| SPATA20 | 64847 | 17 | 45975560 | 45983560 | 0.0003 | Tumor hypermethylation in CpG island right shore |
| MPO | 4353 | 17 | 53709295 | 53717295 | 0.0027 | Tumor hypermethylation in CpG island right shore |
| LOC146880 | 146880 | 17 | 60204084 | 60212084 | 0 | Tumor hypermethylation in CpG island right shore |
| USH1G | 124590 | 17 | 70426946 | 70434946 | 0.0001 | Tumor hypermethylation in CpG island right shore |
| NT5C | 30833 | 17 | 70635472 | 70643472 | 0.0064 | Tumor hypermethylation in CpG island right shore |
| GALR2 | 8811 | 17 | 71578486 | 71586486 | 0 | Tumor hypermethylation in CpG island right shore |
| FOXJ1 | 2302 | 17 | 71644975 | 71652975 | 0 | Tumor hypermethylation in CpG island right shore |
| CHMP6 | 79643 | 17 | 76576235 | 76584235 | 0.0017 | Tumor hypermethylation in CpG island right shore |
| C17orf70 | 80233 | 17 | 77125848 | 77133848 | 0.0008 | Tumor hypermethylation in CpG island right shore |
| SLC25A10 | 1468 | 17 | 77285775 | 77293775 | 0 | Tumor hypermethylation in CpG island right shore |
| ARHGDIA | 396 | 17 | 77418527 | 77426527 | 0.0007 | Tumor hypermethylation in CpG island right shore |
| THOC4 | 10189 | 17 | 77438758 | 77446758 | 0.0097 | Tumor hypermethylation in CpG island right shore |
| SLC16A3 | 9123 | 17 | 77775581 | 77783581 | 0.0002 | Tumor hypermethylation in CpG island right shore |
| FN3K | 64122 | 17 | 78282740 | 78290740 | 0.0025 | Tumor hypermethylation in CpG island right shore |
| ADCYAP1 | 116 | 18 | 890943 | 898943 | 0 | Tumor hypermethylation in CpG island right shore |
| ZNF521 | 25925 | 18 | 21182212 | 21190212 | 0 | Tumor hypermethylation in CpG island right shore |
| ONECUT2 | 9480 | 18 | 53249914 | 53257914 | 0 | Tumor hypermethylation in CpG island right shore |
| CBLN2 | 147381 | 18 | 68358703 | 68366703 | 0 | Tumor hypermethylation in CpG island right shore |
| NFATC1 | 4772 | 18 | 75252759 | 75260759 | 0.0002 | Tumor hypermethylation in CpG island right shore |
| C2CD4C | 126567 | 19 | 356170 | 364170 | 0 | Tumor hypermethylation in CpG island right shore |
| BSG | 682 | 19 | 519536 | 527536 | 0.0016 | Tumor hypermethylation in CpG island right shore |
| POLRMT | 5442 | 19 | 580568 | 588568 | 0 | Tumor hypermethylation in CpG island right shore |
| FGF22 | 27006 | 19 | 586925 | 594925 | 0.0001 | Tumor hypermethylation in CpG island right shore |
| RNF126 | 55658 | 19 | 610227 | 618227 | 0.0075 | Tumor hypermethylation in CpG island right shore |
| MED16 | 10025 | 19 | 840218 | 848218 | 0.0002 | Tumor hypermethylation in CpG island right shore |
| GRIN3B | 116444 | 19 | 947436 | 955436 | 0.0057 | Tumor hypermethylation in CpG island right shore |
| ABCA7 | 10347 | 19 | 987101 | 995101 | 0 | Tumor hypermethylation in CpG island right shore |
| POLR2E | 5434 | 19 | 1042391 | 1050391 | 0.005 | Tumor hypermethylation in CpG island right shore |
| C19orf26 | 255057 | 19 | 1184990 | 1192990 | 0 | Tumor hypermethylation in CpG island right shore |
| CIRBP | 1153 | 19 | 1216266 | 1224266 | 0.0001 | Tumor hypermethylation in CpG island right shore |
| EFNA2 | 1943 | 19 | 1233167 | 1241167 | 0 | Tumor hypermethylation in CpG island right shore |
| NDUFS7 | 374291 | 19 | 1330882 | 1338882 | 0.0097 | Tumor hypermethylation in CpG island right shore |
| GAMT | 2593 | 19 | 1348552 | 1356552 | 0.0077 | Tumor hypermethylation in CpG island right shore |
| PCSK4 | 54760 | 19 | 1437407 | 1445407 | 0 | Tumor hypermethylation in CpG island right shore |
| ADAMTSL5 | 339366 | 19 | 1460188 | 1468188 | 0.0038 | Tumor hypermethylation in CpG island right shore |
| ONECUT3 | 390874 | 19 | 1700661 | 1708661 | 0 | Tumor hypermethylation in CpG island right shore |
| KLF16 | 83855 | 19 | 1810564 | 1818564 | 0.0004 | Tumor hypermethylation in CpG island right shore |
| JSRP1 | 126306 | 19 | 2202344 | 2210344 | 0 | Tumor hypermethylation in CpG island right shore |
| OAZ1 | 4946 | 19 | 2216519 | 2224519 | 0.0042 | Tumor hypermethylation in CpG island right shore |
| GNA11 | 2767 | 19 | 3041407 | 3049407 | 0 | Tumor hypermethylation in CpG island right shore |
| NCLN | 56926 | 19 | 3132874 | 3140874 | 0 | Tumor hypermethylation in CpG island right shore |
| FZR1 | 51343 | 19 | 3453294 | 3461294 | 0.0007 | Tumor hypermethylation in CpG island right shore |
| HMG20B | 10362 | 19 | 3519942 | 3527942 | 0 | Tumor hypermethylation in CpG island right shore |
| PIP5K1C | 23396 | 19 | 3647445 | 3655445 | 0.0001 | Tumor hypermethylation in CpG island right shore |
| RAX2 | 84839 | 19 | 3719219 | 3727219 | 0 | Tumor hypermethylation in CpG island right shore |
| EEF2 | 1938 | 19 | 3932461 | 3940461 | 0.0002 | Tumor hypermethylation in CpG island right shore |
| PIAS4 | 51588 | 19 | 3954748 | 3962748 | 0.0055 | Tumor hypermethylation in CpG island right shore |
| PTPRS | 5802 | 19 | 5287814 | 5295814 | 0.0041 | Tumor hypermethylation in CpG island right shore |
| DUS3L | 56931 | 19 | 5738249 | 5746249 | 0 | Tumor hypermethylation in CpG island right shore |
| ZNF358 | 140467 | 19 | 7483003 | 7491003 | 0.003 | Tumor hypermethylation in CpG island right shore |
| CLEC4G | 339390 | 19 | 7699057 | 7707057 | 0.0012 | Tumor hypermethylation in CpG island right shore |
| EVI5L | 115704 | 19 | 7813385 | 7821385 | 0.0011 | Tumor hypermethylation in CpG island right shore |
| CTXN1 | 404217 | 19 | 7893051 | 7901051 | 0.0013 | Tumor hypermethylation in CpG island right shore |
| RAB11B | 339122 | 19 | 8357204 | 8365204 | 0.006 | Tumor hypermethylation in CpG island right shore |
| ZNF414 | 84330 | 19 | 8481048 | 8489048 | 0.0001 | Tumor hypermethylation in CpG island right shore |
| PPAN | 56342 | 19 | 10073964 | 10081964 | 0.0014 | Tumor hypermethylation in CpG island right shore |
| EIF3G | 8666 | 19 | 10087599 | 10095599 | 0.0008 | Tumor hypermethylation in CpG island right shore |
| S1PR5 | 53637 | 19 | 10485126 | 10493126 | 0 | Tumor hypermethylation in CpG island right shore |
| ZNF833 | 401898 | 19 | 11641812 | 11649812 | 0.0008 | Tumor hypermethylation in CpG island right shore |
| RAD23A | 5886 | 19 | 12913653 | 12921653 | 0.0078 | Tumor hypermethylation in CpG island right shore |
| NFIX | 4784 | 19 | 12963583 | 12971583 | 0.0002 | Tumor hypermethylation in CpG island right shore |
| DCAF15 | 90379 | 19 | 13920318 | 13928318 | 0.0021 | Tumor hypermethylation in CpG island right shore |
| PTGER1 | 5731 | 19 | 14443174 | 14451174 | 0 | Tumor hypermethylation in CpG island right shore |
| SYDE1 | 85360 | 19 | 15075213 | 15083213 | 0.0005 | Tumor hypermethylation in CpG island right shore |
| KLF2 | 10365 | 19 | 16292650 | 16300650 | 0.0049 | Tumor hypermethylation in CpG island right shore |
| ABHD8 | 79575 | 19 | 17271282 | 17279282 | 0.0013 | Tumor hypermethylation in CpG island right shore |
| GTPBP3 | 84705 | 19 | 17305355 | 17313355 | 0 | Tumor hypermethylation in CpG island right shore |
| RPL18AP3 | 390354 | 19 | 17827726 | 17835726 | 0.0097 | Tumor hypermethylation in CpG island right shore |
| ISYNA1 | 51477 | 19 | 18406111 | 18414111 | 0 | Tumor hypermethylation in CpG island right shore |
| C19orf60 | 55049 | 19 | 18556494 | 18564494 | 0 | Tumor hypermethylation in CpG island right shore |
| COMP | 1311 | 19 | 18759114 | 18767114 | 0.0001 | Tumor hypermethylation in CpG island right shore |
| MLL4 | 9757 | 19 | 40896760 | 40904760 | 0.0002 | Tumor hypermethylation in CpG island right shore |
| LRFN3 | 79414 | 19 | 41115861 | 41123861 | 0 | Tumor hypermethylation in CpG island right shore |
| POLR2I | 5438 | 19 | 41294046 | 41302046 | 0.0047 | Tumor hypermethylation in CpG island right shore |
| DPF1 | 8193 | 19 | 43402730 | 43410730 | 0.0055 | Tumor hypermethylation in CpG island right shore |
| GGN | 199720 | 19 | 43566508 | 43574508 | 0.0004 | Tumor hypermethylation in CpG island right shore |
| PLEKHG2 | 64857 | 19 | 44591589 | 44599589 | 0.0049 | Tumor hypermethylation in CpG island right shore |
| CNTD2 | 79935 | 19 | 45420437 | 45428437 | 0.0012 | Tumor hypermethylation in CpG island right shore |
| EGLN2 | 112398 | 19 | 45992887 | 46000887 | 0.0083 | Tumor hypermethylation in CpG island right shore |
| PHLDB3 | 653583 | 19 | 48696825 | 48704825 | 0.0004 | Tumor hypermethylation in CpG island right shore |
| SIX5 | 147912 | 19 | 50960337 | 50968337 | 0 | Tumor hypermethylation in CpG island right shore |
| DMPK | 1760 | 19 | 50971701 | 50979701 | 0.0001 | Tumor hypermethylation in CpG island right shore |
| IRF2BP1 | 26145 | 19 | 51077216 | 51085216 | 0.003 | Tumor hypermethylation in CpG island right shore |
| PTGIR | 5739 | 19 | 51816194 | 51824194 | 0.0046 | Tumor hypermethylation in CpG island right shore |
| TMEM160 | 54958 | 19 | 52239722 | 52247722 | 0.0003 | Tumor hypermethylation in CpG island right shore |
| PPP1R15A | 23645 | 19 | 54063460 | 54071460 | 0.0009 | Tumor hypermethylation in CpG island right shore |
| DHDH | 27294 | 19 | 54124750 | 54132750 | 0.0003 | Tumor hypermethylation in CpG island right shore |
| LHB | 3972 | 19 | 54208159 | 54216159 | 0.0006 | Tumor hypermethylation in CpG island right shore |
| NR1H2 | 7376 | 19 | 55567496 | 55575496 | 0.0012 | Tumor hypermethylation in CpG island right shore |
| SNAR-F | 1E+08 | 19 | 55796031 | 55804031 | 0.0002 | Tumor hypermethylation in CpG island right shore |
| EPS8L1 | 54869 | 19 | 60279571 | 60287571 | 0 | Tumor hypermethylation in CpG island right shore |
| SUV420H2 | 84787 | 19 | 60539032 | 60547032 | 0.0055 | Tumor hypermethylation in CpG island right shore |
| SHISA7 | 729956 | 19 | 60642042 | 60650042 | 0.0058 | Tumor hypermethylation in CpG island right shore |
| ZNF579 | 163033 | 19 | 60780023 | 60788023 | 0 | Tumor hypermethylation in CpG island right shore |
| ZNF784 | 147808 | 19 | 60823753 | 60831753 | 0.002 | Tumor hypermethylation in CpG island right shore |
| ZBTB45 | 84878 | 19 | 63718733 | 63726733 | 0.0006 | Tumor hypermethylation in CpG island right shore |
| FAM84A | 151354 | 2 | 14686260 | 14694260 | 0 | Tumor hypermethylation in CpG island right shore |
| ZNF513 | 130557 | 2 | 27453097 | 27461097 | 0.0098 | Tumor hypermethylation in CpG island right shore |
| EPAS1 | 2034 | 2 | 46374044 | 46382044 | 0.006 | Tumor hypermethylation in CpG island right shore |
| FBXO41 | 150726 | 2 | 73347551 | 73355551 | 0.0099 | Tumor hypermethylation in CpG island right shore |
| EGR4 | 1961 | 2 | 73370337 | 73378337 | 0 | Tumor hypermethylation in CpG island right shore |
| C2orf81 | 388963 | 2 | 74494352 | 74502352 | 0 | Tumor hypermethylation in CpG island right shore |
| LBX2 | 85474 | 2 | 74579951 | 74587951 | 0.0033 | Tumor hypermethylation in CpG island right shore |
| CD8A | 925 | 2 | 86868348 | 86876348 | 0 | Tumor hypermethylation in CpG island right shore |
| RGPD4 | 285190 | 2 | 107805819 | 107813819 | 0.004 | Tumor hypermethylation in CpG island right shore |
| PAX8 | 7849 | 2 | 113748968 | 113756968 | 0.0005 | Tumor hypermethylation in CpG island right shore |
| C1QL2 | 165257 | 2 | 119628941 | 119636941 | 0.0002 | Tumor hypermethylation in CpG island right shore |
| KIAA0319 | 9856 | 2 | 172671724 | 172679724 | 0 | Tumor hypermethylation in CpG island right shore |
| EVX2 | 344191 | 2 | 176652936 | 176660936 | 0 | Tumor hypermethylation in CpG island right shore |
| NRP2 | 8828 | 2 | 206251468 | 206259468 | 0.0086 | Tumor hypermethylation in CpG island right shore |
| CDK5R2 | 8941 | 2 | 219528641 | 219536641 | 0.0001 | Tumor hypermethylation in CpG island right shore |
| FEV | 54738 | 2 | 219554623 | 219562623 | 0 | Tumor hypermethylation in CpG island right shore |
| B3GNT7 | 93010 | 2 | 231964578 | 231972578 | 0.0006 | Tumor hypermethylation in CpG island right shore |
| KLHL30 | 377007 | 2 | 238708101 | 238716101 | 0.0062 | Tumor hypermethylation in CpG island right shore |
| BOK | 666 | 2 | 242142864 | 242150864 | 0.0019 | Tumor hypermethylation in CpG island right shore |
| GFRA4 | 64096 | 20 | 3588046 | 3596046 | 0.0008 | Tumor hypermethylation in CpG island right shore |
| C20orf29 | 55317 | 20 | 3745202 | 3753202 | 0.0078 | Tumor hypermethylation in CpG island right shore |
| BMP2 | 650 | 20 | 6692744 | 6700744 | 0.0015 | Tumor hypermethylation in CpG island right shore |
| KCNS1 | 3787 | 20 | 43159167 | 43167167 | 0.0032 | Tumor hypermethylation in CpG island right shore |
| MMP9 | 4318 | 20 | 44066953 | 44074953 | 0 | Tumor hypermethylation in CpG island right shore |
| SALL4 | 57167 | 20 | 49848455 | 49856455 | 0.004 | Tumor hypermethylation in CpG island right shore |
| HRH3 | 11255 | 20 | 60224718 | 60232718 | 0.0016 | Tumor hypermethylation in CpG island right shore |
| C20orf20 | 55257 | 20 | 60894249 | 60902249 | 0.0007 | Tumor hypermethylation in CpG island right shore |
| C20orf195 | 79025 | 20 | 61650816 | 61658816 | 0 | Tumor hypermethylation in CpG island right shore |
| PRIC285 | 85441 | 20 | 61665551 | 61673551 | 0 | Tumor hypermethylation in CpG island right shore |
| RTEL1 | 51750 | 20 | 61756090 | 61764090 | 0.0003 | Tumor hypermethylation in CpG island right shore |
| ZNF512B | 57473 | 20 | 62067662 | 62075662 | 0 | Tumor hypermethylation in CpG island right shore |
| C20orf201 | 198437 | 20 | 62182156 | 62190156 | 0.0042 | Tumor hypermethylation in CpG island right shore |
| CRYAA | 1409 | 21 | 43458209 | 43466209 | 0.0001 | Tumor hypermethylation in CpG island right shore |
| C21orf29 | 54084 | 21 | 44951923 | 44959923 | 0.0012 | Tumor hypermethylation in CpG island right shore |
| COL6A1 | 1291 | 21 | 46222090 | 46230090 | 0.0004 | Tumor hypermethylation in CpG island right shore |
| C21orf56 | 84221 | 21 | 46424801 | 46432801 | 0.0001 | Tumor hypermethylation in CpG island right shore |
| SLC25A1 | 6576 | 22 | 17542301 | 17550301 | 0.0099 | Tumor hypermethylation in CpG island right shore |
| CLDN5 | 7122 | 22 | 17888860 | 17896860 | 0.0053 | Tumor hypermethylation in CpG island right shore |
| GP1BB | 2812 | 22 | 18087065 | 18095065 | 0 | Tumor hypermethylation in CpG island right shore |
| MN1 | 4330 | 22 | 26523486 | 26531486 | 0 | Tumor hypermethylation in CpG island right shore |
| RASL10A | 10633 | 22 | 28037748 | 28045748 | 0 | Tumor hypermethylation in CpG island right shore |
| SMTN | 6525 | 22 | 29803304 | 29811304 | 0.0014 | Tumor hypermethylation in CpG island right shore |
| SELM | 140606 | 22 | 29829551 | 29837551 | 0.0024 | Tumor hypermethylation in CpG island right shore |
| SLC16A8 | 23539 | 22 | 36805116 | 36813116 | 0.0025 | Tumor hypermethylation in CpG island right shore |
| PHF21B | 112885 | 22 | 43780473 | 43788473 | 0.0009 | Tumor hypermethylation in CpG island right shore |
| CELSR1 | 9620 | 22 | 45307731 | 45315731 | 0 | Tumor hypermethylation in CpG island right shore |
| MAPK12 | 6300 | 22 | 49038216 | 49046216 | 0.0076 | Tumor hypermethylation in CpG island right shore |
| MAPK11 | 5600 | 22 | 49046906 | 49054906 | 0.0069 | Tumor hypermethylation in CpG island right shore |
| LMF2 | 91289 | 22 | 49289001 | 49297001 | 0.0001 | Tumor hypermethylation in CpG island right shore |
| MAPK8IP2 | 23542 | 22 | 49381996 | 49389996 | 0.0016 | Tumor hypermethylation in CpG island right shore |
| VGLL4 | 9686 | 3 | 11581398 | 11589398 | 0.0002 | Tumor hypermethylation in CpG island right shore |
| GNAT1 | 2779 | 3 | 50200046 | 50208046 | 0 | Tumor hypermethylation in CpG island right shore |
| PTPRG | 5793 | 3 | 61518282 | 61526282 | 0 | Tumor hypermethylation in CpG island right shore |
| FEZF2 | 55079 | 3 | 62330230 | 62338230 | 0 | Tumor hypermethylation in CpG island right shore |
| ADAMTS9 | 56999 | 3 | 64644405 | 64652405 | 0 | Tumor hypermethylation in CpG island right shore |
| FOXL2 | 668 | 3 | 140144672 | 140152672 | 0 | Tumor hypermethylation in CpG island right shore |
| FAM131A | 131408 | 3 | 185532410 | 185540410 | 0.0006 | Tumor hypermethylation in CpG island right shore |
| GP5 | 2814 | 3 | 195597284 | 195605284 | 0.0002 | Tumor hypermethylation in CpG island right shore |
| TMEM44 | 93109 | 3 | 195831439 | 195839439 | 0.0033 | Tumor hypermethylation in CpG island right shore |
| FAM43A | 131583 | 3 | 195883910 | 195891910 | 0.0001 | Tumor hypermethylation in CpG island right shore |
| TNK2 | 10188 | 3 | 197102829 | 197110829 | 0.0013 | Tumor hypermethylation in CpG island right shore |
| PIGZ | 80235 | 3 | 198176101 | 198184101 | 0.0051 | Tumor hypermethylation in CpG island right shore |
| MFI2 | 4241 | 3 | 198237083 | 198245083 | 0.0085 | Tumor hypermethylation in CpG island right shore |
| MFSD7 | 84179 | 4 | 668973 | 676973 | 0 | Tumor hypermethylation in CpG island right shore |
| IDUA | 3425 | 4 | 966784 | 974784 | 0 | Tumor hypermethylation in CpG island right shore |
| SLC26A1 | 10861 | 4 | 973183 | 981183 | 0 | Tumor hypermethylation in CpG island right shore |
| FGFRL1 | 53834 | 4 | 991609 | 999609 | 0.0004 | Tumor hypermethylation in CpG island right shore |
| MFSD10 | 10227 | 4 | 2901762 | 2909762 | 0.0002 | Tumor hypermethylation in CpG island right shore |
| GRIA2 | 2891 | 4 | 158357185 | 158365185 | 0.0004 | Tumor hypermethylation in CpG island right shore |
| SLC12A7 | 10723 | 5 | 1161172 | 1169172 | 0.0003 | Tumor hypermethylation in CpG island right shore |
| RXFP3 | 51289 | 5 | 33968247 | 33976247 | 0.0015 | Tumor hypermethylation in CpG island right shore |
| ATF1 | 2668 | 5 | 37866655 | 37874655 | 0 | Tumor hypermethylation in CpG island right shore |
| SHROOM1 | 134549 | 5 | 132185901 | 132193901 | 0.0066 | Tumor hypermethylation in CpG island right shore |
| PITX1 | 5307 | 5 | 134393863 | 134401863 | 0.003 | Tumor hypermethylation in CpG island right shore |
| EGR1 | 1958 | 5 | 137825079 | 137833079 | 0.0019 | Tumor hypermethylation in CpG island right shore |
| PCDHA6 | 56142 | 5 | 140183833 | 140191833 | 0.0096 | Tumor hypermethylation in CpG island right shore |
| PCDHA9 | 9752 | 5 | 140203540 | 140211540 | 0.0048 | Tumor hypermethylation in CpG island right shore |
| PCDHA11 | 56138 | 5 | 140224014 | 140232014 | 0.002 | Tumor hypermethylation in CpG island right shore |
| PCDHA12 | 56137 | 5 | 140231114 | 140239114 | 0.0049 | Tumor hypermethylation in CpG island right shore |
| PCDHB7 | 56129 | 5 | 140528426 | 140536426 | 0.0053 | Tumor hypermethylation in CpG island right shore |
| PCDHB18 | 54660 | 5 | 140590121 | 140598121 | 0.0019 | Tumor hypermethylation in CpG island right shore |
| PCDHGB3 | 56102 | 5 | 140726145 | 140734145 | 0.0006 | Tumor hypermethylation in CpG island right shore |
| MGAT4B | 11282 | 5 | 179158481 | 179166481 | 0.0019 | Tumor hypermethylation in CpG island right shore |
| HIST1H2AL | 8332 | 6 | 27937085 | 27945085 | 0 | Tumor hypermethylation in CpG island right shore |
| HIST1H2AM | 8336 | 6 | 27964942 | 27972942 | 0.0006 | Tumor hypermethylation in CpG island right shore |
| DDAH2 | 23564 | 6 | 31802018 | 31810018 | 0.0063 | Tumor hypermethylation in CpG island right shore |
| PRRT1 | 80863 | 6 | 32223698 | 32231698 | 0.0002 | Tumor hypermethylation in CpG island right shore |
| MDGA1 | 266727 | 6 | 37769744 | 37777744 | 0.0054 | Tumor hypermethylation in CpG island right shore |
| TFAP2D | 83741 | 6 | 50785215 | 50793215 | 0 | Tumor hypermethylation in CpG island right shore |
| TFAP2B | 7021 | 6 | 50890397 | 50898397 | 0.0001 | Tumor hypermethylation in CpG island right shore |
| FOXO3 | 2309 | 6 | 108983718 | 108991718 | 0.0008 | Tumor hypermethylation in CpG island right shore |
| UNCX | 340260 | 7 | 1235179 | 1243179 | 0 | Tumor hypermethylation in CpG island right shore |
| INTS1 | 26173 | 7 | 1506544 | 1514544 | 0.0007 | Tumor hypermethylation in CpG island right shore |
| PSMG3 | 84262 | 7 | 1572194 | 1580194 | 0.0065 | Tumor hypermethylation in CpG island right shore |
| ACTB | 60 | 7 | 5532758 | 5540758 | 0.0005 | Tumor hypermethylation in CpG island right shore |
| HOXA6 | 3203 | 7 | 27149893 | 27157893 | 0 | Tumor hypermethylation in CpG island right shore |
| MIR196B | 442920 | 7 | 27171707 | 27179707 | 0 | Tumor hypermethylation in CpG island right shore |
| PRR15 | 222171 | 7 | 29565951 | 29573951 | 0.0008 | Tumor hypermethylation in CpG island right shore |
| TNS3 | 64759 | 7 | 47541724 | 47549724 | 0.0061 | Tumor hypermethylation in CpG island right shore |
| GUSB | 2990 | 7 | 65080736 | 65088736 | 0.0047 | Tumor hypermethylation in CpG island right shore |
| FZD1 | 8321 | 7 | 90727718 | 90735718 | 0.0022 | Tumor hypermethylation in CpG island right shore |
| DLX5 | 1749 | 7 | 96488079 | 96496079 | 0 | Tumor hypermethylation in CpG island right shore |
| BRI3 | 81618 | 7 | 97744914 | 97752914 | 0.0011 | Tumor hypermethylation in CpG island right shore |
| GIGYF1 | 64599 | 7 | 100120806 | 100128806 | 0 | Tumor hypermethylation in CpG island right shore |
| ACHE | 43 | 7 | 100327477 | 100335477 | 0.0001 | Tumor hypermethylation in CpG island right shore |
| REPIN1 | 29803 | 7 | 149692811 | 149700811 | 0.0002 | Tumor hypermethylation in CpG island right shore |
| LOC728743 | 728743 | 7 | 149729772 | 149737772 | 0.0032 | Tumor hypermethylation in CpG island right shore |
| SHH | 6469 | 7 | 155293728 | 155301728 | 0 | Tumor hypermethylation in CpG island right shore |
| CSMD1 | 64478 | 8 | 4835736 | 4843736 | 0 | Tumor hypermethylation in CpG island right shore |
| MFHAS1 | 9258 | 8 | 8784541 | 8792541 | 0.0076 | Tumor hypermethylation in CpG island right shore |
| EGR3 | 1960 | 8 | 22602760 | 22610760 | 0 | Tumor hypermethylation in CpG island right shore |
| ZFP41 | 286128 | 8 | 144396483 | 144404483 | 0.0052 | Tumor hypermethylation in CpG island right shore |
| MAFA | 389692 | 8 | 144579719 | 144587719 | 0 | Tumor hypermethylation in CpG island right shore |
| ZC3H3 | 23144 | 8 | 144690763 | 144698763 | 0.0006 | Tumor hypermethylation in CpG island right shore |
| TSTA3 | 7264 | 8 | 144766875 | 144774875 | 0.0013 | Tumor hypermethylation in CpG island right shore |
| MIR937 | 1E+08 | 8 | 144963200 | 144971200 | 0 | Tumor hypermethylation in CpG island right shore |
| NRBP2 | 340371 | 8 | 144992188 | 145000188 | 0 | Tumor hypermethylation in CpG island right shore |
| EPPK1 | 83481 | 8 | 145015422 | 145023422 | 0 | Tumor hypermethylation in CpG island right shore |
| GRINA | 2907 | 8 | 145132213 | 145140213 | 0.0047 | Tumor hypermethylation in CpG island right shore |
| OPLAH | 26873 | 8 | 145183572 | 145191572 | 0 | Tumor hypermethylation in CpG island right shore |
| SCXA | 1E+08 | 8 | 145457410 | 145465410 | 0.0003 | Tumor hypermethylation in CpG island right shore |
| FOXH1 | 8928 | 8 | 145668526 | 145676526 | 0 | Tumor hypermethylation in CpG island right shore |
| FOXD4 | 2298 | 9 | 104417 | 112417 | 0.0077 | Tumor hypermethylation in CpG island right shore |
| SLC24A4 | 123041 | 9 | 19772926 | 19780926 | 0.0002 | Tumor hypermethylation in CpG island right shore |
| CA9 | 768 | 9 | 35659914 | 35667914 | 0.004 | Tumor hypermethylation in CpG island right shore |
| NFIL3 | 4783 | 9 | 93221965 | 93229965 | 0.0006 | Tumor hypermethylation in CpG island right shore |
| NR4A3 | 8013 | 9 | 101619957 | 101627957 | 0.0032 | Tumor hypermethylation in CpG island right shore |
| PAPPA | 5069 | 9 | 117951891 | 117959891 | 0.0099 | Tumor hypermethylation in CpG island right shore |
| LHX6 | 26468 | 9 | 124026840 | 124034840 | 0 | Tumor hypermethylation in CpG island right shore |
| GPR144 | 347088 | 9 | 126249243 | 126257243 | 0.0023 | Tumor hypermethylation in CpG island right shore |
| ZDHHC12 | 84885 | 9 | 130522229 | 130530229 | 0.0021 | Tumor hypermethylation in CpG island right shore |
| ASB6 | 140459 | 9 | 131440265 | 131448265 | 0.0054 | Tumor hypermethylation in CpG island right shore |
| NTNG2 | 84628 | 9 | 134023154 | 134031154 | 0.0007 | Tumor hypermethylation in CpG island right shore |
| SLC2A6 | 11182 | 9 | 135330097 | 135338097 | 0.0077 | Tumor hypermethylation in CpG island right shore |
| C9orf69 | 90120 | 9 | 138146552 | 138154552 | 0 | Tumor hypermethylation in CpG island right shore |
| DNLZ | 728489 | 9 | 138374062 | 138382062 | 0 | Tumor hypermethylation in CpG island right shore |
| FBXW5 | 54461 | 9 | 138954994 | 138962994 | 0 | Tumor hypermethylation in CpG island right shore |
| C9orf142 | 286257 | 9 | 139002690 | 139010690 | 0.0002 | Tumor hypermethylation in CpG island right shore |
| FUT7 | 2529 | 9 | 139043113 | 139051113 | 0.0041 | Tumor hypermethylation in CpG island right shore |
| UAP1L1 | 91373 | 9 | 139087773 | 139095773 | 0.0015 | Tumor hypermethylation in CpG island right shore |
| DPP7 | 29952 | 9 | 139125016 | 139133016 | 0 | Tumor hypermethylation in CpG island right shore |
| ANAPC2 | 29882 | 9 | 139198878 | 139206878 | 0.0004 | Tumor hypermethylation in CpG island right shore |
| RNF208 | 727800 | 9 | 139231596 | 139239596 | 0.0022 | Tumor hypermethylation in CpG island right shore |
| C9orf167 | 54863 | 9 | 139288100 | 139296100 | 0 | Tumor hypermethylation in CpG island right shore |
| KLHL15 | 80311 | X | 23951224 | 23959224 | 0.0023 | Tumor hypermethylation in CpG island right shore |
| ARX | 170302 | X | 24939986 | 24947986 | 0 | Tumor hypermethylation in CpG island right shore |
| KCND1 | 3750 | X | 48709195 | 48717195 | 0.0076 | Tumor hypermethylation in CpG island right shore |
| PRAF2 | 11230 | X | 48814606 | 48822606 | 0.0072 | Tumor hypermethylation in CpG island right shore |
| CACNA1F | 778 | X | 48972777 | 48980777 | 0.0075 | Tumor hypermethylation in CpG island right shore |
| HUWE1 | 10075 | X | 53726398 | 53734398 | 0.0036 | Tumor hypermethylation in CpG island right shore |
| PCDH11X | 27328 | X | 90916915 | 90924915 | 0.0014 | Tumor hypermethylation in CpG island right shore |
| PCDH19 | 57575 | X | 99547927 | 99555927 | 0 | Tumor hypermethylation in CpG island right shore |
| RBMXL3 | 139804 | X | 114326218 | 114334218 | 0 | Tumor hypermethylation in CpG island right shore |
| PLXNA3 | 55558 | X | 153335816 | 153343816 | 0.0001 | Tumor hypermethylation in CpG island right shore |
| MIR200B | 406984 | 1 | 1088346 | 1096346 | 0 | Tumor hypermethylation in CpG island both shores |
| TNFRSF18 | 8784 | 1 | 1127952 | 1135952 | 0.0006 | Tumor hypermethylation in CpG island both shores |
| TNFRSF4 | 7293 | 1 | 1135375 | 1143375 | 0 | Tumor hypermethylation in CpG island both shores |
| SDF4 | 51150 | 1 | 1153310 | 1161310 | 0 | Tumor hypermethylation in CpG island both shores |
| ACAP3 | 116983 | 1 | 1229132 | 1237132 | 0.0008 | Tumor hypermethylation in CpG island both shores |
| CPSF3L | 54973 | 1 | 1245909 | 1253909 | 0.0007 | Tumor hypermethylation in CpG island both shores |
| TAS1R3 | 83756 | 1 | 1252588 | 1260588 | 0 | Tumor hypermethylation in CpG island both shores |
| VWA1 | 64856 | 1 | 1356765 | 1364765 | 0.0043 | Tumor hypermethylation in CpG island both shores |
| C1orf70 | 339453 | 1 | 1461603 | 1469603 | 0 | Tumor hypermethylation in CpG island both shores |
| SSU72 | 29101 | 1 | 1496125 | 1504125 | 0.0088 | Tumor hypermethylation in CpG island both shores |
| DMRTA2 | 63950 | 1 | 50657729 | 50665729 | 0 | Tumor hypermethylation in CpG island both shores |
| FOXD3 | 27022 | 1 | 63557317 | 63565317 | 0 | Tumor hypermethylation in CpG island both shores |
| TCHH | 7062 | 1 | 150349180 | 150357180 | 0 | Tumor hypermethylation in CpG island both shores |
| SLC27A3 | 11000 | 1 | 152010391 | 152018391 | 0.0016 | Tumor hypermethylation in CpG island both shores |
| C10orf114 | 399726 | 10 | 21822219 | 21830219 | 0.0007 | Tumor hypermethylation in CpG island both shores |
| CCAR1 | 55749 | 10 | 70146976 | 70154976 | 0.0092 | Tumor hypermethylation in CpG island both shores |
| NKX2-3 | 159296 | 10 | 101278679 | 101286679 | 0 | Tumor hypermethylation in CpG island both shores |
| PAX2 | 5076 | 10 | 102491457 | 102499457 | 0 | Tumor hypermethylation in CpG island both shores |
| CALHM2 | 51063 | 10 | 105198152 | 105206152 | 0.0093 | Tumor hypermethylation in CpG island both shores |
| VAX1 | 11023 | 10 | 118883802 | 118891802 | 0 | Tumor hypermethylation in CpG island both shores |
| HMX2 | 3167 | 10 | 124893627 | 124901627 | 0 | Tumor hypermethylation in CpG island both shores |
| KNDC1 | 85442 | 10 | 134819960 | 134827960 | 0.0066 | Tumor hypermethylation in CpG island both shores |
| PKP3 | 11187 | 11 | 380216 | 388216 | 0 | Tumor hypermethylation in CpG island both shores |
| C11orf35 | 256329 | 11 | 546779 | 554779 | 0.0006 | Tumor hypermethylation in CpG island both shores |
| IRF7 | 3665 | 11 | 601999 | 609999 | 0.0002 | Tumor hypermethylation in CpG island both shores |
| MUPCDH | 53841 | 11 | 611007 | 619007 | 0 | Tumor hypermethylation in CpG island both shores |
| CEND1 | 51286 | 11 | 776126 | 784126 | 0.0013 | Tumor hypermethylation in CpG island both shores |
| EFCAB4A | 283229 | 11 | 813584 | 821584 | 0 | Tumor hypermethylation in CpG island both shores |
| POLR2L | 5441 | 11 | 828529 | 836529 | 0.0048 | Tumor hypermethylation in CpG island both shores |
| H19 | 283120 | 11 | 1971641 | 1979641 | 0 | Tumor hypermethylation in CpG island both shores |
| DBX1 | 120237 | 11 | 20134446 | 20142446 | 0 | Tumor hypermethylation in CpG island both shores |
| SLC6A5 | 9152 | 11 | 20573521 | 20581521 | 0 | Tumor hypermethylation in CpG island both shores |
| ARFGAP2 | 84364 | 11 | 47150995 | 47158995 | 0.0068 | Tumor hypermethylation in CpG island both shores |
| EML3 | 256364 | 11 | 62132813 | 62140813 | 0.0047 | Tumor hypermethylation in CpG island both shores |
| GANAB | 23193 | 11 | 62166680 | 62174680 | 0.0092 | Tumor hypermethylation in CpG island both shores |
| LRRN4CL | 221091 | 11 | 62209776 | 62217776 | 0.0018 | Tumor hypermethylation in CpG island both shores |
| C11orf95 | 65998 | 11 | 63288689 | 63296689 | 0.0009 | Tumor hypermethylation in CpG island both shores |
| TRPT1 | 83707 | 11 | 63746302 | 63754302 | 0.0011 | Tumor hypermethylation in CpG island both shores |
| MAP4K2 | 5871 | 11 | 64323289 | 64331289 | 0.0075 | Tumor hypermethylation in CpG island both shores |
| ADRBK1 | 156 | 11 | 66786480 | 66794480 | 0.0094 | Tumor hypermethylation in CpG island both shores |
| ANKRD13D | 338692 | 11 | 66809337 | 66817337 | 0.0021 | Tumor hypermethylation in CpG island both shores |
| PTPRCAP | 5790 | 11 | 66957729 | 66965729 | 0 | Tumor hypermethylation in CpG island both shores |
| TCIRG1 | 10312 | 11 | 67559058 | 67567058 | 0.0002 | Tumor hypermethylation in CpG island both shores |
| SUV420H1 | 51111 | 11 | 67733360 | 67741360 | 0.0016 | Tumor hypermethylation in CpG island both shores |
| FLI1 | 2313 | 11 | 128063598 | 128071598 | 0 | Tumor hypermethylation in CpG island both shores |
| PTHLH | 5744 | 12 | 28012183 | 28020183 | 0.0012 | Tumor hypermethylation in CpG island both shores |
| HOXC12 | 3228 | 12 | 52630980 | 52638980 | 0 | Tumor hypermethylation in CpG island both shores |
| HOXC5 | 3222 | 12 | 52692908 | 52700908 | 0.0001 | Tumor hypermethylation in CpG island both shores |
| NDUFA4L2 | 56901 | 12 | 55916742 | 55924742 | 0.0001 | Tumor hypermethylation in CpG island both shores |
| SH2B3 | 10019 | 12 | 110324134 | 110332134 | 0.0043 | Tumor hypermethylation in CpG island both shores |
| KDM2B | 84678 | 12 | 120498747 | 120506747 | 0.0007 | Tumor hypermethylation in CpG island both shores |
| ARL6IP4 | 51329 | 12 | 122026832 | 122034832 | 0.0064 | Tumor hypermethylation in CpG island both shores |
| CDK2AP1 | 8099 | 12 | 122318640 | 122326640 | 0 | Tumor hypermethylation in CpG island both shores |
| FGF9 | 2254 | 13 | 21139214 | 21147214 | 0.0008 | Tumor hypermethylation in CpG island both shores |
| PDX1 | 8050 | 13 | 27388167 | 27396167 | 0 | Tumor hypermethylation in CpG island both shores |
| ZIC5 | 85416 | 13 | 99418179 | 99426179 | 0 | Tumor hypermethylation in CpG island both shores |
| CDH24 | 64403 | 14 | 22592587 | 22600587 | 0.0043 | Tumor hypermethylation in CpG island both shores |
| JPH4 | 84502 | 14 | 23113849 | 23121849 | 0 | Tumor hypermethylation in CpG island both shores |
| C14orf23 | 387978 | 14 | 28307660 | 28315660 | 0.0002 | Tumor hypermethylation in CpG island both shores |
| NKX2-1 | 7080 | 14 | 36055181 | 36063181 | 0 | Tumor hypermethylation in CpG island both shores |
| NKX2-8 | 26257 | 14 | 36117537 | 36125537 | 0 | Tumor hypermethylation in CpG island both shores |
| FOXA1 | 3169 | 14 | 37130240 | 37138240 | 0 | Tumor hypermethylation in CpG island both shores |
| OTX2OS1 | 1E+08 | 14 | 56345653 | 56353653 | 0 | Tumor hypermethylation in CpG island both shores |
| GSC | 145258 | 14 | 94302252 | 94310252 | 0 | Tumor hypermethylation in CpG island both shores |
| XRCC3 | 7517 | 14 | 103247576 | 103255576 | 0.001 | Tumor hypermethylation in CpG island both shores |
| ADSSL1 | 122622 | 14 | 104257578 | 104265578 | 0.002 | Tumor hypermethylation in CpG island both shores |
| NUDT14 | 256281 | 14 | 104714705 | 104722705 | 0.0063 | Tumor hypermethylation in CpG island both shores |
| CRIP2 | 1397 | 14 | 105008175 | 105016175 | 0.0084 | Tumor hypermethylation in CpG island both shores |
| DUOX2 | 50506 | 15 | 43189651 | 43197651 | 0 | Tumor hypermethylation in CpG island both shores |
| DUOXA2 | 405753 | 15 | 43189815 | 43197815 | 0 | Tumor hypermethylation in CpG island both shores |
| LBXCOR1 | 390598 | 15 | 65900994 | 65908994 | 0 | Tumor hypermethylation in CpG island both shores |
| MRPL28 | 51263 | 16 | 356541 | 364541 | 0 | Tumor hypermethylation in CpG island both shores |
| TMEM8A | 58986 | 16 | 367951 | 375951 | 0 | Tumor hypermethylation in CpG island both shores |
| PIGQ | 9091 | 16 | 556004 | 564004 | 0.0004 | Tumor hypermethylation in CpG island both shores |
| WDR90 | 197335 | 16 | 635363 | 643363 | 0 | Tumor hypermethylation in CpG island both shores |
| RHOT2 | 89941 | 16 | 654133 | 662133 | 0 | Tumor hypermethylation in CpG island both shores |
| STUB1 | 10273 | 16 | 666115 | 674115 | 0 | Tumor hypermethylation in CpG island both shores |
| FAM173A | 65990 | 16 | 707158 | 715158 | 0.0023 | Tumor hypermethylation in CpG island both shores |
| HAGHL | 84264 | 16 | 712958 | 720958 | 0 | Tumor hypermethylation in CpG island both shores |
| RPUSD1 | 113000 | 16 | 774384 | 782384 | 0 | Tumor hypermethylation in CpG island both shores |
| GNG13 | 51764 | 16 | 786734 | 794734 | 0.0008 | Tumor hypermethylation in CpG island both shores |
| PRR25 | 388199 | 16 | 791443 | 799443 | 0.0065 | Tumor hypermethylation in CpG island both shores |
| LMF1 | 64788 | 16 | 956985 | 964985 | 0 | Tumor hypermethylation in CpG island both shores |
| CACNA1H | 8912 | 16 | 1139241 | 1147241 | 0.0001 | Tumor hypermethylation in CpG island both shores |
| BAIAP3 | 8938 | 16 | 1320663 | 1328663 | 0 | Tumor hypermethylation in CpG island both shores |
| C16orf42 | 115939 | 16 | 1337874 | 1345874 | 0 | Tumor hypermethylation in CpG island both shores |
| C16orf91 | 283951 | 16 | 1415346 | 1423346 | 0 | Tumor hypermethylation in CpG island both shores |
| CCDC154 | 645811 | 16 | 1430491 | 1438491 | 0 | Tumor hypermethylation in CpG island both shores |
| TMEM204 | 79652 | 16 | 1520231 | 1528231 | 0 | Tumor hypermethylation in CpG island both shores |
| NME3 | 4832 | 16 | 1757711 | 1765711 | 0 | Tumor hypermethylation in CpG island both shores |
| SEPX1 | 51734 | 16 | 1929295 | 1937295 | 0.0005 | Tumor hypermethylation in CpG island both shores |
| TBL3 | 10607 | 16 | 1958064 | 1966064 | 0 | Tumor hypermethylation in CpG island both shores |
| SLC9A3R2 | 9351 | 16 | 2012888 | 2020888 | 0.0004 | Tumor hypermethylation in CpG island both shores |
| NTHL1 | 4913 | 16 | 2033868 | 2041868 | 0.0013 | Tumor hypermethylation in CpG island both shores |
| MIR1225 | 1E+08 | 16 | 2076286 | 2084286 | 0 | Tumor hypermethylation in CpG island both shores |
| DCI | 1632 | 16 | 2237604 | 2245604 | 0.0084 | Tumor hypermethylation in CpG island both shores |
| C16orf59 | 80178 | 16 | 2446115 | 2454115 | 0.0005 | Tumor hypermethylation in CpG island both shores |
| NUDT16L1 | 84309 | 16 | 4679716 | 4687716 | 0.0001 | Tumor hypermethylation in CpG island both shores |
| CARHSP1 | 23589 | 16 | 8865749 | 8873749 | 0.0027 | Tumor hypermethylation in CpG island both shores |
| ATP2A1 | 487 | 16 | 28793309 | 28801309 | 0.0056 | Tumor hypermethylation in CpG island both shores |
| CD2BP2 | 10421 | 16 | 30270183 | 30278183 | 0.0024 | Tumor hypermethylation in CpG island both shores |
| ZNF747 | 65988 | 16 | 30449695 | 30457695 | 0.0076 | Tumor hypermethylation in CpG island both shores |
| ZNF764 | 92595 | 16 | 30473085 | 30481085 | 0.0002 | Tumor hypermethylation in CpG island both shores |
| PYDC1 | 260434 | 16 | 31131896 | 31139896 | 0.0013 | Tumor hypermethylation in CpG island both shores |
| CDH15 | 1013 | 16 | 67231651 | 67239651 | 0.0045 | Tumor hypermethylation in CpG island both shores |
| TUBB3 | 10381 | 16 | 88507787 | 88515787 | 0.0014 | Tumor hypermethylation in CpG island both shores |
| KCTD11 | 147040 | 17 | 7191931 | 7199931 | 0.0032 | Tumor hypermethylation in CpG island both shores |
| C17orf44 | 284029 | 17 | 8064086 | 8072086 | 0.0068 | Tumor hypermethylation in CpG island both shores |
| GIT1 | 28964 | 17 | 24936736 | 24944736 | 0.0054 | Tumor hypermethylation in CpG island both shores |
| CORO6 | 84940 | 17 | 24968567 | 24976567 | 0.0071 | Tumor hypermethylation in CpG island both shores |
| LHX1 | 3975 | 17 | 32364611 | 32372611 | 0 | Tumor hypermethylation in CpG island both shores |
| HSD17B1 | 3292 | 17 | 37953509 | 37961509 | 0.0006 | Tumor hypermethylation in CpG island both shores |
| PLEKHH3 | 79990 | 17 | 38078574 | 38086574 | 0.001 | Tumor hypermethylation in CpG island both shores |
| CCR10 | 51554 | 17 | 38083371 | 38091371 | 0.0006 | Tumor hypermethylation in CpG island both shores |
| CNTNAP1 | 8506 | 17 | 38084157 | 38092157 | 0.0002 | Tumor hypermethylation in CpG island both shores |
| MIR10A | 406902 | 17 | 44008308 | 44016308 | 0 | Tumor hypermethylation in CpG island both shores |
| C17orf93 | 360205 | 17 | 44151540 | 44159540 | 0 | Tumor hypermethylation in CpG island both shores |
| DLX4 | 1748 | 17 | 45397560 | 45405560 | 0 | Tumor hypermethylation in CpG island both shores |
| TBX2 | 6909 | 17 | 56828038 | 56836038 | 0 | Tumor hypermethylation in CpG island both shores |
| AXIN2 | 8313 | 17 | 60984202 | 60992202 | 0.005 | Tumor hypermethylation in CpG island both shores |
| SOX9 | 6662 | 17 | 67624755 | 67632755 | 0 | Tumor hypermethylation in CpG island both shores |
| TRIM65 | 201292 | 17 | 71400649 | 71408649 | 0.0001 | Tumor hypermethylation in CpG island both shores |
| SPHK1 | 8877 | 17 | 71888284 | 71896284 | 0.0001 | Tumor hypermethylation in CpG island both shores |
| GAA | 2548 | 17 | 75685949 | 75693949 | 0.0027 | Tumor hypermethylation in CpG island both shores |
| SGSH | 6448 | 17 | 75804794 | 75812794 | 0.005 | Tumor hypermethylation in CpG island both shores |
| FLJ90757 | 440465 | 17 | 76619114 | 76627114 | 0 | Tumor hypermethylation in CpG island both shores |
| BAIAP2 | 10458 | 17 | 76619541 | 76627541 | 0.0002 | Tumor hypermethylation in CpG island both shores |
| C17orf56 | 146705 | 17 | 76823450 | 76831450 | 0.0001 | Tumor hypermethylation in CpG island both shores |
| ARL16 | 339231 | 17 | 77257359 | 77265359 | 0.0001 | Tumor hypermethylation in CpG island both shores |
| MRPL12 | 6182 | 17 | 77276811 | 77284811 | 0 | Tumor hypermethylation in CpG island both shores |
| NPB | 256933 | 17 | 77449363 | 77457363 | 0.0085 | Tumor hypermethylation in CpG island both shores |
| SIRT7 | 51547 | 17 | 77465332 | 77473332 | 0.0063 | Tumor hypermethylation in CpG island both shores |
| PYCR1 | 5831 | 17 | 77484259 | 77492259 | 0.0007 | Tumor hypermethylation in CpG island both shores |
| STRA13 | 201254 | 17 | 77570062 | 77578062 | 0.0005 | Tumor hypermethylation in CpG island both shores |
| LRRC45 | 201255 | 17 | 77570568 | 77578568 | 0.0022 | Tumor hypermethylation in CpG island both shores |
| NCOA3 | 8202 | 17 | 77578820 | 77586820 | 0.0002 | Tumor hypermethylation in CpG island both shores |
| RFNG | 5986 | 17 | 77598939 | 77606939 | 0 | Tumor hypermethylation in CpG island both shores |
| FASN | 2194 | 17 | 77645395 | 77653395 | 0.0026 | Tumor hypermethylation in CpG island both shores |
| SECTM1 | 6398 | 17 | 77881210 | 77889210 | 0.0002 | Tumor hypermethylation in CpG island both shores |
| FN3KRP | 79672 | 17 | 78263870 | 78271870 | 0.0054 | Tumor hypermethylation in CpG island both shores |
| METRNL | 284207 | 17 | 78626855 | 78634855 | 0.0006 | Tumor hypermethylation in CpG island both shores |
| GATA6 | 2627 | 18 | 17999413 | 18007413 | 0 | Tumor hypermethylation in CpG island both shores |
| KCTD1 | 284252 | 18 | 22379397 | 22387397 | 0.0024 | Tumor hypermethylation in CpG island both shores |
| SHC2 | 25759 | 19 | 407996 | 415996 | 0.0003 | Tumor hypermethylation in CpG island both shores |
| CDC34 | 997 | 19 | 478732 | 486732 | 0.0085 | Tumor hypermethylation in CpG island both shores |
| FSTL3 | 10272 | 19 | 623388 | 631388 | 0.0017 | Tumor hypermethylation in CpG island both shores |
| CFD | 200576 | 19 | 806664 | 814664 | 0.0001 | Tumor hypermethylation in CpG island both shores |
| HMHA1 | 23526 | 19 | 1014173 | 1022173 | 0.0001 | Tumor hypermethylation in CpG island both shores |
| GPX4 | 2879 | 19 | 1050935 | 1058935 | 0.0064 | Tumor hypermethylation in CpG island both shores |
| SBNO2 | 22904 | 19 | 1121282 | 1129282 | 0 | Tumor hypermethylation in CpG island both shores |
| ATP5D | 513 | 19 | 1188748 | 1196748 | 0.0016 | Tumor hypermethylation in CpG island both shores |
| C19orf24 | 55009 | 19 | 1222519 | 1230519 | 0.0001 | Tumor hypermethylation in CpG island both shores |
| RPS15 | 6209 | 19 | 1385362 | 1393362 | 0.0001 | Tumor hypermethylation in CpG island both shores |
| C19orf25 | 148223 | 19 | 1426228 | 1434228 | 0.0001 | Tumor hypermethylation in CpG island both shores |
| ATP8B3 | 148229 | 19 | 1759270 | 1767270 | 0.0001 | Tumor hypermethylation in CpG island both shores |
| REXO1 | 57455 | 19 | 1795452 | 1803452 | 0 | Tumor hypermethylation in CpG island both shores |
| FAM108A1 | 81926 | 19 | 1832518 | 1840518 | 0.0041 | Tumor hypermethylation in CpG island both shores |
| SCAMP4 | 113178 | 19 | 1852372 | 1860372 | 0.0056 | Tumor hypermethylation in CpG island both shores |
| CSNK1G2 | 1455 | 19 | 1888160 | 1896160 | 0.0003 | Tumor hypermethylation in CpG island both shores |
| C19orf34 | 255193 | 19 | 1901548 | 1909548 | 0 | Tumor hypermethylation in CpG island both shores |
| MKNK2 | 2872 | 19 | 1998243 | 2006243 | 0.006 | Tumor hypermethylation in CpG island both shores |
| MOBKL2A | 126308 | 19 | 2043269 | 2051269 | 0.0009 | Tumor hypermethylation in CpG island both shores |
| TIMM13 | 26517 | 19 | 2374875 | 2382875 | 0 | Tumor hypermethylation in CpG island both shores |
| DIRAS1 | 148252 | 19 | 2668390 | 2676390 | 0.0044 | Tumor hypermethylation in CpG island both shores |
| TBXA2R | 6915 | 19 | 3553831 | 3561831 | 0.0079 | Tumor hypermethylation in CpG island both shores |
| FEM1A | 56929 | 19 | 4738727 | 4746727 | 0.0083 | Tumor hypermethylation in CpG island both shores |
| RPL36 | 25873 | 19 | 5637271 | 5645271 | 0 | Tumor hypermethylation in CpG island both shores |
| ALKBH7 | 84266 | 19 | 6319443 | 6327443 | 0 | Tumor hypermethylation in CpG island both shores |
| MCOLN1 | 57192 | 19 | 7489495 | 7497495 | 0 | Tumor hypermethylation in CpG island both shores |
| XAB2 | 56949 | 19 | 7596439 | 7604439 | 0.0001 | Tumor hypermethylation in CpG island both shores |
| MAP2K7 | 5609 | 19 | 7870764 | 7878764 | 0.0001 | Tumor hypermethylation in CpG island both shores |
| KEAP1 | 9817 | 19 | 10470481 | 10478481 | 0.0057 | Tumor hypermethylation in CpG island both shores |
| RGL3 | 57139 | 19 | 11387018 | 11395018 | 0.0068 | Tumor hypermethylation in CpG island both shores |
| C19orf43 | 79002 | 19 | 12702529 | 12710529 | 0.0071 | Tumor hypermethylation in CpG island both shores |
| PRDX2 | 7001 | 19 | 12769694 | 12777694 | 0.007 | Tumor hypermethylation in CpG island both shores |
| MAST1 | 23332 | 19 | 12806258 | 12814258 | 0.004 | Tumor hypermethylation in CpG island both shores |
| LYL1 | 4066 | 19 | 13070681 | 13078681 | 0 | Tumor hypermethylation in CpG island both shores |
| MRI1 | 84245 | 19 | 13732336 | 13740336 | 0.0008 | Tumor hypermethylation in CpG island both shores |
| ANO8 | 57719 | 19 | 17302638 | 17310638 | 0.0009 | Tumor hypermethylation in CpG island both shores |
| ARRDC2 | 27106 | 19 | 17968943 | 17976943 | 0.0054 | Tumor hypermethylation in CpG island both shores |
| PIK3R2 | 5296 | 19 | 18121015 | 18129015 | 0.0002 | Tumor hypermethylation in CpG island both shores |
| MPV17L2 | 84769 | 19 | 18161039 | 18169039 | 0.0004 | Tumor hypermethylation in CpG island both shores |
| RAB3A | 5864 | 19 | 18171874 | 18179874 | 0.0087 | Tumor hypermethylation in CpG island both shores |
| SSBP4 | 170463 | 19 | 18387220 | 18395220 | 0.0007 | Tumor hypermethylation in CpG island both shores |
| CILP2 | 148113 | 19 | 19506073 | 19514073 | 0 | Tumor hypermethylation in CpG island both shores |
| KCTD15 | 79047 | 19 | 38975590 | 38983590 | 0.0029 | Tumor hypermethylation in CpG island both shores |
| LGALS7B | 653499 | 19 | 43967689 | 43975689 | 0.0006 | Tumor hypermethylation in CpG island both shores |
| TTC9B | 148014 | 19 | 45412146 | 45420146 | 0.0026 | Tumor hypermethylation in CpG island both shores |
| ZNF575 | 284346 | 19 | 48725179 | 48733179 | 0.0012 | Tumor hypermethylation in CpG island both shores |
| IRGQ | 126298 | 19 | 48788127 | 48796127 | 0.0004 | Tumor hypermethylation in CpG island both shores |
| RPL18 | 6141 | 19 | 53810245 | 53818245 | 0.0032 | Tumor hypermethylation in CpG island both shores |
| FTL | 2512 | 19 | 54156377 | 54164377 | 0.0033 | Tumor hypermethylation in CpG island both shores |
| RPS11 | 6205 | 19 | 54687445 | 54695445 | 0.0092 | Tumor hypermethylation in CpG island both shores |
| PRR12 | 57479 | 19 | 54782723 | 54790723 | 0.0039 | Tumor hypermethylation in CpG island both shores |
| C19orf76 | 199800 | 19 | 54879753 | 54887753 | 0 | Tumor hypermethylation in CpG island both shores |
| POLD1 | 5424 | 19 | 55575404 | 55583404 | 0.001 | Tumor hypermethylation in CpG island both shores |
| LENG8 | 114823 | 19 | 59647876 | 59655876 | 0.0063 | Tumor hypermethylation in CpG island both shores |
| LENG9 | 94059 | 19 | 59662706 | 59670706 | 0.0003 | Tumor hypermethylation in CpG island both shores |
| BRSK1 | 84446 | 19 | 60483345 | 60491345 | 0.0087 | Tumor hypermethylation in CpG island both shores |
| ZNF628 | 89887 | 19 | 60675510 | 60683510 | 0.0038 | Tumor hypermethylation in CpG island both shores |
| SSC5D | 284297 | 19 | 60687681 | 60695681 | 0.0001 | Tumor hypermethylation in CpG island both shores |
| FIZ1 | 84922 | 19 | 60798705 | 60806705 | 0.0003 | Tumor hypermethylation in CpG island both shores |
| ZNF524 | 147807 | 19 | 60799541 | 60807541 | 0 | Tumor hypermethylation in CpG island both shores |
| CCDC106 | 29903 | 19 | 60846765 | 60854765 | 0.0029 | Tumor hypermethylation in CpG island both shores |
| UBE2M | 9040 | 19 | 63758155 | 63766155 | 0.0031 | Tumor hypermethylation in CpG island both shores |
| MEIS1 | 4211 | 2 | 66512035 | 66520035 | 0.0055 | Tumor hypermethylation in CpG island both shores |
| ANKRD39 | 51239 | 2 | 96883483 | 96891483 | 0.0035 | Tumor hypermethylation in CpG island both shores |
| POU3F3 | 5455 | 2 | 104834400 | 104842400 | 0 | Tumor hypermethylation in CpG island both shores |
| LOC440925 | 440925 | 2 | 171275323 | 171283323 | 0.0041 | Tumor hypermethylation in CpG island both shores |
| GAD1 | 2571 | 2 | 171377445 | 171385445 | 0 | Tumor hypermethylation in CpG island both shores |
| MIR10B | 406903 | 2 | 176719276 | 176727276 | 0.0049 | Tumor hypermethylation in CpG island both shores |
| FLJ32063 | 150538 | 2 | 200037065 | 200045065 | 0 | Tumor hypermethylation in CpG island both shores |
| FZD7 | 8324 | 2 | 202603554 | 202611554 | 0.0071 | Tumor hypermethylation in CpG island both shores |
| FZD5 | 7855 | 2 | 208338388 | 208346388 | 0 | Tumor hypermethylation in CpG island both shores |
| PAX3 | 5077 | 2 | 222867944 | 222875944 | 0 | Tumor hypermethylation in CpG island both shores |
| GBX2 | 2637 | 2 | 236737391 | 236745391 | 0 | Tumor hypermethylation in CpG island both shores |
| INSM1 | 3642 | 20 | 20292764 | 20300764 | 0 | Tumor hypermethylation in CpG island both shores |
| PAX1 | 5075 | 20 | 21630296 | 21638296 | 0 | Tumor hypermethylation in CpG island both shores |
| FOXA2 | 3170 | 20 | 22509101 | 22517101 | 0 | Tumor hypermethylation in CpG island both shores |
| ID1 | 3397 | 20 | 29652752 | 29660752 | 0.0015 | Tumor hypermethylation in CpG island both shores |
| TAF4 | 6874 | 20 | 60070261 | 60078261 | 0.0032 | Tumor hypermethylation in CpG island both shores |
| ADRM1 | 11047 | 20 | 60307421 | 60315421 | 0 | Tumor hypermethylation in CpG island both shores |
| C20orf166 | 128826 | 20 | 60554104 | 60562104 | 0.0012 | Tumor hypermethylation in CpG island both shores |
| C20orf200 | 253868 | 20 | 60555213 | 60563213 | 0.0027 | Tumor hypermethylation in CpG island both shores |
| PTK6 | 5753 | 20 | 61635151 | 61643151 | 0 | Tumor hypermethylation in CpG island both shores |
| TNFRSF6B | 8771 | 20 | 61794464 | 61802464 | 0 | Tumor hypermethylation in CpG island both shores |
| SLC2A4RG | 56731 | 20 | 61837654 | 61845654 | 0 | Tumor hypermethylation in CpG island both shores |
| TPD52L2 | 7165 | 20 | 61963033 | 61971033 | 0 | Tumor hypermethylation in CpG island both shores |
| UCKL1 | 54963 | 20 | 62054212 | 62062212 | 0.0026 | Tumor hypermethylation in CpG island both shores |
| OLIG2 | 10215 | 21 | 33316108 | 33324108 | 0 | Tumor hypermethylation in CpG island both shores |
| RUNX1 | 861 | 21 | 35178857 | 35186857 | 0.001 | Tumor hypermethylation in CpG island both shores |
| YDJC | 150223 | 22 | 20310340 | 20318340 | 0.0018 | Tumor hypermethylation in CpG island both shores |
| GAS2L1 | 10634 | 22 | 28028996 | 28036996 | 0.0002 | Tumor hypermethylation in CpG island both shores |
| CYP2D6 | 1565 | 22 | 40852827 | 40860827 | 0.0025 | Tumor hypermethylation in CpG island both shores |
| LOC400931 | 400931 | 22 | 44856540 | 44864540 | 0 | Tumor hypermethylation in CpG island both shores |
| IL17REL | 400935 | 22 | 48789182 | 48797182 | 0.0013 | Tumor hypermethylation in CpG island both shores |
| AMIGO3 | 386724 | 3 | 49728242 | 49736242 | 0.0028 | Tumor hypermethylation in CpG island both shores |
| SOX14 | 8403 | 3 | 138962268 | 138970268 | 0 | Tumor hypermethylation in CpG island both shores |
| DGKQ | 1609 | 4 | 953344 | 961344 | 0.0098 | Tumor hypermethylation in CpG island both shores |
| MSX1 | 4487 | 4 | 4908292 | 4916292 | 0.0057 | Tumor hypermethylation in CpG island both shores |
| PHOX2B | 8929 | 4 | 41441744 | 41449744 | 0 | Tumor hypermethylation in CpG island both shores |
| PDGFRA | 5156 | 4 | 54786020 | 54794020 | 0 | Tumor hypermethylation in CpG island both shores |
| MAB21L2 | 10586 | 4 | 151718526 | 151726526 | 0.0066 | Tumor hypermethylation in CpG island both shores |
| LOC25845 | 25845 | 5 | 522080 | 530080 | 0.0019 | Tumor hypermethylation in CpG island both shores |
| IRX4 | 50805 | 5 | 1931880 | 1939880 | 0 | Tumor hypermethylation in CpG island both shores |
| IRX2 | 153572 | 5 | 2800769 | 2808769 | 0 | Tumor hypermethylation in CpG island both shores |
| OTP | 23440 | 5 | 76966278 | 76974278 | 0 | Tumor hypermethylation in CpG island both shores |
| CNR1 | 56144 | 5 | 140162855 | 140170855 | 0.002 | Tumor hypermethylation in CpG island both shores |
| PCDHB8 | 56128 | 5 | 140533613 | 140541613 | 0.0012 | Tumor hypermethylation in CpG island both shores |
| PCDHB9 | 56127 | 5 | 140543076 | 140551076 | 0.0031 | Tumor hypermethylation in CpG island both shores |
| PCDHB10 | 56126 | 5 | 140548135 | 140556135 | 0.0064 | Tumor hypermethylation in CpG island both shores |
| PCDHB13 | 56123 | 5 | 140569692 | 140577692 | 0.0032 | Tumor hypermethylation in CpG island both shores |
| PCDHB19P | 84054 | 5 | 140595872 | 140603872 | 0.0003 | Tumor hypermethylation in CpG island both shores |
| PCDHB15 | 56121 | 5 | 140601330 | 140609330 | 0.0034 | Tumor hypermethylation in CpG island both shores |
| ME3 | 56110 | 5 | 140720081 | 140728081 | 0.0001 | Tumor hypermethylation in CpG island both shores |
| PCDHGA6 | 56109 | 5 | 140729834 | 140737834 | 0 | Tumor hypermethylation in CpG island both shores |
| PCDHGB4 | 8641 | 5 | 140743635 | 140751635 | 0.004 | Tumor hypermethylation in CpG island both shores |
| PCDHGA8 | 9708 | 5 | 140747666 | 140755666 | 0.0001 | Tumor hypermethylation in CpG island both shores |
| PCDHGB5 | 56101 | 5 | 140753878 | 140761878 | 0.0023 | Tumor hypermethylation in CpG island both shores |
| PCDHGA10 | 56106 | 5 | 140768926 | 140776926 | 0.0004 | Tumor hypermethylation in CpG island both shores |
| PCDHGB7 | 56099 | 5 | 140773465 | 140781465 | 0 | Tumor hypermethylation in CpG island both shores |
| PCDHGA11 | 56105 | 5 | 140776720 | 140784720 | 0 | Tumor hypermethylation in CpG island both shores |
| PCDHGB8P | 56120 | 5 | 140782036 | 140790036 | 0.0004 | Tumor hypermethylation in CpG island both shores |
| PCDHGA12 | 26025 | 5 | 140786341 | 140794341 | 0.0003 | Tumor hypermethylation in CpG island both shores |
| NKX2-5 | 1482 | 5 | 172590921 | 172598921 | 0 | Tumor hypermethylation in CpG island both shores |
| FLJ22536 | 401237 | 6 | 21770653 | 21778653 | 0.0068 | Tumor hypermethylation in CpG island both shores |
| KIAA1949 | 1E+08 | 6 | 30759072 | 30767072 | 0.0079 | Tumor hypermethylation in CpG island both shores |
| ZBTB22 | 9278 | 6 | 33389490 | 33397490 | 0.0032 | Tumor hypermethylation in CpG island both shores |
| COL12A1 | 1303 | 6 | 75968343 | 75976343 | 0.0096 | Tumor hypermethylation in CpG island both shores |
| PRDM13 | 59336 | 6 | 100157370 | 100165370 | 0 | Tumor hypermethylation in CpG island both shores |
| FAM20C | 56975 | 7 | 284051 | 292051 | 0.0029 | Tumor hypermethylation in CpG island both shores |
| CYP2W1 | 54905 | 7 | 985360 | 993360 | 0.0007 | Tumor hypermethylation in CpG island both shores |
| GPER | 2852 | 7 | 1088968 | 1096968 | 0 | Tumor hypermethylation in CpG island both shores |
| HOXA2 | 3199 | 7 | 27104919 | 27112919 | 0.0018 | Tumor hypermethylation in CpG island both shores |
| HOXA3 | 3200 | 7 | 27116141 | 27124141 | 0.001 | Tumor hypermethylation in CpG island both shores |
| HOXA11AS | 221883 | 7 | 27187551 | 27195551 | 0.0001 | Tumor hypermethylation in CpG island both shores |
| EVX1 | 2128 | 7 | 27244688 | 27252688 | 0 | Tumor hypermethylation in CpG island both shores |
| BHLHA15 | 168620 | 7 | 97675501 | 97683501 | 0.0004 | Tumor hypermethylation in CpG island both shores |
| ZNF789 | 285989 | 7 | 98904450 | 98912450 | 0.0037 | Tumor hypermethylation in CpG island both shores |
| C7orf43 | 55262 | 7 | 99590238 | 99598238 | 0.0037 | Tumor hypermethylation in CpG island both shores |
| UFSP1 | 402682 | 7 | 100321275 | 100329275 | 0 | Tumor hypermethylation in CpG island both shores |
| MNX1 | 3110 | 7 | 156490890 | 156498890 | 0.0004 | Tumor hypermethylation in CpG island both shores |
| SOX17 | 64321 | 8 | 55529047 | 55537047 | 0 | Tumor hypermethylation in CpG island both shores |
| LOC401463 | 401463 | 8 | 65648374 | 65656374 | 0.0024 | Tumor hypermethylation in CpG island both shores |
| OSR2 | 116039 | 8 | 100021806 | 100029806 | 0 | Tumor hypermethylation in CpG island both shores |
| SLC45A4 | 57210 | 8 | 142303855 | 142311855 | 0 | Tumor hypermethylation in CpG island both shores |
| ARC | 23237 | 8 | 143688835 | 143696835 | 0.0006 | Tumor hypermethylation in CpG island both shores |
| LY6E | 4061 | 8 | 144167276 | 144175276 | 0.0087 | Tumor hypermethylation in CpG island both shores |
| ZNF696 | 79943 | 8 | 144440933 | 144448933 | 0.0095 | Tumor hypermethylation in CpG island both shores |
| C8orf73 | 642475 | 8 | 144722071 | 144730071 | 0.0001 | Tumor hypermethylation in CpG island both shores |
| NAPRT1 | 93100 | 8 | 144727656 | 144735656 | 0 | Tumor hypermethylation in CpG island both shores |
| EEF1D | 1936 | 8 | 144746988 | 144754988 | 0 | Tumor hypermethylation in CpG island both shores |
| PYCRL | 65263 | 8 | 144758907 | 144766907 | 0.0001 | Tumor hypermethylation in CpG island both shores |
| SCRIB | 23513 | 8 | 144965537 | 144973537 | 0 | Tumor hypermethylation in CpG island both shores |
| PLEC1 | 5339 | 8 | 145084680 | 145092680 | 0 | Tumor hypermethylation in CpG island both shores |
| GPAA1 | 8733 | 8 | 145205511 | 145213511 | 0.0037 | Tumor hypermethylation in CpG island both shores |
| SCRT1 | 83482 | 8 | 145526751 | 145534751 | 0 | Tumor hypermethylation in CpG island both shores |
| FBXL6 | 26233 | 8 | 145548940 | 145556940 | 0.0009 | Tumor hypermethylation in CpG island both shores |
| VPS28 | 51160 | 8 | 145620735 | 145628735 | 0.0038 | Tumor hypermethylation in CpG island both shores |
| NFKBIL2 | 4796 | 8 | 145636620 | 145644620 | 0.0014 | Tumor hypermethylation in CpG island both shores |
| GPT | 2875 | 8 | 145696272 | 145704272 | 0 | Tumor hypermethylation in CpG island both shores |
| MFSD3 | 113655 | 8 | 145701359 | 145709359 | 0 | Tumor hypermethylation in CpG island both shores |
| RECQL4 | 9401 | 8 | 145710018 | 145718018 | 0.0002 | Tumor hypermethylation in CpG island both shores |
| LRRC24 | 441381 | 8 | 145719224 | 145727224 | 0 | Tumor hypermethylation in CpG island both shores |
| C9orf53 | 51198 | 9 | 21953137 | 21961137 | 0 | Tumor hypermethylation in CpG island both shores |
| CDKN2A | 1029 | 9 | 21961038 | 21969038 | 0.0007 | Tumor hypermethylation in CpG island both shores |
| FOXB2 | 442425 | 9 | 78820390 | 78828390 | 0 | Tumor hypermethylation in CpG island both shores |
| BARX1 | 56033 | 9 | 95753429 | 95761429 | 0 | Tumor hypermethylation in CpG island both shores |
| KLF4 | 9314 | 9 | 109287868 | 109295868 | 0 | Tumor hypermethylation in CpG island both shores |
| LHX2 | 9355 | 9 | 125809709 | 125817709 | 0 | Tumor hypermethylation in CpG island both shores |
| INPP5E | 56623 | 9 | 138450077 | 138458077 | 0.0004 | Tumor hypermethylation in CpG island both shores |
| NOTCH1 | 4851 | 9 | 138556059 | 138564059 | 0.0003 | Tumor hypermethylation in CpG island both shores |
| MIR126 | 406913 | 9 | 138680874 | 138688874 | 0.001 | Tumor hypermethylation in CpG island both shores |
| AGPAT2 | 10555 | 9 | 138697732 | 138705732 | 0.0028 | Tumor hypermethylation in CpG island both shores |
| C9orf172 | 389813 | 9 | 138854687 | 138862687 | 0 | Tumor hypermethylation in CpG island both shores |
| CLIC3 | 9022 | 9 | 139006845 | 139014845 | 0.0025 | Tumor hypermethylation in CpG island both shores |
| NPDC1 | 56654 | 9 | 139056497 | 139064497 | 0.0084 | Tumor hypermethylation in CpG island both shores |
| NRARP | 441478 | 9 | 139312524 | 139320524 | 0.0002 | Tumor hypermethylation in CpG island both shores |
| WDR85 | 92715 | 9 | 139589208 | 139597208 | 0.0009 | Tumor hypermethylation in CpG island both shores |
| BCOR | 54880 | X | 39837663 | 39845663 | 0 | Tumor hypermethylation in CpG island both shores |
| ERAS | 3266 | X | 48568226 | 48576226 | 0 | Tumor hypermethylation in CpG island both shores |
| MGC16121 | 84848 | X | 133504326 | 133512326 | 0.0009 | Tumor hypermethylation in CpG island both shores |
| SRPK3 | 26576 | X | 152695649 | 152703649 | 0 | Tumor hypermethylation in CpG island both shores |
| FLNA | 2316 | X | 153252200 | 153260200 | 0 | Tumor hypermethylation in CpG island both shores |
| LOC100190940 | 1E+08 | 12 | 129088840 | 129096840 | 0.0019 | Tumor hypomethylation in CpG island left shore |
| A2LD1 | 87769 | 13 | 99979998 | 99987998 | 0.0052 | Tumor hypomethylation in CpG island left shore |
| KCNG2 | 26251 | 18 | 75720655 | 75728655 | 0.0004 | Tumor hypomethylation in CpG island left shore |
| SULF2 | 55959 | 20 | 45844767 | 45852767 | 0.0061 | Tumor hypomethylation in CpG island left shore |
| UFD1L | 7353 | 22 | 17842738 | 17850738 | 0.0041 | Tumor hypomethylation in CpG island left shore |
| ARSA | 410 | 22 | 49409473 | 49417473 | 0.0018 | Tumor hypomethylation in CpG island left shore |
| ULK4 | 54986 | 3 | 41974664 | 41982664 | 0.007 | Tumor hypomethylation in CpG island left shore |
| CCDC110 | 256309 | 4 | 186625907 | 186633907 | 0.0045 | Tumor hypomethylation in CpG island left shore |
| LMBRD2 | 92255 | 5 | 36183772 | 36191772 | 0.0056 | Tumor hypomethylation in CpG island left shore |
| HBS1L | 10767 | 6 | 135413729 | 135421729 | 0.0093 | Tumor hypomethylation in CpG island left shore |
| ADAMTSL2 | 9719 | 9 | 135385795 | 135393795 | 0.0023 | Tumor hypomethylation in CpG island left shore |
| ACTRT2 | 140625 | 1 | 2923905 | 2931905 | 0.0003 | Tumor hypomethylation in CpG island right shore |
|  | 10801 | 17 | 72785086 | 72793086 | 0.006 | Tumor hypomethylation in CpG island right shore |
| RHBDD1 | 84236 | 2 | 227404914 | 227412914 | 0.0063 | Tumor hypomethylation in CpG island right shore |
| HJURP | 55355 | 2 | 234423951 | 234431951 | 0.002 | Tumor hypomethylation in CpG island right shore |
| RAMP1 | 10267 | 2 | 238428925 | 238436925 | 0 | Tumor hypomethylation in CpG island right shore |
| PKDREJ | 10343 | 22 | 45033883 | 45041883 | 0.0002 | Tumor hypomethylation in CpG island right shore |
| C6orf146 | 222826 | 6 | 4020390 | 4028390 | 0.0035 | Tumor hypomethylation in CpG island right shore |
| CDK5 | 1020 | 7 | 150381985 | 150389985 | 0.0059 | Tumor hypomethylation in CpG island right shore |
| CALML5 | 51806 | 10 | 5527533 | 5535533 | 0.0075 | Tumor hypomethylation in CpG island both shores |
| MGMT | 4255 | 10 | 131151443 | 131159443 | 0.0059 | Tumor hypomethylation in CpG island both shores |
| TSPAN11 | 441631 | 12 | 30967104 | 30975104 | 0.0014 | Tumor hypomethylation in CpG island both shores |
| NUDT7 | 283927 | 16 | 76309911 | 76317911 | 0.0001 | Tumor hypomethylation in CpG island both shores |
| WFDC1 | 58189 | 16 | 82881901 | 82889901 | 0.0051 | Tumor hypomethylation in CpG island both shores |
| TMEM185B | 79134 | 2 | 120693454 | 120701454 | 0.0051 | Tumor hypomethylation in CpG island both shores |
| C20orf85 | 128602 | 20 | 56155388 | 56163388 | 0.0084 | Tumor hypomethylation in CpG island both shores |
| APCDD1L | 164284 | 20 | 56519355 | 56527355 | 0.0038 | Tumor hypomethylation in CpG island both shores |
| PICK1 | 9463 | 22 | 36779207 | 36787207 | 0.0015 | Tumor hypomethylation in CpG island both shores |
| TFRC | 7037 | 3 | 197289429 | 197297429 | 0.008 | Tumor hypomethylation in CpG island both shores |
| CYTL1 | 54360 | 4 | 5068098 | 5076098 | 0.0064 | Tumor hypomethylation in CpG island both shores |
| WDR1 | 9948 | 4 | 9723671 | 9731671 | 0.0012 | Tumor hypomethylation in CpG island both shores |
| C6orf123 | 26238 | 6 | 167936388 | 167944388 | 0.0012 | Tumor hypomethylation in CpG island both shores |
| C6orf208 | 80069 | 6 | 169928093 | 169936093 | 0.0076 | Tumor hypomethylation in CpG island both shores |
| TRRAP | 8295 | 7 | 98310048 | 98318048 | 0.0039 | Tumor hypomethylation in CpG island both shores |

**Table 4.** List of primer sequences

| **Gene** | **Forward** | **Reverse** | **Method** |
| --- | --- | --- | --- |
| MT1A | TCCTGCAAATGCAAAGAGTG | CAGCTGCACTTCTCTGATGC | RT-PCR |
| MT1B | GCAAGAAGTGCTGCTGCTCTT | TCTGATGAGCCTTTGCAGACA | RT-PCR |
| MT1E | AATGGACCCCAACTGCTCTTG | CAGCCCTGGGCACACTTG | RT-PCR |
| MT1F | TGCGCCGCTGGTGTCT | CGCCCCTTTGCAAACACA | RT-PCR |
| MT1G | TCTAGTCTCGCCTCGGGTT | AGGAGCAGCAGCTCTTCTTG | RT-PCR |
| MT1H | TTGCAATGGACCCCAACTG | CAGCCCTGGGCACACTTG | RT-PCR |
| MT1IP | TGCCTGTTCAAGTCTGCTGTGA | AAACACATCCCTGGGCACACT | RT-PCR |
| MT1L | CTCGAAATGGACCCCAACTG | GAGCAGCAGCTCTTCTTGCA | RT-PCR |
| MT1M | GCTTGAGATCTCCAGCCTTACC | TTGCAGGAGGTGCATTTG | RT-PCR |
| MT1X | TCCTGCAAATGCAAAGAGTG | ACAGCTGTCCTGGCATCAG | RT-PCR |

**Table 5.** List of siRNA sequences

| **Gene** | **Type** | **Target sequence** |
| --- | --- | --- |
| MT1F | siRNA | CUGUGAAAUAUGUGAGUGA |
|  | siRNA | GCGACUGAUGCCAGGACAA |
|  | siRNA | GUAAACAGAGAGACAUGUA |
|  | siRNA | UAUACCACCUUGACCCAUU |
| MT1M | siRNA | GUUAAUAGAACAAGCUGCA |
|  | siRNA | UGCAAAGGGACGUUGGAGA |
|  | siRNA | GGACGUUGGAGAACUGCAG |
|  | siRNA | AAGGGACGUUGGAGAACUG |
